# Network Pharmacology and Metabolomics Studies on Antimigraine Mechanisms of Da Chuan Xiong Fang (DCXF)

**DOI:** 10.1155/2021/6665137

**Published:** 2021-04-20

**Authors:** Shiyu Ma, Lin Zheng, Xiao Lin, Yi Feng, Ming Yang, Lan Shen

**Affiliations:** ^1^School of Pharmacy, Shanghai University of Traditional Chinese Medicine, Shanghai, China; ^2^Department of Pharmacy, Ruijin Hospital, Affiliated to Shanghai Jiaotong University School of Medicine, Shanghai, China; ^3^Department of Bone and Joint Surgery, Shanghai GuangHua Hospital of Integrated Traditional Chinese and Western Medicine, Shanghai, China; ^4^Engineering Research Center of Modern Preparation Technology of Traditional Chinese Medicine of the Ministry of Education, Shanghai University of Traditional Chinese Medicine, Shanghai, China; ^5^Department of Good Clinical Practice, Longhua Hospital, Affiliated to Shanghai University of Traditional Chinese Medicine, Shanghai, China

## Abstract

**Background:**

Da Chuan Xiong Fang (DCXF) is a traditional Chinese medicine (TCM) formula used to treat migraines. Previously, we uncovered partial mechanisms involved in the therapeutic actions of DCXF on migraines.

**Methods:**

In this study, we further elucidated its antimigraine mechanisms in vivo by using an integrated strategy coupling with network pharmacology and metabolomics techniques.

**Results:**

Network pharmacology identified 33 genes linked with both migraine and DCXF, most of which were 5-hydroxytryptamine receptors, dopamine, and peptide receptors. The results of GO and KEGG enrichment analysis showed that DCXF significantly regulated tyrosine metabolism, tryptophan metabolism, dopamine metabolic process, glucose transmembrane transport, lipid metabolism, and fatty acid transport. The results of metabolomics analysis found that the metabolism of tryptophan and tyrosine in the brain tissue and energy and lipid metabolism of rats tended towards normal and reached normal levels after administering DCXF. The metabolomics and network pharmacology approaches demonstrated similar antimigraine effects of DCXF on endogenous neurotransmitters and overall trends in serum and brain tissue. Using both approaches, 62 hub genes were identified from the protein-protein interaction (PPI) network of DCXF and gene-metabolite interaction network, with hub genes and different metabolites in serum and brain tissue. The hub genes of DCXF, which were mostly linked with inflammation, might affect mainly neurotransmitters in serum and brain tissue metabolisms.

**Conclusion:**

Network pharmacology and metabolomics study may help identify hub genes, metabolites, and possible pathways of disease and treatment. Additionally, two parts of the results were integrated to confirm each other. Their combination may help elucidate the relationship between hub genes and metabolites and provide the further understanding of TCM mechanisms.

## 1. Background

Migraine is a recurrent, common debilitating condition with neurovascular pathophysiology. The 2016 Global Burden of Disease study found that it affects an estimated 1.04 billion people (18.9% of women and 9.8% of men), with the highest prevalence in Europe and the lowest in Africa [1]. It is the second most disabling condition worldwide and a highly burdensome condition for individuals, families, and society [2]. It results in a high cost to healthcare insurance systems and patients due to the lost productive time. In mild migraine attacks, medications such as nonsteroidal anti-inflammatory drugs (NSAIDs), for example, acetaminophen and aspirin are used, whereas dihydroergotamine (DHE) or triptans are used for the treatment of moderate-to-severe migraine. Usually, migraine is associated with cardiovascular disease, psychiatric disease, and sleep disorders. However, triptans should not be prescribed to patients with a history or risk factors of coronary artery disease [3]. Medication overuse headache (MOH) occurs in predisposed subjects affected by migraine or tension-type headache. In this condition, overuse of drugs such as triptans, for example, for more than 10 days per month over three months, may transform the headache from an episode to a chronic state. MOH has limited the medication of migraine to a certain degree [3, 4].

Da Chuan Xiong Fang (DCXF) was derived from the “Xuanming Lun Fang” (an ancient Chinese medical book written in A. D. 1172), which contained two herbs, Chuan Xiong/LC (*Ligusticum striatum DC*.) and Tian Ma/GE (*Gastrodia elata* Blume). It has been extensively applied in treating migraines for thousands of years. We have previously demonstrated that DCXF may decrease blood–brain barrier (BBB) permeability and maintain its integrity through regulating serotonin, excitatory amino acids (EAAs), and matrix metalloprotein-9 (MMP-9) in rats with migraine [5–7]. Meanwhile, DCXF has also played roles in treating migraines through upregulation of 5-HT levels and downregulation of L-Glu levels and calcitonin gene-related peptide (CGRP) expressions in the hypothalamus and periaqueductal gray (PAG), reducing CGRP synthesis and inhibiting neurogenic inflammation [8]. These findings suggest that DCXF may treat migraine by regulating endogenous neurotransmitters. Recent studies showed that DCXF may improve the metabolic profile of serum and trigeminocervical complex (TCC) in rats with migraine through effects on glutamate, aspartic acid, and alanine metabolic processes and also showed the time extent of treatment effects. The possible metabolism pathway of DCXF in urine was mainly kynurenine–tryptophan (Trp-KYN) metabolism, and kynurenine was the product of the tryptophan metabolism pathway [9, 10].

Network pharmacology, as one of the new technologies to comprehensively and systematically demonstrate the therapeutic mechanisms of TCM, has been successfully and widely applied in many related research fields [11, 12]. This approach largely reflects the holistic and systematic characteristics of TCM and has clear advantages over the conventional methods in the understanding of illustration of comprehensive mechanisms [11–14]. Metabolomics is an ideal tool for the evaluation the effects of exogenous substances (drugs) on humans by tracking the products of metabolic pathways. In recent years, it has played a critical role in illustrating the possible mechanisms of TCM treatment on diseases such as diabetes and hyperthyroidism [15]. It is a strategy to find active ingredients and validate the effectiveness and mechanism of network pharmacology, confirm the effective active products of the metabolic process, and investigate the prototype compounds [16–20].

In this study, network pharmacology and metabolomics were both used to investigate the neurotransmission and metabolic mechanisms of DCXF for the treatment of migraine. Network pharmacology was applied to discover the possible therapeutic targets from multiple ingredients in LC and GE and illustrate the possible pathways in antimigraine mechanisms. Metabolomics was used to identify the possible metabolic mechanism by which DCXF has an effect on migraines.

## 2. Methods

### 2.1. Chemicals and Reagents

The two crude drugs *Ligusticum striatum DC.* (LC) and *Gastrodia elata* Blume (GE) were purchased from Shanghai Kangqiao Chinese Herbal Medicine Co., Ltd. (Shanghai, China) and were identified by Professor Yajun CUI (Department of Chinese Pharmacy, Shanghai University of Traditional Chinese Medicine) under the guidance of Chinese Pharmacopeia 2020 edition. Voucher specimens (ID: 010–5 tianma-01 and 010-chuanxiong-01) were deposited in the Museum of Shanghai University of Traditional Chinese Medicine, Shanghai University of Traditional Chinese Medicine, China. Active components of both LC and GE were identified in the preliminary studies [16–18]. Pellets were formed from the active components of LC and GE using extrusion-spheronization technology.

DCXF was formulated as follows: component A was formed from LC pellets of 33% drug loading for active components (including 9.52% ferulic acid and 18.31% senkyunolide I); component B was formed from GE pellets of 33% drug loading for active components (including 6.13% gastrodin). Components A and B (0.95 g and 2.15 g, resp.; dose ratio of 4 : 9) were then dissolved in 10 ml 0.9% normal saline (NS). The preparation, quality control, and high-performance liquid chromatography (HPLC) of DCXF, GE, and LC are shown in Supplementary S1.

Methanol, acetonitrile, and chloroform (chromatographic grade) were obtained from Merck Chemicals (Germany). Pyridine, L-thyroxine sodium, methoxyamine, and N, O-bis (trimethylsilyl) trifluoroacetamide (BSTFA) containing 1% trimethylchlorosilane (TMCS) used in the study were all purchased from Sigma-Aldrich (St. Louis, MO, USA). Nitroglycerin injection was purchased from Beijing Yimin Pharmaceutical Co., Ltd. (China). L-2-Chlorophenylalanine at 0.3 mg/mL in water was prepared and used as the internal standard. Ultrapure water was prepared with a Milli-Q system (Millipore, Billerica, MA, USA). The assay kits for serotonin (5-HT), 5-hydroxy indoleacetic acid (5-HIAA), nitrogen monoxide (NO), nitric oxide synthase (NOs), calcitonin gene-related peptide (CGRP), and dopamine (DA) were all purchased from the Beijing North Biotechnology Institute (Beijing, China).

The standards of ferulic acid, 4-hydroxy benzyl alcohol, and gastrodin were purchased from the National Institute for Food and Drug Control (Beijing, China) with a minimum purity of 98%. The standards of senkyunolide I, senkyunolide H, butylphthalide, butylidenephthalide, and vanillin were separated from LC with a minimum purity of at least 98%. Parishin was purchased from TAUTO Biotech Co., Ltd. (Shanghai, China) with a minimum purity of 95%. Phosphate was obtained commercially from China Sinopharm Co., Ltd., part of the Shanghai Chemical Reagent Company (Shanghai, China). Milli-Q Pure water system (for HPLC and ultraperformance liquid chromatography (UPLC)) was purchased from the Millipore Company (France). Acetonitrile and methanol, both HPLC grade, were purchased from Merck (Germany).

### 2.2. Network Pharmacology Analysis

#### 2.2.1. Ingredients Preparation from DCXF and Target Prediction [21]

Information about the chemical ingredients of LC and GE in the DCXF was gathered from the following literature and data sources: Herb Ingredients' Targets (HIT database); STITCH database; Chinese Academy of Sciences Chemistry database; Traditional Chinese Medicine Systems Pharmacology (TCMSP) database; Traditional Chinese Medicine Integrated Database (TCMID). We collected 49 and 12 chemical ingredients from LC and GE, respectively (Supplementary S2), and their structures were retrieved from the PubChem database (https://pubchem.ncbi.nlm.nih.gov/). The quantitative estimate of drug-likeness (QED) [11, 12] was calculated to prescreen pharmaceutically active compounds in DCXF. Those compounds with QED >0.3 were chosen for further target prediction analysis. After filtering redundant information, we obtained 46 ingredients, including 44 and two compounds from LC and GE, respectively. All chemical data were then used as a data source for target prediction (Supplementary S3).

Potential targets of these ingredients were analyzed using the score for a specific target (GS) in accordance with relevant literature [21]. A target interacting with many compounds may be regarded as a core target in the pharmacological effects of the formula. We selected the targets with GS > 0, resulting in 531 core targets (Supplementary S4).

#### 2.2.2. Molecular Docking [22]

PSOVina (http://cbbio.cis.umac.mo) is a hybrid model that combines particle swarm optimization (PSO) global search and Broyden-Fletcher-Goldfarb-Shannon (BFGS) local search methods in AutoDock Vina to tackle the conformational search problem in docking. It also has the advantage of execution time reduction (by 51–60%) [22], without compromising the prediction accuracies in the docking and virtual screening experiments. In this docking assay, six human receptors were retrieved from Protein Data Bank (http://www.wwpdb.org/), including human NO (PDB ID : 4N8T), human calcitonin gene-related peptide (PDB ID : 3AQF), human dopamine D_3_ receptor (PDB ID : 3PBL), human NOs (PDB ID : 1NSI), human 5-HT1B (PDB ID : 4IAR), and human 5-HT2AR (PDB ID : 6A93). To evaluate the accuracy of the ligand docked pose, the predicted position of each ligand atom was compared with its standard ligand using the standard root-mean-square deviation (RMSD).

#### 2.2.3. Enrichment Analysis and Network Construction

To investigate potential pathways regulated by DCXF, enrichment analysis was carried out to identify the significant biological proﬁle of DCXF. The hypergeometric *P* value has been widely used to investigate genes from predefined functional terms. In this work, gene ontology (GO) enrichment analysis for molecular function (MF), cellular component (CC), and biological process (BP) was performed, and pathway enrichment analysis was based on the Kyoto Encyclopedia of Genes and Genomes (KEGG) pathway database [21].

Analysis of the functional interactions between proteins may provide insights into the mechanisms of DCXF, and in this study, the protein–protein interaction (PPI) network was predicted using the Search Tool for the Retrieval of Interacting Genes (STRING; http://string-db.org) (version 11.0) online database. A combined score >0.4 was considered statistically significant. The plug-in application Molecular Complex Detection (MCODE) (version 1.4.2) of Cytoscape (version 3.7.0, https://cytoscape.org/) was an application for clustering a given network based on its topology to find densely connected regions [21]. The PPI networks were drawn using Cytoscape, and the significant modules in the PPI networks were identified using MCODE. The criteria for selection were as follows: MCODE scores >5, degree cut-off = 2, node score cut-off = 0.2, max. depth = 100, and k-score = 2. Subsequently, the KEGG and GO enrichment analyses were performed for genes in these modules. Hub genes were those with degrees ≥10.

Some of the aforementioned analysis was conducted using the Traditional Chinese Medicine Network Pharmacology Analysis System (TCMNPAS). The National Computer Software registration number was 2019SR1127090 (produced by author MY).

### 2.3. Disease-Associated Genes

In this work, migraine was considered a disease, and 72 associated genes were collected from the GeneCards database [23] (Supplementary S5).

### 2.4. Animals and Treatment

Twenty-four male Sprague-Dawley (SD) rats (age: four weeks; body weight: 150 ± 20 g) were supplied by the Lab Animal Center of Shanghai University of Traditional Chinese Medicine (SCXK 2013-0016). For one week prior to experiments, rats were raised in a humidity and temperature-controlled specific pathogen-free room (humidity: 60%, temperature: 22 ± 3°C) in the Experimental Animal Center of Shanghai Traditional Chinese Medicine University (Shanghai, China), with a 12 h light and dark cycle and with free access to food and sterile water. The animal facilities and protocols were approved by the Institutional Animal Care and Use Committee, Shanghai University of Traditional Chinese Medicine, on 4th of September 2016 (no. SZY201609010). All procedures were conducted in accordance with the Guide for the Care and Use of Laboratory Animal (The National Academies Press, revised edition 2010).

After three days, the animals were divided at random into three groups with eight rats in each, control group, nitroglycerin- (NTG-) induced migraine group, and DCXF group, and subjected to the following treatment schedules:

1. control group (*n* = 8): NTG vehicle, 10 ml/kg, i.h. +saline (30 min)
2. NTG-induced migraine model group (*n* = 8): NTG, 10 mg/kg, i.h. + saline (30 min)
3. DXCF + NTG group (*n* = 8): NTG, 10 mg/kg, i.h.+DCXF (30 min)

We used the NTG model followed by [24]. NTG was administered subcutaneously at a dosage of 10 mg/Kg. In the DXCF group, physiological saline and DCXF were orally administered 30 minutes after NTG at a dosage of 10 mL/Kg (weight). All rats were anesthetized 30 minutes after drug or vehicle administration.

Animal sacrifice involved anesthesia by intraperitoneal injection of ketamine (100 mg/Kg) and xylazine (12 mg/Kg) over a period of one hour. Following this, blood was drawn from the abdominal aorta, and each blood sample was centrifuged at 3500 rpm for 15 min at 4°C. Brains were removed immediately after collecting blood, and serum and brain tissue samples were stored at −80°C.

The following parameters were assessed from the blood samples: NO, NOs, 5-HT, 5-HIAA, DA, and CGRP values. The metabolites of serum and brain tissue were assessed from blood and brain samples, respectively. More details about the animals and treatment are shown in Supplementary S6.

### 2.5. Biomedical Analysis

Analysis was conducted according to the kit instructions for NO, NOs, 5-HT, 5-HIAA, DA, and CGRP. First, the standard was diluted at five grades, with each gradient sample volume being 50 *μ*L. The sample dilution of 40 *μ*L was added to the sample holes, and then, the testing sample 10 *μ*L was added to the holes. After sealing the plate with a membrane, the plate was incubated at 37°C for 30 min, after which the membrane was removed, the liquid discarded, and the sample was dried. Each hole was filled with washing liquid, allowed to stand for 30 s, and then emptied, and this process was repeated five times, after which the sample was dried again. Enzyme reagent (50 *μ*L) was added to each sample, and again, the samples were membrane-sealed and incubated at 37°C for 30 min. After this, the washing cycles previously described were repeated and the samples were dried. The chromogenic reagent A (50 *μ*L) was added and then chromogenic reagent B (50 *μ*L) was added, gently mixed by shaking, and stored in a dark environment at 37°C for 15 min. Stop buffer (50 *μ*L) was then added to each sample, the reaction was terminated, and within 15 min of this step, spectrophotometer was set to a baseline of zero, and 450 nm wavelength sequentially measured the absorbance of each hold (OD).

Data were expressed as means ± SD. For the comparisons of different groups, one-way analysis of variance (ANOVA) with LSD-t correction for multiple comparisons was conducted using SPSS version 25.0. A *P* value below 0.05 was taken to indicate a significant difference between data means.

### 2.6. Metabolites of Serum and Brain Tissue [15, 25, 26]

#### 2.6.1. Sample Preparation of Serum

Thawed serum samples were centrifuged at 3500 rpm for 10 min at 4°C, and 100 *μ*L supernatant was extracted. Methanol (400 *μ*L) was added to the supernatant and vortex-mixed for 2 min, and then the mixture was centrifuged at 10,000 rpm for 15 min at 4°C. Supernatant (20 *μ*L) was transferred into injection vials and kept at −20°C for UPLC-TOF/MS analysis.

#### 2.6.2. Sample Preparation of Brain Tissue

Methanol-water (200 *μ*L) solution was added to 50 *μ*g brain tissue sample to extract metabolites, and then, the mixture was vigorously vortexed and centrifuged at 12 000 rpm for 15 min at 4°C. The supernatant was collected and the residues were extracted using 200 *μ*L methanol. The resulting aliquots were then mixed with water-extracted supernatants. The mixtures were dried completely and combined with 80 *μ*L methoxylamine (15 mg/mL in pyridine) at 37°C for 2 h, to allow methoxylation. Subsequently, the sample was trimethylsilylated with BSTFA (with 1% TMCS) at 70°C for 1 h. All the processed samples were immediately analyzed using gas chromatography time-of-flight mass spectrometry (GC TOF/MS).

In the GC TOF/MS analysis, we followed the methods of Zhu et al., 2014 [26], to study the metabolites of brain tissue. More details of UPLC- TOF/MS and GC TOF/MS analyses were shown in Supplementary S7

## 3. Results

### 3.1. Target Prediction and Network Analysis of the Antimigraine Mechanisms of DCXF

Migraine-related genes (72 gene symbols) and core targets of DCXF (531 gene symbols) were identified. Of these, 33 genes were associated with both migraine and DCXF (Table 1). Most of the intersecting genes were 5-hydroxytryptamine receptor (including HTR1B, HTR2A, HTR7, HTR2C, HTR2B, HTR1F, and HTR1A), dopamine receptor (including DRD2, DRD5, DRD3, and DRD4), and peptide receptor (including CALCA, TAC1, HCRT, VIP, NPY, ADCYAP1, and NPS). Overall, these results are consistent with those of our previous studies.

**Figure 1:**
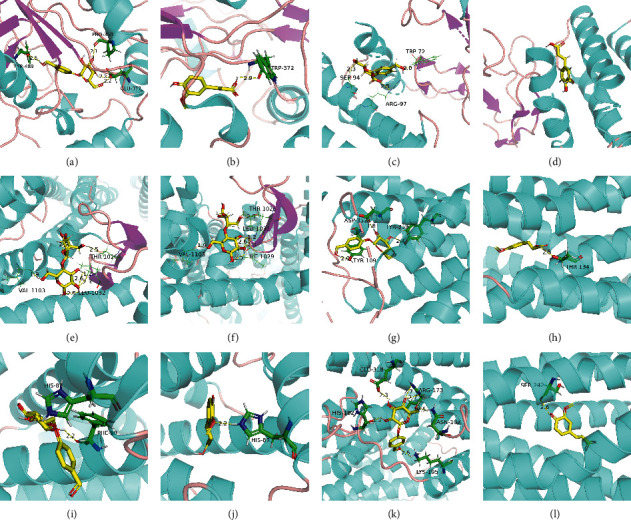
3D molecular docking model of the gastrodin and ferulic acid with six proteins. (a) Gastrodin and NOs; (b) ferulic acid and NOs; (c) gastrodin and CGRP; (d) ferulic acid and CGRP; (e) gastrodin and dopamine D3; (f) ferulic acid and dopamine D3; (g) gastrodin and 5-HT1B; (h) ferulic acid and 5-HT1B; (i) gastrodin and NO; (j) ferulic acid and NO; (k) gastrodin and 5-HT2AR; (l) ferulic acid and 5-HT2AR.

Molecular docking analysis of all active ingredients (including 46 compounds from network pharmacology analysis and seven compounds from HPLC quality analysis) showed that most of the key active ingredients, including validated gastrodin and ferulic acid, were well-docked to proteins of six human receptors, with several interactions with amino acid residues (Table 2 and Supplementary S8). A 3D molecular docking model of the gastrodin and ferulic acid with six proteins is shown in Figure 1.

**Figure 2:**
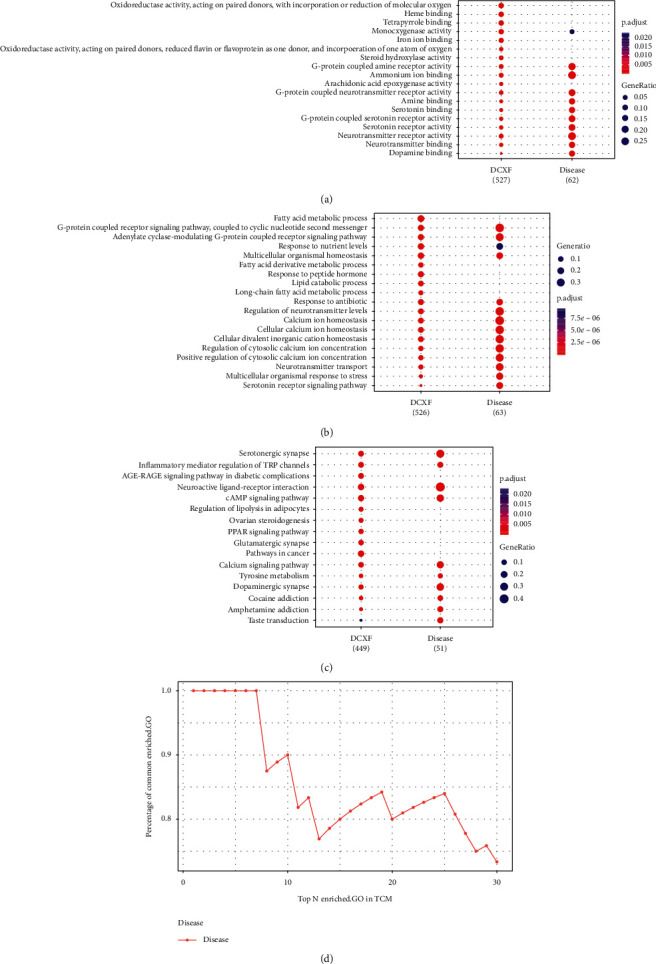
Top 10 GO terms: (a) MF, (b) BP, (c) KEGG pathways associated with DCXF and migraine, and (d) the cumulative distribution of percentages of common terms from top (30) enriched-GO. The terms are presented in descending *P* adjust value.

Further, potential pathways in the pathogenesis of migraine under DCXF treatment were screened out from candidate targets from GO and KEGG enrichment analysis.  Figure 2 showed the top 10 GO terms and KEGG pathways associated with DCXF and migraine, respectively. The number of GO (MF and BP) and KEGG terms that were significantly associated with the DCXF targets were 150, 1836, and 141 (adjusted *P* value < 0.01), respectively. For MF ontology, some of the top GO terms (GO:0030594, GO:0051378, GO:0004993, GO:0099589, GO:0099528, GO:0035240, and GO:0042165) associated with migraine were neurotransmitter receptor activity, serotonin binding, G-protein coupled serotonin receptor activity, serotonin receptor activity, G-protein coupled neurotransmitter receptor activity, dopamine binding, and neurotransmitter binding. For BP ontology, there were seven GO terms (GO:0007187, GO:0008015, GO:0003013, GO:1904659, GO:0007210, GO:0006836, and GO:0007188), which were G-protein coupled receptor signaling pathway, coupled to cyclic nucleotide second messenger, blood circulation, circulatory system process, glucose transmembrane transport, serotonin receptor signaling pathway, and neurotransmitter transport. All were significantly associated with migraine and DCXF (*P* < 0.05). From the KEGG enrichment analysis, ten KEGG pathways (hsa00350, hsa00380, hsa01522, hsa04020, hsa04024, hsa04080, hsa04540, hsa04726, hsa04728, and hsa04750), namely, tyrosine metabolism, tryptophan metabolism, endocrine resistance, calcium signaling pathway, cAMP signaling pathway, neuroactive ligand-receptor interaction, gap junction, serotonergic synapse, dopaminergic synapse, and inflammatory mediator regulation of TRP channels, were associated with migraine and DCXF.

Apart from some well-known migraine-related pathways involved in neurotransmitter receptor activity (peptide receptor activity, serotonin receptor signaling pathway, and calcium signaling pathway), the main finding was that DCXF significantly altered the regulation of the dopamine metabolic process, glucose transmembrane transport, lipid metabolism, fatty acid transport, tyrosine metabolism, and tryptophan metabolism.

Furthermore, the cumulative distribution of the percentages of common enriched-GO pathways was used as a measure for comparing the degree of association between migraine and DCXF [21]. This method was used to gauge the level of probability of association between DCXF and migraine. The significantly enriched-GO terms were combined and sorted in ascending order according to adjusted *P* values, and the percentage of common terms from the top 30 enriched terms in DCXF for migraine were presented in Figure 2. The area under the cumulative curve (AUC) was calculated using trapezoidal integration. The similarity between DCXF targets and disease genes was 0.79, and the AUC value was 17.1 for migraine. The results showed a strong association between DCXF targets and migraine genes in terms of GO.

### 3.2. PPI Network Construction and Module Analysis

**Figure 3:**
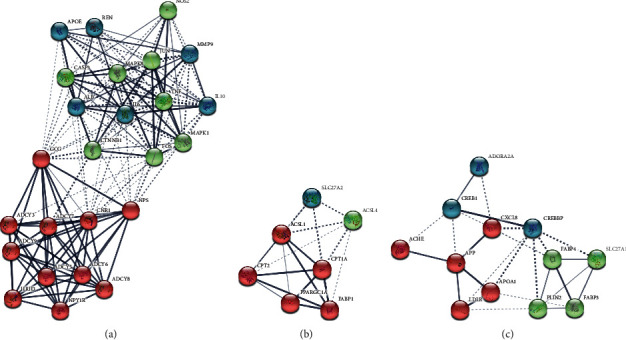
The PPI network of DCXF.

The PPI network of DCXF was constructed (number of nodes: 531; number of edges: 11115) and the three significant modules (A: scores 15.143, 29 nodes, and 212 edges; B: scores 6.33, 7 nodes and 19 edges; C: scores 4.727, 12 nodes, and 26 edges) were obtained using Cytoscape MCODE (Figure 3). A total of 62 genes were identified as hub genes with degrees ≥10 (Table 3).

The results of functional analyses showed that genes in three modules were mainly enriched and shown in Supplementary S9 (FDR < 0.05). Genes in module A were mainly enriched adenylate cyclase activity, guanylate cyclase activity, and response to forskolin. Genes in both modules B and C were enriched in long-chain fatty acid metabolism, peroxisome proliferator-activated receptor (PPAR) signaling pathway, and cholesterol metabolism.

### 3.3. Nitroglycerin (NTG) Modeling and Biomedical Analysis

The rats from the NTG model group and DCXF group displayed more impatience and anxiety (scratching, crawling, and biting) than the rats in the control group. After administration of DCXF, the rats calmed down and this spontaneous activity reduced.

**Figure 4:**
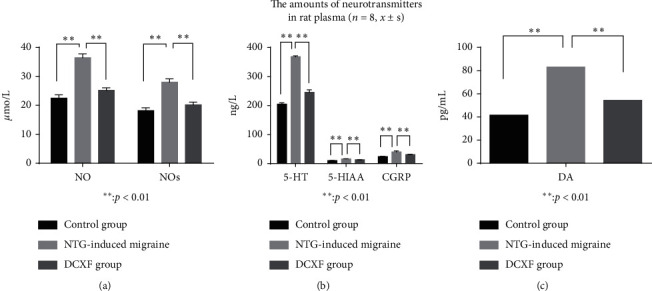
The rat plasma of 5-HT, 5-HIAA, CGRP, DA, NO, and NOs values in the control group, model group, and DCXF group (*n* = 8, *x* ± *s*).

Plasma levels of 5-HT, 5-HIAA, CGRP, DA, NO, and NOs in the model group were significantly higher than in the controls and in the DCXF group (*P* < 0.01) as shown in Figure 4. This indicates that the migraine model induced by nitroglycerin was successful and that DCXF may have therapeutic effects on the migraine model.

### 3.4. Effect of DCXF on Serum Metabolic Profiling

**Figure 5:**
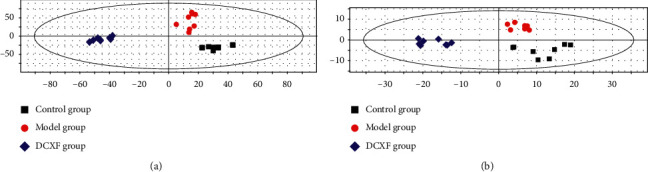
The PLS-DA clustering score map. (a) Serum at positive ion for control group (*n* = 8), NTG-induced migraine model group (*n* = 8), and DCXF group (*n* = 8); (b) brain tissue at positive ion for the three groups.

A partial least-squares discriminant analysis (PLS-DA) model (Figure 5) was constructed to visualize the influence of DCXF on the metabolic profiling of different treatment groups. This analysis showed clear separations of the control, NTG-induced migraine model, and DCXF groups, suggesting that biochemical changes differed between them (Figure 5). The DCXF profile was closer to that of the control than the model group, indicating that the metabolites had trended towards normal after DCXF injection. Differential metabolites that were accountable for intergroup variation with VIP value > 1.0 and Student's *t* test *P* value <0.05 were listed in Supplementary S10 (the significance level was set at 0.05).

After searching the HMDB and KEGG databases, the remaining metabolites were identified. Eighteen metabolites were chosen as potential biomarkers: 14 positive modes and four negative modes.

Tryptophan (Trp), tyrosine (Tyr), choline, and phosphatidylcholines (PC) were all decreased in the model group than in controls. Conversely, 5-HT, 5-hydroxytryptophan (5-HTP), 5-HIAA, DA, *γ*-aminobutyric acid (GABA), lactic acid, pyruvic acid, valine, (R)-3-hydroxybutyric acid, glutamic acid (Glu), glutamine (Gln), aspartic acid (Asp), and vanylglycol (MHPG) were all increased in the NTG-induced migraine model group compared with controls. On the whole, after DCXF treatment, these metabolites tended to decrease in comparison with the NTG-induced migraine group. Of note, MHPG, GABA, DA, 5-HT, 5-HTP, 5-HIAA, (R)-3-hydroxybutyric acid, lactic acid, and pyruvic acid returned to normal levels after DCXF administration.

### 3.5. Effects of DCXF on Brain Tissue Metabolic Profiling

The PLS-DA model (Figure 5) was constructed to visualize the influence of DCXF on the metabolic profiling of NTG-induced migraine model group rats and identify potential metabolites.

Compared with the control group, arachidonic acid (AA), valine, proline, serine, isoleucine, leucine, Asp, asparagine, Glu, and *α*-alanine increased in the NTG-induced migraine group, while Trp, Tyr, inosine, ribonic acid, and D-ribofuranose decreased in NTG-induced migraine model group.

On the whole, after DCXF treatment, proline, asparagine, Glu, Asp, serine, *α*-alanine, and valine tended closer to normal levels but they did not all reach normal levels. Fifteen metabolites were chosen as potential biomarkers and were listed in Supplementary S10.

### 3.6. Possible Pathways

To explore the possible metabolic pathways involved in the therapeutic mechanism, the metabolites that contributed to the change of the metabolic state resulting from DCXF were imported to the MetaboAnalyst 4.0 (https://www.metaboanalyst.ca/home.xhtml) (shown in Table 4). MetaboAnalyst is a comprehensive and widely used platform (>300,000 users) dedicated to metabolomics data analysis via a user-friendly and web-based interface [27, 28]. Amino acid metabolism is a common pathway for DCXF in serum and brain tissue. In addition, alanine, aspartate, and glutamate metabolism may be the metabolic pathways of DCXF in both serum and brain tissue. Differences in potential pathways obtained from serum and brain tissue might lead to different mechanisms, which could provide crucial information for understanding the mechanisms associated with DCXF.

### 3.7. Gene-Metabolite Interaction Network

The Network Explorer module in MetaboAnalyst is an easy-to-use tool that permits the mapping of metabolites and/or genes (including KEGG orthologs or KOs) onto different types of molecular interaction networks, as well as molecule-phenotype association networks [27–29]. The gene-metabolite interaction network was enabled for the exploration and visualization of interactions between functionally related metabolites and genes. The human gene associations were extracted from STITCH database, such that only interactions identified with high confidence were used.

**Figure 6:**
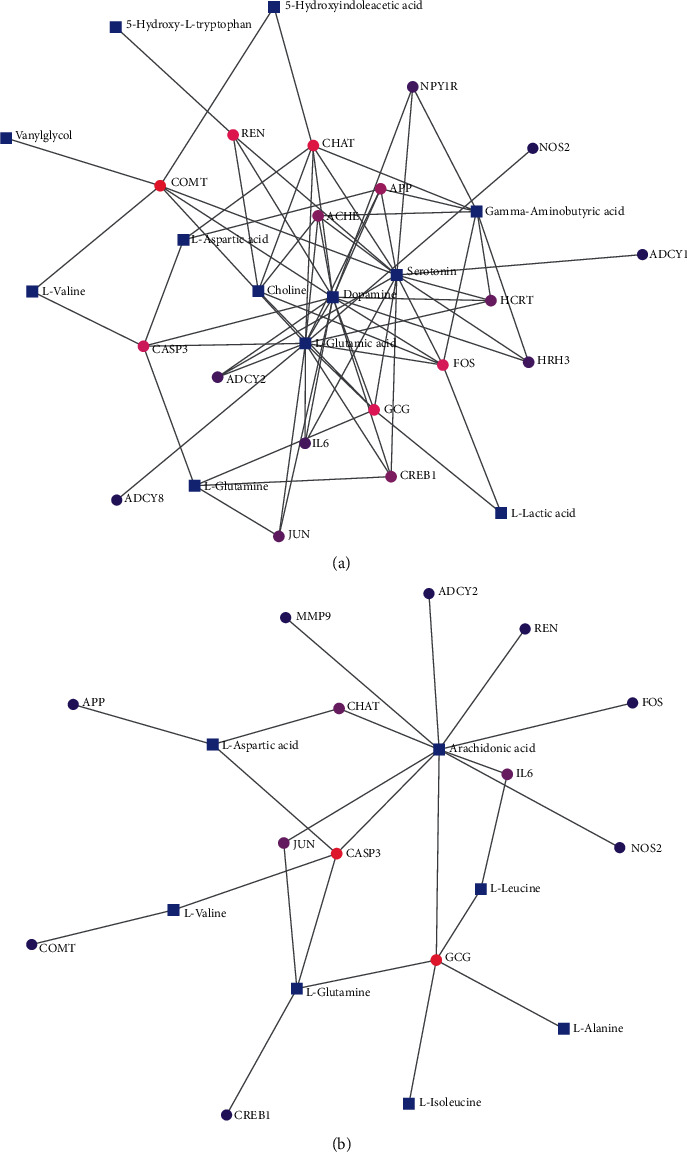
Gene-metabolite interaction network with hub genes and serum metabolites (a) and brain tissue (b) metabolites.

To explore the possible gene-metabolite interaction network related to the impact of DCXF, the hub genes and metabolites that contributed to the change of the metabolic state resulting from DCXF were imported to the MetaboAnalyst 4.0. The results are shown in Figure 6. Tables are shown in Supplementary S11.

The results of the gene-metabolite interaction network in serum metabonomics (Figure 6) showed that hub genes were related to DA, Glu, 5-HT, GABA, choline, Gln, Asp, L-Valine, 5-HIAA, L-lactic acid, Try, and vanylglycol. The most relevant metabolites were DA, Glu, and 5-HT with a degree of 15, 14, and 14, respectively, and betweenness of 100.57, 106.82, and 94.71, respectively.

CHAT, COMT, GCG, FOS, and CASP3 were the most relevant genes with metabolites at degrees of 7, 6, 6, 6, and 5, respectively. ADCY2, APP, FOS, IL6, CREB1, ACHE, GCG, CHAT, COMT, and HCRT were linked with DA, Glu, and 5-HT.

Figure 6 showed the most relevant metabolites to be AA, Gln, and Asp with degrees of 10, 4, and 3, respectively, and betweenness of 115.1, 29.9, and 20, respectively. CASP3 was linked with AA, Gln, and Asp. GCG, CASP3, CHAT, IL6, and JUN also contributed to metabolites associated with brain tissue metabonomics. The GTEx RNA-seq data were used to verify the expression of hub genes in the brain tissues (Supplementary S12). ADCY2, APP, FOS, IL6, CREB1, ACHE, CASP3, and COMT showed the highest expression among the subregions representing the brain tissue.

The most relevant metabolites were Glu and Asp both in serum and brain tissue, and these results coincided with those of the possible metabolic pathway of DCXF (alanine, aspartate, and glutamate metabolism). In addition, the hub genes of DCXF may affect mainly neurotransmitters in serum metabonomics, while in brain tissue metabonomics, the hub genes of DCXF may mostly be linked with inflammation, especially with AA.

## 4. Discussion

In this study, the antimigraine mechanism of DCXF was elucidated using network pharmacology combined with metabolomics techniques. Network pharmacology analysis demonstrated that multiple components of DCXF exerted a therapeutic effect on migraine by modulating multiple targets and pathways. Of these components, the main compounds of DCXF (e.g., SEI, gastrodin, and ferulic acid [7, 8]) were shown to have the potential for migraine treatment, which confirmed the therapeutic value of DCXF against this disease. We found 532 genes as potential targets of DCXF by network-based target prediction. Furthermore, 33 genes associated with both migraine and DCXF were investigated, and most of the intersecting genes were 5-hydroxytryptamine receptors, dopamine receptors, and peptide receptors.

DCXF has been found to affect the expression of the 5-HT1D receptor, upregulate the expression of monoamine neurotransmitter 5-HT1B receptor, and downregulate the expression of C-JUN [30, 31]. It may also significantly inhibit the expansion of dural blood vessels caused by NTG modeling and the activation of NOS, SP, and CGRP receptors caused by NO [32]. In addition, DCXF induced a downward trend in expression of the dopamine D2 receptor in the midbrain trigeminal nucleus [33].

In our previous study, we focused on the action of DCXF on the peptide receptor. The mechanism of DCXF in migraine management may be associated with its capability in downregulating CGRP expressions in the hypothalamus and PAG, reducing CGRP synthesis, and inhibiting neurogenic inflammation [7]. Combining previous research with the present findings, we hypothesize that various compounds in DCXF may act on migraine through different biological pathways, which may be highly dependent on the 5-hydroxytryptamine receptors, dopamine receptors, and peptide receptors.

Studies have suggested possible endogenous metabolite biomarkers in the development and progression of migraine, including glutamate/glutamine [10], aspartate [10], GABA [34], PC [35], choline [35], lactate [36], and fatty acids [37]. Amino acid metabolism is vital to the understanding of migraine. Amino acids not only consist of units of protein but also play roles in signal transduction and participate in metabolic pathways as neurotransmitters. In addition to affecting amino acid metabolism, prevalent triggers of migraine attacks could all be linked to unbalanced cerebral energy metabolism, especially lactic acidosis (a nonspecific hallmark of mitochondrial disorders). Recent findings have suggested an association between migraine and changes in high-density lipoprotein subspecies (HDL) metabolism rather than general dyslipidaemia [37]. This may suggest that the migraine is linked with lipid metabolism. In our study, the results of GO and KEGG enrichment analysis showed that DCXF significantly regulated the dopamine metabolic process, glucose transmembrane transport, lipid metabolism, fatty acid transport, tyrosine metabolism, and tryptophan metabolism.

**Figure 7:**
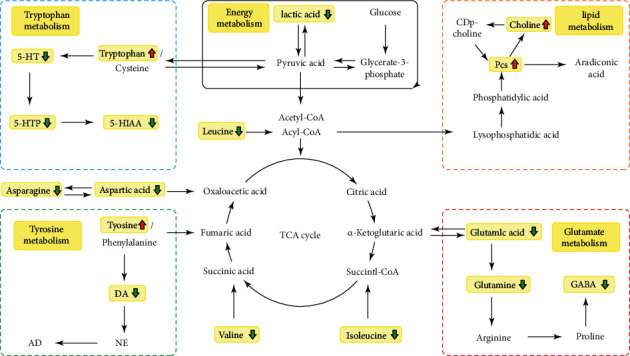
The possible metabolic pathways of DCXF in case of migraine.

From the metabolomics study (Figure 7), the therapeutic effects of DCXF and its possible mechanisms in vivo verified the same antimigraine effect on the changes of endogenous neurotransmitters (5-HT, 5-HIAA, CGRP, DA, NO, and NOs) and similar metabolic (glutamate and aspartate, tyrosine, tryptophan, energy, and lipid metabolism) trends in serum and brain tissue. After NTG modeling, 5-HT and 5-HIAA increased significantly in serum.

Tryptophan is metabolized in the brain by tryptophan hydroxylase 2 (TPH2) to produce 5-HTP, and 5-HTP generates 5-HT via serotonin decarboxylase. 5-HT generates 5-HIAA under the action of monoamine oxidase (Mao) [38–41]. Therefore, 5-HT led tryptophan to be increased in the brain and decreased in the blood. DCXF regulated tryptophan metabolism, by increasing it in serum and reducing 5-HT, 5-HIAA, and 5-HTP to further relieve the increased neurotransmitter after NTG modeling. Excitatory amino acids, such as Glu, Gln, and asp, increased significantly in the blood in migraine attacks, while brain norepinephrine levels dropped, causing a dopamine waterfall effect in the sympathetic nerve endings [41]. This forces tyrosine levels to increase in the brain and decrease in the blood, and then, DA, MHPG, and norepinephrine are synthesized in the brain, thus leading to increased blood content. The changes in these transmitters affect the central nervous system of migraine patients, leading to mitochondrial dysfunction, increased glutamate levels, and release of CGRP, which contribute to sensitization and inflammation of nerves [42].

DCXF may increase tyrosine and decrease DA. In our previous study, we found that DCXF could improve the metabolic profile of serum and TCC in migraine rats and showed the process of treatment over time, involving mainly amino acid (glutamate and aspartic acid) metabolism. In the present study, in terms of brain tissue metabolomics, Asp and Glu were markedly increased in the NTG-induced migraine model group compared with the control group. DCXF had the same effect on glutamate and aspartic acid metabolism, by decreasing asparagine (synthesized from aspartic acid and ATP), aspartic acid, glutamine acid, glutamine, and GABA (transformed from glutamate catalyzes decarboxylation by glutamate decarboxylase). We also found that DCXF may affect lactic acid, choline, and PCs by regulating energy and lipid metabolism. This was embodied in the increased serum contents of choline and phosphatidylcholine along with decreased lactic acid content in rats, indicating the anaerobic glycolysis of glucose. Anaerobic glycolysis of pyruvic acid would produce pyruvic acid, valine, and leucine and would increase their levels in blood and brain. Most amino acids can be transformed into each other or other types of substances (such as lipids or carbohydrates) through the tricarboxylic acid cycle. The metabolomics approach in the present study found no metabolites closely linked with the TCA cycle. One explanation might be that our study mainly focused on the metabolic trend after 30 minutes of DCXF administration. In our previous study, we found that DCXF regulated succinic acid and citric acid after 90 minutes of administration. Taken together, these findings may suggest that DCXF firstly regulates amino acid metabolism (30 minutes) and later affects energy and lipid metabolism (90 minutes).

Using the aforementioned two technologies in combination, the gene-metabolite interaction networks with hub genes and metabolites (serum and brain tissue) were established. Overall, in serum and brain tissue, the most relevant metabolites were Gln and AA, which were also key to the possible metabolic pathway of DCXF (alanine, aspartate, and glutamate metabolism). Using serum metabolomics, we also found that the hub genes of DCXF may affect mainly neurotransmitters and may mostly be linked with inflammation in brain tissue metabolomics, particularly with AA, which was notably elevated in brain tissue of the model group. This might be attributed to peroxidation of the cell membrane by reactive oxygen species production, thus contributing to the release of the cell phospholipase. The increased serine and alanine in the brain also suggest halted folic acid anabolism or Gln synthesis.

**Figure 8:**
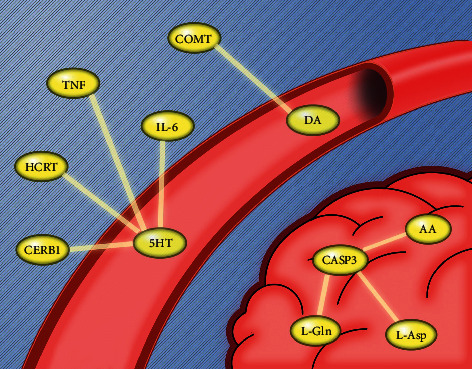
Some of the hub genes linked with metabolites.

We found that arachidonic acid metabolism was closely related to 5-HT. Previous research [43] has found that arachidonic acid and its metabolites serve as messengers within or between cells. They may oxidize the coupling of 5-HT2A (5-HT2B receptor), activate the 5-HT2A/PLA2 signal pathway, and have an effect on migraine. With the establishment of a gene-metabolite interaction network, we found some significant hub genes of DCXF that were associated with metabolites (Figure 8). CASP3 was linked with AA, Gln, and Asp in brain tissue metabolism. The protein encoded by CASP3 is a cysteine-aspartic acid protease that plays a central role in the execution phase of cell apoptosis. In injury, AA activates a stress response resulting in CASP3 activation, which contributes to cerebral vascular damage and dysfunction with an increase in CASP3 levels during neuroinflammation [44]. CASP3 may accelerate neuronal cell death in several cortical spreading depression-related neurological disorders, such as stroke, migraine, and epilepsy [45]. CERB1, HCRT, IL-6, and TNF have been mainly linked with 5-HT. CERB1 encodes a transcription factor, which is a member of the leucine zipper family of DNA binding proteins, and bonds as a homodimer to the cAMP-responsive element, an octameric palindrome. Upon exposure of sensory neurons to the neurotransmitter 5-HT, CREB1 is activated via the protein kinase A (PKA) intracellular signaling pathways [46].

IL-6 and TNF are primarily produced at the sites of acute and chronic inflammation, where they are secreted into the serum and induce a transcriptional inflammatory response. Some studies [45] have underlined that peripheral inflammation (injection with TNF and/or IL-6) reached the hypothalamus, where it affected serotonergic metabolism. A hypothalamic neuropeptide precursor protein encoded by HCRT has been found to give rise to two mature neuropeptides. It was proposed that HCRT may be responsible for migraines, mainly in the early phases of attack, as hypocretinergic neurons originate from the hypothalamus and control the serotonergic system [45]. Another study found that the COMT gene was mainly associated with dopamine, degrading dopamine and its precursor l-DOPA and also playing a critical role in regulating synaptic dopamine actions [46].

In the present study, MCODE and hub gene analysis of PPI in DCXF found that DCXF could act on inflammation-related genes, such as IL-10, TNF, IL-6, which were mainly enriched under adenylate cyclase activity and guanylate cyclase activity and in response to forskolin (FSK). Other studies found that active ingredients, such as gastrodin [47, 48] and ligustrazine [49], from DCXF reduced the serum levels of proinflammatory cytokines (IL-6, IL-1*β*, and TNF), increased the activity of superoxide dismutase, and reduced the concentration of malondialdehyde. In recent studies, high serum levels of IL-1*β*, IL-6, and TNF (proinflammatory cytokines) in patients with migraine indicated that migraine was associated with inflammation within peripheral endings of trigeminal ganglion sensory neurons [50]. So, CGRP, TNF-*α*, and IL-1*β* were proposed as therapeutic targets of migraine [3, 43]. It is well known that FSK, as an adenylate cyclase-specific activator, can upregulate intracellular cAMP levels and affect the BBB permeability. It could also evoke an increased NO release from trigeminal satellite glial cells, which given the putative role of NO in painful conditions such as migraine could be modulated by Glu [43]. Moreover, FSK could potentiate the TNF-*α* mediated upregulation of BDNF expression. In other studies, LC acts as a vasodilator to increase the levels of cyclic adenosine monophosphate (cAMP) and cyclic guanosine monophosphate (cGMP) in muscle and blood and may be partly mediated by the inhibition of cAMP phosphodiesterase or cGMP phosphodiesterase. Since DCXF consists of both GE and LC, its regulation of cAMP and cGMP might be different from that of LC. The results of the MCODE analysis and the hub genes might provide a new hypothesis of DCXF on antimigraine mechanisms.

## 5. Conclusions

Our results firmly supported and enhanced the current understanding of the therapeutic effects of DCXF on migraine. We have demonstrated an effective strategy combining network pharmacology and metabolomics to understand the mechanisms of action of DCXF. Our findings may facilitate the generation of new hypotheses to reveal the mechanisms of TCM treatment for migraine.

## Figures and Tables

**Table 1:** Genes associated with both migraine and DCXF.

Disease gene symbol (33 genes)
CALCA, TNF, HTR1B, TAC1, HTR2A, HTR7, DRD2, HTR2C, MAOA, HTR2B, HTR1F, TACR1, HCRT, HRH3, HTR1A, TRPV1, VIP, NPY, DRD5, SLC6A4, ADCYAP1, NPS, DRD3, DRD4, PPBP, FOS, CNR1, COMT, EDNRA, TRPA1, EDNRB, MAOB, and CYP2D6

**Table 2:** GO and KEGG terms enrichment analysis of DCXF in the most significant module. FDR: false discovery rate.

Pathway ID	Pathway description	% associated gene	FDR
GO:0004016	Adenylate cyclase activity	72.73	1.29 ^*∗*^ 10^−21^
GO:1904321	Response to forskolin	60.00	1.25 ^*∗*^ 10^−15^
GO:1904322	Cellular response to forskolin	60.00	1.25 ^*∗*^ 10^−15^
GO:0004383	Guanylate cyclase activity	42.11	5.86 ^*∗*^ 10^−19^
GO:0008074	Guanylate cyclase complex, soluble	42.11	5.86 ^*∗*^ 10^−19^
GO:0016849	Phosphorus-oxygen lyase activity	34.78	3.79 ^*∗*^ 10^−18^

**Table 3:** Hub genes of DCXF.

Hub genes (62 genes)
MAPK8, JUN, APOA1, APOB, FOS, CREB1, CREBBP, IL10, TNF, IL6, MAPK1, LDLR, APOE, CXCL8, APP, PPARA, PPARGC1A, ALB, FABP1, NPS, HCRT, NOS2, CTNNB1, CASP3, GCG, GNG2, CPT1A, FABP4, CPT2, SLC27A1, PTGS2, ACSL1, PLIN2, ADCY2, ADCY1, ADCY5, ADCY7, MMP9, ADCY8, ADCY9, ADCY6, CHAT, ACHE, COMT, ADCY3, CNR1, HRH3, NPY1R, SLC27A2, FABP3, ADORA2A, and REN

**Table 4:** Possible pathways for DCXF (FDR: false detection rate).

Serum	FDR	Impact	Brain	FDR	Impact
Alanine, aspartate, and glutamate metabolism	0.00030	0.621	Aminoacyl-tRNA biosynthesis	6.85 ^*∗*^ 10^−12^	0.167
Arginine biosynthesis	0.0097	0.117	Valine, leucine and isoleucine biosynthesis	0.00106	0
Butanoate metabolism	0.0097	0.0318	Alanine, aspartate and glutamate metabolism	0.0375	0.337
Tryptophan metabolism	0.012	0.393	Arginine biosynthesis	0.00556	0
Tyrosine metabolism	0.012	0.269	Pantothenate and CoA biosynthesis	0.0103	0
Nitrogen metabolism	0.0037	0.017	Glyoxylate and dicarboxylate metabolism	0.0280	0.0423
D-Glutamine and D-glutamate metabolism	0.0089	0.018	Phenylalanine, tyrosine and tryptophan biosynthesis	0.0332	0.5
Glyoxylate and dicarboxylate metabolism	0.016	0.042	D-Glutamine and D-glutamate metabolism	0.0494	0

## Data Availability

The datasets used for the current study are available from the corresponding author upon request

## Abbreviations

DCXF: Da Chuan Xiong Fang
TCM: Traditional Chinese medicine
NSAIDs: Nonsteroidal anti-inflammatory drugs
DHE: Dihydroergotamine
MOH: Medication overuse headache
TCC: Trigeminocervical complex
Trp-KYN: Tryptophan-kynurenine pathway
GABA: g-Aminobutyrate acid
5-HT: Serotonin
BBB: Blood–brain barrier
EAAs: Excitatory amino acids
MMP-9: Matrix metalloprotein-9
PAG: Periaqueductal gray
LC: *Ligusticum striatum DC.*
GE: *Gastrodia elata* Blume
CDMT: Codergocrine mesylate tablets
5-HIAA: 5-Hydroxyindolacetic acid
NO: Nitrogen monoxide
NOs: Nitric oxide synthase
CGRP: Calcitonin gene-related peptide
DA: Dopamine
QED: Quantitative estimate of drug-likeness
RMSD: Standard root-mean-square deviation
MF: Molecular function
CC: Cellular component
BP: Biological process
MCODE: Molecular complex detection
PLS-DA: Partial least square discriminant analysis
VIP: Variable importance in the projection
NTG: Nitroglycerin
Trp: Tryptophan
Tyr: Tyrosine
PC: Phosphatidylcholines
5-HTP: 5-Hydroxytryptophan
Glu: Glutamic acid
Gln: Glutamine
Asp: Aspartic acid
SOD: Superoxide dismutase
MDA: Malondialdehyde
cAMP: Cyclic adenosine monophosphate
cGMP: Cyclic guanosine monophosphate
FSK: Forskolin
AA: Arachidonic acid
PKA: Protein kinase A.

## Supplementary Materials

Supplementary Materials Supplementary S1: preparation, quality control, and HPLC of DCXF, GE, and LC. Supplementary S2: ingredients from LC and GE. Supplementary S3: QED results of GE and LC. Supplementary S4: 531 core targets. Supplementary S5: migraine genes. Supplementary S6: ARRIVE statement for animal experiments. Supplementary S7: metabolites of serum of brain tissue. Supplementary S8: all active ingredients molecular docking results. Supplementary S9: results of MCODE. Supplementary S10: effect of DCXF on serum and brain tissue metabolic profiling. Supplementary S11: gene-metabolite interaction network. Supplementary S12: GTEx RNA-seq data to verify the expression of hub genes in the brain tissues.
