# Supplementary material for: Network Pharmacology and Metabolomics Studies on Antimigraine Mechanisms of Da Chuan Xiong Fang (DCXF)
Source: Evid Based Complement Alternat Med. 2021 Apr 20;2021:6665137. doi: 10.1155/2021/6665137 (PMC8081595; doi:10.1155/2021/6665137)
Supplement: Supplementary Materials — Supplementary S1: preparation, quality control, and HPLC of DCXF, GE, and LC. Supplementary S2: ingredients from LC and GE. Supplementary S3: QED results of GE and LC. Supplementary S4: 531 core targets. Supplementary S5: migraine genes. Supplementary S6: ARRIVE statement for animal experiments. Supplementary S7: metabolites of serum of brain tissue. Supplementary S8: all active ingredients molecular docking results. Supplementary S9: results of MCODE. Supplementary S10: effect of DCXF on serum and brain tissue metabolic profiling. Supplementary S11: gene-metabolite interaction network. Supplementary S12: GTEx RNA-seq data to verify the expression of hub genes in the brain tissues. [file 6665137.f1.zip › 6665137.f1/Supplementary S10 Effect of DCXF on serum and brai.docx]

**Supplementary S10 Effect of DCXF on serum and brain tissue metabolic profiling**

Differential metabolites that were accountable for inter-group variation with VIP value >1.0 and Student’s t-test P-value <0.05 were listed in it (the significance level was set at 0.05).The fold changes (FC) of the potential metabolites based on peak intensity value were listed in it.

Table 1 The identification of potential biomarkers in serum

| Group | No. | *R.T*  /min | Actual Mass  /m·z^-1^ | Selected ion | Identification results | Element composition | FC  Model/Control | F C  DCXF  /Control | F C  DCXF/Model |
| --- | --- | --- | --- | --- | --- | --- | --- | --- | --- |
| Serum positive ion | 1 | 4.69 | 205.0854 | [M+H]^+^ | Tryptophan | C_11_H_12_N_2_O_2_ | 0.48(+) | 1.84(-) | 3.79(-) |
|  | 2 | 0.73 | 182.0798 | [M+H]^+^ | Tyrosine | C_9_H_11_NO_3_ | 0.52(+) | 0.64(-) | 1.24(-) |
|  | 3 | 12.02 | 213.0716 | [M+H]^+^ | Serotonin | C_10_H_12_ N_2_O | 1.52(+) | 1.42(+) | 0.94(-) |
|  | 4 | 12.03 | 223.0883 | [M+H]^+^ | 5-hydroxytryptophan | C_11_H_12_ N_2_O_3_ | 1.94(+) | 1.03(-) | 0.52(+) |
|  | 5 | 12.27 | 154.0888 | [M+H]^+^ | Dopamine | C_8_H_11_NO_2_ | 3.34(+) | 0.85(-) | 0.25(+) |
|  | 6 | 9.80 | 192.0846 | [M+H]^+^ | 5-hydroxyindoleacetic acid | C_10_H_9_NO_3_ | 1.75(+) | 1.27(-) | 0.73(-) |
|  | 7 | 5.27 | 104.1047 | [M+H]^+^ | γ-aminobutyric acid | C_4_H_9_NO_2_ | 2.69(+) | 0.77(+) | 0.29(-) |
|  | 8 | 9.57 | 90.0272 | [M+H]^+^ | Lactic acid | C_3_H_6_O_3_ | 2.35(+) | 0.91 (-) | 0.39(+) |
|  | 9 | 11.99 | 868.637 | [M+H]^+^ | PC(P-16:0/20:5(5Z,8Z,11Z,14Z,17Z)) | C_50_H_94_NO_8_P | 0.06(+) | 0.21 (-) | 3.60 (-) |
|  | 10 | 1.94 | 104.1035 | [M+H]^+^ | Choline | C_5_H_14_NO | 0.46(+) | 0.72(-) | 1.57(-) |
|  | 11 | 12.43 | 88.0153 | [M+H]^+^ | Pyruvic acid | C_3_H_4_O_3_ | 1.58(+) | 1.12 (-) | 0.71(+) |
|  | 12 | 7.92 | 764.5547 | [M+H]^+^ | PC(24:1(15Z)/18:2(9Z,12Z)) | C_44_H_78_NO_7_P | 0.20(+) | 0.94(-) | 4.71(-) |
|  | 13 | 3.98 | 118.0861 | [M+H]^+^ | Valine | C_5_H_11_NO_2_ | 2.86(+) | 1.65(-) | 0.58(-) |
|  | 14 | 1.19 | 105.0414 | [M+H]^+^ | (R)-3-Hydroxybutyric acid | C_4_H_8_O_3_ | 0.70(+) | 0.10(+) | 0.12(+) |
| Serum  Negative ion | 15 | 1.27 | 147.0790 | [M-H]^-^ | Glutamic acid | C_5_H_9_NO_4_ | 5.47(+) | 3.68(+) | 0.67 (-) |
|  | 16 | 0.597 | 132.8859 | [M-H]^-^ | Aspartic acid | C_4_H_7_NO_4_ | 4.34(+) | 6.56(+) | 1.51 (-) |
|  | 17 | 0.658 | 146.0621 | [M-H]^-^ | Glutamine | C_5_H_10_N_2_O_3_ | 1.69(+) | 1.95(+) | 1.16(+) |
|  | 18 | 0.657 | 183.0605 | [M-H]^-^ | Vanylglycol | C_9_H_12_O_4_ | 2.66(+) | 0.95(-) | 0.36(+) |

(+) significant difference between groups; (-) no significant difference between group.

Table 2 The identification of potential biomarkers in brain tissue

| No. | Identification  results | Element composition | FC  Model/Control | FC  DCXF/Control | F C  DCXF/Model |
| --- | --- | --- | --- | --- | --- |
| 1 | Tryptophan | C_11_H_12_N_2_O_2_ | 0.44(+) | 0.47 (-) | 1.06(+) |
| 2 | Tyrosine | C_9_H_11_NO_3_ | 0.77(+) | 0.38 (+) | 0.49 (+) |
| 3 | Arachidonic acid | C_20_H_32_O_2_ | 0.46 (+) | 0.46 (+) | 0.99 (+) |
| 4 | Glutamine | C_5_H_10_N_2_O_3_ | 1.17 (+) | 0.47 (+) | 0.40 (+) |
| 5 | Valine | C_5_H_11_NO_2_ | 1.36 (+) | 0.67(-) | 0.49 (+) |
| 6 | Proline | C_5_H_9_NO_2_ | 1.16 (+) | 0.61 (+) | 0.53 (+) |
| 7 | Serine | C_3_H_7_NO_3_ | 1.12 (+) | 0.44 (+) | 0.40 (-) |
| 8 | Isoleucine | C_6_H_13_NO_2_ | 1.25 (+) | 0.57 (+) | 0.46(+) |
| 9 | Leucine | C_6_H_13_NO_2_ | 1.16 (+) | 0.71 (-) | 0.61 (+) |
| 10 | Asparagine | C_4_H_8_N_2_O_3_ | 1.64 (+) | 0.65(-) | 0.39(+) |
| 11 | Aspartic acid | C_4_H_7_NO_4_ | 1.14 (+) | 0.80 (-) | 0.70 (-) |
| 12 | α-alanine | C_3_H_7_NO_2_ | 0.48 (+) | 0.86 (-) | 1.79 (+) |
| 13 | Inosine | C_10_H_12_N_4_O_5_ | 0.14 (+) | 0.03 (+) | 0.22 (+) |
| 14 | Ribonic acid | C_5_H_10_O_6_ | 0.77 (+) | 0.72 (+) | 0.93(-) |
| 15 | D-Ribofuranose | C_5_H_10_O_5_ | 0.80 (+) | 0.50 (+) | 0.62 (+) |

(+) significant difference between groups; (-) no significant difference between group.
