# Supplementary material for: Network Pharmacology and Metabolomics Studies on Antimigraine Mechanisms of Da Chuan Xiong Fang (DCXF)
Source: Evid Based Complement Alternat Med. 2021 Apr 20;2021:6665137. doi: 10.1155/2021/6665137 (PMC8081595; doi:10.1155/2021/6665137)
Supplement: Supplementary Materials — Supplementary S1: preparation, quality control, and HPLC of DCXF, GE, and LC. Supplementary S2: ingredients from LC and GE. Supplementary S3: QED results of GE and LC. Supplementary S4: 531 core targets. Supplementary S5: migraine genes. Supplementary S6: ARRIVE statement for animal experiments. Supplementary S7: metabolites of serum of brain tissue. Supplementary S8: all active ingredients molecular docking results. Supplementary S9: results of MCODE. Supplementary S10: effect of DCXF on serum and brain tissue metabolic profiling. Supplementary S11: gene-metabolite interaction network. Supplementary S12: GTEx RNA-seq data to verify the expression of hub genes in the brain tissues. [file 6665137.f1.zip › 6665137.f1/Supplementary S11 Gene-Metabolite Interaction Network.docx]

**Supplementary S11 Gene-Metabolite Interaction Network**

Important nodes can be identified based on their position within the network. The assumption is that changes in the key positions of a network will have more impact on the network than changes on marginal or relatively isolated positions. MetaboAnalyst provides two well-established node centrality measures to estimate node importance - degree centrality and betweenness centrality. In a graph network, the degree of a node is the number of connections it has to other nodes. Nodes with higher node degree act as hubs in a network. The betweenness centrality measures the number of shortest paths going through the node. It takes into consideration the global network structure. For example, nodes that occur between two dense clusters will have a high betweenness centrality even if their degree centrality values are not high. Note, you can sort the node table based on either degree or betweenness values by double clicking the corresponding column header.

Table 1(1) Gene-Metabolite Interaction Network associated with hub genes and seurm metabolites

| Id | Label | Degree | Betweenness |
| --- | --- | --- | --- |
| C03758 | Dopamine | 15 | 100.57 |
| C00025 | L-Glutamic acid | 14 | 106.82 |
| C00780 | Serotonin | 14 | 94.71 |
| 1103 | CHAT | 7 | 35.53 |
| C00334 | Gamma-Aminobutyric acid | 7 | 11.01 |
| 1312 | COMT | 6 | 55.09 |
| 2641 | GCG | 6 | 27.8 |
| 2353 | FOS | 6 | 23.57 |
| 836 | CASP3 | 5 | 21.87 |
| 351 | APP | 5 | 13.48 |
| 43 | ACHE | 5 | 9.82 |
| C00114 | Choline | 5 | 8.75 |
| 5972 | REN | 4 | 30.43 |
| 1385 | CREB1 | 4 | 8.63 |
| 3060 | HCRT | 4 | 5.21 |
| C00064 | L-Glutamine | 4 | 4.08 |
| 3725 | JUN | 3 | 4.33 |
| C00049 | L-Aspartic acid | 3 | 2.24 |
| 108 | ADCY2 | 3 | 2.21 |
| 3569 | IL6 | 3 | 2.21 |
| 4886 | NPY1R | 3 | 1.9 |
| 11255 | HRH3 | 3 | 1.9 |
| C00183 | L-Valine | 2 | 1.33 |
| C05635 | 5-Hydroxyindoleacetic acid | 2 | 1.14 |
| C00186 | L-Lactic acid | 2 | 0.34 |
| 114 | ADCY8 | 1 | 0 |
| 4843 | NOS2 | 1 | 0 |
| 107 | ADCY1 | 1 | 0 |
| C01017 | 5-Hydroxy-L-tryptophan | 1 | 0 |
| C05594 | Vanylglycol | 1 | 0 |

Table 1(2) Gene-Metabolite Interaction Network associated with hub genes and seurm metabolites

| hub genes | associated with  seurm metabolites |
| --- | --- |
| APP | L-Aspartic acid |
| CASP3 | L-Aspartic acid |
| CHAT | L-Aspartic acid |
| REN | 5-Hydroxy-L-tryptophan |
| ADCY2 | L-Glutamic acid |
| JUN | L-Glutamic acid |
| APP | L-Glutamic acid |
| FOS | L-Glutamic acid |
| IL6 | L-Glutamic acid |
| CASP3 | L-Glutamic acid |
| CREB1 | L-Glutamic acid |
| ACHE | L-Glutamic acid |
| GCG | L-Glutamic acid |
| ADCY8 | L-Glutamic acid |
| NOS2 | L-Glutamic acid |
| CHAT | L-Glutamic acid |
| COMT | L-Glutamic acid |
| HCRT | L-Glutamic acid |
| ADCY2 | Serotonin |
| REN | Serotonin |
| APP | Serotonin |
| FOS | Serotonin |
| IL6 | Serotonin |
| CREB1 | Serotonin |
| ADCY1 | Serotonin |
| ACHE | Serotonin |
| GCG | Serotonin |
| NPY1R | Serotonin |
| CHAT | Serotonin |
| HRH3 | Serotonin |
| COMT | Serotonin |
| HCRT | Serotonin |
| ADCY2 | Dopamine |
| JUN | Dopamine |
| REN | Dopamine |
| APP | Dopamine |
| FOS | Dopamine |
| IL6 | Dopamine |
| CASP3 | Dopamine |
| CREB1 | Dopamine |
| ACHE | Dopamine |
| GCG | Dopamine |
| NPY1R | Dopamine |
| CHAT | Dopamine |
| HRH3 | Dopamine |
| COMT | Dopamine |
| HCRT | Dopamine |
| JUN | L-Glutamine |
| CASP3 | L-Glutamine |
| CREB1 | L-Glutamine |
| GCG | L-Glutamine |
| REN | Choline |
| FOS | Choline |
| ACHE | Choline |
| GCG | Choline |
| CHAT | Choline |
| APP | Gamma-Aminobutyric acid |
| FOS | Gamma-Aminobutyric acid |
| ACHE | Gamma-Aminobutyric acid |
| NPY1R | Gamma-Aminobutyric acid |
| CHAT | Gamma-Aminobutyric acid |
| HRH3 | Gamma-Aminobutyric acid |
| HCRT | Gamma-Aminobutyric acid |
| FOS | L-Lactic acid |
| GCG | L-Lactic acid |
| CHAT | 5-Hydroxyindoleacetic acid |
| COMT | 5-Hydroxyindoleacetic acid |
| CASP3 | L-Valine |
| COMT | L-Valine |
| COMT | Vanylglycol |

Table2(1) Gene-Metabolite Interaction Network associated with hub genes and brain tissue metabolites

| Id | Label | Degree | Betweenness |
| --- | --- | --- | --- |
| C00219 | Arachidonic acid | 10 | 115.1 |
| 2641 | GCG | 5 | 49.87 |
| 836 | CASP3 | 4 | 57.03 |
| C00064 | L-Glutamine | 4 | 29.9 |
| C00049 | L-Aspartic acid | 3 | 20 |
| C00183 | L-Valine | 2 | 18 |
| 1103 | CHAT | 2 | 10.47 |
| 3569 | IL6 | 2 | 5.63 |
| 3725 | JUN | 2 | 5 |
| C00123 | L-Leucine | 2 | 2 |
| 351 | APP | 1 | 0 |
| 1385 | CREB1 | 1 | 0 |
| 108 | ADCY2 | 1 | 0 |
| 5972 | REN | 1 | 0 |
| 2353 | FOS | 1 | 0 |
| 4318 | MMP9 | 1 | 0 |
| 4843 | NOS2 | 1 | 0 |
| 1312 | COMT | 1 | 0 |
| C00041 | L-Alanine | 1 | 0 |
| C00407 | L-Isoleucine | 1 | 0 |

Table2(2) Gene-Metabolite Interaction Network associated with hub genes and brain tissue metabolites

| hub genes | associated with  brain tissue metabolites |
| --- | --- |
| APP | L-Aspartic acid |
| CASP3 | L-Aspartic acid |
| CHAT | L-Aspartic acid |
| JUN | L-Glutamine |
| CASP3 | L-Glutamine |
| CREB1 | L-Glutamine |
| GCG | L-Glutamine |
| ADCY2 | Arachidonic acid |
| JUN | Arachidonic acid |
| REN | Arachidonic acid |
| FOS | Arachidonic acid |
| MMP9 | Arachidonic acid |
| IL6 | Arachidonic acid |
| CASP3 | Arachidonic acid |
| GCG | Arachidonic acid |
| NOS2 | Arachidonic acid |
| CHAT | Arachidonic acid |
| GCG | L-Alanine |
| GCG | L-Isoleucine |
| IL6 | L-Leucine |
| GCG | L-Leucine |
| CASP3 | L-Valine |
| COMT | L-Valine |
