# Supplementary material for: Network Pharmacology and Metabolomics Studies on Antimigraine Mechanisms of Da Chuan Xiong Fang (DCXF)
Source: Evid Based Complement Alternat Med. 2021 Apr 20;2021:6665137. doi: 10.1155/2021/6665137 (PMC8081595; doi:10.1155/2021/6665137)
Supplement: Supplementary Materials — Supplementary S1: preparation, quality control, and HPLC of DCXF, GE, and LC. Supplementary S2: ingredients from LC and GE. Supplementary S3: QED results of GE and LC. Supplementary S4: 531 core targets. Supplementary S5: migraine genes. Supplementary S6: ARRIVE statement for animal experiments. Supplementary S7: metabolites of serum of brain tissue. Supplementary S8: all active ingredients molecular docking results. Supplementary S9: results of MCODE. Supplementary S10: effect of DCXF on serum and brain tissue metabolic profiling. Supplementary S11: gene-metabolite interaction network. Supplementary S12: GTEx RNA-seq data to verify the expression of hub genes in the brain tissues. [file 6665137.f1.zip › 6665137.f1/Supplementary S2 Ingredients from LC and GE.docx]

**Supplementary S2 Ingredients from LC and GE**

We collected 49 and 12 chemical ingredients of from LC and GE, respectively (Supplementary S2 Ingredients from LC and GE) and their Pubchem ids, structures, names were retrieved from the PubChem database (https://pubchem.ncbi.nlm.nih.gov/).

Table 1 ingredients from Tian Ma/GE [*Gastrodia elata Blume*]

| **No.** | **CID** | **ChemName** |
| --- | --- | --- |
| 1 | 6325360 | 7-hydroxybiopterin |
| 2 | 97472 | 4-hydroxybenzylamine |
| 3 | 125 | p-hydroxybenzyl alcohol |
| 4 | 1183 | vanillin |
| 5 | 126 | p-hydroxybenzaldehyde |
| 6 | 62348 | vanillyl alcohol |
| 7 | 7794 | citronellal |
| 8 | 5318157 | 4-(4'-hydroxybenzyloxy)benzyl methyl ether |
| 9 | 5988 | sucrose |
| 10 | 5317235 | ethoxysanguinarine |
| 11 | 5315477 | bis(4-hydroxybenzyl) ether |
| 12 | 10393 | 4-Hydroxyphenethyl alcohol |

Table 2 ingredients from Chuan Xiong/LC [*Ligusticum striatum DC.*]

| **1** | CID | **ChemName** |
| --- | --- | --- |
| **1** | 5281520 | alpha-humulene |
| **2** | 12389 | tetradecane |
| **3** | 8181 | methyl palmitate |
| **4** | 61361 | butylphthalide |
| **5** | 6184 | hexanal |
| **6** | 5319022 | z-ligustilide |
| **7** | 689043 | caffeic acid |
| **8** | 6918391 | beta-elemene |
| **9** | 332 | EUG |
| **10** | 11552 | 3-methyl-butanal |
| **11** | 305 | choline |
| **12** | 957 | octanol |
| **13** | 213039 | OCT |
| **14** | 454 | OYA |
| **15** | 14296 | tetramethylpyrazine |
| **16** | 998 | phenylacetaldehyde |
| **17** | 194725 | EIC |
| **18** | 689043 | caffeicacid |
| **19** | 24762 | MYS |
| **20** | 70789239 | ADO |
| **21** | 931 | naphthalene |
| **22** | 5281 | stearic acid |
| **23** | 6918391 | beta-elemene |
| **24** | 335 | o-cresol |
| **25** | 5319022 | z-ligustilide |
| **26** | 5877292 | ligustilide |
| **27** | 445639 | oleic acid |
| **28** | 1174 | uracil |
| **29** | 6549 | linalool |
| **30** | 985 | palmitic acid |
| **31** | 2537 | camphor |
| **32** | 1183 | vanillin |
| **33** | 190 | adenine |
| **34** | 8955 | PLO |
| **35** | 439250 | L-Limonen |
| **36** | 244 | WLN: Q1R |
| **37** | 7148 | WLN: 2VR |
| **38** | 27867 | menthyl acetate |
| **39** | 6654 | alpha-pinene |
| **40** | 26049 | carene |
| **41** | 6327372 | PHB |
| **42** | 7362 | Furol |
| **43** | 8180 | Undekansaeure |
| **44** | 10208 | Crysophanol |
| **45** | 6394 | TML |
| **46** | 6989 | thymol |
| **47** | 3026 | dibutyl phthalate |
| **48** | 31289 | Nonanal |
| **49** | 445858 | Ferulic acid |
