# Supplementary material for: Network Pharmacology and Metabolomics Studies on Antimigraine Mechanisms of Da Chuan Xiong Fang (DCXF)
Source: Evid Based Complement Alternat Med. 2021 Apr 20;2021:6665137. doi: 10.1155/2021/6665137 (PMC8081595; doi:10.1155/2021/6665137)
Supplement: Supplementary Materials — Supplementary S1: preparation, quality control, and HPLC of DCXF, GE, and LC. Supplementary S2: ingredients from LC and GE. Supplementary S3: QED results of GE and LC. Supplementary S4: 531 core targets. Supplementary S5: migraine genes. Supplementary S6: ARRIVE statement for animal experiments. Supplementary S7: metabolites of serum of brain tissue. Supplementary S8: all active ingredients molecular docking results. Supplementary S9: results of MCODE. Supplementary S10: effect of DCXF on serum and brain tissue metabolic profiling. Supplementary S11: gene-metabolite interaction network. Supplementary S12: GTEx RNA-seq data to verify the expression of hub genes in the brain tissues. [file 6665137.f1.zip › 6665137.f1/Supplementary S3 QED results of GE and LC.docx]

**Supplementary S3 QED results of GE and LC**

The quantitative estimate of drug-likeness (QED) was calculated to prescreen pharmaceutically active compounds in DCXF. Those compounds with QED > 0.3 were chosen for further target prediction analysis. After filtering redundant information, we obtained 46 ingredients, including 44 and 2 compounds from LC and GE respectively. All chemical data were then used as a data source for target prediction.

Table 1 QED results of GE and LC

| herb | chemical_name | gene list | inchikey | database | QED_DES |
| --- | --- | --- | --- | --- | --- |
| GE | p-hydroxybenzaldehyde | ALDH5A1 | RGHHSNMVTDWUBI-UHFFFAOYSA-N | TCMID | 0.5991092 |
| GE | vanillin | KCNK3 | MWOOGOJBHIARFG-UHFFFAOYSA-N | TCMID | 0.600522 |
| GE | vanillin | MMP9 | MWOOGOJBHIARFG-UHFFFAOYSA-N | TCMID | 0.600522 |
| GE | vanillin | TRPV3 | MWOOGOJBHIARFG-UHFFFAOYSA-N | TCMID | 0.600522 |
| LC | PHB | CA2 | FJKROLUGYXJWQN-UHFFFAOYSA-N | STITCH | 0.601439227 |
| LC | PHB | COQ2 | FJKROLUGYXJWQN-UHFFFAOYSA-N | STITCH | 0.601439227 |
| LC | PHB | COQ6 | FJKROLUGYXJWQN-UHFFFAOYSA-N | STITCH | 0.601439227 |
| LC | PHB | PDSS2 | FJKROLUGYXJWQN-UHFFFAOYSA-N | STITCH | 0.601439227 |
| LC | PHB | PDSS1 | FJKROLUGYXJWQN-UHFFFAOYSA-N | STITCH | 0.601439227 |
| LC | Furol | HBA1 | HYBBIBNJHNGZAN-UHFFFAOYSA-N | STITCH | 0.559832598 |
| LC | Furol | TBXT | HYBBIBNJHNGZAN-UHFFFAOYSA-N | STITCH | 0.559832598 |
| LC | Furol | HBB | HYBBIBNJHNGZAN-UHFFFAOYSA-N | STITCH | 0.559832598 |
| LC | caffeic acid | MIF | QAIPRVGONGVQAS-DUXPYHPUSA-N | STITCH | 0.457601063 |
| LC | caffeic acid | MAPK1 | QAIPRVGONGVQAS-DUXPYHPUSA-N | STITCH | 0.457601063 |
| LC | caffeic acid | TYR | QAIPRVGONGVQAS-DUXPYHPUSA-N | STITCH | 0.457601063 |
| LC | caffeic acid | MAPK8 | QAIPRVGONGVQAS-DUXPYHPUSA-N | STITCH | 0.457601063 |
| LC | caffeic acid | COMT | QAIPRVGONGVQAS-DUXPYHPUSA-N | STITCH | 0.457601063 |
| LC | caffeic acid | ALOX5 | QAIPRVGONGVQAS-DUXPYHPUSA-N | STITCH | 0.457601063 |
| LC | L-Limonen | CYP2C9 | XMGQYMWWDOXHJM-SNVBAGLBSA-N | STITCH | 0.279255003 |
| LC | L-Limonen | CYP2C19 | XMGQYMWWDOXHJM-SNVBAGLBSA-N | STITCH | 0.279255003 |
| LC | vanillin | KCNK3 | MWOOGOJBHIARFG-UHFFFAOYSA-N | STITCH | 0.600521992 |
| LC | vanillin | MMP9 | MWOOGOJBHIARFG-UHFFFAOYSA-N | STITCH | 0.600521992 |
| LC | vanillin | TRPV3 | MWOOGOJBHIARFG-UHFFFAOYSA-N | STITCH | 0.600521992 |
| LC | hexanal | FOS | JARKCYVAAOWBJS-UHFFFAOYSA-N | STITCH | 0.348869365 |
| LC | hexanal | CYP1A1 | JARKCYVAAOWBJS-UHFFFAOYSA-N | STITCH | 0.348869365 |
| LC | WLN: Q1R | CES2 | WVDDGKGOMKODPV-UHFFFAOYSA-N | STITCH | 0.516392345 |
| LC | WLN: Q1R | CES1 | WVDDGKGOMKODPV-UHFFFAOYSA-N | STITCH | 0.516392345 |
| LC | WLN: Q1R | CES5A | WVDDGKGOMKODPV-UHFFFAOYSA-N | STITCH | 0.516392345 |
| LC | WLN: Q1R | CES4A | WVDDGKGOMKODPV-UHFFFAOYSA-N | STITCH | 0.516392345 |
| LC | Crysophanol | NLRP3 | LQGUBLBATBMXHT-UHFFFAOYSA-N | STITCH | 0.522522125 |
| LC | uracil | DHODH | ISAKRJDGNUQOIC-UHFFFAOYSA-N | STITCH | 0.45316348 |
| LC | uracil | PUS3 | ISAKRJDGNUQOIC-UHFFFAOYSA-N | STITCH | 0.45316348 |
| LC | uracil | UNG | ISAKRJDGNUQOIC-UHFFFAOYSA-N | STITCH | 0.45316348 |
| LC | uracil | MBD4 | ISAKRJDGNUQOIC-UHFFFAOYSA-N | STITCH | 0.45316348 |
| LC | uracil | TYMP | ISAKRJDGNUQOIC-UHFFFAOYSA-N | STITCH | 0.45316348 |
| LC | uracil | GPR17 | ISAKRJDGNUQOIC-UHFFFAOYSA-N | STITCH | 0.45316348 |
| LC | uracil | DPYS | ISAKRJDGNUQOIC-UHFFFAOYSA-N | STITCH | 0.45316348 |
| LC | uracil | CCNO | ISAKRJDGNUQOIC-UHFFFAOYSA-N | STITCH | 0.45316348 |
| LC | uracil | TRUB1 | ISAKRJDGNUQOIC-UHFFFAOYSA-N | STITCH | 0.45316348 |
| LC | uracil | JUP | ISAKRJDGNUQOIC-UHFFFAOYSA-N | STITCH | 0.45316348 |
| LC | uracil | TYMS | ISAKRJDGNUQOIC-UHFFFAOYSA-N | STITCH | 0.45316348 |
| LC | uracil | SMUG1 | ISAKRJDGNUQOIC-UHFFFAOYSA-N | STITCH | 0.45316348 |
| LC | uracil | CTNNB1 | ISAKRJDGNUQOIC-UHFFFAOYSA-N | STITCH | 0.45316348 |
| LC | uracil | UCKL1 | ISAKRJDGNUQOIC-UHFFFAOYSA-N | STITCH | 0.45316348 |
| LC | uracil | PUS7 | ISAKRJDGNUQOIC-UHFFFAOYSA-N | STITCH | 0.45316348 |
| LC | uracil | SLC29A2 | ISAKRJDGNUQOIC-UHFFFAOYSA-N | STITCH | 0.45316348 |
| LC | uracil | PNP | ISAKRJDGNUQOIC-UHFFFAOYSA-N | STITCH | 0.45316348 |
| LC | uracil | DPYD | ISAKRJDGNUQOIC-UHFFFAOYSA-N | STITCH | 0.45316348 |
| LC | uracil | UPRT | ISAKRJDGNUQOIC-UHFFFAOYSA-N | STITCH | 0.45316348 |
| LC | uracil | TDG | ISAKRJDGNUQOIC-UHFFFAOYSA-N | STITCH | 0.45316348 |
| LC | uracil | POLD1 | ISAKRJDGNUQOIC-UHFFFAOYSA-N | STITCH | 0.45316348 |
| LC | EUG | IL6 | YOMSJEATGXXYPX-UHFFFAOYSA-N | STITCH | 0.616736593 |
| LC | adenine | MRI1 | GFFGJBXGBJISGV-UHFFFAOYSA-N | STITCH | 0.44729963 |
| LC | adenine | PYGM | GFFGJBXGBJISGV-UHFFFAOYSA-N | STITCH | 0.44729963 |
| LC | adenine | ALKBH1 | GFFGJBXGBJISGV-UHFFFAOYSA-N | STITCH | 0.44729963 |
| LC | adenine | SF3B6 | GFFGJBXGBJISGV-UHFFFAOYSA-N | STITCH | 0.44729963 |
| LC | adenine | ALKBH7 | GFFGJBXGBJISGV-UHFFFAOYSA-N | STITCH | 0.44729963 |
| LC | adenine | PECR | GFFGJBXGBJISGV-UHFFFAOYSA-N | STITCH | 0.44729963 |
| LC | adenine | PNMT | GFFGJBXGBJISGV-UHFFFAOYSA-N | STITCH | 0.44729963 |
| LC | adenine | ACP1 | GFFGJBXGBJISGV-UHFFFAOYSA-N | STITCH | 0.44729963 |
| LC | adenine | ALKBH4 | GFFGJBXGBJISGV-UHFFFAOYSA-N | STITCH | 0.44729963 |
| LC | adenine | HPRT1 | GFFGJBXGBJISGV-UHFFFAOYSA-N | STITCH | 0.44729963 |
| LC | adenine | ALKBH3 | GFFGJBXGBJISGV-UHFFFAOYSA-N | STITCH | 0.44729963 |
| LC | adenine | P2RY1 | GFFGJBXGBJISGV-UHFFFAOYSA-N | STITCH | 0.44729963 |
| LC | adenine | KNTC1 | GFFGJBXGBJISGV-UHFFFAOYSA-N | STITCH | 0.44729963 |
| LC | adenine | HSP90AA1 | GFFGJBXGBJISGV-UHFFFAOYSA-N | STITCH | 0.44729963 |
| LC | adenine | SF3B1 | GFFGJBXGBJISGV-UHFFFAOYSA-N | STITCH | 0.44729963 |
| LC | adenine | ACVR2B | GFFGJBXGBJISGV-UHFFFAOYSA-N | STITCH | 0.44729963 |
| LC | adenine | ACACB | GFFGJBXGBJISGV-UHFFFAOYSA-N | STITCH | 0.44729963 |
| LC | adenine | SLC29A2 | GFFGJBXGBJISGV-UHFFFAOYSA-N | STITCH | 0.44729963 |
| LC | adenine | PNP | GFFGJBXGBJISGV-UHFFFAOYSA-N | STITCH | 0.44729963 |
| LC | adenine | MUTYH | GFFGJBXGBJISGV-UHFFFAOYSA-N | STITCH | 0.44729963 |
| LC | adenine | SLC29A3 | GFFGJBXGBJISGV-UHFFFAOYSA-N | STITCH | 0.44729963 |
| LC | adenine | STK24 | GFFGJBXGBJISGV-UHFFFAOYSA-N | STITCH | 0.44729963 |
| LC | adenine | APRT | GFFGJBXGBJISGV-UHFFFAOYSA-N | STITCH | 0.44729963 |
| LC | adenine | MTAP | GFFGJBXGBJISGV-UHFFFAOYSA-N | STITCH | 0.44729963 |
| LC | adenine | ALKBH8 | GFFGJBXGBJISGV-UHFFFAOYSA-N | STITCH | 0.44729963 |
| LC | adenine | SRPK2 | GFFGJBXGBJISGV-UHFFFAOYSA-N | STITCH | 0.44729963 |
| LC | adenine | ALKBH5 | GFFGJBXGBJISGV-UHFFFAOYSA-N | STITCH | 0.44729963 |
| LC | adenine | CHFR | GFFGJBXGBJISGV-UHFFFAOYSA-N | STITCH | 0.44729963 |
| LC | adenine | XPNPEP1 | GFFGJBXGBJISGV-UHFFFAOYSA-N | STITCH | 0.44729963 |
| LC | PLO | SULT2B1 | ORNBQBCIOKFOEO-QGVNFLHTSA-N | STITCH | 0.485574093 |
| LC | PLO | STS | ORNBQBCIOKFOEO-QGVNFLHTSA-N | STITCH | 0.485574093 |
| LC | PLO | LHB | ORNBQBCIOKFOEO-QGVNFLHTSA-N | STITCH | 0.485574093 |
| LC | PLO | SULT2A1 | ORNBQBCIOKFOEO-QGVNFLHTSA-N | STITCH | 0.485574093 |
| LC | PLO | POMC | ORNBQBCIOKFOEO-QGVNFLHTSA-N | STITCH | 0.485574093 |
| LC | PLO | CYP11A1 | ORNBQBCIOKFOEO-QGVNFLHTSA-N | STITCH | 0.485574093 |
| LC | PLO | STAR | ORNBQBCIOKFOEO-QGVNFLHTSA-N | STITCH | 0.485574093 |
| LC | PLO | CLIP1 | ORNBQBCIOKFOEO-QGVNFLHTSA-N | STITCH | 0.485574093 |
| LC | PLO | CYP7B1 | ORNBQBCIOKFOEO-QGVNFLHTSA-N | STITCH | 0.485574093 |
| LC | PLO | BPNT1 | ORNBQBCIOKFOEO-QGVNFLHTSA-N | STITCH | 0.485574093 |
| LC | PLO | NR1I2 | ORNBQBCIOKFOEO-QGVNFLHTSA-N | STITCH | 0.485574093 |
| LC | PLO | HSD3B1 | ORNBQBCIOKFOEO-QGVNFLHTSA-N | STITCH | 0.485574093 |
| LC | PLO | HSD3B2 | ORNBQBCIOKFOEO-QGVNFLHTSA-N | STITCH | 0.485574093 |
| LC | PLO | CYP17A1 | ORNBQBCIOKFOEO-QGVNFLHTSA-N | STITCH | 0.485574093 |
| LC | PLO | TRPM3 | ORNBQBCIOKFOEO-QGVNFLHTSA-N | STITCH | 0.485574093 |
| LC | PLO | CYP21A2 | ORNBQBCIOKFOEO-QGVNFLHTSA-N | STITCH | 0.485574093 |
| LC | TML | KIF11 | GETQZCLCWQTVFV-UHFFFAOYSA-N | STITCH | 0.39383493 |
| LC | TML | FMO3 | GETQZCLCWQTVFV-UHFFFAOYSA-N | STITCH | 0.39383493 |
| LC | WLN: 2VR | ALDH3A1 | KRIOVPPHQSLHCZ-UHFFFAOYSA-N | STITCH | 0.496781652 |
| LC | tetramethylpyrazine | CXCL8 | FINHMKGKINIASC-UHFFFAOYSA-N | STITCH | 0.481345804 |
| LC | tetramethylpyrazine | HIF1A | FINHMKGKINIASC-UHFFFAOYSA-N | STITCH | 0.481345804 |
| LC | menthyl acetate | CYP3A4 | XHXUANMFYXWVNG-WCQGTBRESA-N | HIT | 0.498055753 |
| LC | z-ligustilide | ACHE | WMBOCUXXNSOQHM-FLIBITNWSA-N | HIT | 0.572637473 |
| LC | z-ligustilide | SOD1 | WMBOCUXXNSOQHM-FLIBITNWSA-N | HIT | 0.572637473 |
| LC | z-ligustilide | CHAT | WMBOCUXXNSOQHM-FLIBITNWSA-N | HIT | 0.572637473 |
| LC | z-ligustilide | BAX | WMBOCUXXNSOQHM-FLIBITNWSA-N | HIT | 0.572637473 |
| LC | z-ligustilide | CASP3 | WMBOCUXXNSOQHM-FLIBITNWSA-N | HIT | 0.572637473 |
| LC | z-ligustilide | BCL2 | WMBOCUXXNSOQHM-FLIBITNWSA-N | HIT | 0.572637473 |
| LC | z-ligustilide | RELA | WMBOCUXXNSOQHM-FLIBITNWSA-N | HIT | 0.572637473 |
| LC | z-ligustilide | TNF | WMBOCUXXNSOQHM-FLIBITNWSA-N | HIT | 0.572637473 |
| LC | carene | SPP1 | BQOFWKZOCNGFEC-UHFFFAOYSA-N | HIT | 0.306764373 |
| LC | carene | COL1A1 | BQOFWKZOCNGFEC-UHFFFAOYSA-N | HIT | 0.306764373 |
| LC | butylphthalide | BDNF | HJXMNVQARNZTEE-UHFFFAOYSA-N | HIT | 0.576360989 |
| LC | butylphthalide | NGF | HJXMNVQARNZTEE-UHFFFAOYSA-N | HIT | 0.576360989 |
| LC | thymol | ELANE | MGSRCZKZVOBKFT-UHFFFAOYSA-N | HIT | 0.482797915 |
| LC | linalool | KATNA1 | CDOSHBSSFJOMGT-UHFFFAOYSA-N | HIT | 0.379889074 |
| LC | linalool | ADORA2A | CDOSHBSSFJOMGT-UHFFFAOYSA-N | HIT | 0.379889074 |
| LC | linalool | NOS2 | CDOSHBSSFJOMGT-UHFFFAOYSA-N | HIT | 0.379889074 |
| LC | linalool | PTGS2 | CDOSHBSSFJOMGT-UHFFFAOYSA-N | HIT | 0.379889074 |
| LC | tetramethylpyrazine | ITGA2B | FINHMKGKINIASC-UHFFFAOYSA-N | HIT | 0.481345804 |
| LC | tetramethylpyrazine | VEGFA | FINHMKGKINIASC-UHFFFAOYSA-N | HIT | 0.481345804 |
| LC | tetramethylpyrazine | HIF1A | FINHMKGKINIASC-UHFFFAOYSA-N | HIT | 0.481345804 |
| LC | tetramethylpyrazine | TBXAS1 | FINHMKGKINIASC-UHFFFAOYSA-N | HIT | 0.481345804 |
| LC | camphor | CHRNA4 | DSSYKIVIOFKYAU-UHFFFAOYSA-N | HIT | 0.403737157 |
| LC | naphthalene | CYP1A1 | UFWIBTONFRDIAS-UHFFFAOYSA-N | HIT | 0.36872104 |
| LC | naphthalene | UCHL1 | UFWIBTONFRDIAS-UHFFFAOYSA-N | HIT | 0.36872104 |
| LC | naphthalene | EIF6 | UFWIBTONFRDIAS-UHFFFAOYSA-N | HIT | 0.36872104 |
| LC | naphthalene | MPO | UFWIBTONFRDIAS-UHFFFAOYSA-N | HIT | 0.36872104 |
| LC | naphthalene | PRDX5 | UFWIBTONFRDIAS-UHFFFAOYSA-N | HIT | 0.36872104 |
| LC | naphthalene | FUS | UFWIBTONFRDIAS-UHFFFAOYSA-N | HIT | 0.36872104 |
| LC | naphthalene | BCL2 | UFWIBTONFRDIAS-UHFFFAOYSA-N | HIT | 0.36872104 |
| LC | naphthalene | JUN | UFWIBTONFRDIAS-UHFFFAOYSA-N | HIT | 0.36872104 |
| LC | naphthalene | FOS | UFWIBTONFRDIAS-UHFFFAOYSA-N | HIT | 0.36872104 |
| LC | naphthalene | RAF1 | UFWIBTONFRDIAS-UHFFFAOYSA-N | HIT | 0.36872104 |
| LC | naphthalene | CXCL8 | UFWIBTONFRDIAS-UHFFFAOYSA-N | HIT | 0.36872104 |
| LC | octanol | PRKCB | KBPLFHHGFOOTCA-UHFFFAOYSA-N | HIT | 0.293420166 |
| LC | o-cresol | PTGS1 | QWVGKYWNOKOFNN-UHFFFAOYSA-N | HIT | 0.494060487 |
| LC | o-cresol | PTGS2 | QWVGKYWNOKOFNN-UHFFFAOYSA-N | HIT | 0.494060487 |
| LC | 3-methyl-butanal | ADH1B | YGHRJJRRZDOVPD-UHFFFAOYSA-N | TCMID | 0.442644804 |
| LC | 3-methyl-butanal | ADH6 | YGHRJJRRZDOVPD-UHFFFAOYSA-N | TCMID | 0.442644804 |
| LC | vanillin | KCNK3 | MWOOGOJBHIARFG-UHFFFAOYSA-N | TCMID | 0.600521992 |
| LC | vanillin | MMP9 | MWOOGOJBHIARFG-UHFFFAOYSA-N | TCMID | 0.600521992 |
| LC | vanillin | TRPV3 | MWOOGOJBHIARFG-UHFFFAOYSA-N | TCMID | 0.600521992 |
| LC | choline | ADH1A | OEYIOHPDSNJKLS-UHFFFAOYSA-N | TCMID | 0.415186794 |
| LC | choline | RAB3A | OEYIOHPDSNJKLS-UHFFFAOYSA-N | TCMID | 0.415186794 |
| LC | choline | CLEC2B | OEYIOHPDSNJKLS-UHFFFAOYSA-N | TCMID | 0.415186794 |
| LC | choline | PLBD1 | OEYIOHPDSNJKLS-UHFFFAOYSA-N | TCMID | 0.415186794 |
| LC | choline | SYT1 | OEYIOHPDSNJKLS-UHFFFAOYSA-N | TCMID | 0.415186794 |
| LC | choline | PLD2 | OEYIOHPDSNJKLS-UHFFFAOYSA-N | TCMID | 0.415186794 |
| LC | choline | SLC5A7 | OEYIOHPDSNJKLS-UHFFFAOYSA-N | TCMID | 0.415186794 |
| LC | choline | BCHE | OEYIOHPDSNJKLS-UHFFFAOYSA-N | TCMID | 0.415186794 |
| LC | choline | ADH4 | OEYIOHPDSNJKLS-UHFFFAOYSA-N | TCMID | 0.415186794 |
| LC | choline | CHKA | OEYIOHPDSNJKLS-UHFFFAOYSA-N | TCMID | 0.415186794 |
| LC | choline | ETNK1 | OEYIOHPDSNJKLS-UHFFFAOYSA-N | TCMID | 0.415186794 |
| LC | choline | SLC44A3 | OEYIOHPDSNJKLS-UHFFFAOYSA-N | TCMID | 0.415186794 |
| LC | choline | REN | OEYIOHPDSNJKLS-UHFFFAOYSA-N | TCMID | 0.415186794 |
| LC | choline | BHMT | OEYIOHPDSNJKLS-UHFFFAOYSA-N | TCMID | 0.415186794 |
| LC | choline | PLA2G4D | OEYIOHPDSNJKLS-UHFFFAOYSA-N | TCMID | 0.415186794 |
| LC | choline | PCYT1A | OEYIOHPDSNJKLS-UHFFFAOYSA-N | TCMID | 0.415186794 |
| LC | choline | ADH5 | OEYIOHPDSNJKLS-UHFFFAOYSA-N | TCMID | 0.415186794 |
| LC | choline | ACHE | OEYIOHPDSNJKLS-UHFFFAOYSA-N | TCMID | 0.415186794 |
| LC | choline | PRKCE | OEYIOHPDSNJKLS-UHFFFAOYSA-N | TCMID | 0.415186794 |
| LC | choline | ADH1B | OEYIOHPDSNJKLS-UHFFFAOYSA-N | TCMID | 0.415186794 |
| LC | choline | VAMP2 | OEYIOHPDSNJKLS-UHFFFAOYSA-N | TCMID | 0.415186794 |
| LC | choline | PLD6 | OEYIOHPDSNJKLS-UHFFFAOYSA-N | TCMID | 0.415186794 |
| LC | choline | CHDH | OEYIOHPDSNJKLS-UHFFFAOYSA-N | TCMID | 0.415186794 |
| LC | choline | PRKCD | OEYIOHPDSNJKLS-UHFFFAOYSA-N | TCMID | 0.415186794 |
| LC | choline | GRIN2A | OEYIOHPDSNJKLS-UHFFFAOYSA-N | TCMID | 0.415186794 |
| LC | choline | SLC44A2 | OEYIOHPDSNJKLS-UHFFFAOYSA-N | TCMID | 0.415186794 |
| LC | choline | CHAT | OEYIOHPDSNJKLS-UHFFFAOYSA-N | TCMID | 0.415186794 |
| LC | choline | PLD1 | OEYIOHPDSNJKLS-UHFFFAOYSA-N | TCMID | 0.415186794 |
| LC | choline | PLD3 | OEYIOHPDSNJKLS-UHFFFAOYSA-N | TCMID | 0.415186794 |
| LC | choline | SLC22A2 | OEYIOHPDSNJKLS-UHFFFAOYSA-N | TCMID | 0.415186794 |
| LC | choline | SLC22A1 | OEYIOHPDSNJKLS-UHFFFAOYSA-N | TCMID | 0.415186794 |
| LC | choline | SLC44A5 | OEYIOHPDSNJKLS-UHFFFAOYSA-N | TCMID | 0.415186794 |
| LC | choline | SLC18A3 | OEYIOHPDSNJKLS-UHFFFAOYSA-N | TCMID | 0.415186794 |
| LC | choline | SLC44A1 | OEYIOHPDSNJKLS-UHFFFAOYSA-N | TCMID | 0.415186794 |
| LC | choline | PLA2G2F | OEYIOHPDSNJKLS-UHFFFAOYSA-N | TCMID | 0.415186794 |
| LC | choline | PLA2G2A | OEYIOHPDSNJKLS-UHFFFAOYSA-N | TCMID | 0.415186794 |
| LC | choline | SUV39H1 | OEYIOHPDSNJKLS-UHFFFAOYSA-N | TCMID | 0.415186794 |
| LC | choline | GPCPD1 | OEYIOHPDSNJKLS-UHFFFAOYSA-N | TCMID | 0.415186794 |
| LC | choline | PCYT1B | OEYIOHPDSNJKLS-UHFFFAOYSA-N | TCMID | 0.415186794 |
| LC | choline | PLD4 | OEYIOHPDSNJKLS-UHFFFAOYSA-N | TCMID | 0.415186794 |
| LC | choline | ADH6 | OEYIOHPDSNJKLS-UHFFFAOYSA-N | TCMID | 0.415186794 |
| LC | choline | PLPP4 | OEYIOHPDSNJKLS-UHFFFAOYSA-N | TCMID | 0.415186794 |
| LC | choline | PLA2G4E | OEYIOHPDSNJKLS-UHFFFAOYSA-N | TCMID | 0.415186794 |
| LC | choline | CHKB | OEYIOHPDSNJKLS-UHFFFAOYSA-N | TCMID | 0.415186794 |
| LC | choline | GCG | OEYIOHPDSNJKLS-UHFFFAOYSA-N | TCMID | 0.415186794 |
| LC | choline | PLA2G4B | OEYIOHPDSNJKLS-UHFFFAOYSA-N | TCMID | 0.415186794 |
| LC | choline | TNF | OEYIOHPDSNJKLS-UHFFFAOYSA-N | TCMID | 0.415186794 |
| LC | choline | PHOSPHO1 | OEYIOHPDSNJKLS-UHFFFAOYSA-N | TCMID | 0.415186794 |
| LC | choline | CHRNA7 | OEYIOHPDSNJKLS-UHFFFAOYSA-N | TCMID | 0.415186794 |
| LC | choline | BDNF | OEYIOHPDSNJKLS-UHFFFAOYSA-N | TCMID | 0.415186794 |
| LC | choline | ADH7 | OEYIOHPDSNJKLS-UHFFFAOYSA-N | TCMID | 0.415186794 |
| LC | choline | PTDSS1 | OEYIOHPDSNJKLS-UHFFFAOYSA-N | TCMID | 0.415186794 |
| LC | z-ligustilide | SST | IQVQXVFMNOFTMU-UHFFFAOYSA-N | TCMID | 0.528597431 |
| LC | z-ligustilide | KL | IQVQXVFMNOFTMU-UHFFFAOYSA-N | TCMID | 0.528597431 |
| LC | dibutyl phthalate | NR1I3 | DOIRQSBPFJWKBE-UHFFFAOYSA-N | TCMID | 0.367112698 |
| LC | caffeicacid | TYR | QAIPRVGONGVQAS-RQOWECAXSA-N | TCMID | 0.457601063 |
| LC | alpha-pinene | CYP2B6 | GRWFGVWFFZKLTI-RKDXNWHRSA-N | TCMID | 0.306846137 |
| LC | caproaldehyde | FOS | JARKCYVAAOWBJS-UHFFFAOYSA-N | TCMID | 0.399464964 |
| LC | caproaldehyde | CYP1A1 | JARKCYVAAOWBJS-UHFFFAOYSA-N | TCMID | 0.399464964 |
| LC | chuanxiongzine | CXCL8 | FINHMKGKINIASC-UHFFFAOYSA-N | TCMID | 0.481345804 |
| LC | chuanxiongzine | HIF1A | FINHMKGKINIASC-UHFFFAOYSA-N | TCMID | 0.481345804 |
| LC | limonene | CYP2C9 | XMGQYMWWDOXHJM-SNVBAGLBSA-N | TCMID | 0.279255003 |
| LC | limonene | CYP2C19 | XMGQYMWWDOXHJM-SNVBAGLBSA-N | TCMID | 0.279255003 |
| LC | ligustilide | SST | IQVQXVFMNOFTMU-FLIBITNWSA-N | TCMID | 0.528597431 |
| LC | ligustilide | KL | IQVQXVFMNOFTMU-FLIBITNWSA-N | TCMID | 0.528597431 |
| LC | phenylacetaldehyde | ALDH3A1 | DTUQWGWMVIHBKE-UHFFFAOYSA-N | TCMID | 0.488150371 |
| LC | phenylacetaldehyde | AOC2 | DTUQWGWMVIHBKE-UHFFFAOYSA-N | TCMID | 0.488150371 |
| LC | phenylacetaldehyde | ALDH3B2 | DTUQWGWMVIHBKE-UHFFFAOYSA-N | TCMID | 0.488150371 |
| LC | phenylacetaldehyde | ALDH2 | DTUQWGWMVIHBKE-UHFFFAOYSA-N | TCMID | 0.488150371 |
| LC | phenylacetaldehyde | FURIN | DTUQWGWMVIHBKE-UHFFFAOYSA-N | TCMID | 0.488150371 |
| LC | phenylacetaldehyde | ADH1B | DTUQWGWMVIHBKE-UHFFFAOYSA-N | TCMID | 0.488150371 |
| LC | phenylacetaldehyde | AOC3 | DTUQWGWMVIHBKE-UHFFFAOYSA-N | TCMID | 0.488150371 |
| LC | phenylacetaldehyde | ALDH1A3 | DTUQWGWMVIHBKE-UHFFFAOYSA-N | TCMID | 0.488150371 |
| LC | phenylacetaldehyde | MAOA | DTUQWGWMVIHBKE-UHFFFAOYSA-N | TCMID | 0.488150371 |
| LC | phenylacetaldehyde | ALDH3A2 | DTUQWGWMVIHBKE-UHFFFAOYSA-N | TCMID | 0.488150371 |
| LC | phenylacetaldehyde | MAOB | DTUQWGWMVIHBKE-UHFFFAOYSA-N | TCMID | 0.488150371 |
| LC | phenylacetaldehyde | ADH6 | DTUQWGWMVIHBKE-UHFFFAOYSA-N | TCMID | 0.488150371 |
| LC | butylphthalide | MPO | HJXMNVQARNZTEE-UHFFFAOYSA-N | HIT | 0.758318025 |
| LC | butylphthalide | ICAM1 | HJXMNVQARNZTEE-UHFFFAOYSA-N | HIT | 0.758318025 |
| LC | butylphthalide | TNF | HJXMNVQARNZTEE-UHFFFAOYSA-N | HIT | 0.758318025 |
| LC | beta-elemene | MAPK13 | OPFTUNCRGUEPRZ-UHFFFAOYSA-N | HIT | 0.279562 |
| LC | beta-elemene | VEGFC | OPFTUNCRGUEPRZ-UHFFFAOYSA-N | HIT | 0.279562 |
| LC | beta-elemene | FLT4 | OPFTUNCRGUEPRZ-UHFFFAOYSA-N | HIT | 0.279562 |
| LC | beta-elemene | EIF4E | OPFTUNCRGUEPRZ-UHFFFAOYSA-N | HIT | 0.279562 |
| LC | beta-elemene | FGF2 | OPFTUNCRGUEPRZ-UHFFFAOYSA-N | HIT | 0.279562 |
| LC | beta-elemene | VEGFA | OPFTUNCRGUEPRZ-UHFFFAOYSA-N | HIT | 0.279562 |
| LC | beta-elemene | CASP3 | OPFTUNCRGUEPRZ-UHFFFAOYSA-N | STITCH | 0.279562 |
| LC | beta-elemene | EIF4G1 | OPFTUNCRGUEPRZ-UHFFFAOYSA-N | HIT | 0.279562 |
| LC | tetramethylpyrazine | CXCL8 | FINHMKGKINIASC-UHFFFAOYSA-N | HIT | 0.581527131 |
| LC | camphor | HEBP1 | DSSYKIVIOFKYAU-UHFFFAOYSA-N | HIT | 0.473229522 |
| LC | camphor | DRD4 | DSSYKIVIOFKYAU-UHFFFAOYSA-N | HIT | 0.473229522 |
| LC | camphor | BDKRB1 | DSSYKIVIOFKYAU-UHFFFAOYSA-N | HIT | 0.473229522 |
| LC | camphor | PDYN | DSSYKIVIOFKYAU-UHFFFAOYSA-N | HIT | 0.473229522 |
| LC | camphor | C5 | DSSYKIVIOFKYAU-UHFFFAOYSA-N | HIT | 0.473229522 |
| LC | camphor | PPY | DSSYKIVIOFKYAU-UHFFFAOYSA-N | HIT | 0.473229522 |
| LC | camphor | CXCL6 | DSSYKIVIOFKYAU-UHFFFAOYSA-N | HIT | 0.473229522 |
| LC | camphor | GRM6 | DSSYKIVIOFKYAU-UHFFFAOYSA-N | HIT | 0.473229522 |
| LC | camphor | OPRD1 | DSSYKIVIOFKYAU-UHFFFAOYSA-N | HIT | 0.473229522 |
| LC | camphor | TAS2R8 | DSSYKIVIOFKYAU-UHFFFAOYSA-N | HIT | 0.473229522 |
| LC | camphor | TAS2R10 | DSSYKIVIOFKYAU-UHFFFAOYSA-N | HIT | 0.473229522 |
| LC | camphor | TAS2R7 | DSSYKIVIOFKYAU-UHFFFAOYSA-N | HIT | 0.473229522 |
| LC | camphor | NPY | DSSYKIVIOFKYAU-UHFFFAOYSA-N | HIT | 0.473229522 |
| LC | camphor | C3 | DSSYKIVIOFKYAU-UHFFFAOYSA-N | HIT | 0.473229522 |
| LC | camphor | S1PR4 | DSSYKIVIOFKYAU-UHFFFAOYSA-N | HIT | 0.473229522 |
| LC | camphor | CCR7 | DSSYKIVIOFKYAU-UHFFFAOYSA-N | HIT | 0.473229522 |
| LC | camphor | TAS2R3 | DSSYKIVIOFKYAU-UHFFFAOYSA-N | HIT | 0.473229522 |
| LC | camphor | TAS2R5 | DSSYKIVIOFKYAU-UHFFFAOYSA-N | HIT | 0.473229522 |
| LC | camphor | MCHR1 | DSSYKIVIOFKYAU-UHFFFAOYSA-N | HIT | 0.473229522 |
| LC | camphor | GALR3 | DSSYKIVIOFKYAU-UHFFFAOYSA-N | HIT | 0.473229522 |
| LC | camphor | TAS2R16 | DSSYKIVIOFKYAU-UHFFFAOYSA-N | HIT | 0.473229522 |
| LC | camphor | ADCY7 | DSSYKIVIOFKYAU-UHFFFAOYSA-N | HIT | 0.473229522 |
| LC | camphor | SSTR4 | DSSYKIVIOFKYAU-UHFFFAOYSA-N | HIT | 0.473229522 |
| LC | camphor | NMUR2 | DSSYKIVIOFKYAU-UHFFFAOYSA-N | HIT | 0.473229522 |
| LC | camphor | HRH4 | DSSYKIVIOFKYAU-UHFFFAOYSA-N | HIT | 0.473229522 |
| LC | camphor | MTNR1B | DSSYKIVIOFKYAU-UHFFFAOYSA-N | HIT | 0.473229522 |
| LC | camphor | APLNR | DSSYKIVIOFKYAU-UHFFFAOYSA-N | HIT | 0.473229522 |
| LC | camphor | ANXA1 | DSSYKIVIOFKYAU-UHFFFAOYSA-N | HIT | 0.473229522 |
| LC | camphor | CCL21 | DSSYKIVIOFKYAU-UHFFFAOYSA-N | HIT | 0.473229522 |
| LC | camphor | CCL27 | DSSYKIVIOFKYAU-UHFFFAOYSA-N | HIT | 0.473229522 |
| LC | camphor | ADCY3 | DSSYKIVIOFKYAU-UHFFFAOYSA-N | HIT | 0.473229522 |
| LC | camphor | TRPA1 | DSSYKIVIOFKYAU-UHFFFAOYSA-N | HIT | 0.473229522 |
| LC | camphor | NMU | DSSYKIVIOFKYAU-UHFFFAOYSA-N | HIT | 0.473229522 |
| LC | camphor | POMC | DSSYKIVIOFKYAU-UHFFFAOYSA-N | HIT | 0.473229522 |
| LC | camphor | KNG1 | DSSYKIVIOFKYAU-UHFFFAOYSA-N | HIT | 0.473229522 |
| LC | camphor | OPRK1 | DSSYKIVIOFKYAU-UHFFFAOYSA-N | HIT | 0.473229522 |
| LC | camphor | GAL | DSSYKIVIOFKYAU-UHFFFAOYSA-N | HIT | 0.473229522 |
| LC | camphor | SSTR1 | DSSYKIVIOFKYAU-UHFFFAOYSA-N | HIT | 0.473229522 |
| LC | camphor | GPR17 | DSSYKIVIOFKYAU-UHFFFAOYSA-N | HIT | 0.473229522 |
| LC | camphor | ACKR3 | DSSYKIVIOFKYAU-UHFFFAOYSA-N | HIT | 0.473229522 |
| LC | camphor | ADRA2A | DSSYKIVIOFKYAU-UHFFFAOYSA-N | HIT | 0.473229522 |
| LC | camphor | MCHR2 | DSSYKIVIOFKYAU-UHFFFAOYSA-N | HIT | 0.473229522 |
| LC | camphor | APP | DSSYKIVIOFKYAU-UHFFFAOYSA-N | HIT | 0.473229522 |
| LC | camphor | ADCY8 | DSSYKIVIOFKYAU-UHFFFAOYSA-N | HIT | 0.473229522 |
| LC | camphor | CXCL13 | DSSYKIVIOFKYAU-UHFFFAOYSA-N | HIT | 0.473229522 |
| LC | camphor | SST | DSSYKIVIOFKYAU-UHFFFAOYSA-N | HIT | 0.473229522 |
| LC | camphor | HTR5A | DSSYKIVIOFKYAU-UHFFFAOYSA-N | HIT | 0.473229522 |
| LC | camphor | CXCR5 | DSSYKIVIOFKYAU-UHFFFAOYSA-N | HIT | 0.473229522 |
| LC | camphor | CCR2 | DSSYKIVIOFKYAU-UHFFFAOYSA-N | HIT | 0.473229522 |
| LC | camphor | CCR5 | DSSYKIVIOFKYAU-UHFFFAOYSA-N | HIT | 0.473229522 |
| LC | camphor | CXCL16 | DSSYKIVIOFKYAU-UHFFFAOYSA-N | HIT | 0.473229522 |
| LC | camphor | SSTR5 | DSSYKIVIOFKYAU-UHFFFAOYSA-N | HIT | 0.473229522 |
| LC | camphor | ADCY9 | DSSYKIVIOFKYAU-UHFFFAOYSA-N | HIT | 0.473229522 |
| LC | camphor | CXCR1 | DSSYKIVIOFKYAU-UHFFFAOYSA-N | HIT | 0.473229522 |
| LC | camphor | CXCL3 | DSSYKIVIOFKYAU-UHFFFAOYSA-N | HIT | 0.473229522 |
| LC | camphor | CXCL5 | DSSYKIVIOFKYAU-UHFFFAOYSA-N | HIT | 0.473229522 |
| LC | camphor | PPBP | DSSYKIVIOFKYAU-UHFFFAOYSA-N | HIT | 0.473229522 |
| LC | camphor | CCR1 | DSSYKIVIOFKYAU-UHFFFAOYSA-N | HIT | 0.473229522 |
| LC | camphor | RGS20 | DSSYKIVIOFKYAU-UHFFFAOYSA-N | HIT | 0.473229522 |
| LC | camphor | ADCY1 | DSSYKIVIOFKYAU-UHFFFAOYSA-N | HIT | 0.473229522 |
| LC | camphor | GPER1 | DSSYKIVIOFKYAU-UHFFFAOYSA-N | HIT | 0.473229522 |
| LC | camphor | OXGR1 | DSSYKIVIOFKYAU-UHFFFAOYSA-N | HIT | 0.473229522 |
| LC | camphor | GALR1 | DSSYKIVIOFKYAU-UHFFFAOYSA-N | HIT | 0.473229522 |
| LC | camphor | PNOC | DSSYKIVIOFKYAU-UHFFFAOYSA-N | HIT | 0.473229522 |
| LC | camphor | C3AR1 | DSSYKIVIOFKYAU-UHFFFAOYSA-N | HIT | 0.473229522 |
| LC | camphor | FPR1 | DSSYKIVIOFKYAU-UHFFFAOYSA-N | HIT | 0.473229522 |
| LC | camphor | MTNR1A | DSSYKIVIOFKYAU-UHFFFAOYSA-N | HIT | 0.473229522 |
| LC | camphor | CXCR6 | DSSYKIVIOFKYAU-UHFFFAOYSA-N | HIT | 0.473229522 |
| LC | camphor | S1PR1 | DSSYKIVIOFKYAU-UHFFFAOYSA-N | HIT | 0.473229522 |
| LC | camphor | CXCL10 | DSSYKIVIOFKYAU-UHFFFAOYSA-N | HIT | 0.473229522 |
| LC | camphor | NMUR1 | DSSYKIVIOFKYAU-UHFFFAOYSA-N | HIT | 0.473229522 |
| LC | camphor | CXCL8 | DSSYKIVIOFKYAU-UHFFFAOYSA-N | HIT | 0.473229522 |
| LC | camphor | CXCL11 | DSSYKIVIOFKYAU-UHFFFAOYSA-N | HIT | 0.473229522 |
| LC | camphor | P2RY12 | DSSYKIVIOFKYAU-UHFFFAOYSA-N | HIT | 0.473229522 |
| LC | camphor | HTR1E | DSSYKIVIOFKYAU-UHFFFAOYSA-N | HIT | 0.473229522 |
| LC | camphor | P2RY14 | DSSYKIVIOFKYAU-UHFFFAOYSA-N | HIT | 0.473229522 |
| LC | camphor | ADORA1 | DSSYKIVIOFKYAU-UHFFFAOYSA-N | HIT | 0.473229522 |
| LC | camphor | CCL19 | DSSYKIVIOFKYAU-UHFFFAOYSA-N | HIT | 0.473229522 |
| LC | camphor | ADCY6 | DSSYKIVIOFKYAU-UHFFFAOYSA-N | HIT | 0.473229522 |
| LC | camphor | ADCY4 | DSSYKIVIOFKYAU-UHFFFAOYSA-N | HIT | 0.473229522 |
| LC | camphor | GNAI2 | DSSYKIVIOFKYAU-UHFFFAOYSA-N | HIT | 0.473229522 |
| LC | camphor | HTR1A | DSSYKIVIOFKYAU-UHFFFAOYSA-N | HIT | 0.473229522 |
| LC | camphor | CXCR2 | DSSYKIVIOFKYAU-UHFFFAOYSA-N | HIT | 0.473229522 |
| LC | camphor | CHRM2 | DSSYKIVIOFKYAU-UHFFFAOYSA-N | HIT | 0.473229522 |
| LC | camphor | P2RY13 | DSSYKIVIOFKYAU-UHFFFAOYSA-N | HIT | 0.473229522 |
| LC | camphor | HTR1F | DSSYKIVIOFKYAU-UHFFFAOYSA-N | HIT | 0.473229522 |
| LC | camphor | PENK | DSSYKIVIOFKYAU-UHFFFAOYSA-N | HIT | 0.473229522 |
| LC | camphor | CCR8 | DSSYKIVIOFKYAU-UHFFFAOYSA-N | HIT | 0.473229522 |
| LC | camphor | TAS2R60 | DSSYKIVIOFKYAU-UHFFFAOYSA-N | HIT | 0.473229522 |
| LC | camphor | LPAR5 | DSSYKIVIOFKYAU-UHFFFAOYSA-N | HIT | 0.473229522 |
| LC | camphor | S1PR5 | DSSYKIVIOFKYAU-UHFFFAOYSA-N | HIT | 0.473229522 |
| LC | camphor | RXFP3 | DSSYKIVIOFKYAU-UHFFFAOYSA-N | HIT | 0.473229522 |
| LC | camphor | GALR2 | DSSYKIVIOFKYAU-UHFFFAOYSA-N | HIT | 0.473229522 |
| LC | camphor | NPW | DSSYKIVIOFKYAU-UHFFFAOYSA-N | HIT | 0.473229522 |
| LC | camphor | NPBWR1 | DSSYKIVIOFKYAU-UHFFFAOYSA-N | HIT | 0.473229522 |
| LC | camphor | PMCH | DSSYKIVIOFKYAU-UHFFFAOYSA-N | HIT | 0.473229522 |
| LC | camphor | CCR10 | DSSYKIVIOFKYAU-UHFFFAOYSA-N | HIT | 0.473229522 |
| LC | camphor | NPY2R | DSSYKIVIOFKYAU-UHFFFAOYSA-N | HIT | 0.473229522 |
| LC | camphor | CCR4 | DSSYKIVIOFKYAU-UHFFFAOYSA-N | HIT | 0.473229522 |
| LC | camphor | NPB | DSSYKIVIOFKYAU-UHFFFAOYSA-N | HIT | 0.473229522 |
| LC | camphor | PTGDR2 | DSSYKIVIOFKYAU-UHFFFAOYSA-N | HIT | 0.473229522 |
| LC | camphor | RGS19 | DSSYKIVIOFKYAU-UHFFFAOYSA-N | HIT | 0.473229522 |
| LC | camphor | GNG2 | DSSYKIVIOFKYAU-UHFFFAOYSA-N | HIT | 0.473229522 |
| LC | camphor | OPRL1 | DSSYKIVIOFKYAU-UHFFFAOYSA-N | HIT | 0.473229522 |
| LC | camphor | NPY5R | DSSYKIVIOFKYAU-UHFFFAOYSA-N | HIT | 0.473229522 |
| LC | camphor | CCR6 | DSSYKIVIOFKYAU-UHFFFAOYSA-N | HIT | 0.473229522 |
| LC | camphor | FPR2 | DSSYKIVIOFKYAU-UHFFFAOYSA-N | HIT | 0.473229522 |
| LC | camphor | CYB5A | DSSYKIVIOFKYAU-UHFFFAOYSA-N | HIT | 0.473229522 |
| LC | camphor | FPR3 | DSSYKIVIOFKYAU-UHFFFAOYSA-N | HIT | 0.473229522 |
| LC | camphor | HRH3 | DSSYKIVIOFKYAU-UHFFFAOYSA-N | HIT | 0.473229522 |
| LC | camphor | ADCY2 | DSSYKIVIOFKYAU-UHFFFAOYSA-N | HIT | 0.473229522 |
| LC | camphor | GNAI1 | DSSYKIVIOFKYAU-UHFFFAOYSA-N | HIT | 0.473229522 |
| LC | camphor | GPR18 | DSSYKIVIOFKYAU-UHFFFAOYSA-N | HIT | 0.473229522 |
| LC | camphor | GRM8 | DSSYKIVIOFKYAU-UHFFFAOYSA-N | HIT | 0.473229522 |
| LC | camphor | C5AR1 | DSSYKIVIOFKYAU-UHFFFAOYSA-N | HIT | 0.473229522 |
| LC | camphor | SAA1 | DSSYKIVIOFKYAU-UHFFFAOYSA-N | HIT | 0.473229522 |
| LC | camphor | PTGER3 | DSSYKIVIOFKYAU-UHFFFAOYSA-N | HIT | 0.473229522 |
| LC | camphor | SSTR2 | DSSYKIVIOFKYAU-UHFFFAOYSA-N | HIT | 0.473229522 |
| LC | camphor | CCR9 | DSSYKIVIOFKYAU-UHFFFAOYSA-N | HIT | 0.473229522 |
| LC | camphor | GRM7 | DSSYKIVIOFKYAU-UHFFFAOYSA-N | HIT | 0.473229522 |
| LC | camphor | S1PR3 | DSSYKIVIOFKYAU-UHFFFAOYSA-N | HIT | 0.473229522 |
| LC | camphor | CCL20 | DSSYKIVIOFKYAU-UHFFFAOYSA-N | HIT | 0.473229522 |
| LC | camphor | LPAR1 | DSSYKIVIOFKYAU-UHFFFAOYSA-N | HIT | 0.473229522 |
| LC | camphor | PYY | DSSYKIVIOFKYAU-UHFFFAOYSA-N | HIT | 0.473229522 |
| LC | camphor | CCL28 | DSSYKIVIOFKYAU-UHFFFAOYSA-N | HIT | 0.473229522 |
| LC | camphor | NPY1R | DSSYKIVIOFKYAU-UHFFFAOYSA-N | HIT | 0.473229522 |
| LC | camphor | DRD2 | DSSYKIVIOFKYAU-UHFFFAOYSA-N | HIT | 0.473229522 |
| LC | camphor | CXCL9 | DSSYKIVIOFKYAU-UHFFFAOYSA-N | HIT | 0.473229522 |
| LC | camphor | SUCNR1 | DSSYKIVIOFKYAU-UHFFFAOYSA-N | HIT | 0.473229522 |
| LC | camphor | GRM3 | DSSYKIVIOFKYAU-UHFFFAOYSA-N | HIT | 0.473229522 |
| LC | camphor | RGS7 | DSSYKIVIOFKYAU-UHFFFAOYSA-N | HIT | 0.473229522 |
| LC | camphor | AGT | DSSYKIVIOFKYAU-UHFFFAOYSA-N | HIT | 0.473229522 |
| LC | camphor | RGS1 | DSSYKIVIOFKYAU-UHFFFAOYSA-N | HIT | 0.473229522 |
| LC | camphor | RGS18 | DSSYKIVIOFKYAU-UHFFFAOYSA-N | HIT | 0.473229522 |
| LC | camphor | RXFP4 | DSSYKIVIOFKYAU-UHFFFAOYSA-N | HIT | 0.473229522 |
| LC | camphor | RGS10 | DSSYKIVIOFKYAU-UHFFFAOYSA-N | HIT | 0.473229522 |
| LC | camphor | CNR1 | DSSYKIVIOFKYAU-UHFFFAOYSA-N | HIT | 0.473229522 |
| LC | camphor | TMIGD3 | DSSYKIVIOFKYAU-UHFFFAOYSA-N | HIT | 0.473229522 |
| LC | camphor | NPBWR2 | DSSYKIVIOFKYAU-UHFFFAOYSA-N | HIT | 0.473229522 |
| LC | camphor | GNAI3 | DSSYKIVIOFKYAU-UHFFFAOYSA-N | HIT | 0.473229522 |
| LC | camphor | HTR1B | DSSYKIVIOFKYAU-UHFFFAOYSA-N | HIT | 0.473229522 |
| LC | camphor | LPAR3 | DSSYKIVIOFKYAU-UHFFFAOYSA-N | HIT | 0.473229522 |
| LC | camphor | AGTR2 | DSSYKIVIOFKYAU-UHFFFAOYSA-N | HIT | 0.473229522 |
| LC | camphor | CXCR3 | DSSYKIVIOFKYAU-UHFFFAOYSA-N | HIT | 0.473229522 |
| LC | camphor | GRM4 | DSSYKIVIOFKYAU-UHFFFAOYSA-N | HIT | 0.473229522 |
| LC | camphor | NPY4R2 | DSSYKIVIOFKYAU-UHFFFAOYSA-N | HIT | 0.473229522 |
| LC | camphor | CNR2 | DSSYKIVIOFKYAU-UHFFFAOYSA-N | HIT | 0.473229522 |
| LC | camphor | P2RY4 | DSSYKIVIOFKYAU-UHFFFAOYSA-N | HIT | 0.473229522 |
| LC | camphor | NMS | DSSYKIVIOFKYAU-UHFFFAOYSA-N | HIT | 0.473229522 |
| LC | camphor | OXER1 | DSSYKIVIOFKYAU-UHFFFAOYSA-N | HIT | 0.473229522 |
| LC | camphor | TAS2R1 | DSSYKIVIOFKYAU-UHFFFAOYSA-N | HIT | 0.473229522 |
| LC | camphor | DRD3 | DSSYKIVIOFKYAU-UHFFFAOYSA-N | HIT | 0.473229522 |
| LC | camphor | HCAR2 | DSSYKIVIOFKYAU-UHFFFAOYSA-N | HIT | 0.473229522 |
| LC | camphor | CCL25 | DSSYKIVIOFKYAU-UHFFFAOYSA-N | HIT | 0.473229522 |
| LC | camphor | TAS2R19 | DSSYKIVIOFKYAU-UHFFFAOYSA-N | HIT | 0.473229522 |
| LC | camphor | TAS2R31 | DSSYKIVIOFKYAU-UHFFFAOYSA-N | HIT | 0.473229522 |
| LC | camphor | TAS2R13 | DSSYKIVIOFKYAU-UHFFFAOYSA-N | HIT | 0.473229522 |
| LC | camphor | GPR55 | DSSYKIVIOFKYAU-UHFFFAOYSA-N | HIT | 0.473229522 |
| LC | camphor | GRM2 | DSSYKIVIOFKYAU-UHFFFAOYSA-N | HIT | 0.473229522 |
| LC | camphor | CXCL1 | DSSYKIVIOFKYAU-UHFFFAOYSA-N | HIT | 0.473229522 |
| LC | camphor | CXCL12 | DSSYKIVIOFKYAU-UHFFFAOYSA-N | HIT | 0.473229522 |
| LC | camphor | GNAT3 | DSSYKIVIOFKYAU-UHFFFAOYSA-N | HIT | 0.473229522 |
| LC | camphor | TRPV1 | DSSYKIVIOFKYAU-UHFFFAOYSA-N | STITCH | 0.473229522 |
| LC | camphor | LPAR2 | DSSYKIVIOFKYAU-UHFFFAOYSA-N | HIT | 0.473229522 |
| LC | camphor | ADRA2C | DSSYKIVIOFKYAU-UHFFFAOYSA-N | HIT | 0.473229522 |
| LC | camphor | TAS2R41 | DSSYKIVIOFKYAU-UHFFFAOYSA-N | HIT | 0.473229522 |
| LC | camphor | TAS2R40 | DSSYKIVIOFKYAU-UHFFFAOYSA-N | HIT | 0.473229522 |
| LC | camphor | RGS14 | DSSYKIVIOFKYAU-UHFFFAOYSA-N | HIT | 0.473229522 |
| LC | camphor | CXCR4 | DSSYKIVIOFKYAU-UHFFFAOYSA-N | HIT | 0.473229522 |
| LC | camphor | OPRM1 | DSSYKIVIOFKYAU-UHFFFAOYSA-N | HIT | 0.473229522 |
| LC | camphor | RGS4 | DSSYKIVIOFKYAU-UHFFFAOYSA-N | HIT | 0.473229522 |
| LC | camphor | TAS2R39 | DSSYKIVIOFKYAU-UHFFFAOYSA-N | HIT | 0.473229522 |
| LC | camphor | CHRM4 | DSSYKIVIOFKYAU-UHFFFAOYSA-N | HIT | 0.473229522 |
| LC | camphor | ADCY5 | DSSYKIVIOFKYAU-UHFFFAOYSA-N | HIT | 0.473229522 |
| LC | camphor | TAS2R50 | DSSYKIVIOFKYAU-UHFFFAOYSA-N | HIT | 0.473229522 |
| LC | camphor | CXCL2 | DSSYKIVIOFKYAU-UHFFFAOYSA-N | HIT | 0.473229522 |
| LC | camphor | TAS2R43 | DSSYKIVIOFKYAU-UHFFFAOYSA-N | HIT | 0.473229522 |
| LC | camphor | TAS2R46 | DSSYKIVIOFKYAU-UHFFFAOYSA-N | HIT | 0.473229522 |
| LC | camphor | HCAR3 | DSSYKIVIOFKYAU-UHFFFAOYSA-N | HIT | 0.473229522 |
| LC | camphor | CCR3 | DSSYKIVIOFKYAU-UHFFFAOYSA-N | HIT | 0.473229522 |
| LC | camphor | TAS2R20 | DSSYKIVIOFKYAU-UHFFFAOYSA-N | HIT | 0.473229522 |
| LC | camphor | TAS2R14 | DSSYKIVIOFKYAU-UHFFFAOYSA-N | HIT | 0.473229522 |
| LC | camphor | TAS2R30 | DSSYKIVIOFKYAU-UHFFFAOYSA-N | HIT | 0.473229522 |
| LC | camphor | TAS2R38 | DSSYKIVIOFKYAU-UHFFFAOYSA-N | HIT | 0.473229522 |
| LC | camphor | RGS6 | DSSYKIVIOFKYAU-UHFFFAOYSA-N | HIT | 0.473229522 |
| LC | camphor | TRPV3 | DSSYKIVIOFKYAU-UHFFFAOYSA-N | HIT | 0.473229522 |
| LC | camphor | S1PR2 | DSSYKIVIOFKYAU-UHFFFAOYSA-N | HIT | 0.473229522 |
| LC | naphthalene | CYP1A1 | UFWIBTONFRDIAS-UHFFFAOYSA-N | STITCH | 0.338816693 |
| LC | naphthalene | CYP3A5 | UFWIBTONFRDIAS-UHFFFAOYSA-N | HIT | 0.338816693 |
| LC | naphthalene | RPS6KB1 | UFWIBTONFRDIAS-UHFFFAOYSA-N | HIT | 0.338816693 |
| LC | naphthalene | CYP4F11 | UFWIBTONFRDIAS-UHFFFAOYSA-N | HIT | 0.338816693 |
| LC | naphthalene | ADRA2A | UFWIBTONFRDIAS-UHFFFAOYSA-N | HIT | 0.338816693 |
| LC | naphthalene | CYP2C18 | UFWIBTONFRDIAS-UHFFFAOYSA-N | HIT | 0.338816693 |
| LC | naphthalene | CYP2A7 | UFWIBTONFRDIAS-UHFFFAOYSA-N | HIT | 0.338816693 |
| LC | naphthalene | CYP2S1 | UFWIBTONFRDIAS-UHFFFAOYSA-N | HIT | 0.338816693 |
| LC | naphthalene | CYP4F12 | UFWIBTONFRDIAS-UHFFFAOYSA-N | HIT | 0.338816693 |
| LC | naphthalene | NPY2R | UFWIBTONFRDIAS-UHFFFAOYSA-N | HIT | 0.338816693 |
| LC | naphthalene | CYP2A13 | UFWIBTONFRDIAS-UHFFFAOYSA-N | HIT | 0.338816693 |
| LC | naphthalene | CYP2F1 | UFWIBTONFRDIAS-UHFFFAOYSA-N | HIT | 0.338816693 |
| LC | naphthalene | CYP4Z1 | UFWIBTONFRDIAS-UHFFFAOYSA-N | HIT | 0.338816693 |
| LC | naphthalene | CYP3A7-CYP3A51P | UFWIBTONFRDIAS-UHFFFAOYSA-N | HIT | 0.338816693 |
| LC | naphthalene | NPY5R | UFWIBTONFRDIAS-UHFFFAOYSA-N | HIT | 0.338816693 |
| LC | naphthalene | CYP1A2 | UFWIBTONFRDIAS-UHFFFAOYSA-N | HIT | 0.338816693 |
| LC | naphthalene | CYP2D6 | UFWIBTONFRDIAS-UHFFFAOYSA-N | HIT | 0.338816693 |
| LC | naphthalene | NPY1R | UFWIBTONFRDIAS-UHFFFAOYSA-N | HIT | 0.338816693 |
| LC | naphthalene | CYP2J2 | UFWIBTONFRDIAS-UHFFFAOYSA-N | HIT | 0.338816693 |
| LC | naphthalene | CYP2C8 | UFWIBTONFRDIAS-UHFFFAOYSA-N | HIT | 0.338816693 |
| LC | naphthalene | CYP4X1 | UFWIBTONFRDIAS-UHFFFAOYSA-N | HIT | 0.338816693 |
| LC | naphthalene | CYP4B1 | UFWIBTONFRDIAS-UHFFFAOYSA-N | HIT | 0.338816693 |
| LC | naphthalene | ADRA2C | UFWIBTONFRDIAS-UHFFFAOYSA-N | HIT | 0.338816693 |
| LC | naphthalene | EIF4E | UFWIBTONFRDIAS-UHFFFAOYSA-N | HIT | 0.338816693 |
| LC | octanol | ADH1A | KBPLFHHGFOOTCA-UHFFFAOYSA-N | HIT | 0.343408735 |
| LC | octanol | ADH4 | KBPLFHHGFOOTCA-UHFFFAOYSA-N | HIT | 0.343408735 |
| LC | octanol | ADH5 | KBPLFHHGFOOTCA-UHFFFAOYSA-N | HIT | 0.343408735 |
| LC | octanol | ADH1B | KBPLFHHGFOOTCA-UHFFFAOYSA-N | HIT | 0.343408735 |
| LC | octanol | CYP4A11 | KBPLFHHGFOOTCA-UHFFFAOYSA-N | HIT | 0.343408735 |
| LC | octanol | CYP4A22 | KBPLFHHGFOOTCA-UHFFFAOYSA-N | HIT | 0.343408735 |
| LC | octanol | ADH6 | KBPLFHHGFOOTCA-UHFFFAOYSA-N | HIT | 0.343408735 |
| LC | octanol | ADH7 | KBPLFHHGFOOTCA-UHFFFAOYSA-N | HIT | 0.343408735 |
| LC | PHB | CA2 | FJKROLUGYXJWQN-UHFFFAOYSA-N | TCMSP | 0.688480497 |
| LC | PHB | COQ2 | FJKROLUGYXJWQN-UHFFFAOYSA-N | TCMSP | 0.688480497 |
| LC | PHB | COQ6 | FJKROLUGYXJWQN-UHFFFAOYSA-N | TCMSP | 0.688480497 |
| LC | PHB | PDSS2 | FJKROLUGYXJWQN-UHFFFAOYSA-N | TCMSP | 0.688480497 |
| LC | PHB | PDSS1 | FJKROLUGYXJWQN-UHFFFAOYSA-N | TCMSP | 0.688480497 |
| LC | Nonanal | TFPI2 | GYHFUZHODSMOHU-UHFFFAOYSA-N | TCMSP | 0.467045174 |
| LC | Nonanal | RDH12 | GYHFUZHODSMOHU-UHFFFAOYSA-N | TCMSP | 0.467045174 |
| LC | EIC | CETP | OYHQOLUKZRVURQ-HZJYTTRNSA-N | TCMSP | 0.378598977 |
| LC | EIC | PLA2G3 | OYHQOLUKZRVURQ-HZJYTTRNSA-N | TCMSP | 0.378598977 |
| LC | EIC | PCK2 | OYHQOLUKZRVURQ-HZJYTTRNSA-N | TCMSP | 0.378598977 |
| LC | EIC | TRIB3 | OYHQOLUKZRVURQ-HZJYTTRNSA-N | TCMSP | 0.378598977 |
| LC | EIC | TBL1X | OYHQOLUKZRVURQ-HZJYTTRNSA-N | TCMSP | 0.378598977 |
| LC | EIC | FDFT1 | OYHQOLUKZRVURQ-HZJYTTRNSA-N | TCMSP | 0.378598977 |
| LC | EIC | SULT2A1 | OYHQOLUKZRVURQ-HZJYTTRNSA-N | TCMSP | 0.378598977 |
| LC | EIC | PON1 | OYHQOLUKZRVURQ-HZJYTTRNSA-N | TCMSP | 0.378598977 |
| LC | EIC | CYP3A5 | OYHQOLUKZRVURQ-HZJYTTRNSA-N | TCMSP | 0.378598977 |
| LC | EIC | SERPINE1 | OYHQOLUKZRVURQ-HZJYTTRNSA-N | TCMSP | 0.378598977 |
| LC | EIC | NRF1 | OYHQOLUKZRVURQ-HZJYTTRNSA-N | TCMSP | 0.378598977 |
| LC | EIC | MED31 | OYHQOLUKZRVURQ-HZJYTTRNSA-N | TCMSP | 0.378598977 |
| LC | EIC | APOA5 | OYHQOLUKZRVURQ-HZJYTTRNSA-N | TCMSP | 0.378598977 |
| LC | EIC | APOC3 | OYHQOLUKZRVURQ-HZJYTTRNSA-N | TCMSP | 0.378598977 |
| LC | EIC | RBP1 | OYHQOLUKZRVURQ-HZJYTTRNSA-N | TCMSP | 0.378598977 |
| LC | EIC | APOB | OYHQOLUKZRVURQ-HZJYTTRNSA-N | TCMSP | 0.378598977 |
| LC | EIC | APOA1 | OYHQOLUKZRVURQ-HZJYTTRNSA-N | TCMSP | 0.378598977 |
| LC | EIC | ACOT2 | OYHQOLUKZRVURQ-HZJYTTRNSA-N | TCMSP | 0.378598977 |
| LC | EIC | NFYB | OYHQOLUKZRVURQ-HZJYTTRNSA-N | TCMSP | 0.378598977 |
| LC | EIC | CAT | OYHQOLUKZRVURQ-HZJYTTRNSA-N | TCMSP | 0.378598977 |
| LC | EIC | LRP1 | OYHQOLUKZRVURQ-HZJYTTRNSA-N | TCMSP | 0.378598977 |
| LC | EIC | PLA2G12A | OYHQOLUKZRVURQ-HZJYTTRNSA-N | TCMSP | 0.378598977 |
| LC | EIC | FFAR1 | OYHQOLUKZRVURQ-HZJYTTRNSA-N | TCMSP | 0.378598977 |
| LC | EIC | CYP4F11 | OYHQOLUKZRVURQ-HZJYTTRNSA-N | TCMSP | 0.378598977 |
| LC | EIC | MED17 | OYHQOLUKZRVURQ-HZJYTTRNSA-N | TCMSP | 0.378598977 |
| LC | EIC | APOE | OYHQOLUKZRVURQ-HZJYTTRNSA-N | TCMSP | 0.378598977 |
| LC | EIC | SLC27A1 | OYHQOLUKZRVURQ-HZJYTTRNSA-N | TCMSP | 0.378598977 |
| LC | EIC | CYP2E1 | OYHQOLUKZRVURQ-HZJYTTRNSA-N | TCMSP | 0.378598977 |
| LC | EIC | SDC1 | OYHQOLUKZRVURQ-HZJYTTRNSA-N | TCMSP | 0.378598977 |
| LC | EIC | MED10 | OYHQOLUKZRVURQ-HZJYTTRNSA-N | TCMSP | 0.378598977 |
| LC | EIC | FABP4 | OYHQOLUKZRVURQ-HZJYTTRNSA-N | TCMSP | 0.378598977 |
| LC | EIC | MED6 | OYHQOLUKZRVURQ-HZJYTTRNSA-N | TCMSP | 0.378598977 |
| LC | EIC | MED4 | OYHQOLUKZRVURQ-HZJYTTRNSA-N | TCMSP | 0.378598977 |
| LC | EIC | IL6 | OYHQOLUKZRVURQ-HZJYTTRNSA-N | TCMSP | 0.378598977 |
| LC | EIC | ACSBG1 | OYHQOLUKZRVURQ-HZJYTTRNSA-N | TCMSP | 0.378598977 |
| LC | EIC | BAAT | OYHQOLUKZRVURQ-HZJYTTRNSA-N | TCMSP | 0.378598977 |
| LC | EIC | TGS1 | OYHQOLUKZRVURQ-HZJYTTRNSA-N | TCMSP | 0.378598977 |
| LC | EIC | CYP2C9 | OYHQOLUKZRVURQ-HZJYTTRNSA-N | TCMSP | 0.378598977 |
| LC | EIC | RORA | OYHQOLUKZRVURQ-HZJYTTRNSA-N | TCMSP | 0.378598977 |
| LC | EIC | SMARCD3 | OYHQOLUKZRVURQ-HZJYTTRNSA-N | TCMSP | 0.378598977 |
| LC | EIC | RPE65 | OYHQOLUKZRVURQ-HZJYTTRNSA-N | TCMSP | 0.378598977 |
| LC | EIC | CREBBP | OYHQOLUKZRVURQ-HZJYTTRNSA-N | TCMSP | 0.378598977 |
| LC | EIC | PPARA | OYHQOLUKZRVURQ-HZJYTTRNSA-N | TCMSP | 0.378598977 |
| LC | EIC | MED15 | OYHQOLUKZRVURQ-HZJYTTRNSA-N | TCMSP | 0.378598977 |
| LC | EIC | EP300 | OYHQOLUKZRVURQ-HZJYTTRNSA-N | TCMSP | 0.378598977 |
| LC | EIC | TYR | OYHQOLUKZRVURQ-HZJYTTRNSA-N | TCMSP | 0.378598977 |
| LC | EIC | MED26 | OYHQOLUKZRVURQ-HZJYTTRNSA-N | TCMSP | 0.378598977 |
| LC | EIC | LRP2 | OYHQOLUKZRVURQ-HZJYTTRNSA-N | TCMSP | 0.378598977 |
| LC | EIC | GPC1 | OYHQOLUKZRVURQ-HZJYTTRNSA-N | TCMSP | 0.378598977 |
| LC | EIC | PPARGC1A | OYHQOLUKZRVURQ-HZJYTTRNSA-N | TCMSP | 0.378598977 |
| LC | EIC | MED20 | OYHQOLUKZRVURQ-HZJYTTRNSA-N | TCMSP | 0.378598977 |
| LC | EIC | CPT1A | OYHQOLUKZRVURQ-HZJYTTRNSA-N | TCMSP | 0.378598977 |
| LC | EIC | ABCB4 | OYHQOLUKZRVURQ-HZJYTTRNSA-N | TCMSP | 0.378598977 |
| LC | EIC | GLIPR1 | OYHQOLUKZRVURQ-HZJYTTRNSA-N | TCMSP | 0.378598977 |
| LC | EIC | SLC27A2 | OYHQOLUKZRVURQ-HZJYTTRNSA-N | TCMSP | 0.378598977 |
| LC | EIC | MED9 | OYHQOLUKZRVURQ-HZJYTTRNSA-N | TCMSP | 0.378598977 |
| LC | EIC | NCOR1 | OYHQOLUKZRVURQ-HZJYTTRNSA-N | TCMSP | 0.378598977 |
| LC | EIC | PLIN2 | OYHQOLUKZRVURQ-HZJYTTRNSA-N | TCMSP | 0.378598977 |
| LC | EIC | FADS2 | OYHQOLUKZRVURQ-HZJYTTRNSA-N | TCMSP | 0.378598977 |
| LC | EIC | ACSL1 | OYHQOLUKZRVURQ-HZJYTTRNSA-N | TCMSP | 0.378598977 |
| LC | EIC | MED13L | OYHQOLUKZRVURQ-HZJYTTRNSA-N | TCMSP | 0.378598977 |
| LC | EIC | YAP1 | OYHQOLUKZRVURQ-HZJYTTRNSA-N | TCMSP | 0.378598977 |
| LC | EIC | MED21 | OYHQOLUKZRVURQ-HZJYTTRNSA-N | TCMSP | 0.378598977 |
| LC | EIC | CYP2C18 | OYHQOLUKZRVURQ-HZJYTTRNSA-N | TCMSP | 0.378598977 |
| LC | EIC | PPARG | OYHQOLUKZRVURQ-HZJYTTRNSA-N | TCMSP | 0.378598977 |
| LC | EIC | HMGCR | OYHQOLUKZRVURQ-HZJYTTRNSA-N | TCMSP | 0.378598977 |
| LC | EIC | ACSM3 | OYHQOLUKZRVURQ-HZJYTTRNSA-N | TCMSP | 0.378598977 |
| LC | EIC | PLA2G4D | OYHQOLUKZRVURQ-HZJYTTRNSA-N | TCMSP | 0.378598977 |
| LC | EIC | MED8 | OYHQOLUKZRVURQ-HZJYTTRNSA-N | TCMSP | 0.378598977 |
| LC | EIC | MED27 | OYHQOLUKZRVURQ-HZJYTTRNSA-N | TCMSP | 0.378598977 |
| LC | EIC | GPC2 | OYHQOLUKZRVURQ-HZJYTTRNSA-N | TCMSP | 0.378598977 |
| LC | EIC | ALOX15 | OYHQOLUKZRVURQ-HZJYTTRNSA-N | TCMSP | 0.378598977 |
| LC | EIC | MED11 | OYHQOLUKZRVURQ-HZJYTTRNSA-N | TCMSP | 0.378598977 |
| LC | EIC | VCAM1 | OYHQOLUKZRVURQ-HZJYTTRNSA-N | TCMSP | 0.378598977 |
| LC | EIC | FABP1 | OYHQOLUKZRVURQ-HZJYTTRNSA-N | TCMSP | 0.378598977 |
| LC | EIC | TNFRSF21 | OYHQOLUKZRVURQ-HZJYTTRNSA-N | TCMSP | 0.378598977 |
| LC | EIC | ACSL6 | OYHQOLUKZRVURQ-HZJYTTRNSA-N | TCMSP | 0.378598977 |
| LC | EIC | FABP5 | OYHQOLUKZRVURQ-HZJYTTRNSA-N | TCMSP | 0.378598977 |
| LC | EIC | MED30 | OYHQOLUKZRVURQ-HZJYTTRNSA-N | TCMSP | 0.378598977 |
| LC | EIC | PEX11A | OYHQOLUKZRVURQ-HZJYTTRNSA-N | TCMSP | 0.378598977 |
| LC | EIC | MED1 | OYHQOLUKZRVURQ-HZJYTTRNSA-N | TCMSP | 0.378598977 |
| LC | EIC | CYP2A7 | OYHQOLUKZRVURQ-HZJYTTRNSA-N | TCMSP | 0.378598977 |
| LC | EIC | ANGPTL4 | OYHQOLUKZRVURQ-HZJYTTRNSA-N | TCMSP | 0.378598977 |
| LC | EIC | CYP7A1 | OYHQOLUKZRVURQ-HZJYTTRNSA-N | TCMSP | 0.378598977 |
| LC | EIC | ACSM1 | OYHQOLUKZRVURQ-HZJYTTRNSA-N | TCMSP | 0.378598977 |
| LC | EIC | HDAC3 | OYHQOLUKZRVURQ-HZJYTTRNSA-N | TCMSP | 0.378598977 |
| LC | EIC | RGL1 | OYHQOLUKZRVURQ-HZJYTTRNSA-N | TCMSP | 0.378598977 |
| LC | EIC | LRP8 | OYHQOLUKZRVURQ-HZJYTTRNSA-N | TCMSP | 0.378598977 |
| LC | EIC | UGT1A1 | OYHQOLUKZRVURQ-HZJYTTRNSA-N | TCMSP | 0.378598977 |
| LC | EIC | FOS | OYHQOLUKZRVURQ-HZJYTTRNSA-N | TCMSP | 0.378598977 |
| LC | EIC | SDC2 | OYHQOLUKZRVURQ-HZJYTTRNSA-N | TCMSP | 0.378598977 |
| LC | EIC | FADS6 | OYHQOLUKZRVURQ-HZJYTTRNSA-N | TCMSP | 0.378598977 |
| LC | EIC | CYP2S1 | OYHQOLUKZRVURQ-HZJYTTRNSA-N | TCMSP | 0.378598977 |
| LC | EIC | CD36 | OYHQOLUKZRVURQ-HZJYTTRNSA-N | TCMSP | 0.378598977 |
| LC | EIC | CLOCK | OYHQOLUKZRVURQ-HZJYTTRNSA-N | TCMSP | 0.378598977 |
| LC | EIC | OLR1 | OYHQOLUKZRVURQ-HZJYTTRNSA-N | TCMSP | 0.378598977 |
| LC | EIC | ALAS1 | OYHQOLUKZRVURQ-HZJYTTRNSA-N | TCMSP | 0.378598977 |
| LC | EIC | TEAD2 | OYHQOLUKZRVURQ-HZJYTTRNSA-N | TCMSP | 0.378598977 |
| LC | EIC | CYP4A11 | OYHQOLUKZRVURQ-HZJYTTRNSA-N | TCMSP | 0.378598977 |
| LC | EIC | ACOT1 | OYHQOLUKZRVURQ-HZJYTTRNSA-N | TCMSP | 0.378598977 |
| LC | EIC | PLA2G1B | OYHQOLUKZRVURQ-HZJYTTRNSA-N | TCMSP | 0.378598977 |
| LC | EIC | PPARGC1B | OYHQOLUKZRVURQ-HZJYTTRNSA-N | TCMSP | 0.378598977 |
| LC | EIC | LEP | OYHQOLUKZRVURQ-HZJYTTRNSA-N | TCMSP | 0.378598977 |
| LC | EIC | MED29 | OYHQOLUKZRVURQ-HZJYTTRNSA-N | TCMSP | 0.378598977 |
| LC | EIC | GLTP | OYHQOLUKZRVURQ-HZJYTTRNSA-N | TCMSP | 0.378598977 |
| LC | EIC | PCK1 | OYHQOLUKZRVURQ-HZJYTTRNSA-N | TCMSP | 0.378598977 |
| LC | EIC | PLA2G16 | OYHQOLUKZRVURQ-HZJYTTRNSA-N | TCMSP | 0.378598977 |
| LC | EIC | NCOA1 | OYHQOLUKZRVURQ-HZJYTTRNSA-N | TCMSP | 0.378598977 |
| LC | EIC | CYP4F12 | OYHQOLUKZRVURQ-HZJYTTRNSA-N | TCMSP | 0.378598977 |
| LC | EIC | FADS1 | OYHQOLUKZRVURQ-HZJYTTRNSA-N | TCMSP | 0.378598977 |
| LC | EIC | HMGCS1 | OYHQOLUKZRVURQ-HZJYTTRNSA-N | TCMSP | 0.378598977 |
| LC | EIC | FHL2 | OYHQOLUKZRVURQ-HZJYTTRNSA-N | TCMSP | 0.378598977 |
| LC | EIC | ACOT4 | OYHQOLUKZRVURQ-HZJYTTRNSA-N | TCMSP | 0.378598977 |
| LC | EIC | MED14 | OYHQOLUKZRVURQ-HZJYTTRNSA-N | TCMSP | 0.378598977 |
| LC | EIC | GRHL1 | OYHQOLUKZRVURQ-HZJYTTRNSA-N | TCMSP | 0.378598977 |
| LC | EIC | MED16 | OYHQOLUKZRVURQ-HZJYTTRNSA-N | TCMSP | 0.378598977 |
| LC | EIC | CARM1 | OYHQOLUKZRVURQ-HZJYTTRNSA-N | TCMSP | 0.378598977 |
| LC | EIC | MED25 | OYHQOLUKZRVURQ-HZJYTTRNSA-N | TCMSP | 0.378598977 |
| LC | EIC | TIAM2 | OYHQOLUKZRVURQ-HZJYTTRNSA-N | TCMSP | 0.378598977 |
| LC | EIC | ACSM5 | OYHQOLUKZRVURQ-HZJYTTRNSA-N | TCMSP | 0.378598977 |
| LC | EIC | SP1 | OYHQOLUKZRVURQ-HZJYTTRNSA-N | TCMSP | 0.378598977 |
| LC | EIC | PLB1 | OYHQOLUKZRVURQ-HZJYTTRNSA-N | TCMSP | 0.378598977 |
| LC | EIC | SELE | OYHQOLUKZRVURQ-HZJYTTRNSA-N | TCMSP | 0.378598977 |
| LC | EIC | CYP2A13 | OYHQOLUKZRVURQ-HZJYTTRNSA-N | TCMSP | 0.378598977 |
| LC | EIC | PLA2G6 | OYHQOLUKZRVURQ-HZJYTTRNSA-N | TCMSP | 0.378598977 |
| LC | EIC | CYP2F1 | OYHQOLUKZRVURQ-HZJYTTRNSA-N | TCMSP | 0.378598977 |
| LC | EIC | CYP4Z1 | OYHQOLUKZRVURQ-HZJYTTRNSA-N | TCMSP | 0.378598977 |
| LC | EIC | LRAT | OYHQOLUKZRVURQ-HZJYTTRNSA-N | TCMSP | 0.378598977 |
| LC | EIC | MED19 | OYHQOLUKZRVURQ-HZJYTTRNSA-N | TCMSP | 0.378598977 |
| LC | EIC | CYP3A7-CYP3A51P | OYHQOLUKZRVURQ-HZJYTTRNSA-N | TCMSP | 0.378598977 |
| LC | EIC | CYP3A4 | OYHQOLUKZRVURQ-HZJYTTRNSA-N | TCMSP | 0.378598977 |
| LC | EIC | HIF1A | OYHQOLUKZRVURQ-HZJYTTRNSA-N | TCMSP | 0.378598977 |
| LC | EIC | NPAS2 | OYHQOLUKZRVURQ-HZJYTTRNSA-N | TCMSP | 0.378598977 |
| LC | EIC | ACSL4 | OYHQOLUKZRVURQ-HZJYTTRNSA-N | TCMSP | 0.378598977 |
| LC | EIC | ACSM6 | OYHQOLUKZRVURQ-HZJYTTRNSA-N | TCMSP | 0.378598977 |
| LC | EIC | CYP1A2 | OYHQOLUKZRVURQ-HZJYTTRNSA-N | TCMSP | 0.378598977 |
| LC | EIC | MED22 | OYHQOLUKZRVURQ-HZJYTTRNSA-N | TCMSP | 0.378598977 |
| LC | EIC | SDC3 | OYHQOLUKZRVURQ-HZJYTTRNSA-N | TCMSP | 0.378598977 |
| LC | EIC | NFYA | OYHQOLUKZRVURQ-HZJYTTRNSA-N | TCMSP | 0.378598977 |
| LC | EIC | TEAD3 | OYHQOLUKZRVURQ-HZJYTTRNSA-N | TCMSP | 0.378598977 |
| LC | EIC | UGT1A9 | OYHQOLUKZRVURQ-HZJYTTRNSA-N | TCMSP | 0.378598977 |
| LC | EIC | ACSL5 | OYHQOLUKZRVURQ-HZJYTTRNSA-N | TCMSP | 0.378598977 |
| LC | EIC | ACSL3 | OYHQOLUKZRVURQ-HZJYTTRNSA-N | TCMSP | 0.378598977 |
| LC | EIC | NCOA6 | OYHQOLUKZRVURQ-HZJYTTRNSA-N | TCMSP | 0.378598977 |
| LC | EIC | MAPK8 | OYHQOLUKZRVURQ-HZJYTTRNSA-N | TCMSP | 0.378598977 |
| LC | EIC | SIN3A | OYHQOLUKZRVURQ-HZJYTTRNSA-N | TCMSP | 0.378598977 |
| LC | EIC | MME | OYHQOLUKZRVURQ-HZJYTTRNSA-N | TCMSP | 0.378598977 |
| LC | EIC | CYP2D6 | OYHQOLUKZRVURQ-HZJYTTRNSA-N | TCMSP | 0.378598977 |
| LC | EIC | WWTR1 | OYHQOLUKZRVURQ-HZJYTTRNSA-N | TCMSP | 0.378598977 |
| LC | EIC | SREBF2 | OYHQOLUKZRVURQ-HZJYTTRNSA-N | TCMSP | 0.378598977 |
| LC | EIC | AGT | OYHQOLUKZRVURQ-HZJYTTRNSA-N | TCMSP | 0.378598977 |
| LC | EIC | G0S2 | OYHQOLUKZRVURQ-HZJYTTRNSA-N | TCMSP | 0.378598977 |
| LC | EIC | PLA2G4A | OYHQOLUKZRVURQ-HZJYTTRNSA-N | TCMSP | 0.378598977 |
| LC | EIC | CTGF | OYHQOLUKZRVURQ-HZJYTTRNSA-N | TCMSP | 0.378598977 |
| LC | EIC | APOA2 | OYHQOLUKZRVURQ-HZJYTTRNSA-N | TCMSP | 0.378598977 |
| LC | EIC | MED23 | OYHQOLUKZRVURQ-HZJYTTRNSA-N | TCMSP | 0.378598977 |
| LC | EIC | CDK19 | OYHQOLUKZRVURQ-HZJYTTRNSA-N | TCMSP | 0.378598977 |
| LC | EIC | HMGCS2 | OYHQOLUKZRVURQ-HZJYTTRNSA-N | TCMSP | 0.378598977 |
| LC | EIC | ME1 | OYHQOLUKZRVURQ-HZJYTTRNSA-N | TCMSP | 0.378598977 |
| LC | EIC | GPC4 | OYHQOLUKZRVURQ-HZJYTTRNSA-N | TCMSP | 0.378598977 |
| LC | EIC | CYP2J2 | OYHQOLUKZRVURQ-HZJYTTRNSA-N | TCMSP | 0.378598977 |
| LC | EIC | CYP2C8 | OYHQOLUKZRVURQ-HZJYTTRNSA-N | TCMSP | 0.378598977 |
| LC | EIC | CYP2C19 | OYHQOLUKZRVURQ-HZJYTTRNSA-N | TCMSP | 0.378598977 |
| LC | EIC | CPT2 | OYHQOLUKZRVURQ-HZJYTTRNSA-N | TCMSP | 0.378598977 |
| LC | EIC | ANKRD1 | OYHQOLUKZRVURQ-HZJYTTRNSA-N | TCMSP | 0.378598977 |
| LC | EIC | CYP4X1 | OYHQOLUKZRVURQ-HZJYTTRNSA-N | TCMSP | 0.378598977 |
| LC | EIC | CYP4B1 | OYHQOLUKZRVURQ-HZJYTTRNSA-N | TCMSP | 0.378598977 |
| LC | EIC | NCOA3 | OYHQOLUKZRVURQ-HZJYTTRNSA-N | TCMSP | 0.378598977 |
| LC | EIC | SDC4 | OYHQOLUKZRVURQ-HZJYTTRNSA-N | TCMSP | 0.378598977 |
| LC | EIC | PLA2G12B | OYHQOLUKZRVURQ-HZJYTTRNSA-N | TCMSP | 0.378598977 |
| LC | EIC | MED18 | OYHQOLUKZRVURQ-HZJYTTRNSA-N | TCMSP | 0.378598977 |
| LC | EIC | MED12 | OYHQOLUKZRVURQ-HZJYTTRNSA-N | TCMSP | 0.378598977 |
| LC | EIC | ALOX5 | OYHQOLUKZRVURQ-HZJYTTRNSA-N | TCMSP | 0.378598977 |
| LC | EIC | HSPG2 | OYHQOLUKZRVURQ-HZJYTTRNSA-N | TCMSP | 0.378598977 |
| LC | EIC | ABCA1 | OYHQOLUKZRVURQ-HZJYTTRNSA-N | TCMSP | 0.378598977 |
| LC | EIC | PLA2G2F | OYHQOLUKZRVURQ-HZJYTTRNSA-N | TCMSP | 0.378598977 |
| LC | EIC | PLA2G5 | OYHQOLUKZRVURQ-HZJYTTRNSA-N | TCMSP | 0.378598977 |
| LC | EIC | PLA2G2A | OYHQOLUKZRVURQ-HZJYTTRNSA-N | TCMSP | 0.378598977 |
| LC | EIC | PLA2G2E | OYHQOLUKZRVURQ-HZJYTTRNSA-N | TCMSP | 0.378598977 |
| LC | EIC | GPC6 | OYHQOLUKZRVURQ-HZJYTTRNSA-N | TCMSP | 0.378598977 |
| LC | EIC | GPC5 | OYHQOLUKZRVURQ-HZJYTTRNSA-N | TCMSP | 0.378598977 |
| LC | EIC | ACOT7 | OYHQOLUKZRVURQ-HZJYTTRNSA-N | TCMSP | 0.378598977 |
| LC | EIC | AGRN | OYHQOLUKZRVURQ-HZJYTTRNSA-N | TCMSP | 0.378598977 |
| LC | EIC | CYP1A1 | OYHQOLUKZRVURQ-HZJYTTRNSA-N | TCMSP | 0.378598977 |
| LC | EIC | ALOX15B | OYHQOLUKZRVURQ-HZJYTTRNSA-N | TCMSP | 0.378598977 |
| LC | EIC | CDK8 | OYHQOLUKZRVURQ-HZJYTTRNSA-N | TCMSP | 0.378598977 |
| LC | EIC | ARNTL | OYHQOLUKZRVURQ-HZJYTTRNSA-N | TCMSP | 0.378598977 |
| LC | EIC | MED24 | OYHQOLUKZRVURQ-HZJYTTRNSA-N | TCMSP | 0.378598977 |
| LC | EIC | GPC3 | OYHQOLUKZRVURQ-HZJYTTRNSA-N | TCMSP | 0.378598977 |
| LC | EIC | MED13 | OYHQOLUKZRVURQ-HZJYTTRNSA-N | TCMSP | 0.378598977 |
| LC | EIC | ACSM4 | OYHQOLUKZRVURQ-HZJYTTRNSA-N | TCMSP | 0.378598977 |
| LC | EIC | PLA2G4E | OYHQOLUKZRVURQ-HZJYTTRNSA-N | TCMSP | 0.378598977 |
| LC | EIC | NCOR2 | OYHQOLUKZRVURQ-HZJYTTRNSA-N | TCMSP | 0.378598977 |
| LC | EIC | MYO7A | OYHQOLUKZRVURQ-HZJYTTRNSA-N | TCMSP | 0.378598977 |
| LC | EIC | PLA2G10 | OYHQOLUKZRVURQ-HZJYTTRNSA-N | TCMSP | 0.378598977 |
| LC | EIC | PLA2G4B | OYHQOLUKZRVURQ-HZJYTTRNSA-N | TCMSP | 0.378598977 |
| LC | EIC | NFYC | OYHQOLUKZRVURQ-HZJYTTRNSA-N | TCMSP | 0.378598977 |
| LC | EIC | TNF | OYHQOLUKZRVURQ-HZJYTTRNSA-N | TCMSP | 0.378598977 |
| LC | EIC | NCOA2 | OYHQOLUKZRVURQ-HZJYTTRNSA-N | TCMSP | 0.378598977 |
| LC | EIC | TBL1XR1 | OYHQOLUKZRVURQ-HZJYTTRNSA-N | TCMSP | 0.378598977 |
| LC | EIC | ACADM | OYHQOLUKZRVURQ-HZJYTTRNSA-N | TCMSP | 0.378598977 |
| LC | EIC | HELZ2 | OYHQOLUKZRVURQ-HZJYTTRNSA-N | TCMSP | 0.378598977 |
| LC | EIC | RXRA | OYHQOLUKZRVURQ-HZJYTTRNSA-N | TCMSP | 0.378598977 |
| LC | EIC | CCNC | OYHQOLUKZRVURQ-HZJYTTRNSA-N | TCMSP | 0.378598977 |
| LC | EIC | TXNRD1 | OYHQOLUKZRVURQ-HZJYTTRNSA-N | TCMSP | 0.378598977 |
| LC | EIC | LDLR | OYHQOLUKZRVURQ-HZJYTTRNSA-N | TCMSP | 0.378598977 |
| LC | EIC | CHD9 | OYHQOLUKZRVURQ-HZJYTTRNSA-N | TCMSP | 0.378598977 |
| LC | WLN: Q1R | CES2 | WVDDGKGOMKODPV-UHFFFAOYSA-N | TCMSP | 0.526653005 |
| LC | WLN: Q1R | CES1 | WVDDGKGOMKODPV-UHFFFAOYSA-N | TCMSP | 0.526653005 |
| LC | WLN: Q1R | CES5A | WVDDGKGOMKODPV-UHFFFAOYSA-N | TCMSP | 0.526653005 |
| LC | WLN: Q1R | CES4A | WVDDGKGOMKODPV-UHFFFAOYSA-N | TCMSP | 0.526653005 |
| LC | Furol | HBA1 | HYBBIBNJHNGZAN-UHFFFAOYSA-N | TCMSP | 0.570410017 |
| LC | Furol | TBXT | HYBBIBNJHNGZAN-UHFFFAOYSA-N | TCMSP | 0.570410017 |
| LC | Furol | HBB | HYBBIBNJHNGZAN-UHFFFAOYSA-N | TCMSP | 0.570410017 |
| LC | Crysophanol | NLRP3 | LQGUBLBATBMXHT-UHFFFAOYSA-N | TCMSP | 0.796854882 |
| LC | uracil | DHODH | ISAKRJDGNUQOIC-UHFFFAOYSA-N | TCMSP | 0.479192415 |
| LC | uracil | PUS3 | ISAKRJDGNUQOIC-UHFFFAOYSA-N | TCMSP | 0.479192415 |
| LC | uracil | UNG | ISAKRJDGNUQOIC-UHFFFAOYSA-N | TCMSP | 0.479192415 |
| LC | uracil | MBD4 | ISAKRJDGNUQOIC-UHFFFAOYSA-N | TCMSP | 0.479192415 |
| LC | uracil | TYMP | ISAKRJDGNUQOIC-UHFFFAOYSA-N | TCMSP | 0.479192415 |
| LC | uracil | GPR17 | ISAKRJDGNUQOIC-UHFFFAOYSA-N | TCMSP | 0.479192415 |
| LC | uracil | DPYS | ISAKRJDGNUQOIC-UHFFFAOYSA-N | TCMSP | 0.479192415 |
| LC | uracil | CCNO | ISAKRJDGNUQOIC-UHFFFAOYSA-N | TCMSP | 0.479192415 |
| LC | uracil | TRUB1 | ISAKRJDGNUQOIC-UHFFFAOYSA-N | TCMSP | 0.479192415 |
| LC | uracil | JUP | ISAKRJDGNUQOIC-UHFFFAOYSA-N | TCMSP | 0.479192415 |
| LC | uracil | TYMS | ISAKRJDGNUQOIC-UHFFFAOYSA-N | TCMSP | 0.479192415 |
| LC | uracil | SMUG1 | ISAKRJDGNUQOIC-UHFFFAOYSA-N | TCMSP | 0.479192415 |
| LC | uracil | CTNNB1 | ISAKRJDGNUQOIC-UHFFFAOYSA-N | TCMSP | 0.479192415 |
| LC | uracil | UCKL1 | ISAKRJDGNUQOIC-UHFFFAOYSA-N | TCMSP | 0.479192415 |
| LC | uracil | PUS7 | ISAKRJDGNUQOIC-UHFFFAOYSA-N | TCMSP | 0.479192415 |
| LC | uracil | SLC29A2 | ISAKRJDGNUQOIC-UHFFFAOYSA-N | TCMSP | 0.479192415 |
| LC | uracil | PNP | ISAKRJDGNUQOIC-UHFFFAOYSA-N | TCMSP | 0.479192415 |
| LC | uracil | DPYD | ISAKRJDGNUQOIC-UHFFFAOYSA-N | TCMSP | 0.479192415 |
| LC | uracil | UPRT | ISAKRJDGNUQOIC-UHFFFAOYSA-N | TCMSP | 0.479192415 |
| LC | uracil | TDG | ISAKRJDGNUQOIC-UHFFFAOYSA-N | TCMSP | 0.479192415 |
| LC | uracil | POLD1 | ISAKRJDGNUQOIC-UHFFFAOYSA-N | TCMSP | 0.479192415 |
| LC | EUG | IL6 | YOMSJEATGXXYPX-UHFFFAOYSA-N | TCMSP | 0.743229214 |
| LC | ADO | CX3CL1 | OIRDTQYFTABQOQ-KQYNXXCUSA-N | TCMSP | 0.31947565 |
| LC | ADO | HEBP1 | OIRDTQYFTABQOQ-KQYNXXCUSA-N | TCMSP | 0.31947565 |
| LC | ADO | SCTR | OIRDTQYFTABQOQ-KQYNXXCUSA-N | TCMSP | 0.31947565 |
| LC | ADO | DRD4 | OIRDTQYFTABQOQ-KQYNXXCUSA-N | TCMSP | 0.31947565 |
| LC | ADO | SCT | OIRDTQYFTABQOQ-KQYNXXCUSA-N | TCMSP | 0.31947565 |
| LC | ADO | HMOX1 | OIRDTQYFTABQOQ-KQYNXXCUSA-N | TCMSP | 0.31947565 |
| LC | ADO | BDKRB1 | OIRDTQYFTABQOQ-KQYNXXCUSA-N | TCMSP | 0.31947565 |
| LC | ADO | AURKA | OIRDTQYFTABQOQ-KQYNXXCUSA-N | TCMSP | 0.31947565 |
| LC | ADO | PDYN | OIRDTQYFTABQOQ-KQYNXXCUSA-N | TCMSP | 0.31947565 |
| LC | ADO | OXT | OIRDTQYFTABQOQ-KQYNXXCUSA-N | TCMSP | 0.31947565 |
| LC | ADO | AHCY | OIRDTQYFTABQOQ-KQYNXXCUSA-N | TCMSP | 0.31947565 |
| LC | ADO | TIMP1 | OIRDTQYFTABQOQ-KQYNXXCUSA-N | TCMSP | 0.31947565 |
| LC | ADO | C5 | OIRDTQYFTABQOQ-KQYNXXCUSA-N | TCMSP | 0.31947565 |
| LC | ADO | PPY | OIRDTQYFTABQOQ-KQYNXXCUSA-N | TCMSP | 0.31947565 |
| LC | ADO | CXCL6 | OIRDTQYFTABQOQ-KQYNXXCUSA-N | TCMSP | 0.31947565 |
| LC | ADO | GRM6 | OIRDTQYFTABQOQ-KQYNXXCUSA-N | TCMSP | 0.31947565 |
| LC | ADO | OPRD1 | OIRDTQYFTABQOQ-KQYNXXCUSA-N | TCMSP | 0.31947565 |
| LC | ADO | NT5C1A | OIRDTQYFTABQOQ-KQYNXXCUSA-N | TCMSP | 0.31947565 |
| LC | ADO | GHRH | OIRDTQYFTABQOQ-KQYNXXCUSA-N | TCMSP | 0.31947565 |
| LC | ADO | TAS2R8 | OIRDTQYFTABQOQ-KQYNXXCUSA-N | TCMSP | 0.31947565 |
| LC | ADO | TAS2R10 | OIRDTQYFTABQOQ-KQYNXXCUSA-N | TCMSP | 0.31947565 |
| LC | ADO | IAPP | OIRDTQYFTABQOQ-KQYNXXCUSA-N | TCMSP | 0.31947565 |
| LC | ADO | TAS2R7 | OIRDTQYFTABQOQ-KQYNXXCUSA-N | TCMSP | 0.31947565 |
| LC | ADO | NPY | OIRDTQYFTABQOQ-KQYNXXCUSA-N | TCMSP | 0.31947565 |
| LC | ADO | NT5C3A | OIRDTQYFTABQOQ-KQYNXXCUSA-N | TCMSP | 0.31947565 |
| LC | ADO | MC3R | OIRDTQYFTABQOQ-KQYNXXCUSA-N | TCMSP | 0.31947565 |
| LC | ADO | PTGER2 | OIRDTQYFTABQOQ-KQYNXXCUSA-N | TCMSP | 0.31947565 |
| LC | ADO | NT5C | OIRDTQYFTABQOQ-KQYNXXCUSA-N | TCMSP | 0.31947565 |
| LC | ADO | C3 | OIRDTQYFTABQOQ-KQYNXXCUSA-N | TCMSP | 0.31947565 |
| LC | ADO | S1PR4 | OIRDTQYFTABQOQ-KQYNXXCUSA-N | TCMSP | 0.31947565 |
| LC | ADO | CCR7 | OIRDTQYFTABQOQ-KQYNXXCUSA-N | TCMSP | 0.31947565 |
| LC | ADO | TAS2R3 | OIRDTQYFTABQOQ-KQYNXXCUSA-N | TCMSP | 0.31947565 |
| LC | ADO | TAS2R5 | OIRDTQYFTABQOQ-KQYNXXCUSA-N | TCMSP | 0.31947565 |
| LC | ADO | MCHR1 | OIRDTQYFTABQOQ-KQYNXXCUSA-N | TCMSP | 0.31947565 |
| LC | ADO | GALR3 | OIRDTQYFTABQOQ-KQYNXXCUSA-N | TCMSP | 0.31947565 |
| LC | ADO | TAS2R16 | OIRDTQYFTABQOQ-KQYNXXCUSA-N | TCMSP | 0.31947565 |
| LC | ADO | EPO | OIRDTQYFTABQOQ-KQYNXXCUSA-N | TCMSP | 0.31947565 |
| LC | ADO | ADCY7 | OIRDTQYFTABQOQ-KQYNXXCUSA-N | TCMSP | 0.31947565 |
| LC | ADO | SSTR4 | OIRDTQYFTABQOQ-KQYNXXCUSA-N | TCMSP | 0.31947565 |
| LC | ADO | NMUR2 | OIRDTQYFTABQOQ-KQYNXXCUSA-N | TCMSP | 0.31947565 |
| LC | ADO | HRH4 | OIRDTQYFTABQOQ-KQYNXXCUSA-N | TCMSP | 0.31947565 |
| LC | ADO | MTNR1B | OIRDTQYFTABQOQ-KQYNXXCUSA-N | TCMSP | 0.31947565 |
| LC | ADO | APLNR | OIRDTQYFTABQOQ-KQYNXXCUSA-N | TCMSP | 0.31947565 |
| LC | ADO | ANXA1 | OIRDTQYFTABQOQ-KQYNXXCUSA-N | TCMSP | 0.31947565 |
| LC | ADO | NT5E | OIRDTQYFTABQOQ-KQYNXXCUSA-N | TCMSP | 0.31947565 |
| LC | ADO | IL6 | OIRDTQYFTABQOQ-KQYNXXCUSA-N | TCMSP | 0.31947565 |
| LC | ADO | CCL21 | OIRDTQYFTABQOQ-KQYNXXCUSA-N | TCMSP | 0.31947565 |
| LC | ADO | CCL27 | OIRDTQYFTABQOQ-KQYNXXCUSA-N | TCMSP | 0.31947565 |
| LC | ADO | ADCY3 | OIRDTQYFTABQOQ-KQYNXXCUSA-N | TCMSP | 0.31947565 |
| LC | ADO | SLC6A4 | OIRDTQYFTABQOQ-KQYNXXCUSA-N | TCMSP | 0.31947565 |
| LC | ADO | CCNT1 | OIRDTQYFTABQOQ-KQYNXXCUSA-N | TCMSP | 0.31947565 |
| LC | ADO | VIPR2 | OIRDTQYFTABQOQ-KQYNXXCUSA-N | TCMSP | 0.31947565 |
| LC | ADO | GLP2R | OIRDTQYFTABQOQ-KQYNXXCUSA-N | TCMSP | 0.31947565 |
| LC | ADO | ADA2 | OIRDTQYFTABQOQ-KQYNXXCUSA-N | TCMSP | 0.31947565 |
| LC | ADO | KCNN4 | OIRDTQYFTABQOQ-KQYNXXCUSA-N | TCMSP | 0.31947565 |
| LC | ADO | TYR | OIRDTQYFTABQOQ-KQYNXXCUSA-N | TCMSP | 0.31947565 |
| LC | ADO | NMU | OIRDTQYFTABQOQ-KQYNXXCUSA-N | TCMSP | 0.31947565 |
| LC | ADO | MTRR | OIRDTQYFTABQOQ-KQYNXXCUSA-N | TCMSP | 0.31947565 |
| LC | ADO | POMC | OIRDTQYFTABQOQ-KQYNXXCUSA-N | TCMSP | 0.31947565 |
| LC | ADO | KNG1 | OIRDTQYFTABQOQ-KQYNXXCUSA-N | TCMSP | 0.31947565 |
| LC | ADO | AMBP | OIRDTQYFTABQOQ-KQYNXXCUSA-N | TCMSP | 0.31947565 |
| LC | ADO | AFF4 | OIRDTQYFTABQOQ-KQYNXXCUSA-N | TCMSP | 0.31947565 |
| LC | ADO | OPRK1 | OIRDTQYFTABQOQ-KQYNXXCUSA-N | TCMSP | 0.31947565 |
| LC | ADO | GAL | OIRDTQYFTABQOQ-KQYNXXCUSA-N | TCMSP | 0.31947565 |
| LC | ADO | SSTR1 | OIRDTQYFTABQOQ-KQYNXXCUSA-N | TCMSP | 0.31947565 |
| LC | ADO | FGF7 | OIRDTQYFTABQOQ-KQYNXXCUSA-N | TCMSP | 0.31947565 |
| LC | ADO | TP53 | OIRDTQYFTABQOQ-KQYNXXCUSA-N | TCMSP | 0.31947565 |
| LC | ADO | BCKDHA | OIRDTQYFTABQOQ-KQYNXXCUSA-N | TCMSP | 0.31947565 |
| LC | ADO | PTH2 | OIRDTQYFTABQOQ-KQYNXXCUSA-N | TCMSP | 0.31947565 |
| LC | ADO | REN | OIRDTQYFTABQOQ-KQYNXXCUSA-N | TCMSP | 0.31947565 |
| LC | ADO | GPR17 | OIRDTQYFTABQOQ-KQYNXXCUSA-N | TCMSP | 0.31947565 |
| LC | ADO | PTH2R | OIRDTQYFTABQOQ-KQYNXXCUSA-N | TCMSP | 0.31947565 |
| LC | ADO | ACKR3 | OIRDTQYFTABQOQ-KQYNXXCUSA-N | TCMSP | 0.31947565 |
| LC | ADO | BHMT | OIRDTQYFTABQOQ-KQYNXXCUSA-N | TCMSP | 0.31947565 |
| LC | ADO | TAAR1 | OIRDTQYFTABQOQ-KQYNXXCUSA-N | TCMSP | 0.31947565 |
| LC | ADO | CRH | OIRDTQYFTABQOQ-KQYNXXCUSA-N | TCMSP | 0.31947565 |
| LC | ADO | NTRK2 | OIRDTQYFTABQOQ-KQYNXXCUSA-N | TCMSP | 0.31947565 |
| LC | ADO | ADRA2A | OIRDTQYFTABQOQ-KQYNXXCUSA-N | TCMSP | 0.31947565 |
| LC | ADO | MCHR2 | OIRDTQYFTABQOQ-KQYNXXCUSA-N | TCMSP | 0.31947565 |
| LC | ADO | PTH | OIRDTQYFTABQOQ-KQYNXXCUSA-N | TCMSP | 0.31947565 |
| LC | ADO | APP | OIRDTQYFTABQOQ-KQYNXXCUSA-N | TCMSP | 0.31947565 |
| LC | ADO | ADCY8 | OIRDTQYFTABQOQ-KQYNXXCUSA-N | TCMSP | 0.31947565 |
| LC | ADO | ADK | OIRDTQYFTABQOQ-KQYNXXCUSA-N | TCMSP | 0.31947565 |
| LC | ADO | DCK | OIRDTQYFTABQOQ-KQYNXXCUSA-N | TCMSP | 0.31947565 |
| LC | ADO | SLC28A1 | OIRDTQYFTABQOQ-KQYNXXCUSA-N | TCMSP | 0.31947565 |
| LC | ADO | CXCL13 | OIRDTQYFTABQOQ-KQYNXXCUSA-N | TCMSP | 0.31947565 |
| LC | ADO | AIFM1 | OIRDTQYFTABQOQ-KQYNXXCUSA-N | TCMSP | 0.31947565 |
| LC | ADO | SST | OIRDTQYFTABQOQ-KQYNXXCUSA-N | TCMSP | 0.31947565 |
| LC | ADO | HTR5A | OIRDTQYFTABQOQ-KQYNXXCUSA-N | TCMSP | 0.31947565 |
| LC | ADO | HTR6 | OIRDTQYFTABQOQ-KQYNXXCUSA-N | TCMSP | 0.31947565 |
| LC | ADO | PTGIR | OIRDTQYFTABQOQ-KQYNXXCUSA-N | TCMSP | 0.31947565 |
| LC | ADO | CXCR5 | OIRDTQYFTABQOQ-KQYNXXCUSA-N | TCMSP | 0.31947565 |
| LC | ADO | CCR2 | OIRDTQYFTABQOQ-KQYNXXCUSA-N | TCMSP | 0.31947565 |
| LC | ADO | CCR5 | OIRDTQYFTABQOQ-KQYNXXCUSA-N | TCMSP | 0.31947565 |
| LC | ADO | HCRT | OIRDTQYFTABQOQ-KQYNXXCUSA-N | TCMSP | 0.31947565 |
| LC | ADO | CXCL16 | OIRDTQYFTABQOQ-KQYNXXCUSA-N | TCMSP | 0.31947565 |
| LC | ADO | SSTR5 | OIRDTQYFTABQOQ-KQYNXXCUSA-N | TCMSP | 0.31947565 |
| LC | ADO | ADCY9 | OIRDTQYFTABQOQ-KQYNXXCUSA-N | TCMSP | 0.31947565 |
| LC | ADO | LHCGR | OIRDTQYFTABQOQ-KQYNXXCUSA-N | TCMSP | 0.31947565 |
| LC | ADO | CXCR1 | OIRDTQYFTABQOQ-KQYNXXCUSA-N | TCMSP | 0.31947565 |
| LC | ADO | CXCL3 | OIRDTQYFTABQOQ-KQYNXXCUSA-N | TCMSP | 0.31947565 |
| LC | ADO | CXCL5 | OIRDTQYFTABQOQ-KQYNXXCUSA-N | TCMSP | 0.31947565 |
| LC | ADO | PPBP | OIRDTQYFTABQOQ-KQYNXXCUSA-N | TCMSP | 0.31947565 |
| LC | ADO | CCR1 | OIRDTQYFTABQOQ-KQYNXXCUSA-N | TCMSP | 0.31947565 |
| LC | ADO | SLC29A4 | OIRDTQYFTABQOQ-KQYNXXCUSA-N | TCMSP | 0.31947565 |
| LC | ADO | RGS20 | OIRDTQYFTABQOQ-KQYNXXCUSA-N | TCMSP | 0.31947565 |
| LC | ADO | ADCY1 | OIRDTQYFTABQOQ-KQYNXXCUSA-N | TCMSP | 0.31947565 |
| LC | ADO | GPER1 | OIRDTQYFTABQOQ-KQYNXXCUSA-N | TCMSP | 0.31947565 |
| LC | ADO | NOS3 | OIRDTQYFTABQOQ-KQYNXXCUSA-N | TCMSP | 0.31947565 |
| LC | ADO | TSHR | OIRDTQYFTABQOQ-KQYNXXCUSA-N | TCMSP | 0.31947565 |
| LC | ADO | RXFP2 | OIRDTQYFTABQOQ-KQYNXXCUSA-N | TCMSP | 0.31947565 |
| LC | ADO | OXGR1 | OIRDTQYFTABQOQ-KQYNXXCUSA-N | TCMSP | 0.31947565 |
| LC | ADO | GALR1 | OIRDTQYFTABQOQ-KQYNXXCUSA-N | TCMSP | 0.31947565 |
| LC | ADO | MC4R | OIRDTQYFTABQOQ-KQYNXXCUSA-N | TCMSP | 0.31947565 |
| LC | ADO | NNMT | OIRDTQYFTABQOQ-KQYNXXCUSA-N | TCMSP | 0.31947565 |
| LC | ADO | PNOC | OIRDTQYFTABQOQ-KQYNXXCUSA-N | TCMSP | 0.31947565 |
| LC | ADO | C3AR1 | OIRDTQYFTABQOQ-KQYNXXCUSA-N | TCMSP | 0.31947565 |
| LC | ADO | FPR1 | OIRDTQYFTABQOQ-KQYNXXCUSA-N | TCMSP | 0.31947565 |
| LC | ADO | MTNR1A | OIRDTQYFTABQOQ-KQYNXXCUSA-N | TCMSP | 0.31947565 |
| LC | ADO | PTGER4 | OIRDTQYFTABQOQ-KQYNXXCUSA-N | TCMSP | 0.31947565 |
| LC | ADO | MAT2A | OIRDTQYFTABQOQ-KQYNXXCUSA-N | TCMSP | 0.31947565 |
| LC | ADO | RXFP1 | OIRDTQYFTABQOQ-KQYNXXCUSA-N | TCMSP | 0.31947565 |
| LC | ADO | PTGDR | OIRDTQYFTABQOQ-KQYNXXCUSA-N | TCMSP | 0.31947565 |
| LC | ADO | HINT1 | OIRDTQYFTABQOQ-KQYNXXCUSA-N | TCMSP | 0.31947565 |
| LC | ADO | RAC3 | OIRDTQYFTABQOQ-KQYNXXCUSA-N | TCMSP | 0.31947565 |
| LC | ADO | CXCR6 | OIRDTQYFTABQOQ-KQYNXXCUSA-N | TCMSP | 0.31947565 |
| LC | ADO | ADORA2B | OIRDTQYFTABQOQ-KQYNXXCUSA-N | TCMSP | 0.31947565 |
| LC | ADO | P2RY1 | OIRDTQYFTABQOQ-KQYNXXCUSA-N | TCMSP | 0.31947565 |
| LC | ADO | IL13 | OIRDTQYFTABQOQ-KQYNXXCUSA-N | TCMSP | 0.31947565 |
| LC | ADO | ADRB2 | OIRDTQYFTABQOQ-KQYNXXCUSA-N | TCMSP | 0.31947565 |
| LC | ADO | S1PR1 | OIRDTQYFTABQOQ-KQYNXXCUSA-N | TCMSP | 0.31947565 |
| LC | ADO | CXCL10 | OIRDTQYFTABQOQ-KQYNXXCUSA-N | TCMSP | 0.31947565 |
| LC | ADO | NMUR1 | OIRDTQYFTABQOQ-KQYNXXCUSA-N | TCMSP | 0.31947565 |
| LC | ADO | DRD5 | OIRDTQYFTABQOQ-KQYNXXCUSA-N | TCMSP | 0.31947565 |
| LC | ADO | FOS | OIRDTQYFTABQOQ-KQYNXXCUSA-N | TCMSP | 0.31947565 |
| LC | ADO | CXCL8 | OIRDTQYFTABQOQ-KQYNXXCUSA-N | TCMSP | 0.31947565 |
| LC | ADO | CXCL11 | OIRDTQYFTABQOQ-KQYNXXCUSA-N | TCMSP | 0.31947565 |
| LC | ADO | P2RY12 | OIRDTQYFTABQOQ-KQYNXXCUSA-N | TCMSP | 0.31947565 |
| LC | ADO | HTR1E | OIRDTQYFTABQOQ-KQYNXXCUSA-N | TCMSP | 0.31947565 |
| LC | ADO | P2RY14 | OIRDTQYFTABQOQ-KQYNXXCUSA-N | TCMSP | 0.31947565 |
| LC | ADO | ADORA1 | OIRDTQYFTABQOQ-KQYNXXCUSA-N | TCMSP | 0.31947565 |
| LC | ADO | CCL19 | OIRDTQYFTABQOQ-KQYNXXCUSA-N | TCMSP | 0.31947565 |
| LC | ADO | CASP3 | OIRDTQYFTABQOQ-KQYNXXCUSA-N | TCMSP | 0.31947565 |
| LC | ADO | ADCY6 | OIRDTQYFTABQOQ-KQYNXXCUSA-N | TCMSP | 0.31947565 |
| LC | ADO | ADCY4 | OIRDTQYFTABQOQ-KQYNXXCUSA-N | TCMSP | 0.31947565 |
| LC | ADO | TPMT | OIRDTQYFTABQOQ-KQYNXXCUSA-N | TCMSP | 0.31947565 |
| LC | ADO | GNAI2 | OIRDTQYFTABQOQ-KQYNXXCUSA-N | TCMSP | 0.31947565 |
| LC | ADO | HNRNPD | OIRDTQYFTABQOQ-KQYNXXCUSA-N | TCMSP | 0.31947565 |
| LC | ADO | SPHK1 | OIRDTQYFTABQOQ-KQYNXXCUSA-N | TCMSP | 0.31947565 |
| LC | ADO | SLC28A2 | OIRDTQYFTABQOQ-KQYNXXCUSA-N | TCMSP | 0.31947565 |
| LC | ADO | AHCYL2 | OIRDTQYFTABQOQ-KQYNXXCUSA-N | TCMSP | 0.31947565 |
| LC | ADO | HTR1A | OIRDTQYFTABQOQ-KQYNXXCUSA-N | TCMSP | 0.31947565 |
| LC | ADO | MC5R | OIRDTQYFTABQOQ-KQYNXXCUSA-N | TCMSP | 0.31947565 |
| LC | ADO | MARCO | OIRDTQYFTABQOQ-KQYNXXCUSA-N | TCMSP | 0.31947565 |
| LC | ADO | CXCR2 | OIRDTQYFTABQOQ-KQYNXXCUSA-N | TCMSP | 0.31947565 |
| LC | ADO | CHRM2 | OIRDTQYFTABQOQ-KQYNXXCUSA-N | TCMSP | 0.31947565 |
| LC | ADO | GHRHR | OIRDTQYFTABQOQ-KQYNXXCUSA-N | TCMSP | 0.31947565 |
| LC | ADO | P2RY13 | OIRDTQYFTABQOQ-KQYNXXCUSA-N | TCMSP | 0.31947565 |
| LC | ADO | PTH1R | OIRDTQYFTABQOQ-KQYNXXCUSA-N | TCMSP | 0.31947565 |
| LC | ADO | MMP1 | OIRDTQYFTABQOQ-KQYNXXCUSA-N | TCMSP | 0.31947565 |
| LC | ADO | HTR1F | OIRDTQYFTABQOQ-KQYNXXCUSA-N | TCMSP | 0.31947565 |
| LC | ADO | PENK | OIRDTQYFTABQOQ-KQYNXXCUSA-N | TCMSP | 0.31947565 |
| LC | ADO | MAT2B | OIRDTQYFTABQOQ-KQYNXXCUSA-N | TCMSP | 0.31947565 |
| LC | ADO | CCR8 | OIRDTQYFTABQOQ-KQYNXXCUSA-N | TCMSP | 0.31947565 |
| LC | ADO | VIPR1 | OIRDTQYFTABQOQ-KQYNXXCUSA-N | TCMSP | 0.31947565 |
| LC | ADO | TAS2R60 | OIRDTQYFTABQOQ-KQYNXXCUSA-N | TCMSP | 0.31947565 |
| LC | ADO | LPAR5 | OIRDTQYFTABQOQ-KQYNXXCUSA-N | TCMSP | 0.31947565 |
| LC | ADO | S1PR5 | OIRDTQYFTABQOQ-KQYNXXCUSA-N | TCMSP | 0.31947565 |
| LC | ADO | RXFP3 | OIRDTQYFTABQOQ-KQYNXXCUSA-N | TCMSP | 0.31947565 |
| LC | ADO | GALR2 | OIRDTQYFTABQOQ-KQYNXXCUSA-N | TCMSP | 0.31947565 |
| LC | ADO | NPW | OIRDTQYFTABQOQ-KQYNXXCUSA-N | TCMSP | 0.31947565 |
| LC | ADO | NPBWR1 | OIRDTQYFTABQOQ-KQYNXXCUSA-N | TCMSP | 0.31947565 |
| LC | ADO | CALCA | OIRDTQYFTABQOQ-KQYNXXCUSA-N | TCMSP | 0.31947565 |
| LC | ADO | PMCH | OIRDTQYFTABQOQ-KQYNXXCUSA-N | TCMSP | 0.31947565 |
| LC | ADO | ADAT3 | OIRDTQYFTABQOQ-KQYNXXCUSA-N | TCMSP | 0.31947565 |
| LC | ADO | CCR10 | OIRDTQYFTABQOQ-KQYNXXCUSA-N | TCMSP | 0.31947565 |
| LC | ADO | NPY2R | OIRDTQYFTABQOQ-KQYNXXCUSA-N | TCMSP | 0.31947565 |
| LC | ADO | CCR4 | OIRDTQYFTABQOQ-KQYNXXCUSA-N | TCMSP | 0.31947565 |
| LC | ADO | NPB | OIRDTQYFTABQOQ-KQYNXXCUSA-N | TCMSP | 0.31947565 |
| LC | ADO | PTGDR2 | OIRDTQYFTABQOQ-KQYNXXCUSA-N | TCMSP | 0.31947565 |
| LC | ADO | RGS19 | OIRDTQYFTABQOQ-KQYNXXCUSA-N | TCMSP | 0.31947565 |
| LC | ADO | MC2R | OIRDTQYFTABQOQ-KQYNXXCUSA-N | TCMSP | 0.31947565 |
| LC | ADO | F3 | OIRDTQYFTABQOQ-KQYNXXCUSA-N | TCMSP | 0.31947565 |
| LC | ADO | GNG2 | OIRDTQYFTABQOQ-KQYNXXCUSA-N | TCMSP | 0.31947565 |
| LC | ADO | GHRL | OIRDTQYFTABQOQ-KQYNXXCUSA-N | TCMSP | 0.31947565 |
| LC | ADO | ADORA2A | OIRDTQYFTABQOQ-KQYNXXCUSA-N | TCMSP | 0.31947565 |
| LC | ADO | OPRL1 | OIRDTQYFTABQOQ-KQYNXXCUSA-N | TCMSP | 0.31947565 |
| LC | ADO | HTR7 | OIRDTQYFTABQOQ-KQYNXXCUSA-N | TCMSP | 0.31947565 |
| LC | ADO | HIF1A | OIRDTQYFTABQOQ-KQYNXXCUSA-N | TCMSP | 0.31947565 |
| LC | ADO | AVPR2 | OIRDTQYFTABQOQ-KQYNXXCUSA-N | TCMSP | 0.31947565 |
| LC | ADO | NPY5R | OIRDTQYFTABQOQ-KQYNXXCUSA-N | TCMSP | 0.31947565 |
| LC | ADO | CCR6 | OIRDTQYFTABQOQ-KQYNXXCUSA-N | TCMSP | 0.31947565 |
| LC | ADO | NT5C2 | OIRDTQYFTABQOQ-KQYNXXCUSA-N | TCMSP | 0.31947565 |
| LC | ADO | PKLR | OIRDTQYFTABQOQ-KQYNXXCUSA-N | TCMSP | 0.31947565 |
| LC | ADO | FPR2 | OIRDTQYFTABQOQ-KQYNXXCUSA-N | TCMSP | 0.31947565 |
| LC | ADO | CRHR2 | OIRDTQYFTABQOQ-KQYNXXCUSA-N | TCMSP | 0.31947565 |
| LC | ADO | FPR3 | OIRDTQYFTABQOQ-KQYNXXCUSA-N | TCMSP | 0.31947565 |
| LC | ADO | HRH3 | OIRDTQYFTABQOQ-KQYNXXCUSA-N | TCMSP | 0.31947565 |
| LC | ADO | ADCY2 | OIRDTQYFTABQOQ-KQYNXXCUSA-N | TCMSP | 0.31947565 |
| LC | ADO | GNAI1 | OIRDTQYFTABQOQ-KQYNXXCUSA-N | TCMSP | 0.31947565 |
| LC | ADO | GPR18 | OIRDTQYFTABQOQ-KQYNXXCUSA-N | TCMSP | 0.31947565 |
| LC | ADO | ADRB3 | OIRDTQYFTABQOQ-KQYNXXCUSA-N | TCMSP | 0.31947565 |
| LC | ADO | GRM8 | OIRDTQYFTABQOQ-KQYNXXCUSA-N | TCMSP | 0.31947565 |
| LC | ADO | RNASE1 | OIRDTQYFTABQOQ-KQYNXXCUSA-N | TCMSP | 0.31947565 |
| LC | ADO | CALCB | OIRDTQYFTABQOQ-KQYNXXCUSA-N | TCMSP | 0.31947565 |
| LC | ADO | FYN | OIRDTQYFTABQOQ-KQYNXXCUSA-N | TCMSP | 0.31947565 |
| LC | ADO | FN1 | OIRDTQYFTABQOQ-KQYNXXCUSA-N | TCMSP | 0.31947565 |
| LC | ADO | C5AR1 | OIRDTQYFTABQOQ-KQYNXXCUSA-N | TCMSP | 0.31947565 |
| LC | ADO | RET | OIRDTQYFTABQOQ-KQYNXXCUSA-N | TCMSP | 0.31947565 |
| LC | ADO | RAC1 | OIRDTQYFTABQOQ-KQYNXXCUSA-N | TCMSP | 0.31947565 |
| LC | ADO | SAA1 | OIRDTQYFTABQOQ-KQYNXXCUSA-N | TCMSP | 0.31947565 |
| LC | ADO | PTGER3 | OIRDTQYFTABQOQ-KQYNXXCUSA-N | TCMSP | 0.31947565 |
| LC | ADO | GIP | OIRDTQYFTABQOQ-KQYNXXCUSA-N | TCMSP | 0.31947565 |
| LC | ADO | SLC29A2 | OIRDTQYFTABQOQ-KQYNXXCUSA-N | TCMSP | 0.31947565 |
| LC | ADO | SSTR2 | OIRDTQYFTABQOQ-KQYNXXCUSA-N | TCMSP | 0.31947565 |
| LC | ADO | CCR9 | OIRDTQYFTABQOQ-KQYNXXCUSA-N | TCMSP | 0.31947565 |
| LC | ADO | GRM7 | OIRDTQYFTABQOQ-KQYNXXCUSA-N | TCMSP | 0.31947565 |
| LC | ADO | DDC | OIRDTQYFTABQOQ-KQYNXXCUSA-N | TCMSP | 0.31947565 |
| LC | ADO | S1PR3 | OIRDTQYFTABQOQ-KQYNXXCUSA-N | TCMSP | 0.31947565 |
| LC | ADO | CCL20 | OIRDTQYFTABQOQ-KQYNXXCUSA-N | TCMSP | 0.31947565 |
| LC | ADO | LPAR1 | OIRDTQYFTABQOQ-KQYNXXCUSA-N | TCMSP | 0.31947565 |
| LC | ADO | CALCR | OIRDTQYFTABQOQ-KQYNXXCUSA-N | TCMSP | 0.31947565 |
| LC | ADO | NPSR1 | OIRDTQYFTABQOQ-KQYNXXCUSA-N | TCMSP | 0.31947565 |
| LC | ADO | NT5C1B-RDH14 | OIRDTQYFTABQOQ-KQYNXXCUSA-N | TCMSP | 0.31947565 |
| LC | ADO | PYY | OIRDTQYFTABQOQ-KQYNXXCUSA-N | TCMSP | 0.31947565 |
| LC | ADO | DPP4 | OIRDTQYFTABQOQ-KQYNXXCUSA-N | TCMSP | 0.31947565 |
| LC | ADO | HTR4 | OIRDTQYFTABQOQ-KQYNXXCUSA-N | TCMSP | 0.31947565 |
| LC | ADO | CCL28 | OIRDTQYFTABQOQ-KQYNXXCUSA-N | TCMSP | 0.31947565 |
| LC | ADO | COMT | OIRDTQYFTABQOQ-KQYNXXCUSA-N | TCMSP | 0.31947565 |
| LC | ADO | PNP | OIRDTQYFTABQOQ-KQYNXXCUSA-N | TCMSP | 0.31947565 |
| LC | ADO | NPY1R | OIRDTQYFTABQOQ-KQYNXXCUSA-N | TCMSP | 0.31947565 |
| LC | ADO | DRD2 | OIRDTQYFTABQOQ-KQYNXXCUSA-N | TCMSP | 0.31947565 |
| LC | ADO | CXCL9 | OIRDTQYFTABQOQ-KQYNXXCUSA-N | TCMSP | 0.31947565 |
| LC | ADO | SUCNR1 | OIRDTQYFTABQOQ-KQYNXXCUSA-N | TCMSP | 0.31947565 |
| LC | ADO | GRM3 | OIRDTQYFTABQOQ-KQYNXXCUSA-N | TCMSP | 0.31947565 |
| LC | ADO | WDTC1 | OIRDTQYFTABQOQ-KQYNXXCUSA-N | TCMSP | 0.31947565 |
| LC | ADO | RGS7 | OIRDTQYFTABQOQ-KQYNXXCUSA-N | TCMSP | 0.31947565 |
| LC | ADO | MTR | OIRDTQYFTABQOQ-KQYNXXCUSA-N | TCMSP | 0.31947565 |
| LC | ADO | AGT | OIRDTQYFTABQOQ-KQYNXXCUSA-N | TCMSP | 0.31947565 |
| LC | ADO | EPRS | OIRDTQYFTABQOQ-KQYNXXCUSA-N | TCMSP | 0.31947565 |
| LC | ADO | VIP | OIRDTQYFTABQOQ-KQYNXXCUSA-N | TCMSP | 0.31947565 |
| LC | ADO | RGS1 | OIRDTQYFTABQOQ-KQYNXXCUSA-N | TCMSP | 0.31947565 |
| LC | ADO | RGS18 | OIRDTQYFTABQOQ-KQYNXXCUSA-N | TCMSP | 0.31947565 |
| LC | ADO | RXFP4 | OIRDTQYFTABQOQ-KQYNXXCUSA-N | TCMSP | 0.31947565 |
| LC | ADO | RGS10 | OIRDTQYFTABQOQ-KQYNXXCUSA-N | TCMSP | 0.31947565 |
| LC | ADO | ADRB1 | OIRDTQYFTABQOQ-KQYNXXCUSA-N | TCMSP | 0.31947565 |
| LC | ADO | MAP3K7 | OIRDTQYFTABQOQ-KQYNXXCUSA-N | TCMSP | 0.31947565 |
| LC | ADO | CNR1 | OIRDTQYFTABQOQ-KQYNXXCUSA-N | TCMSP | 0.31947565 |
| LC | ADO | TMIGD3 | OIRDTQYFTABQOQ-KQYNXXCUSA-N | TCMSP | 0.31947565 |
| LC | ADO | NPBWR2 | OIRDTQYFTABQOQ-KQYNXXCUSA-N | TCMSP | 0.31947565 |
| LC | ADO | AHCYL1 | OIRDTQYFTABQOQ-KQYNXXCUSA-N | TCMSP | 0.31947565 |
| LC | ADO | GNAI3 | OIRDTQYFTABQOQ-KQYNXXCUSA-N | TCMSP | 0.31947565 |
| LC | ADO | HTR1B | OIRDTQYFTABQOQ-KQYNXXCUSA-N | TCMSP | 0.31947565 |
| LC | ADO | LPAR3 | OIRDTQYFTABQOQ-KQYNXXCUSA-N | TCMSP | 0.31947565 |
| LC | ADO | PI4K2A | OIRDTQYFTABQOQ-KQYNXXCUSA-N | TCMSP | 0.31947565 |
| LC | ADO | GNAS | OIRDTQYFTABQOQ-KQYNXXCUSA-N | TCMSP | 0.31947565 |
| LC | ADO | SLC29A1 | OIRDTQYFTABQOQ-KQYNXXCUSA-N | TCMSP | 0.31947565 |
| LC | ADO | AGTR2 | OIRDTQYFTABQOQ-KQYNXXCUSA-N | TCMSP | 0.31947565 |
| LC | ADO | MMP9 | OIRDTQYFTABQOQ-KQYNXXCUSA-N | TCMSP | 0.31947565 |
| LC | ADO | ADA | OIRDTQYFTABQOQ-KQYNXXCUSA-N | TCMSP | 0.31947565 |
| LC | ADO | SLC29A3 | OIRDTQYFTABQOQ-KQYNXXCUSA-N | TCMSP | 0.31947565 |
| LC | ADO | GLP1R | OIRDTQYFTABQOQ-KQYNXXCUSA-N | TCMSP | 0.31947565 |
| LC | ADO | CDK9 | OIRDTQYFTABQOQ-KQYNXXCUSA-N | TCMSP | 0.31947565 |
| LC | ADO | PIM1 | OIRDTQYFTABQOQ-KQYNXXCUSA-N | TCMSP | 0.31947565 |
| LC | ADO | CXCR3 | OIRDTQYFTABQOQ-KQYNXXCUSA-N | TCMSP | 0.31947565 |
| LC | ADO | PRKG1 | OIRDTQYFTABQOQ-KQYNXXCUSA-N | TCMSP | 0.31947565 |
| LC | ADO | GRM4 | OIRDTQYFTABQOQ-KQYNXXCUSA-N | TCMSP | 0.31947565 |
| LC | ADO | NPY4R2 | OIRDTQYFTABQOQ-KQYNXXCUSA-N | TCMSP | 0.31947565 |
| LC | ADO | CNR2 | OIRDTQYFTABQOQ-KQYNXXCUSA-N | TCMSP | 0.31947565 |
| LC | ADO | P2RY4 | OIRDTQYFTABQOQ-KQYNXXCUSA-N | TCMSP | 0.31947565 |
| LC | ADO | AR | OIRDTQYFTABQOQ-KQYNXXCUSA-N | TCMSP | 0.31947565 |
| LC | ADO | SLC28A3 | OIRDTQYFTABQOQ-KQYNXXCUSA-N | TCMSP | 0.31947565 |
| LC | ADO | NMS | OIRDTQYFTABQOQ-KQYNXXCUSA-N | TCMSP | 0.31947565 |
| LC | ADO | HRH2 | OIRDTQYFTABQOQ-KQYNXXCUSA-N | TCMSP | 0.31947565 |
| LC | ADO | OXER1 | OIRDTQYFTABQOQ-KQYNXXCUSA-N | TCMSP | 0.31947565 |
| LC | ADO | EDN1 | OIRDTQYFTABQOQ-KQYNXXCUSA-N | TCMSP | 0.31947565 |
| LC | ADO | AVP | OIRDTQYFTABQOQ-KQYNXXCUSA-N | TCMSP | 0.31947565 |
| LC | ADO | RDH14 | OIRDTQYFTABQOQ-KQYNXXCUSA-N | TCMSP | 0.31947565 |
| LC | ADO | ATP8A1 | OIRDTQYFTABQOQ-KQYNXXCUSA-N | TCMSP | 0.31947565 |
| LC | ADO | TAS2R1 | OIRDTQYFTABQOQ-KQYNXXCUSA-N | TCMSP | 0.31947565 |
| LC | ADO | DRD3 | OIRDTQYFTABQOQ-KQYNXXCUSA-N | TCMSP | 0.31947565 |
| LC | ADO | NT5M | OIRDTQYFTABQOQ-KQYNXXCUSA-N | TCMSP | 0.31947565 |
| LC | ADO | HCAR2 | OIRDTQYFTABQOQ-KQYNXXCUSA-N | TCMSP | 0.31947565 |
| LC | ADO | CCL25 | OIRDTQYFTABQOQ-KQYNXXCUSA-N | TCMSP | 0.31947565 |
| LC | ADO | TAS2R19 | OIRDTQYFTABQOQ-KQYNXXCUSA-N | TCMSP | 0.31947565 |
| LC | ADO | TAS2R31 | OIRDTQYFTABQOQ-KQYNXXCUSA-N | TCMSP | 0.31947565 |
| LC | ADO | TAS2R13 | OIRDTQYFTABQOQ-KQYNXXCUSA-N | TCMSP | 0.31947565 |
| LC | ADO | GPR55 | OIRDTQYFTABQOQ-KQYNXXCUSA-N | TCMSP | 0.31947565 |
| LC | ADO | GRM2 | OIRDTQYFTABQOQ-KQYNXXCUSA-N | TCMSP | 0.31947565 |
| LC | ADO | ADM2 | OIRDTQYFTABQOQ-KQYNXXCUSA-N | TCMSP | 0.31947565 |
| LC | ADO | CXCL1 | OIRDTQYFTABQOQ-KQYNXXCUSA-N | TCMSP | 0.31947565 |
| LC | ADO | CXCL12 | OIRDTQYFTABQOQ-KQYNXXCUSA-N | TCMSP | 0.31947565 |
| LC | ADO | PTHLH | OIRDTQYFTABQOQ-KQYNXXCUSA-N | TCMSP | 0.31947565 |
| LC | ADO | ADCYAP1R1 | OIRDTQYFTABQOQ-KQYNXXCUSA-N | TCMSP | 0.31947565 |
| LC | ADO | NPS | OIRDTQYFTABQOQ-KQYNXXCUSA-N | TCMSP | 0.31947565 |
| LC | ADO | LINC02210-CRHR1 | OIRDTQYFTABQOQ-KQYNXXCUSA-N | TCMSP | 0.31947565 |
| LC | ADO | GNAT3 | OIRDTQYFTABQOQ-KQYNXXCUSA-N | TCMSP | 0.31947565 |
| LC | ADO | GCGR | OIRDTQYFTABQOQ-KQYNXXCUSA-N | TCMSP | 0.31947565 |
| LC | ADO | LPAR2 | OIRDTQYFTABQOQ-KQYNXXCUSA-N | TCMSP | 0.31947565 |
| LC | ADO | ADRA2C | OIRDTQYFTABQOQ-KQYNXXCUSA-N | TCMSP | 0.31947565 |
| LC | ADO | TAS2R41 | OIRDTQYFTABQOQ-KQYNXXCUSA-N | TCMSP | 0.31947565 |
| LC | ADO | TAS2R40 | OIRDTQYFTABQOQ-KQYNXXCUSA-N | TCMSP | 0.31947565 |
| LC | ADO | RGS14 | OIRDTQYFTABQOQ-KQYNXXCUSA-N | TCMSP | 0.31947565 |
| LC | ADO | CXCR4 | OIRDTQYFTABQOQ-KQYNXXCUSA-N | TCMSP | 0.31947565 |
| LC | ADO | GCG | OIRDTQYFTABQOQ-KQYNXXCUSA-N | TCMSP | 0.31947565 |
| LC | ADO | CREB1 | OIRDTQYFTABQOQ-KQYNXXCUSA-N | TCMSP | 0.31947565 |
| LC | ADO | NT5C3B | OIRDTQYFTABQOQ-KQYNXXCUSA-N | TCMSP | 0.31947565 |
| LC | ADO | OPRM1 | OIRDTQYFTABQOQ-KQYNXXCUSA-N | TCMSP | 0.31947565 |
| LC | ADO | RGS4 | OIRDTQYFTABQOQ-KQYNXXCUSA-N | TCMSP | 0.31947565 |
| LC | ADO | TNF | OIRDTQYFTABQOQ-KQYNXXCUSA-N | TCMSP | 0.31947565 |
| LC | ADO | RHOA | OIRDTQYFTABQOQ-KQYNXXCUSA-N | TCMSP | 0.31947565 |
| LC | ADO | TAS2R39 | OIRDTQYFTABQOQ-KQYNXXCUSA-N | TCMSP | 0.31947565 |
| LC | ADO | GDNF | OIRDTQYFTABQOQ-KQYNXXCUSA-N | TCMSP | 0.31947565 |
| LC | ADO | CHRM4 | OIRDTQYFTABQOQ-KQYNXXCUSA-N | TCMSP | 0.31947565 |
| LC | ADO | CASP1 | OIRDTQYFTABQOQ-KQYNXXCUSA-N | TCMSP | 0.31947565 |
| LC | ADO | ADCYAP1 | OIRDTQYFTABQOQ-KQYNXXCUSA-N | TCMSP | 0.31947565 |
| LC | ADO | IL10 | OIRDTQYFTABQOQ-KQYNXXCUSA-N | TCMSP | 0.31947565 |
| LC | ADO | ADAL | OIRDTQYFTABQOQ-KQYNXXCUSA-N | TCMSP | 0.31947565 |
| LC | ADO | ADCY5 | OIRDTQYFTABQOQ-KQYNXXCUSA-N | TCMSP | 0.31947565 |
| LC | ADO | TAS2R50 | OIRDTQYFTABQOQ-KQYNXXCUSA-N | TCMSP | 0.31947565 |
| LC | ADO | CXCL2 | OIRDTQYFTABQOQ-KQYNXXCUSA-N | TCMSP | 0.31947565 |
| LC | ADO | GPBAR1 | OIRDTQYFTABQOQ-KQYNXXCUSA-N | TCMSP | 0.31947565 |
| LC | ADO | NTRK1 | OIRDTQYFTABQOQ-KQYNXXCUSA-N | TCMSP | 0.31947565 |
| LC | ADO | TAS2R43 | OIRDTQYFTABQOQ-KQYNXXCUSA-N | TCMSP | 0.31947565 |
| LC | ADO | TAS2R46 | OIRDTQYFTABQOQ-KQYNXXCUSA-N | TCMSP | 0.31947565 |
| LC | ADO | HCAR3 | OIRDTQYFTABQOQ-KQYNXXCUSA-N | TCMSP | 0.31947565 |
| LC | ADO | CCR3 | OIRDTQYFTABQOQ-KQYNXXCUSA-N | TCMSP | 0.31947565 |
| LC | ADO | TAS2R20 | OIRDTQYFTABQOQ-KQYNXXCUSA-N | TCMSP | 0.31947565 |
| LC | ADO | TAS2R14 | OIRDTQYFTABQOQ-KQYNXXCUSA-N | TCMSP | 0.31947565 |
| LC | ADO | TAS2R30 | OIRDTQYFTABQOQ-KQYNXXCUSA-N | TCMSP | 0.31947565 |
| LC | ADO | TAS2R38 | OIRDTQYFTABQOQ-KQYNXXCUSA-N | TCMSP | 0.31947565 |
| LC | ADO | RGS6 | OIRDTQYFTABQOQ-KQYNXXCUSA-N | TCMSP | 0.31947565 |
| LC | ADO | MC1R | OIRDTQYFTABQOQ-KQYNXXCUSA-N | TCMSP | 0.31947565 |
| LC | ADO | S1PR2 | OIRDTQYFTABQOQ-KQYNXXCUSA-N | TCMSP | 0.31947565 |
| LC | ADO | GIPR | OIRDTQYFTABQOQ-KQYNXXCUSA-N | TCMSP | 0.31947565 |
| LC | adenine | MRI1 | GFFGJBXGBJISGV-UHFFFAOYSA-N | TCMSP | 0.506373008 |
| LC | adenine | PYGM | GFFGJBXGBJISGV-UHFFFAOYSA-N | TCMSP | 0.506373008 |
| LC | adenine | ALKBH1 | GFFGJBXGBJISGV-UHFFFAOYSA-N | TCMSP | 0.506373008 |
| LC | adenine | SF3B6 | GFFGJBXGBJISGV-UHFFFAOYSA-N | TCMSP | 0.506373008 |
| LC | adenine | ALKBH7 | GFFGJBXGBJISGV-UHFFFAOYSA-N | TCMSP | 0.506373008 |
| LC | adenine | PECR | GFFGJBXGBJISGV-UHFFFAOYSA-N | TCMSP | 0.506373008 |
| LC | adenine | PNMT | GFFGJBXGBJISGV-UHFFFAOYSA-N | TCMSP | 0.506373008 |
| LC | adenine | ACP1 | GFFGJBXGBJISGV-UHFFFAOYSA-N | TCMSP | 0.506373008 |
| LC | adenine | ALKBH4 | GFFGJBXGBJISGV-UHFFFAOYSA-N | TCMSP | 0.506373008 |
| LC | adenine | HPRT1 | GFFGJBXGBJISGV-UHFFFAOYSA-N | TCMSP | 0.506373008 |
| LC | adenine | ALKBH3 | GFFGJBXGBJISGV-UHFFFAOYSA-N | TCMSP | 0.506373008 |
| LC | adenine | P2RY1 | GFFGJBXGBJISGV-UHFFFAOYSA-N | TCMSP | 0.506373008 |
| LC | adenine | KNTC1 | GFFGJBXGBJISGV-UHFFFAOYSA-N | TCMSP | 0.506373008 |
| LC | adenine | HSP90AA1 | GFFGJBXGBJISGV-UHFFFAOYSA-N | TCMSP | 0.506373008 |
| LC | adenine | SF3B1 | GFFGJBXGBJISGV-UHFFFAOYSA-N | TCMSP | 0.506373008 |
| LC | adenine | ACVR2B | GFFGJBXGBJISGV-UHFFFAOYSA-N | TCMSP | 0.506373008 |
| LC | adenine | ACACB | GFFGJBXGBJISGV-UHFFFAOYSA-N | TCMSP | 0.506373008 |
| LC | adenine | SLC29A2 | GFFGJBXGBJISGV-UHFFFAOYSA-N | TCMSP | 0.506373008 |
| LC | adenine | PNP | GFFGJBXGBJISGV-UHFFFAOYSA-N | TCMSP | 0.506373008 |
| LC | adenine | MUTYH | GFFGJBXGBJISGV-UHFFFAOYSA-N | TCMSP | 0.506373008 |
| LC | adenine | SLC29A3 | GFFGJBXGBJISGV-UHFFFAOYSA-N | TCMSP | 0.506373008 |
| LC | adenine | STK24 | GFFGJBXGBJISGV-UHFFFAOYSA-N | TCMSP | 0.506373008 |
| LC | adenine | APRT | GFFGJBXGBJISGV-UHFFFAOYSA-N | TCMSP | 0.506373008 |
| LC | adenine | MTAP | GFFGJBXGBJISGV-UHFFFAOYSA-N | TCMSP | 0.506373008 |
| LC | adenine | ALKBH8 | GFFGJBXGBJISGV-UHFFFAOYSA-N | TCMSP | 0.506373008 |
| LC | adenine | SRPK2 | GFFGJBXGBJISGV-UHFFFAOYSA-N | TCMSP | 0.506373008 |
| LC | adenine | ALKBH5 | GFFGJBXGBJISGV-UHFFFAOYSA-N | TCMSP | 0.506373008 |
| LC | adenine | CHFR | GFFGJBXGBJISGV-UHFFFAOYSA-N | TCMSP | 0.506373008 |
| LC | adenine | XPNPEP1 | GFFGJBXGBJISGV-UHFFFAOYSA-N | TCMSP | 0.506373008 |
| LC | OCT | PRKACA | TVMXDCGIABBOFY-UHFFFAOYSA-N | TCMSP | 0.326992876 |
| LC | OCT | CYP4A11 | TVMXDCGIABBOFY-UHFFFAOYSA-N | TCMSP | 0.326992876 |
| LC | OCT | GLTP | TVMXDCGIABBOFY-UHFFFAOYSA-N | TCMSP | 0.326992876 |
| LC | OCT | CYP4A22 | TVMXDCGIABBOFY-UHFFFAOYSA-N | TCMSP | 0.326992876 |
| LC | PLO | SULT2B1 | ORNBQBCIOKFOEO-QGVNFLHTSA-N | TCMSP | 0.790491035 |
| LC | PLO | STS | ORNBQBCIOKFOEO-QGVNFLHTSA-N | TCMSP | 0.790491035 |
| LC | PLO | LHB | ORNBQBCIOKFOEO-QGVNFLHTSA-N | TCMSP | 0.790491035 |
| LC | PLO | SULT2A1 | ORNBQBCIOKFOEO-QGVNFLHTSA-N | TCMSP | 0.790491035 |
| LC | PLO | POMC | ORNBQBCIOKFOEO-QGVNFLHTSA-N | TCMSP | 0.790491035 |
| LC | PLO | CYP11A1 | ORNBQBCIOKFOEO-QGVNFLHTSA-N | TCMSP | 0.790491035 |
| LC | PLO | STAR | ORNBQBCIOKFOEO-QGVNFLHTSA-N | TCMSP | 0.790491035 |
| LC | PLO | CLIP1 | ORNBQBCIOKFOEO-QGVNFLHTSA-N | TCMSP | 0.790491035 |
| LC | PLO | CYP7B1 | ORNBQBCIOKFOEO-QGVNFLHTSA-N | TCMSP | 0.790491035 |
| LC | PLO | BPNT1 | ORNBQBCIOKFOEO-QGVNFLHTSA-N | TCMSP | 0.790491035 |
| LC | PLO | NR1I2 | ORNBQBCIOKFOEO-QGVNFLHTSA-N | TCMSP | 0.790491035 |
| LC | PLO | HSD3B1 | ORNBQBCIOKFOEO-QGVNFLHTSA-N | TCMSP | 0.790491035 |
| LC | PLO | HSD3B2 | ORNBQBCIOKFOEO-QGVNFLHTSA-N | TCMSP | 0.790491035 |
| LC | PLO | CYP17A1 | ORNBQBCIOKFOEO-QGVNFLHTSA-N | TCMSP | 0.790491035 |
| LC | PLO | TRPM3 | ORNBQBCIOKFOEO-QGVNFLHTSA-N | TCMSP | 0.790491035 |
| LC | PLO | CYP21A2 | ORNBQBCIOKFOEO-QGVNFLHTSA-N | TCMSP | 0.790491035 |
| LC | TML | KIF11 | GETQZCLCWQTVFV-UHFFFAOYSA-N | TCMSP | 0.344632475 |
| LC | TML | FMO3 | GETQZCLCWQTVFV-UHFFFAOYSA-N | TCMSP | 0.344632475 |
| LC | WLN: 2VR | ALDH3A1 | KRIOVPPHQSLHCZ-UHFFFAOYSA-N | TCMSP | 0.542108284 |
| LC | tetramethylpyrazine | CXCL8 | FINHMKGKINIASC-UHFFFAOYSA-N | TCMSP | 0.581527131 |
| LC | tetramethylpyrazine | HIF1A | FINHMKGKINIASC-UHFFFAOYSA-N | TCMSP | 0.581527131 |
| LC | caffeic acid | PTGS1 | QAIPRVGONGVQAS-DUXPYHPUSA-N | TCMSP | 0.592846081 |
| LC | caffeic acid | PTGS2 | QAIPRVGONGVQAS-DUXPYHPUSA-N | TCMSP | 0.592846081 |
| LC | caffeic acid | ACHE | QAIPRVGONGVQAS-DUXPYHPUSA-N | TCMSP | 0.592846081 |
| LC | caffeic acid | TNF | QAIPRVGONGVQAS-DUXPYHPUSA-N | TCMSP | 0.592846081 |
| LC | caffeic acid | SELP | QAIPRVGONGVQAS-DUXPYHPUSA-N | TCMSP | 0.592846081 |
| LC | caffeic acid | BTK | QAIPRVGONGVQAS-DUXPYHPUSA-N | TCMSP | 0.592846081 |
| LC | caffeic acid | RAC1 | QAIPRVGONGVQAS-DUXPYHPUSA-N | TCMSP | 0.592846081 |
| LC | caffeic acid | CYP1A1 | QAIPRVGONGVQAS-DUXPYHPUSA-N | TCMSP | 0.592846081 |
| LC | caffeic acid | MIF | QAIPRVGONGVQAS-DUXPYHPUSA-N | TCMSP | 0.592846081 |
| LC | caffeic acid | MAPK1 | QAIPRVGONGVQAS-DUXPYHPUSA-N | TCMSP | 0.592846081 |
| LC | caffeic acid | TYR | QAIPRVGONGVQAS-DUXPYHPUSA-N | TCMSP | 0.592846081 |
| LC | caffeic acid | MAPK8 | QAIPRVGONGVQAS-DUXPYHPUSA-N | TCMSP | 0.592846081 |
| LC | caffeic acid | COMT | QAIPRVGONGVQAS-DUXPYHPUSA-N | TCMSP | 0.592846081 |
| LC | caffeic acid | ALOX5 | QAIPRVGONGVQAS-DUXPYHPUSA-N | TCMSP | 0.592846081 |
| LC | L-Limonen | CYP2C9 | XMGQYMWWDOXHJM-SNVBAGLBSA-N | TCMSP | 0.285324274 |
| LC | L-Limonen | CYP2C19 | XMGQYMWWDOXHJM-SNVBAGLBSA-N | TCMSP | 0.285324274 |
| LC | Undekansaeure | SEC14L4 | ZDPHROOEEOARMN-UHFFFAOYSA-N | TCMSP | 0.645551231 |
| LC | vanillin | MAPK1 | MWOOGOJBHIARFG-UHFFFAOYSA-N | TCMSP | 0.732345521 |
| LC | vanillin | JUN | MWOOGOJBHIARFG-UHFFFAOYSA-N | TCMSP | 0.732345521 |
| LC | vanillin | KCNK3 | MWOOGOJBHIARFG-UHFFFAOYSA-N | TCMSP | 0.732345521 |
| LC | vanillin | TRPV3 | MWOOGOJBHIARFG-UHFFFAOYSA-N | TCMSP | 0.732345521 |
| LC | hexanal | TNF | JARKCYVAAOWBJS-UHFFFAOYSA-N | TCMSP | 0.46249322 |
| LC | hexanal | JUN | JARKCYVAAOWBJS-UHFFFAOYSA-N | TCMSP | 0.46249322 |
| LC | hexanal | MAPK10 | JARKCYVAAOWBJS-UHFFFAOYSA-N | TCMSP | 0.46249322 |
| LC | hexanal | F3 | JARKCYVAAOWBJS-UHFFFAOYSA-N | TCMSP | 0.46249322 |
| LC | hexanal | FOS | JARKCYVAAOWBJS-UHFFFAOYSA-N | TCMSP | 0.46249322 |
| LC | hexanal | CYP1A1 | JARKCYVAAOWBJS-UHFFFAOYSA-N | TCMSP | 0.46249322 |
| LC | oleic acid | NOS2 | ZQPPMHVWECSIRJ-KTKRTIGZSA-N | TCMSP | 0.385998783 |
| LC | oleic acid | PLG | ZQPPMHVWECSIRJ-KTKRTIGZSA-N | TCMSP | 0.385998783 |
| LC | oleic acid | PLAU | ZQPPMHVWECSIRJ-KTKRTIGZSA-N | TCMSP | 0.385998783 |
| LC | oleic acid | MPO | ZQPPMHVWECSIRJ-KTKRTIGZSA-N | TCMSP | 0.385998783 |
| LC | oleic acid | ENPEP | ZQPPMHVWECSIRJ-KTKRTIGZSA-N | TCMSP | 0.385998783 |
| LC | oleic acid | SOD1 | ZQPPMHVWECSIRJ-KTKRTIGZSA-N | TCMSP | 0.385998783 |
| LC | oleic acid | NTRK2 | ZQPPMHVWECSIRJ-KTKRTIGZSA-N | TCMSP | 0.385998783 |
| LC | oleic acid | INS | ZQPPMHVWECSIRJ-KTKRTIGZSA-N | TCMSP | 0.385998783 |
| LC | oleic acid | PRSS1 | ZQPPMHVWECSIRJ-KTKRTIGZSA-N | TCMSP | 0.385998783 |
| LC | oleic acid | PAM | ZQPPMHVWECSIRJ-KTKRTIGZSA-N | TCMSP | 0.385998783 |
| LC | oleic acid | DNPEP | ZQPPMHVWECSIRJ-KTKRTIGZSA-N | TCMSP | 0.385998783 |
| LC | oleic acid | ERBB2 | ZQPPMHVWECSIRJ-KTKRTIGZSA-N | TCMSP | 0.385998783 |
| LC | oleic acid | UTS2 | ZQPPMHVWECSIRJ-KTKRTIGZSA-N | TCMSP | 0.385998783 |
| LC | oleic acid | GNA11 | ZQPPMHVWECSIRJ-KTKRTIGZSA-N | TCMSP | 0.385998783 |
| LC | oleic acid | P2RY10 | ZQPPMHVWECSIRJ-KTKRTIGZSA-N | TCMSP | 0.385998783 |
| LC | oleic acid | CTSG | ZQPPMHVWECSIRJ-KTKRTIGZSA-N | TCMSP | 0.385998783 |
| LC | oleic acid | BDKRB1 | ZQPPMHVWECSIRJ-KTKRTIGZSA-N | TCMSP | 0.385998783 |
| LC | oleic acid | PROKR2 | ZQPPMHVWECSIRJ-KTKRTIGZSA-N | TCMSP | 0.385998783 |
| LC | oleic acid | OXT | ZQPPMHVWECSIRJ-KTKRTIGZSA-N | TCMSP | 0.385998783 |
| LC | oleic acid | MLNR | ZQPPMHVWECSIRJ-KTKRTIGZSA-N | TCMSP | 0.385998783 |
| LC | oleic acid | RETN | ZQPPMHVWECSIRJ-KTKRTIGZSA-N | TCMSP | 0.385998783 |
| LC | oleic acid | GNRHR | ZQPPMHVWECSIRJ-KTKRTIGZSA-N | TCMSP | 0.385998783 |
| LC | oleic acid | APOC3 | ZQPPMHVWECSIRJ-KTKRTIGZSA-N | TCMSP | 0.385998783 |
| LC | oleic acid | RBP1 | ZQPPMHVWECSIRJ-KTKRTIGZSA-N | TCMSP | 0.385998783 |
| LC | oleic acid | APOB | ZQPPMHVWECSIRJ-KTKRTIGZSA-N | TCMSP | 0.385998783 |
| LC | oleic acid | KISS1R | ZQPPMHVWECSIRJ-KTKRTIGZSA-N | TCMSP | 0.385998783 |
| LC | oleic acid | RGS2 | ZQPPMHVWECSIRJ-KTKRTIGZSA-N | TCMSP | 0.385998783 |
| LC | oleic acid | APOA1 | ZQPPMHVWECSIRJ-KTKRTIGZSA-N | TCMSP | 0.385998783 |
| LC | oleic acid | TTR | ZQPPMHVWECSIRJ-KTKRTIGZSA-N | TCMSP | 0.385998783 |
| LC | oleic acid | ABCG2 | ZQPPMHVWECSIRJ-KTKRTIGZSA-N | TCMSP | 0.385998783 |
| LC | oleic acid | ACOT2 | ZQPPMHVWECSIRJ-KTKRTIGZSA-N | TCMSP | 0.385998783 |
| LC | oleic acid | KALRN | ZQPPMHVWECSIRJ-KTKRTIGZSA-N | TCMSP | 0.385998783 |
| LC | oleic acid | GHSR | ZQPPMHVWECSIRJ-KTKRTIGZSA-N | TCMSP | 0.385998783 |
| LC | oleic acid | LRP1 | ZQPPMHVWECSIRJ-KTKRTIGZSA-N | TCMSP | 0.385998783 |
| LC | oleic acid | FAAH | ZQPPMHVWECSIRJ-KTKRTIGZSA-N | TCMSP | 0.385998783 |
| LC | oleic acid | GNRH2 | ZQPPMHVWECSIRJ-KTKRTIGZSA-N | TCMSP | 0.385998783 |
| LC | oleic acid | FFAR2 | ZQPPMHVWECSIRJ-KTKRTIGZSA-N | TCMSP | 0.385998783 |
| LC | oleic acid | FFAR1 | ZQPPMHVWECSIRJ-KTKRTIGZSA-N | TCMSP | 0.385998783 |
| LC | oleic acid | F2RL3 | ZQPPMHVWECSIRJ-KTKRTIGZSA-N | TCMSP | 0.385998783 |
| LC | oleic acid | MCHR1 | ZQPPMHVWECSIRJ-KTKRTIGZSA-N | TCMSP | 0.385998783 |
| LC | oleic acid | APOE | ZQPPMHVWECSIRJ-KTKRTIGZSA-N | TCMSP | 0.385998783 |
| LC | oleic acid | SLC27A1 | ZQPPMHVWECSIRJ-KTKRTIGZSA-N | TCMSP | 0.385998783 |
| LC | oleic acid | RARA | ZQPPMHVWECSIRJ-KTKRTIGZSA-N | TCMSP | 0.385998783 |
| LC | oleic acid | SDC1 | ZQPPMHVWECSIRJ-KTKRTIGZSA-N | TCMSP | 0.385998783 |
| LC | oleic acid | NMUR2 | ZQPPMHVWECSIRJ-KTKRTIGZSA-N | TCMSP | 0.385998783 |
| LC | oleic acid | CHRM3 | ZQPPMHVWECSIRJ-KTKRTIGZSA-N | TCMSP | 0.385998783 |
| LC | oleic acid | NTS | ZQPPMHVWECSIRJ-KTKRTIGZSA-N | TCMSP | 0.385998783 |
| LC | oleic acid | FABP4 | ZQPPMHVWECSIRJ-KTKRTIGZSA-N | TCMSP | 0.385998783 |
| LC | oleic acid | GRP | ZQPPMHVWECSIRJ-KTKRTIGZSA-N | TCMSP | 0.385998783 |
| LC | oleic acid | ANXA1 | ZQPPMHVWECSIRJ-KTKRTIGZSA-N | TCMSP | 0.385998783 |
| LC | oleic acid | NMBR | ZQPPMHVWECSIRJ-KTKRTIGZSA-N | TCMSP | 0.385998783 |
| LC | oleic acid | HTR2B | ZQPPMHVWECSIRJ-KTKRTIGZSA-N | TCMSP | 0.385998783 |
| LC | oleic acid | BAAT | ZQPPMHVWECSIRJ-KTKRTIGZSA-N | TCMSP | 0.385998783 |
| LC | oleic acid | PLCB2 | ZQPPMHVWECSIRJ-KTKRTIGZSA-N | TCMSP | 0.385998783 |
| LC | oleic acid | RPE65 | ZQPPMHVWECSIRJ-KTKRTIGZSA-N | TCMSP | 0.385998783 |
| LC | oleic acid | SLC27A6 | ZQPPMHVWECSIRJ-KTKRTIGZSA-N | TCMSP | 0.385998783 |
| LC | oleic acid | GNA15 | ZQPPMHVWECSIRJ-KTKRTIGZSA-N | TCMSP | 0.385998783 |
| LC | oleic acid | IL1A | ZQPPMHVWECSIRJ-KTKRTIGZSA-N | TCMSP | 0.385998783 |
| LC | oleic acid | LRP2 | ZQPPMHVWECSIRJ-KTKRTIGZSA-N | TCMSP | 0.385998783 |
| LC | oleic acid | PIK3CA | ZQPPMHVWECSIRJ-KTKRTIGZSA-N | TCMSP | 0.385998783 |
| LC | oleic acid | GPC1 | ZQPPMHVWECSIRJ-KTKRTIGZSA-N | TCMSP | 0.385998783 |
| LC | oleic acid | NMU | ZQPPMHVWECSIRJ-KTKRTIGZSA-N | TCMSP | 0.385998783 |
| LC | oleic acid | STAT3 | ZQPPMHVWECSIRJ-KTKRTIGZSA-N | TCMSP | 0.385998783 |
| LC | oleic acid | ICAM1 | ZQPPMHVWECSIRJ-KTKRTIGZSA-N | TCMSP | 0.385998783 |
| LC | oleic acid | PPARGC1A | ZQPPMHVWECSIRJ-KTKRTIGZSA-N | TCMSP | 0.385998783 |
| LC | oleic acid | KNG1 | ZQPPMHVWECSIRJ-KTKRTIGZSA-N | TCMSP | 0.385998783 |
| LC | oleic acid | NPFF | ZQPPMHVWECSIRJ-KTKRTIGZSA-N | TCMSP | 0.385998783 |
| LC | oleic acid | GPR65 | ZQPPMHVWECSIRJ-KTKRTIGZSA-N | TCMSP | 0.385998783 |
| LC | oleic acid | PROK1 | ZQPPMHVWECSIRJ-KTKRTIGZSA-N | TCMSP | 0.385998783 |
| LC | oleic acid | GPR17 | ZQPPMHVWECSIRJ-KTKRTIGZSA-N | TCMSP | 0.385998783 |
| LC | oleic acid | AGTR1 | ZQPPMHVWECSIRJ-KTKRTIGZSA-N | TCMSP | 0.385998783 |
| LC | oleic acid | FABP2 | ZQPPMHVWECSIRJ-KTKRTIGZSA-N | TCMSP | 0.385998783 |
| LC | oleic acid | HTR2C | ZQPPMHVWECSIRJ-KTKRTIGZSA-N | TCMSP | 0.385998783 |
| LC | oleic acid | GNRH1 | ZQPPMHVWECSIRJ-KTKRTIGZSA-N | TCMSP | 0.385998783 |
| LC | oleic acid | LCN2 | ZQPPMHVWECSIRJ-KTKRTIGZSA-N | TCMSP | 0.385998783 |
| LC | oleic acid | NPFFR1 | ZQPPMHVWECSIRJ-KTKRTIGZSA-N | TCMSP | 0.385998783 |
| LC | oleic acid | FADS2 | ZQPPMHVWECSIRJ-KTKRTIGZSA-N | TCMSP | 0.385998783 |
| LC | oleic acid | PLCB3 | ZQPPMHVWECSIRJ-KTKRTIGZSA-N | TCMSP | 0.385998783 |
| LC | oleic acid | ACSL1 | ZQPPMHVWECSIRJ-KTKRTIGZSA-N | TCMSP | 0.385998783 |
| LC | oleic acid | MCHR2 | ZQPPMHVWECSIRJ-KTKRTIGZSA-N | TCMSP | 0.385998783 |
| LC | oleic acid | CYSLTR2 | ZQPPMHVWECSIRJ-KTKRTIGZSA-N | TCMSP | 0.385998783 |
| LC | oleic acid | GRM1 | ZQPPMHVWECSIRJ-KTKRTIGZSA-N | TCMSP | 0.385998783 |
| LC | oleic acid | APP | ZQPPMHVWECSIRJ-KTKRTIGZSA-N | TCMSP | 0.385998783 |
| LC | oleic acid | GNAQ | ZQPPMHVWECSIRJ-KTKRTIGZSA-N | TCMSP | 0.385998783 |
| LC | oleic acid | ACSM3 | ZQPPMHVWECSIRJ-KTKRTIGZSA-N | TCMSP | 0.385998783 |
| LC | oleic acid | GPC2 | ZQPPMHVWECSIRJ-KTKRTIGZSA-N | TCMSP | 0.385998783 |
| LC | oleic acid | PTGER1 | ZQPPMHVWECSIRJ-KTKRTIGZSA-N | TCMSP | 0.385998783 |
| LC | oleic acid | HCRT | ZQPPMHVWECSIRJ-KTKRTIGZSA-N | TCMSP | 0.385998783 |
| LC | oleic acid | CCKAR | ZQPPMHVWECSIRJ-KTKRTIGZSA-N | TCMSP | 0.385998783 |
| LC | oleic acid | PROK2 | ZQPPMHVWECSIRJ-KTKRTIGZSA-N | TCMSP | 0.385998783 |
| LC | oleic acid | FABP1 | ZQPPMHVWECSIRJ-KTKRTIGZSA-N | TCMSP | 0.385998783 |
| LC | oleic acid | ALB | ZQPPMHVWECSIRJ-KTKRTIGZSA-N | TCMSP | 0.385998783 |
| LC | oleic acid | F2RL2 | ZQPPMHVWECSIRJ-KTKRTIGZSA-N | TCMSP | 0.385998783 |
| LC | oleic acid | F2RL1 | ZQPPMHVWECSIRJ-KTKRTIGZSA-N | TCMSP | 0.385998783 |
| LC | oleic acid | ACSL6 | ZQPPMHVWECSIRJ-KTKRTIGZSA-N | TCMSP | 0.385998783 |
| LC | oleic acid | AVPR1A | ZQPPMHVWECSIRJ-KTKRTIGZSA-N | TCMSP | 0.385998783 |
| LC | oleic acid | TAC3 | ZQPPMHVWECSIRJ-KTKRTIGZSA-N | TCMSP | 0.385998783 |
| LC | oleic acid | SLC27A4 | ZQPPMHVWECSIRJ-KTKRTIGZSA-N | TCMSP | 0.385998783 |
| LC | oleic acid | ACSM1 | ZQPPMHVWECSIRJ-KTKRTIGZSA-N | TCMSP | 0.385998783 |
| LC | oleic acid | TACR3 | ZQPPMHVWECSIRJ-KTKRTIGZSA-N | TCMSP | 0.385998783 |
| LC | oleic acid | TRH | ZQPPMHVWECSIRJ-KTKRTIGZSA-N | TCMSP | 0.385998783 |
| LC | oleic acid | TACR1 | ZQPPMHVWECSIRJ-KTKRTIGZSA-N | TCMSP | 0.385998783 |
| LC | oleic acid | LRP8 | ZQPPMHVWECSIRJ-KTKRTIGZSA-N | TCMSP | 0.385998783 |
| LC | oleic acid | NTSR2 | ZQPPMHVWECSIRJ-KTKRTIGZSA-N | TCMSP | 0.385998783 |
| LC | oleic acid | PROKR1 | ZQPPMHVWECSIRJ-KTKRTIGZSA-N | TCMSP | 0.385998783 |
| LC | oleic acid | RAC3 | ZQPPMHVWECSIRJ-KTKRTIGZSA-N | TCMSP | 0.385998783 |
| LC | oleic acid | P2RY1 | ZQPPMHVWECSIRJ-KTKRTIGZSA-N | TCMSP | 0.385998783 |
| LC | oleic acid | PRKCB | ZQPPMHVWECSIRJ-KTKRTIGZSA-N | TCMSP | 0.385998783 |
| LC | oleic acid | NMUR1 | ZQPPMHVWECSIRJ-KTKRTIGZSA-N | TCMSP | 0.385998783 |
| LC | oleic acid | GRM5 | ZQPPMHVWECSIRJ-KTKRTIGZSA-N | TCMSP | 0.385998783 |
| LC | oleic acid | CHRM1 | ZQPPMHVWECSIRJ-KTKRTIGZSA-N | TCMSP | 0.385998783 |
| LC | oleic acid | ADRA1B | ZQPPMHVWECSIRJ-KTKRTIGZSA-N | TCMSP | 0.385998783 |
| LC | oleic acid | SDC2 | ZQPPMHVWECSIRJ-KTKRTIGZSA-N | TCMSP | 0.385998783 |
| LC | oleic acid | LTB4R | ZQPPMHVWECSIRJ-KTKRTIGZSA-N | TCMSP | 0.385998783 |
| LC | oleic acid | FADS6 | ZQPPMHVWECSIRJ-KTKRTIGZSA-N | TCMSP | 0.385998783 |
| LC | oleic acid | NPFFR2 | ZQPPMHVWECSIRJ-KTKRTIGZSA-N | TCMSP | 0.385998783 |
| LC | oleic acid | PCSK1 | ZQPPMHVWECSIRJ-KTKRTIGZSA-N | TCMSP | 0.385998783 |
| LC | oleic acid | GPRC6A | ZQPPMHVWECSIRJ-KTKRTIGZSA-N | TCMSP | 0.385998783 |
| LC | oleic acid | P2RY6 | ZQPPMHVWECSIRJ-KTKRTIGZSA-N | TCMSP | 0.385998783 |
| LC | oleic acid | TRHR | ZQPPMHVWECSIRJ-KTKRTIGZSA-N | TCMSP | 0.385998783 |
| LC | oleic acid | P2RY2 | ZQPPMHVWECSIRJ-KTKRTIGZSA-N | TCMSP | 0.385998783 |
| LC | oleic acid | XCR1 | ZQPPMHVWECSIRJ-KTKRTIGZSA-N | TCMSP | 0.385998783 |
| LC | oleic acid | CASP3 | ZQPPMHVWECSIRJ-KTKRTIGZSA-N | TCMSP | 0.385998783 |
| LC | oleic acid | CYP4A11 | ZQPPMHVWECSIRJ-KTKRTIGZSA-N | TCMSP | 0.385998783 |
| LC | oleic acid | ACOT1 | ZQPPMHVWECSIRJ-KTKRTIGZSA-N | TCMSP | 0.385998783 |
| LC | oleic acid | UCP2 | ZQPPMHVWECSIRJ-KTKRTIGZSA-N | TCMSP | 0.385998783 |
| LC | oleic acid | GRK2 | ZQPPMHVWECSIRJ-KTKRTIGZSA-N | TCMSP | 0.385998783 |
| LC | oleic acid | EDNRA | ZQPPMHVWECSIRJ-KTKRTIGZSA-N | TCMSP | 0.385998783 |
| LC | oleic acid | GLTP | ZQPPMHVWECSIRJ-KTKRTIGZSA-N | TCMSP | 0.385998783 |
| LC | oleic acid | DEGS1 | ZQPPMHVWECSIRJ-KTKRTIGZSA-N | TCMSP | 0.385998783 |
| LC | oleic acid | GPR4 | ZQPPMHVWECSIRJ-KTKRTIGZSA-N | TCMSP | 0.385998783 |
| LC | oleic acid | CIDEA | ZQPPMHVWECSIRJ-KTKRTIGZSA-N | TCMSP | 0.385998783 |
| LC | oleic acid | TAC1 | ZQPPMHVWECSIRJ-KTKRTIGZSA-N | TCMSP | 0.385998783 |
| LC | oleic acid | F2R | ZQPPMHVWECSIRJ-KTKRTIGZSA-N | TCMSP | 0.385998783 |
| LC | oleic acid | ACOT4 | ZQPPMHVWECSIRJ-KTKRTIGZSA-N | TCMSP | 0.385998783 |
| LC | oleic acid | UTS2R | ZQPPMHVWECSIRJ-KTKRTIGZSA-N | TCMSP | 0.385998783 |
| LC | oleic acid | OXTR | ZQPPMHVWECSIRJ-KTKRTIGZSA-N | TCMSP | 0.385998783 |
| LC | oleic acid | LPAR5 | ZQPPMHVWECSIRJ-KTKRTIGZSA-N | TCMSP | 0.385998783 |
| LC | oleic acid | ACSM5 | ZQPPMHVWECSIRJ-KTKRTIGZSA-N | TCMSP | 0.385998783 |
| LC | oleic acid | FFAR3 | ZQPPMHVWECSIRJ-KTKRTIGZSA-N | TCMSP | 0.385998783 |
| LC | oleic acid | GPR132 | ZQPPMHVWECSIRJ-KTKRTIGZSA-N | TCMSP | 0.385998783 |
| LC | oleic acid | GAST | ZQPPMHVWECSIRJ-KTKRTIGZSA-N | TCMSP | 0.385998783 |
| LC | oleic acid | PMCH | ZQPPMHVWECSIRJ-KTKRTIGZSA-N | TCMSP | 0.385998783 |
| LC | oleic acid | RGS19 | ZQPPMHVWECSIRJ-KTKRTIGZSA-N | TCMSP | 0.385998783 |
| LC | oleic acid | GNG2 | ZQPPMHVWECSIRJ-KTKRTIGZSA-N | TCMSP | 0.385998783 |
| LC | oleic acid | CCKBR | ZQPPMHVWECSIRJ-KTKRTIGZSA-N | TCMSP | 0.385998783 |
| LC | oleic acid | ARHGEF25 | ZQPPMHVWECSIRJ-KTKRTIGZSA-N | TCMSP | 0.385998783 |
| LC | oleic acid | CCK | ZQPPMHVWECSIRJ-KTKRTIGZSA-N | TCMSP | 0.385998783 |
| LC | oleic acid | ATF4 | ZQPPMHVWECSIRJ-KTKRTIGZSA-N | TCMSP | 0.385998783 |
| LC | oleic acid | CHAT | ZQPPMHVWECSIRJ-KTKRTIGZSA-N | TCMSP | 0.385998783 |
| LC | oleic acid | EDN3 | ZQPPMHVWECSIRJ-KTKRTIGZSA-N | TCMSP | 0.385998783 |
| LC | oleic acid | LRAT | ZQPPMHVWECSIRJ-KTKRTIGZSA-N | TCMSP | 0.385998783 |
| LC | oleic acid | DCX | ZQPPMHVWECSIRJ-KTKRTIGZSA-N | TCMSP | 0.385998783 |
| LC | oleic acid | PLCB1 | ZQPPMHVWECSIRJ-KTKRTIGZSA-N | TCMSP | 0.385998783 |
| LC | oleic acid | CAV1 | ZQPPMHVWECSIRJ-KTKRTIGZSA-N | TCMSP | 0.385998783 |
| LC | oleic acid | TRIO | ZQPPMHVWECSIRJ-KTKRTIGZSA-N | TCMSP | 0.385998783 |
| LC | oleic acid | ACSL4 | ZQPPMHVWECSIRJ-KTKRTIGZSA-N | TCMSP | 0.385998783 |
| LC | oleic acid | NAPEPLD | ZQPPMHVWECSIRJ-KTKRTIGZSA-N | TCMSP | 0.385998783 |
| LC | oleic acid | FPR2 | ZQPPMHVWECSIRJ-KTKRTIGZSA-N | TCMSP | 0.385998783 |
| LC | oleic acid | ACSM6 | ZQPPMHVWECSIRJ-KTKRTIGZSA-N | TCMSP | 0.385998783 |
| LC | oleic acid | UTS2B | ZQPPMHVWECSIRJ-KTKRTIGZSA-N | TCMSP | 0.385998783 |
| LC | oleic acid | LPAR6 | ZQPPMHVWECSIRJ-KTKRTIGZSA-N | TCMSP | 0.385998783 |
| LC | oleic acid | SDC3 | ZQPPMHVWECSIRJ-KTKRTIGZSA-N | TCMSP | 0.385998783 |
| LC | oleic acid | QRFP | ZQPPMHVWECSIRJ-KTKRTIGZSA-N | TCMSP | 0.385998783 |
| LC | oleic acid | ACSL5 | ZQPPMHVWECSIRJ-KTKRTIGZSA-N | TCMSP | 0.385998783 |
| LC | oleic acid | RAC1 | ZQPPMHVWECSIRJ-KTKRTIGZSA-N | TCMSP | 0.385998783 |
| LC | oleic acid | SAA1 | ZQPPMHVWECSIRJ-KTKRTIGZSA-N | TCMSP | 0.385998783 |
| LC | oleic acid | GM2A | ZQPPMHVWECSIRJ-KTKRTIGZSA-N | TCMSP | 0.385998783 |
| LC | oleic acid | GIP | ZQPPMHVWECSIRJ-KTKRTIGZSA-N | TCMSP | 0.385998783 |
| LC | oleic acid | ACSL3 | ZQPPMHVWECSIRJ-KTKRTIGZSA-N | TCMSP | 0.385998783 |
| LC | oleic acid | LPAR1 | ZQPPMHVWECSIRJ-KTKRTIGZSA-N | TCMSP | 0.385998783 |
| LC | oleic acid | NPSR1 | ZQPPMHVWECSIRJ-KTKRTIGZSA-N | TCMSP | 0.385998783 |
| LC | oleic acid | AGT | ZQPPMHVWECSIRJ-KTKRTIGZSA-N | TCMSP | 0.385998783 |
| LC | oleic acid | KISS1 | ZQPPMHVWECSIRJ-KTKRTIGZSA-N | TCMSP | 0.385998783 |
| LC | oleic acid | RGS18 | ZQPPMHVWECSIRJ-KTKRTIGZSA-N | TCMSP | 0.385998783 |
| LC | oleic acid | XCL1 | ZQPPMHVWECSIRJ-KTKRTIGZSA-N | TCMSP | 0.385998783 |
| LC | oleic acid | XCL2 | ZQPPMHVWECSIRJ-KTKRTIGZSA-N | TCMSP | 0.385998783 |
| LC | oleic acid | APOA2 | ZQPPMHVWECSIRJ-KTKRTIGZSA-N | TCMSP | 0.385998783 |
| LC | oleic acid | FABP7 | ZQPPMHVWECSIRJ-KTKRTIGZSA-N | TCMSP | 0.385998783 |
| LC | oleic acid | NTSR1 | ZQPPMHVWECSIRJ-KTKRTIGZSA-N | TCMSP | 0.385998783 |
| LC | oleic acid | LPAR3 | ZQPPMHVWECSIRJ-KTKRTIGZSA-N | TCMSP | 0.385998783 |
| LC | oleic acid | PTGFR | ZQPPMHVWECSIRJ-KTKRTIGZSA-N | TCMSP | 0.385998783 |
| LC | oleic acid | GPC4 | ZQPPMHVWECSIRJ-KTKRTIGZSA-N | TCMSP | 0.385998783 |
| LC | oleic acid | HCRTR2 | ZQPPMHVWECSIRJ-KTKRTIGZSA-N | TCMSP | 0.385998783 |
| LC | oleic acid | RBP4 | ZQPPMHVWECSIRJ-KTKRTIGZSA-N | TCMSP | 0.385998783 |
| LC | oleic acid | FFAR4 | ZQPPMHVWECSIRJ-KTKRTIGZSA-N | TCMSP | 0.385998783 |
| LC | oleic acid | PTGDS | ZQPPMHVWECSIRJ-KTKRTIGZSA-N | TCMSP | 0.385998783 |
| LC | oleic acid | CYP4A22 | ZQPPMHVWECSIRJ-KTKRTIGZSA-N | TCMSP | 0.385998783 |
| LC | oleic acid | OPN4 | ZQPPMHVWECSIRJ-KTKRTIGZSA-N | TCMSP | 0.385998783 |
| LC | oleic acid | EDN2 | ZQPPMHVWECSIRJ-KTKRTIGZSA-N | TCMSP | 0.385998783 |
| LC | oleic acid | SDC4 | ZQPPMHVWECSIRJ-KTKRTIGZSA-N | TCMSP | 0.385998783 |
| LC | oleic acid | ANXA7 | ZQPPMHVWECSIRJ-KTKRTIGZSA-N | TCMSP | 0.385998783 |
| LC | oleic acid | CYSLTR1 | ZQPPMHVWECSIRJ-KTKRTIGZSA-N | TCMSP | 0.385998783 |
| LC | oleic acid | TACR2 | ZQPPMHVWECSIRJ-KTKRTIGZSA-N | TCMSP | 0.385998783 |
| LC | oleic acid | HCRTR1 | ZQPPMHVWECSIRJ-KTKRTIGZSA-N | TCMSP | 0.385998783 |
| LC | oleic acid | FABP3 | ZQPPMHVWECSIRJ-KTKRTIGZSA-N | TCMSP | 0.385998783 |
| LC | oleic acid | HSPG2 | ZQPPMHVWECSIRJ-KTKRTIGZSA-N | TCMSP | 0.385998783 |
| LC | oleic acid | FAAH2 | ZQPPMHVWECSIRJ-KTKRTIGZSA-N | TCMSP | 0.385998783 |
| LC | oleic acid | GNA14 | ZQPPMHVWECSIRJ-KTKRTIGZSA-N | TCMSP | 0.385998783 |
| LC | oleic acid | NMS | ZQPPMHVWECSIRJ-KTKRTIGZSA-N | TCMSP | 0.385998783 |
| LC | oleic acid | GPC6 | ZQPPMHVWECSIRJ-KTKRTIGZSA-N | TCMSP | 0.385998783 |
| LC | oleic acid | GPC5 | ZQPPMHVWECSIRJ-KTKRTIGZSA-N | TCMSP | 0.385998783 |
| LC | oleic acid | EDNRB | ZQPPMHVWECSIRJ-KTKRTIGZSA-N | TCMSP | 0.385998783 |
| LC | oleic acid | ACOT7 | ZQPPMHVWECSIRJ-KTKRTIGZSA-N | TCMSP | 0.385998783 |
| LC | oleic acid | OLAH | ZQPPMHVWECSIRJ-KTKRTIGZSA-N | TCMSP | 0.385998783 |
| LC | oleic acid | HTR2A | ZQPPMHVWECSIRJ-KTKRTIGZSA-N | TCMSP | 0.385998783 |
| LC | oleic acid | AGRN | ZQPPMHVWECSIRJ-KTKRTIGZSA-N | TCMSP | 0.385998783 |
| LC | oleic acid | ADRA1D | ZQPPMHVWECSIRJ-KTKRTIGZSA-N | TCMSP | 0.385998783 |
| LC | oleic acid | GRPR | ZQPPMHVWECSIRJ-KTKRTIGZSA-N | TCMSP | 0.385998783 |
| LC | oleic acid | AVP | ZQPPMHVWECSIRJ-KTKRTIGZSA-N | TCMSP | 0.385998783 |
| LC | oleic acid | ADRA1A | ZQPPMHVWECSIRJ-KTKRTIGZSA-N | TCMSP | 0.385998783 |
| LC | oleic acid | CHRM5 | ZQPPMHVWECSIRJ-KTKRTIGZSA-N | TCMSP | 0.385998783 |
| LC | oleic acid | GRK5 | ZQPPMHVWECSIRJ-KTKRTIGZSA-N | TCMSP | 0.385998783 |
| LC | oleic acid | GPC3 | ZQPPMHVWECSIRJ-KTKRTIGZSA-N | TCMSP | 0.385998783 |
| LC | oleic acid | QRFPR | ZQPPMHVWECSIRJ-KTKRTIGZSA-N | TCMSP | 0.385998783 |
| LC | oleic acid | NMB | ZQPPMHVWECSIRJ-KTKRTIGZSA-N | TCMSP | 0.385998783 |
| LC | oleic acid | HRH1 | ZQPPMHVWECSIRJ-KTKRTIGZSA-N | TCMSP | 0.385998783 |
| LC | oleic acid | NPS | ZQPPMHVWECSIRJ-KTKRTIGZSA-N | TCMSP | 0.385998783 |
| LC | oleic acid | ACSM4 | ZQPPMHVWECSIRJ-KTKRTIGZSA-N | TCMSP | 0.385998783 |
| LC | oleic acid | GCGR | ZQPPMHVWECSIRJ-KTKRTIGZSA-N | TCMSP | 0.385998783 |
| LC | oleic acid | LPAR2 | ZQPPMHVWECSIRJ-KTKRTIGZSA-N | TCMSP | 0.385998783 |
| LC | oleic acid | CEBPD | ZQPPMHVWECSIRJ-KTKRTIGZSA-N | TCMSP | 0.385998783 |
| LC | oleic acid | MYO7A | ZQPPMHVWECSIRJ-KTKRTIGZSA-N | TCMSP | 0.385998783 |
| LC | oleic acid | GCG | ZQPPMHVWECSIRJ-KTKRTIGZSA-N | TCMSP | 0.385998783 |
| LC | oleic acid | MLN | ZQPPMHVWECSIRJ-KTKRTIGZSA-N | TCMSP | 0.385998783 |
| LC | oleic acid | TBXA2R | ZQPPMHVWECSIRJ-KTKRTIGZSA-N | TCMSP | 0.385998783 |
| LC | oleic acid | GC | ZQPPMHVWECSIRJ-KTKRTIGZSA-N | TCMSP | 0.385998783 |
| LC | oleic acid | CEBPA | ZQPPMHVWECSIRJ-KTKRTIGZSA-N | TCMSP | 0.385998783 |
| LC | oleic acid | LTB4R2 | ZQPPMHVWECSIRJ-KTKRTIGZSA-N | TCMSP | 0.385998783 |
| LC | oleic acid | GPR68 | ZQPPMHVWECSIRJ-KTKRTIGZSA-N | TCMSP | 0.385998783 |
| LC | oleic acid | DDIT3 | ZQPPMHVWECSIRJ-KTKRTIGZSA-N | TCMSP | 0.385998783 |
| LC | oleic acid | LDLR | ZQPPMHVWECSIRJ-KTKRTIGZSA-N | TCMSP | 0.385998783 |
| LC | palmitic acid | COL1A1 | IPCSVZSSVZVIGE-UHFFFAOYSA-N | TCMSP | 0.436402482 |
| LC | palmitic acid | PCYT1A | IPCSVZSSVZVIGE-UHFFFAOYSA-N | TCMSP | 0.436402482 |
| LC | palmitic acid | IL10 | IPCSVZSSVZVIGE-UHFFFAOYSA-N | TCMSP | 0.436402482 |
| LC | palmitic acid | SLC22A5 | IPCSVZSSVZVIGE-UHFFFAOYSA-N | TCMSP | 0.436402482 |
| LC | palmitic acid | TRAPPC6A | IPCSVZSSVZVIGE-UHFFFAOYSA-N | TCMSP | 0.436402482 |
| LC | palmitic acid | UTS2 | IPCSVZSSVZVIGE-UHFFFAOYSA-N | TCMSP | 0.436402482 |
| LC | palmitic acid | GNA11 | IPCSVZSSVZVIGE-UHFFFAOYSA-N | TCMSP | 0.436402482 |
| LC | palmitic acid | P2RY10 | IPCSVZSSVZVIGE-UHFFFAOYSA-N | TCMSP | 0.436402482 |
| LC | palmitic acid | FAR2 | IPCSVZSSVZVIGE-UHFFFAOYSA-N | TCMSP | 0.436402482 |
| LC | palmitic acid | SIRT1 | IPCSVZSSVZVIGE-UHFFFAOYSA-N | TCMSP | 0.436402482 |
| LC | palmitic acid | MAPK1 | IPCSVZSSVZVIGE-UHFFFAOYSA-N | TCMSP | 0.436402482 |
| LC | palmitic acid | XBP1 | IPCSVZSSVZVIGE-UHFFFAOYSA-N | TCMSP | 0.436402482 |
| LC | palmitic acid | BDKRB1 | IPCSVZSSVZVIGE-UHFFFAOYSA-N | TCMSP | 0.436402482 |
| LC | palmitic acid | TRIB3 | IPCSVZSSVZVIGE-UHFFFAOYSA-N | TCMSP | 0.436402482 |
| LC | palmitic acid | PROKR2 | IPCSVZSSVZVIGE-UHFFFAOYSA-N | TCMSP | 0.436402482 |
| LC | palmitic acid | OXT | IPCSVZSSVZVIGE-UHFFFAOYSA-N | TCMSP | 0.436402482 |
| LC | palmitic acid | ACOT8 | IPCSVZSSVZVIGE-UHFFFAOYSA-N | TCMSP | 0.436402482 |
| LC | palmitic acid | TBL1X | IPCSVZSSVZVIGE-UHFFFAOYSA-N | TCMSP | 0.436402482 |
| LC | palmitic acid | MLNR | IPCSVZSSVZVIGE-UHFFFAOYSA-N | TCMSP | 0.436402482 |
| LC | palmitic acid | FDFT1 | IPCSVZSSVZVIGE-UHFFFAOYSA-N | TCMSP | 0.436402482 |
| LC | palmitic acid | SULT2A1 | IPCSVZSSVZVIGE-UHFFFAOYSA-N | TCMSP | 0.436402482 |
| LC | palmitic acid | NRF1 | IPCSVZSSVZVIGE-UHFFFAOYSA-N | TCMSP | 0.436402482 |
| LC | palmitic acid | RPS6KB1 | IPCSVZSSVZVIGE-UHFFFAOYSA-N | TCMSP | 0.436402482 |
| LC | palmitic acid | MED31 | IPCSVZSSVZVIGE-UHFFFAOYSA-N | TCMSP | 0.436402482 |
| LC | palmitic acid | CCL2 | IPCSVZSSVZVIGE-UHFFFAOYSA-N | TCMSP | 0.436402482 |
| LC | palmitic acid | GNRHR | IPCSVZSSVZVIGE-UHFFFAOYSA-N | TCMSP | 0.436402482 |
| LC | palmitic acid | APOA5 | IPCSVZSSVZVIGE-UHFFFAOYSA-N | TCMSP | 0.436402482 |
| LC | palmitic acid | APOC3 | IPCSVZSSVZVIGE-UHFFFAOYSA-N | TCMSP | 0.436402482 |
| LC | palmitic acid | MAPK14 | IPCSVZSSVZVIGE-UHFFFAOYSA-N | TCMSP | 0.436402482 |
| LC | palmitic acid | RBP2 | IPCSVZSSVZVIGE-UHFFFAOYSA-N | TCMSP | 0.436402482 |
| LC | palmitic acid | RBP1 | IPCSVZSSVZVIGE-UHFFFAOYSA-N | TCMSP | 0.436402482 |
| LC | palmitic acid | APOB | IPCSVZSSVZVIGE-UHFFFAOYSA-N | TCMSP | 0.436402482 |
| LC | palmitic acid | KISS1R | IPCSVZSSVZVIGE-UHFFFAOYSA-N | TCMSP | 0.436402482 |
| LC | palmitic acid | RGS2 | IPCSVZSSVZVIGE-UHFFFAOYSA-N | TCMSP | 0.436402482 |
| LC | palmitic acid | APOA1 | IPCSVZSSVZVIGE-UHFFFAOYSA-N | TCMSP | 0.436402482 |
| LC | palmitic acid | EGR1 | IPCSVZSSVZVIGE-UHFFFAOYSA-N | TCMSP | 0.436402482 |
| LC | palmitic acid | NFYB | IPCSVZSSVZVIGE-UHFFFAOYSA-N | TCMSP | 0.436402482 |
| LC | palmitic acid | KALRN | IPCSVZSSVZVIGE-UHFFFAOYSA-N | TCMSP | 0.436402482 |
| LC | palmitic acid | GHSR | IPCSVZSSVZVIGE-UHFFFAOYSA-N | TCMSP | 0.436402482 |
| LC | palmitic acid | LRP1 | IPCSVZSSVZVIGE-UHFFFAOYSA-N | TCMSP | 0.436402482 |
| LC | palmitic acid | BMP4 | IPCSVZSSVZVIGE-UHFFFAOYSA-N | TCMSP | 0.436402482 |
| LC | palmitic acid | GNRH2 | IPCSVZSSVZVIGE-UHFFFAOYSA-N | TCMSP | 0.436402482 |
| LC | palmitic acid | FFAR2 | IPCSVZSSVZVIGE-UHFFFAOYSA-N | TCMSP | 0.436402482 |
| LC | palmitic acid | FFAR1 | IPCSVZSSVZVIGE-UHFFFAOYSA-N | TCMSP | 0.436402482 |
| LC | palmitic acid | F2RL3 | IPCSVZSSVZVIGE-UHFFFAOYSA-N | TCMSP | 0.436402482 |
| LC | palmitic acid | MCHR1 | IPCSVZSSVZVIGE-UHFFFAOYSA-N | TCMSP | 0.436402482 |
| LC | palmitic acid | INS | IPCSVZSSVZVIGE-UHFFFAOYSA-N | TCMSP | 0.436402482 |
| LC | palmitic acid | MED17 | IPCSVZSSVZVIGE-UHFFFAOYSA-N | TCMSP | 0.436402482 |
| LC | palmitic acid | APOE | IPCSVZSSVZVIGE-UHFFFAOYSA-N | TCMSP | 0.436402482 |
| LC | palmitic acid | SLC27A1 | IPCSVZSSVZVIGE-UHFFFAOYSA-N | TCMSP | 0.436402482 |
| LC | palmitic acid | ACLY | IPCSVZSSVZVIGE-UHFFFAOYSA-N | TCMSP | 0.436402482 |
| LC | palmitic acid | SDC1 | IPCSVZSSVZVIGE-UHFFFAOYSA-N | TCMSP | 0.436402482 |
| LC | palmitic acid | NMUR2 | IPCSVZSSVZVIGE-UHFFFAOYSA-N | TCMSP | 0.436402482 |
| LC | palmitic acid | CHRM3 | IPCSVZSSVZVIGE-UHFFFAOYSA-N | TCMSP | 0.436402482 |
| LC | palmitic acid | MED10 | IPCSVZSSVZVIGE-UHFFFAOYSA-N | TCMSP | 0.436402482 |
| LC | palmitic acid | NTS | IPCSVZSSVZVIGE-UHFFFAOYSA-N | TCMSP | 0.436402482 |
| LC | palmitic acid | PMP2 | IPCSVZSSVZVIGE-UHFFFAOYSA-N | TCMSP | 0.436402482 |
| LC | palmitic acid | FABP4 | IPCSVZSSVZVIGE-UHFFFAOYSA-N | TCMSP | 0.436402482 |
| LC | palmitic acid | MED6 | IPCSVZSSVZVIGE-UHFFFAOYSA-N | TCMSP | 0.436402482 |
| LC | palmitic acid | GRP | IPCSVZSSVZVIGE-UHFFFAOYSA-N | TCMSP | 0.436402482 |
| LC | palmitic acid | ANXA1 | IPCSVZSSVZVIGE-UHFFFAOYSA-N | TCMSP | 0.436402482 |
| LC | palmitic acid | HRK | IPCSVZSSVZVIGE-UHFFFAOYSA-N | TCMSP | 0.436402482 |
| LC | palmitic acid | NMBR | IPCSVZSSVZVIGE-UHFFFAOYSA-N | TCMSP | 0.436402482 |
| LC | palmitic acid | HTR2B | IPCSVZSSVZVIGE-UHFFFAOYSA-N | TCMSP | 0.436402482 |
| LC | palmitic acid | MED4 | IPCSVZSSVZVIGE-UHFFFAOYSA-N | TCMSP | 0.436402482 |
| LC | palmitic acid | IL6 | IPCSVZSSVZVIGE-UHFFFAOYSA-N | TCMSP | 0.436402482 |
| LC | palmitic acid | ACSBG1 | IPCSVZSSVZVIGE-UHFFFAOYSA-N | TCMSP | 0.436402482 |
| LC | palmitic acid | BAAT | IPCSVZSSVZVIGE-UHFFFAOYSA-N | TCMSP | 0.436402482 |
| LC | palmitic acid | CLPS | IPCSVZSSVZVIGE-UHFFFAOYSA-N | TCMSP | 0.436402482 |
| LC | palmitic acid | TLR2 | IPCSVZSSVZVIGE-UHFFFAOYSA-N | TCMSP | 0.436402482 |
| LC | palmitic acid | TGS1 | IPCSVZSSVZVIGE-UHFFFAOYSA-N | TCMSP | 0.436402482 |
| LC | palmitic acid | PLCB2 | IPCSVZSSVZVIGE-UHFFFAOYSA-N | TCMSP | 0.436402482 |
| LC | palmitic acid | RORA | IPCSVZSSVZVIGE-UHFFFAOYSA-N | TCMSP | 0.436402482 |
| LC | palmitic acid | SMARCD3 | IPCSVZSSVZVIGE-UHFFFAOYSA-N | TCMSP | 0.436402482 |
| LC | palmitic acid | RPE65 | IPCSVZSSVZVIGE-UHFFFAOYSA-N | TCMSP | 0.436402482 |
| LC | palmitic acid | CREBBP | IPCSVZSSVZVIGE-UHFFFAOYSA-N | TCMSP | 0.436402482 |
| LC | palmitic acid | PPARA | IPCSVZSSVZVIGE-UHFFFAOYSA-N | TCMSP | 0.436402482 |
| LC | palmitic acid | GNA15 | IPCSVZSSVZVIGE-UHFFFAOYSA-N | TCMSP | 0.436402482 |
| LC | palmitic acid | MAPK3 | IPCSVZSSVZVIGE-UHFFFAOYSA-N | TCMSP | 0.436402482 |
| LC | palmitic acid | MED15 | IPCSVZSSVZVIGE-UHFFFAOYSA-N | TCMSP | 0.436402482 |
| LC | palmitic acid | EP300 | IPCSVZSSVZVIGE-UHFFFAOYSA-N | TCMSP | 0.436402482 |
| LC | palmitic acid | NOX4 | IPCSVZSSVZVIGE-UHFFFAOYSA-N | TCMSP | 0.436402482 |
| LC | palmitic acid | IL1B | IPCSVZSSVZVIGE-UHFFFAOYSA-N | TCMSP | 0.436402482 |
| LC | palmitic acid | MED26 | IPCSVZSSVZVIGE-UHFFFAOYSA-N | TCMSP | 0.436402482 |
| LC | palmitic acid | LRP2 | IPCSVZSSVZVIGE-UHFFFAOYSA-N | TCMSP | 0.436402482 |
| LC | palmitic acid | PIK3CA | IPCSVZSSVZVIGE-UHFFFAOYSA-N | TCMSP | 0.436402482 |
| LC | palmitic acid | GPC1 | IPCSVZSSVZVIGE-UHFFFAOYSA-N | TCMSP | 0.436402482 |
| LC | palmitic acid | AGPS | IPCSVZSSVZVIGE-UHFFFAOYSA-N | TCMSP | 0.436402482 |
| LC | palmitic acid | NMU | IPCSVZSSVZVIGE-UHFFFAOYSA-N | TCMSP | 0.436402482 |
| LC | palmitic acid | PPARGC1A | IPCSVZSSVZVIGE-UHFFFAOYSA-N | TCMSP | 0.436402482 |
| LC | palmitic acid | KNG1 | IPCSVZSSVZVIGE-UHFFFAOYSA-N | TCMSP | 0.436402482 |
| LC | palmitic acid | CASP6 | IPCSVZSSVZVIGE-UHFFFAOYSA-N | TCMSP | 0.436402482 |
| LC | palmitic acid | MED20 | IPCSVZSSVZVIGE-UHFFFAOYSA-N | TCMSP | 0.436402482 |
| LC | palmitic acid | CPT1A | IPCSVZSSVZVIGE-UHFFFAOYSA-N | TCMSP | 0.436402482 |
| LC | palmitic acid | ABCB4 | IPCSVZSSVZVIGE-UHFFFAOYSA-N | TCMSP | 0.436402482 |
| LC | palmitic acid | GLIPR1 | IPCSVZSSVZVIGE-UHFFFAOYSA-N | TCMSP | 0.436402482 |
| LC | palmitic acid | NPFF | IPCSVZSSVZVIGE-UHFFFAOYSA-N | TCMSP | 0.436402482 |
| LC | palmitic acid | GPR65 | IPCSVZSSVZVIGE-UHFFFAOYSA-N | TCMSP | 0.436402482 |
| LC | palmitic acid | SLC27A2 | IPCSVZSSVZVIGE-UHFFFAOYSA-N | TCMSP | 0.436402482 |
| LC | palmitic acid | RLBP1 | IPCSVZSSVZVIGE-UHFFFAOYSA-N | TCMSP | 0.436402482 |
| LC | palmitic acid | ITGAX | IPCSVZSSVZVIGE-UHFFFAOYSA-N | TCMSP | 0.436402482 |
| LC | palmitic acid | MED9 | IPCSVZSSVZVIGE-UHFFFAOYSA-N | TCMSP | 0.436402482 |
| LC | palmitic acid | NCOR1 | IPCSVZSSVZVIGE-UHFFFAOYSA-N | TCMSP | 0.436402482 |
| LC | palmitic acid | TP53 | IPCSVZSSVZVIGE-UHFFFAOYSA-N | TCMSP | 0.436402482 |
| LC | palmitic acid | AKT1 | IPCSVZSSVZVIGE-UHFFFAOYSA-N | TCMSP | 0.436402482 |
| LC | palmitic acid | PROK1 | IPCSVZSSVZVIGE-UHFFFAOYSA-N | TCMSP | 0.436402482 |
| LC | palmitic acid | REN | IPCSVZSSVZVIGE-UHFFFAOYSA-N | TCMSP | 0.436402482 |
| LC | palmitic acid | GPR17 | IPCSVZSSVZVIGE-UHFFFAOYSA-N | TCMSP | 0.436402482 |
| LC | palmitic acid | AGTR1 | IPCSVZSSVZVIGE-UHFFFAOYSA-N | TCMSP | 0.436402482 |
| LC | palmitic acid | FABP2 | IPCSVZSSVZVIGE-UHFFFAOYSA-N | TCMSP | 0.436402482 |
| LC | palmitic acid | HTR2C | IPCSVZSSVZVIGE-UHFFFAOYSA-N | TCMSP | 0.436402482 |
| LC | palmitic acid | GNRH1 | IPCSVZSSVZVIGE-UHFFFAOYSA-N | TCMSP | 0.436402482 |
| LC | palmitic acid | TNFRSF10B | IPCSVZSSVZVIGE-UHFFFAOYSA-N | TCMSP | 0.436402482 |
| LC | palmitic acid | PLIN2 | IPCSVZSSVZVIGE-UHFFFAOYSA-N | TCMSP | 0.436402482 |
| LC | palmitic acid | PAEP | IPCSVZSSVZVIGE-UHFFFAOYSA-N | TCMSP | 0.436402482 |
| LC | palmitic acid | NPFFR1 | IPCSVZSSVZVIGE-UHFFFAOYSA-N | TCMSP | 0.436402482 |
| LC | palmitic acid | PLCB3 | IPCSVZSSVZVIGE-UHFFFAOYSA-N | TCMSP | 0.436402482 |
| LC | palmitic acid | ACSL1 | IPCSVZSSVZVIGE-UHFFFAOYSA-N | TCMSP | 0.436402482 |
| LC | palmitic acid | MCHR2 | IPCSVZSSVZVIGE-UHFFFAOYSA-N | TCMSP | 0.436402482 |
| LC | palmitic acid | MED13L | IPCSVZSSVZVIGE-UHFFFAOYSA-N | TCMSP | 0.436402482 |
| LC | palmitic acid | CYSLTR2 | IPCSVZSSVZVIGE-UHFFFAOYSA-N | TCMSP | 0.436402482 |
| LC | palmitic acid | YAP1 | IPCSVZSSVZVIGE-UHFFFAOYSA-N | TCMSP | 0.436402482 |
| LC | palmitic acid | GRM1 | IPCSVZSSVZVIGE-UHFFFAOYSA-N | TCMSP | 0.436402482 |
| LC | palmitic acid | MED21 | IPCSVZSSVZVIGE-UHFFFAOYSA-N | TCMSP | 0.436402482 |
| LC | palmitic acid | APP | IPCSVZSSVZVIGE-UHFFFAOYSA-N | TCMSP | 0.436402482 |
| LC | palmitic acid | GNAQ | IPCSVZSSVZVIGE-UHFFFAOYSA-N | TCMSP | 0.436402482 |
| LC | palmitic acid | PPARG | IPCSVZSSVZVIGE-UHFFFAOYSA-N | TCMSP | 0.436402482 |
| LC | palmitic acid | HMGCR | IPCSVZSSVZVIGE-UHFFFAOYSA-N | TCMSP | 0.436402482 |
| LC | palmitic acid | MED8 | IPCSVZSSVZVIGE-UHFFFAOYSA-N | TCMSP | 0.436402482 |
| LC | palmitic acid | MED27 | IPCSVZSSVZVIGE-UHFFFAOYSA-N | TCMSP | 0.436402482 |
| LC | palmitic acid | GPC2 | IPCSVZSSVZVIGE-UHFFFAOYSA-N | TCMSP | 0.436402482 |
| LC | palmitic acid | PTGER1 | IPCSVZSSVZVIGE-UHFFFAOYSA-N | TCMSP | 0.436402482 |
| LC | palmitic acid | LTC4S | IPCSVZSSVZVIGE-UHFFFAOYSA-N | TCMSP | 0.436402482 |
| LC | palmitic acid | HCRT | IPCSVZSSVZVIGE-UHFFFAOYSA-N | TCMSP | 0.436402482 |
| LC | palmitic acid | MED11 | IPCSVZSSVZVIGE-UHFFFAOYSA-N | TCMSP | 0.436402482 |
| LC | palmitic acid | CCKAR | IPCSVZSSVZVIGE-UHFFFAOYSA-N | TCMSP | 0.436402482 |
| LC | palmitic acid | PROK2 | IPCSVZSSVZVIGE-UHFFFAOYSA-N | TCMSP | 0.436402482 |
| LC | palmitic acid | FABP1 | IPCSVZSSVZVIGE-UHFFFAOYSA-N | TCMSP | 0.436402482 |
| LC | palmitic acid | ALB | IPCSVZSSVZVIGE-UHFFFAOYSA-N | TCMSP | 0.436402482 |
| LC | palmitic acid | RHO | IPCSVZSSVZVIGE-UHFFFAOYSA-N | TCMSP | 0.436402482 |
| LC | palmitic acid | F2RL2 | IPCSVZSSVZVIGE-UHFFFAOYSA-N | TCMSP | 0.436402482 |
| LC | palmitic acid | F2RL1 | IPCSVZSSVZVIGE-UHFFFAOYSA-N | TCMSP | 0.436402482 |
| LC | palmitic acid | TNFRSF21 | IPCSVZSSVZVIGE-UHFFFAOYSA-N | TCMSP | 0.436402482 |
| LC | palmitic acid | ACSL6 | IPCSVZSSVZVIGE-UHFFFAOYSA-N | TCMSP | 0.436402482 |
| LC | palmitic acid | FABP5 | IPCSVZSSVZVIGE-UHFFFAOYSA-N | TCMSP | 0.436402482 |
| LC | palmitic acid | MED30 | IPCSVZSSVZVIGE-UHFFFAOYSA-N | TCMSP | 0.436402482 |
| LC | palmitic acid | NOS3 | IPCSVZSSVZVIGE-UHFFFAOYSA-N | TCMSP | 0.436402482 |
| LC | palmitic acid | INPPL1 | IPCSVZSSVZVIGE-UHFFFAOYSA-N | TCMSP | 0.436402482 |
| LC | palmitic acid | AVPR1A | IPCSVZSSVZVIGE-UHFFFAOYSA-N | TCMSP | 0.436402482 |
| LC | palmitic acid | CLEC4E | IPCSVZSSVZVIGE-UHFFFAOYSA-N | TCMSP | 0.436402482 |
| LC | palmitic acid | PEX11A | IPCSVZSSVZVIGE-UHFFFAOYSA-N | TCMSP | 0.436402482 |
| LC | palmitic acid | TAC3 | IPCSVZSSVZVIGE-UHFFFAOYSA-N | TCMSP | 0.436402482 |
| LC | palmitic acid | MED1 | IPCSVZSSVZVIGE-UHFFFAOYSA-N | TCMSP | 0.436402482 |
| LC | palmitic acid | LALBA | IPCSVZSSVZVIGE-UHFFFAOYSA-N | TCMSP | 0.436402482 |
| LC | palmitic acid | ANGPTL4 | IPCSVZSSVZVIGE-UHFFFAOYSA-N | TCMSP | 0.436402482 |
| LC | palmitic acid | CYP7A1 | IPCSVZSSVZVIGE-UHFFFAOYSA-N | TCMSP | 0.436402482 |
| LC | palmitic acid | IGF1 | IPCSVZSSVZVIGE-UHFFFAOYSA-N | TCMSP | 0.436402482 |
| LC | palmitic acid | HDAC3 | IPCSVZSSVZVIGE-UHFFFAOYSA-N | TCMSP | 0.436402482 |
| LC | palmitic acid | RGL1 | IPCSVZSSVZVIGE-UHFFFAOYSA-N | TCMSP | 0.436402482 |
| LC | palmitic acid | TACR3 | IPCSVZSSVZVIGE-UHFFFAOYSA-N | TCMSP | 0.436402482 |
| LC | palmitic acid | TRH | IPCSVZSSVZVIGE-UHFFFAOYSA-N | TCMSP | 0.436402482 |
| LC | palmitic acid | TACR1 | IPCSVZSSVZVIGE-UHFFFAOYSA-N | TCMSP | 0.436402482 |
| LC | palmitic acid | LRP8 | IPCSVZSSVZVIGE-UHFFFAOYSA-N | TCMSP | 0.436402482 |
| LC | palmitic acid | NTSR2 | IPCSVZSSVZVIGE-UHFFFAOYSA-N | TCMSP | 0.436402482 |
| LC | palmitic acid | PROKR1 | IPCSVZSSVZVIGE-UHFFFAOYSA-N | TCMSP | 0.436402482 |
| LC | palmitic acid | FASN | IPCSVZSSVZVIGE-UHFFFAOYSA-N | TCMSP | 0.436402482 |
| LC | palmitic acid | P2RY1 | IPCSVZSSVZVIGE-UHFFFAOYSA-N | TCMSP | 0.436402482 |
| LC | palmitic acid | IRS1 | IPCSVZSSVZVIGE-UHFFFAOYSA-N | TCMSP | 0.436402482 |
| LC | palmitic acid | NMUR1 | IPCSVZSSVZVIGE-UHFFFAOYSA-N | TCMSP | 0.436402482 |
| LC | palmitic acid | GRM5 | IPCSVZSSVZVIGE-UHFFFAOYSA-N | TCMSP | 0.436402482 |
| LC | palmitic acid | CHRM1 | IPCSVZSSVZVIGE-UHFFFAOYSA-N | TCMSP | 0.436402482 |
| LC | palmitic acid | CXCL8 | IPCSVZSSVZVIGE-UHFFFAOYSA-N | TCMSP | 0.436402482 |
| LC | palmitic acid | ADRA1B | IPCSVZSSVZVIGE-UHFFFAOYSA-N | TCMSP | 0.436402482 |
| LC | palmitic acid | SDC2 | IPCSVZSSVZVIGE-UHFFFAOYSA-N | TCMSP | 0.436402482 |
| LC | palmitic acid | LTB4R | IPCSVZSSVZVIGE-UHFFFAOYSA-N | TCMSP | 0.436402482 |
| LC | palmitic acid | CYCS | IPCSVZSSVZVIGE-UHFFFAOYSA-N | TCMSP | 0.436402482 |
| LC | palmitic acid | NPFFR2 | IPCSVZSSVZVIGE-UHFFFAOYSA-N | TCMSP | 0.436402482 |
| LC | palmitic acid | PCSK1 | IPCSVZSSVZVIGE-UHFFFAOYSA-N | TCMSP | 0.436402482 |
| LC | palmitic acid | CD36 | IPCSVZSSVZVIGE-UHFFFAOYSA-N | TCMSP | 0.436402482 |
| LC | palmitic acid | CLOCK | IPCSVZSSVZVIGE-UHFFFAOYSA-N | TCMSP | 0.436402482 |
| LC | palmitic acid | ALAS1 | IPCSVZSSVZVIGE-UHFFFAOYSA-N | TCMSP | 0.436402482 |
| LC | palmitic acid | GPRC6A | IPCSVZSSVZVIGE-UHFFFAOYSA-N | TCMSP | 0.436402482 |
| LC | palmitic acid | P2RY6 | IPCSVZSSVZVIGE-UHFFFAOYSA-N | TCMSP | 0.436402482 |
| LC | palmitic acid | TRHR | IPCSVZSSVZVIGE-UHFFFAOYSA-N | TCMSP | 0.436402482 |
| LC | palmitic acid | P2RY2 | IPCSVZSSVZVIGE-UHFFFAOYSA-N | TCMSP | 0.436402482 |
| LC | palmitic acid | XCR1 | IPCSVZSSVZVIGE-UHFFFAOYSA-N | TCMSP | 0.436402482 |
| LC | palmitic acid | TEAD2 | IPCSVZSSVZVIGE-UHFFFAOYSA-N | TCMSP | 0.436402482 |
| LC | palmitic acid | CYP4A11 | IPCSVZSSVZVIGE-UHFFFAOYSA-N | TCMSP | 0.436402482 |
| LC | palmitic acid | ACOT1 | IPCSVZSSVZVIGE-UHFFFAOYSA-N | TCMSP | 0.436402482 |
| LC | palmitic acid | CPT1B | IPCSVZSSVZVIGE-UHFFFAOYSA-N | TCMSP | 0.436402482 |
| LC | palmitic acid | GRK2 | IPCSVZSSVZVIGE-UHFFFAOYSA-N | TCMSP | 0.436402482 |
| LC | palmitic acid | PLA2G1B | IPCSVZSSVZVIGE-UHFFFAOYSA-N | TCMSP | 0.436402482 |
| LC | palmitic acid | PPARGC1B | IPCSVZSSVZVIGE-UHFFFAOYSA-N | TCMSP | 0.436402482 |
| LC | palmitic acid | MED29 | IPCSVZSSVZVIGE-UHFFFAOYSA-N | TCMSP | 0.436402482 |
| LC | palmitic acid | EDNRA | IPCSVZSSVZVIGE-UHFFFAOYSA-N | TCMSP | 0.436402482 |
| LC | palmitic acid | DEGS1 | IPCSVZSSVZVIGE-UHFFFAOYSA-N | TCMSP | 0.436402482 |
| LC | palmitic acid | CES2 | IPCSVZSSVZVIGE-UHFFFAOYSA-N | TCMSP | 0.436402482 |
| LC | palmitic acid | GPR4 | IPCSVZSSVZVIGE-UHFFFAOYSA-N | TCMSP | 0.436402482 |
| LC | palmitic acid | LYPLA1 | IPCSVZSSVZVIGE-UHFFFAOYSA-N | TCMSP | 0.436402482 |
| LC | palmitic acid | ADIPOQ | IPCSVZSSVZVIGE-UHFFFAOYSA-N | TCMSP | 0.436402482 |
| LC | palmitic acid | NCOA1 | IPCSVZSSVZVIGE-UHFFFAOYSA-N | TCMSP | 0.436402482 |
| LC | palmitic acid | TAC1 | IPCSVZSSVZVIGE-UHFFFAOYSA-N | TCMSP | 0.436402482 |
| LC | palmitic acid | F2R | IPCSVZSSVZVIGE-UHFFFAOYSA-N | TCMSP | 0.436402482 |
| LC | palmitic acid | FADS1 | IPCSVZSSVZVIGE-UHFFFAOYSA-N | TCMSP | 0.436402482 |
| LC | palmitic acid | HMGCS1 | IPCSVZSSVZVIGE-UHFFFAOYSA-N | TCMSP | 0.436402482 |
| LC | palmitic acid | FHL2 | IPCSVZSSVZVIGE-UHFFFAOYSA-N | TCMSP | 0.436402482 |
| LC | palmitic acid | ACOT4 | IPCSVZSSVZVIGE-UHFFFAOYSA-N | TCMSP | 0.436402482 |
| LC | palmitic acid | UTS2R | IPCSVZSSVZVIGE-UHFFFAOYSA-N | TCMSP | 0.436402482 |
| LC | palmitic acid | SLC2A2 | IPCSVZSSVZVIGE-UHFFFAOYSA-N | TCMSP | 0.436402482 |
| LC | palmitic acid | MED14 | IPCSVZSSVZVIGE-UHFFFAOYSA-N | TCMSP | 0.436402482 |
| LC | palmitic acid | OXTR | IPCSVZSSVZVIGE-UHFFFAOYSA-N | TCMSP | 0.436402482 |
| LC | palmitic acid | CYP2B6 | IPCSVZSSVZVIGE-UHFFFAOYSA-N | TCMSP | 0.436402482 |
| LC | palmitic acid | GRHL1 | IPCSVZSSVZVIGE-UHFFFAOYSA-N | TCMSP | 0.436402482 |
| LC | palmitic acid | MED16 | IPCSVZSSVZVIGE-UHFFFAOYSA-N | TCMSP | 0.436402482 |
| LC | palmitic acid | CARM1 | IPCSVZSSVZVIGE-UHFFFAOYSA-N | TCMSP | 0.436402482 |
| LC | palmitic acid | MED25 | IPCSVZSSVZVIGE-UHFFFAOYSA-N | TCMSP | 0.436402482 |
| LC | palmitic acid | NOS2 | IPCSVZSSVZVIGE-UHFFFAOYSA-N | TCMSP | 0.436402482 |
| LC | palmitic acid | TIAM2 | IPCSVZSSVZVIGE-UHFFFAOYSA-N | TCMSP | 0.436402482 |
| LC | palmitic acid | LPAR5 | IPCSVZSSVZVIGE-UHFFFAOYSA-N | TCMSP | 0.436402482 |
| LC | palmitic acid | FFAR3 | IPCSVZSSVZVIGE-UHFFFAOYSA-N | TCMSP | 0.436402482 |
| LC | palmitic acid | GPR132 | IPCSVZSSVZVIGE-UHFFFAOYSA-N | TCMSP | 0.436402482 |
| LC | palmitic acid | SP1 | IPCSVZSSVZVIGE-UHFFFAOYSA-N | TCMSP | 0.436402482 |
| LC | palmitic acid | TRAPPC6B | IPCSVZSSVZVIGE-UHFFFAOYSA-N | TCMSP | 0.436402482 |
| LC | palmitic acid | PLB1 | IPCSVZSSVZVIGE-UHFFFAOYSA-N | TCMSP | 0.436402482 |
| LC | palmitic acid | GAST | IPCSVZSSVZVIGE-UHFFFAOYSA-N | TCMSP | 0.436402482 |
| LC | palmitic acid | PMCH | IPCSVZSSVZVIGE-UHFFFAOYSA-N | TCMSP | 0.436402482 |
| LC | palmitic acid | RGS19 | IPCSVZSSVZVIGE-UHFFFAOYSA-N | TCMSP | 0.436402482 |
| LC | palmitic acid | GNG2 | IPCSVZSSVZVIGE-UHFFFAOYSA-N | TCMSP | 0.436402482 |
| LC | palmitic acid | CCKBR | IPCSVZSSVZVIGE-UHFFFAOYSA-N | TCMSP | 0.436402482 |
| LC | palmitic acid | ARHGEF25 | IPCSVZSSVZVIGE-UHFFFAOYSA-N | TCMSP | 0.436402482 |
| LC | palmitic acid | CCK | IPCSVZSSVZVIGE-UHFFFAOYSA-N | TCMSP | 0.436402482 |
| LC | palmitic acid | ATF4 | IPCSVZSSVZVIGE-UHFFFAOYSA-N | TCMSP | 0.436402482 |
| LC | palmitic acid | EDN3 | IPCSVZSSVZVIGE-UHFFFAOYSA-N | TCMSP | 0.436402482 |
| LC | palmitic acid | LRAT | IPCSVZSSVZVIGE-UHFFFAOYSA-N | TCMSP | 0.436402482 |
| LC | palmitic acid | MED19 | IPCSVZSSVZVIGE-UHFFFAOYSA-N | TCMSP | 0.436402482 |
| LC | palmitic acid | HIF1A | IPCSVZSSVZVIGE-UHFFFAOYSA-N | TCMSP | 0.436402482 |
| LC | palmitic acid | PLCB1 | IPCSVZSSVZVIGE-UHFFFAOYSA-N | TCMSP | 0.436402482 |
| LC | palmitic acid | NPAS2 | IPCSVZSSVZVIGE-UHFFFAOYSA-N | TCMSP | 0.436402482 |
| LC | palmitic acid | TRIO | IPCSVZSSVZVIGE-UHFFFAOYSA-N | TCMSP | 0.436402482 |
| LC | palmitic acid | FOXO3 | IPCSVZSSVZVIGE-UHFFFAOYSA-N | TCMSP | 0.436402482 |
| LC | palmitic acid | ACSL4 | IPCSVZSSVZVIGE-UHFFFAOYSA-N | TCMSP | 0.436402482 |
| LC | palmitic acid | FPR2 | IPCSVZSSVZVIGE-UHFFFAOYSA-N | TCMSP | 0.436402482 |
| LC | palmitic acid | UTS2B | IPCSVZSSVZVIGE-UHFFFAOYSA-N | TCMSP | 0.436402482 |
| LC | palmitic acid | MED22 | IPCSVZSSVZVIGE-UHFFFAOYSA-N | TCMSP | 0.436402482 |
| LC | palmitic acid | PTGES | IPCSVZSSVZVIGE-UHFFFAOYSA-N | TCMSP | 0.436402482 |
| LC | palmitic acid | LPAR6 | IPCSVZSSVZVIGE-UHFFFAOYSA-N | TCMSP | 0.436402482 |
| LC | palmitic acid | CTNNB1 | IPCSVZSSVZVIGE-UHFFFAOYSA-N | TCMSP | 0.436402482 |
| LC | palmitic acid | SDC3 | IPCSVZSSVZVIGE-UHFFFAOYSA-N | TCMSP | 0.436402482 |
| LC | palmitic acid | QRFP | IPCSVZSSVZVIGE-UHFFFAOYSA-N | TCMSP | 0.436402482 |
| LC | palmitic acid | NFYA | IPCSVZSSVZVIGE-UHFFFAOYSA-N | TCMSP | 0.436402482 |
| LC | palmitic acid | TEAD3 | IPCSVZSSVZVIGE-UHFFFAOYSA-N | TCMSP | 0.436402482 |
| LC | palmitic acid | GPX4 | IPCSVZSSVZVIGE-UHFFFAOYSA-N | TCMSP | 0.436402482 |
| LC | palmitic acid | UGT1A9 | IPCSVZSSVZVIGE-UHFFFAOYSA-N | TCMSP | 0.436402482 |
| LC | palmitic acid | FAR1 | IPCSVZSSVZVIGE-UHFFFAOYSA-N | TCMSP | 0.436402482 |
| LC | palmitic acid | SREBF1 | IPCSVZSSVZVIGE-UHFFFAOYSA-N | TCMSP | 0.436402482 |
| LC | palmitic acid | ACSL5 | IPCSVZSSVZVIGE-UHFFFAOYSA-N | TCMSP | 0.436402482 |
| LC | palmitic acid | SAA1 | IPCSVZSSVZVIGE-UHFFFAOYSA-N | TCMSP | 0.436402482 |
| LC | palmitic acid | GIP | IPCSVZSSVZVIGE-UHFFFAOYSA-N | TCMSP | 0.436402482 |
| LC | palmitic acid | ACSL3 | IPCSVZSSVZVIGE-UHFFFAOYSA-N | TCMSP | 0.436402482 |
| LC | palmitic acid | LPAR1 | IPCSVZSSVZVIGE-UHFFFAOYSA-N | TCMSP | 0.436402482 |
| LC | palmitic acid | PTPA | IPCSVZSSVZVIGE-UHFFFAOYSA-N | TCMSP | 0.436402482 |
| LC | palmitic acid | NCOA6 | IPCSVZSSVZVIGE-UHFFFAOYSA-N | TCMSP | 0.436402482 |
| LC | palmitic acid | NPSR1 | IPCSVZSSVZVIGE-UHFFFAOYSA-N | TCMSP | 0.436402482 |
| LC | palmitic acid | NR4A1 | IPCSVZSSVZVIGE-UHFFFAOYSA-N | TCMSP | 0.436402482 |
| LC | palmitic acid | MAPK8 | IPCSVZSSVZVIGE-UHFFFAOYSA-N | TCMSP | 0.436402482 |
| LC | palmitic acid | SIN3A | IPCSVZSSVZVIGE-UHFFFAOYSA-N | TCMSP | 0.436402482 |
| LC | palmitic acid | CES1 | IPCSVZSSVZVIGE-UHFFFAOYSA-N | TCMSP | 0.436402482 |
| LC | palmitic acid | WWTR1 | IPCSVZSSVZVIGE-UHFFFAOYSA-N | TCMSP | 0.436402482 |
| LC | palmitic acid | SREBF2 | IPCSVZSSVZVIGE-UHFFFAOYSA-N | TCMSP | 0.436402482 |
| LC | palmitic acid | MTOR | IPCSVZSSVZVIGE-UHFFFAOYSA-N | TCMSP | 0.436402482 |
| LC | palmitic acid | GPX6 | IPCSVZSSVZVIGE-UHFFFAOYSA-N | TCMSP | 0.436402482 |
| LC | palmitic acid | PPT2 | IPCSVZSSVZVIGE-UHFFFAOYSA-N | TCMSP | 0.436402482 |
| LC | palmitic acid | GNPAT | IPCSVZSSVZVIGE-UHFFFAOYSA-N | TCMSP | 0.436402482 |
| LC | palmitic acid | AGT | IPCSVZSSVZVIGE-UHFFFAOYSA-N | TCMSP | 0.436402482 |
| LC | palmitic acid | COG2 | IPCSVZSSVZVIGE-UHFFFAOYSA-N | TCMSP | 0.436402482 |
| LC | palmitic acid | G0S2 | IPCSVZSSVZVIGE-UHFFFAOYSA-N | TCMSP | 0.436402482 |
| LC | palmitic acid | KISS1 | IPCSVZSSVZVIGE-UHFFFAOYSA-N | TCMSP | 0.436402482 |
| LC | palmitic acid | RGS18 | IPCSVZSSVZVIGE-UHFFFAOYSA-N | TCMSP | 0.436402482 |
| LC | palmitic acid | XCL1 | IPCSVZSSVZVIGE-UHFFFAOYSA-N | TCMSP | 0.436402482 |
| LC | palmitic acid | XCL2 | IPCSVZSSVZVIGE-UHFFFAOYSA-N | TCMSP | 0.436402482 |
| LC | palmitic acid | ATF6 | IPCSVZSSVZVIGE-UHFFFAOYSA-N | TCMSP | 0.436402482 |
| LC | palmitic acid | CTGF | IPCSVZSSVZVIGE-UHFFFAOYSA-N | TCMSP | 0.436402482 |
| LC | palmitic acid | APOA2 | IPCSVZSSVZVIGE-UHFFFAOYSA-N | TCMSP | 0.436402482 |
| LC | palmitic acid | MED23 | IPCSVZSSVZVIGE-UHFFFAOYSA-N | TCMSP | 0.436402482 |
| LC | palmitic acid | CDK19 | IPCSVZSSVZVIGE-UHFFFAOYSA-N | TCMSP | 0.436402482 |
| LC | palmitic acid | PNLIP | IPCSVZSSVZVIGE-UHFFFAOYSA-N | TCMSP | 0.436402482 |
| LC | palmitic acid | HMGCS2 | IPCSVZSSVZVIGE-UHFFFAOYSA-N | TCMSP | 0.436402482 |
| LC | palmitic acid | ME1 | IPCSVZSSVZVIGE-UHFFFAOYSA-N | TCMSP | 0.436402482 |
| LC | palmitic acid | SCD | IPCSVZSSVZVIGE-UHFFFAOYSA-N | TCMSP | 0.436402482 |
| LC | palmitic acid | NTSR1 | IPCSVZSSVZVIGE-UHFFFAOYSA-N | TCMSP | 0.436402482 |
| LC | palmitic acid | LPAR3 | IPCSVZSSVZVIGE-UHFFFAOYSA-N | TCMSP | 0.436402482 |
| LC | palmitic acid | PTGFR | IPCSVZSSVZVIGE-UHFFFAOYSA-N | TCMSP | 0.436402482 |
| LC | palmitic acid | GPC4 | IPCSVZSSVZVIGE-UHFFFAOYSA-N | TCMSP | 0.436402482 |
| LC | palmitic acid | HCRTR2 | IPCSVZSSVZVIGE-UHFFFAOYSA-N | TCMSP | 0.436402482 |
| LC | palmitic acid | CYP2C8 | IPCSVZSSVZVIGE-UHFFFAOYSA-N | TCMSP | 0.436402482 |
| LC | palmitic acid | FFAR4 | IPCSVZSSVZVIGE-UHFFFAOYSA-N | TCMSP | 0.436402482 |
| LC | palmitic acid | CPT2 | IPCSVZSSVZVIGE-UHFFFAOYSA-N | TCMSP | 0.436402482 |
| LC | palmitic acid | PTPN1 | IPCSVZSSVZVIGE-UHFFFAOYSA-N | TCMSP | 0.436402482 |
| LC | palmitic acid | PTGDS | IPCSVZSSVZVIGE-UHFFFAOYSA-N | TCMSP | 0.436402482 |
| LC | palmitic acid | ANKRD1 | IPCSVZSSVZVIGE-UHFFFAOYSA-N | TCMSP | 0.436402482 |
| LC | palmitic acid | PTEN | IPCSVZSSVZVIGE-UHFFFAOYSA-N | TCMSP | 0.436402482 |
| LC | palmitic acid | NCOA3 | IPCSVZSSVZVIGE-UHFFFAOYSA-N | TCMSP | 0.436402482 |
| LC | palmitic acid | OPN4 | IPCSVZSSVZVIGE-UHFFFAOYSA-N | TCMSP | 0.436402482 |
| LC | palmitic acid | EDN2 | IPCSVZSSVZVIGE-UHFFFAOYSA-N | TCMSP | 0.436402482 |
| LC | palmitic acid | SDC4 | IPCSVZSSVZVIGE-UHFFFAOYSA-N | TCMSP | 0.436402482 |
| LC | palmitic acid | TRAPPC3 | IPCSVZSSVZVIGE-UHFFFAOYSA-N | TCMSP | 0.436402482 |
| LC | palmitic acid | GLP1R | IPCSVZSSVZVIGE-UHFFFAOYSA-N | TCMSP | 0.436402482 |
| LC | palmitic acid | CYSLTR1 | IPCSVZSSVZVIGE-UHFFFAOYSA-N | TCMSP | 0.436402482 |
| LC | palmitic acid | TACR2 | IPCSVZSSVZVIGE-UHFFFAOYSA-N | TCMSP | 0.436402482 |
| LC | palmitic acid | HCRTR1 | IPCSVZSSVZVIGE-UHFFFAOYSA-N | TCMSP | 0.436402482 |
| LC | palmitic acid | FABP3 | IPCSVZSSVZVIGE-UHFFFAOYSA-N | TCMSP | 0.436402482 |
| LC | palmitic acid | MED18 | IPCSVZSSVZVIGE-UHFFFAOYSA-N | TCMSP | 0.436402482 |
| LC | palmitic acid | TLR4 | IPCSVZSSVZVIGE-UHFFFAOYSA-N | TCMSP | 0.436402482 |
| LC | palmitic acid | MED12 | IPCSVZSSVZVIGE-UHFFFAOYSA-N | TCMSP | 0.436402482 |
| LC | palmitic acid | UGCG | IPCSVZSSVZVIGE-UHFFFAOYSA-N | TCMSP | 0.436402482 |
| LC | palmitic acid | HSPG2 | IPCSVZSSVZVIGE-UHFFFAOYSA-N | TCMSP | 0.436402482 |
| LC | palmitic acid | SLC44A1 | IPCSVZSSVZVIGE-UHFFFAOYSA-N | TCMSP | 0.436402482 |
| LC | palmitic acid | ABCA1 | IPCSVZSSVZVIGE-UHFFFAOYSA-N | TCMSP | 0.436402482 |
| LC | palmitic acid | GNA14 | IPCSVZSSVZVIGE-UHFFFAOYSA-N | TCMSP | 0.436402482 |
| LC | palmitic acid | NMS | IPCSVZSSVZVIGE-UHFFFAOYSA-N | TCMSP | 0.436402482 |
| LC | palmitic acid | GPC6 | IPCSVZSSVZVIGE-UHFFFAOYSA-N | TCMSP | 0.436402482 |
| LC | palmitic acid | GPC5 | IPCSVZSSVZVIGE-UHFFFAOYSA-N | TCMSP | 0.436402482 |
| LC | palmitic acid | EDNRB | IPCSVZSSVZVIGE-UHFFFAOYSA-N | TCMSP | 0.436402482 |
| LC | palmitic acid | ACOT7 | IPCSVZSSVZVIGE-UHFFFAOYSA-N | TCMSP | 0.436402482 |
| LC | palmitic acid | CASK | IPCSVZSSVZVIGE-UHFFFAOYSA-N | TCMSP | 0.436402482 |
| LC | palmitic acid | ACBD7 | IPCSVZSSVZVIGE-UHFFFAOYSA-N | TCMSP | 0.436402482 |
| LC | palmitic acid | OLAH | IPCSVZSSVZVIGE-UHFFFAOYSA-N | TCMSP | 0.436402482 |
| LC | palmitic acid | HTR2A | IPCSVZSSVZVIGE-UHFFFAOYSA-N | TCMSP | 0.436402482 |
| LC | palmitic acid | AGRN | IPCSVZSSVZVIGE-UHFFFAOYSA-N | TCMSP | 0.436402482 |
| LC | palmitic acid | EDN1 | IPCSVZSSVZVIGE-UHFFFAOYSA-N | TCMSP | 0.436402482 |
| LC | palmitic acid | ADRA1D | IPCSVZSSVZVIGE-UHFFFAOYSA-N | TCMSP | 0.436402482 |
| LC | palmitic acid | CYP1A1 | IPCSVZSSVZVIGE-UHFFFAOYSA-N | TCMSP | 0.436402482 |
| LC | palmitic acid | PDHA1 | IPCSVZSSVZVIGE-UHFFFAOYSA-N | TCMSP | 0.436402482 |
| LC | palmitic acid | GRPR | IPCSVZSSVZVIGE-UHFFFAOYSA-N | TCMSP | 0.436402482 |
| LC | palmitic acid | AVP | IPCSVZSSVZVIGE-UHFFFAOYSA-N | TCMSP | 0.436402482 |
| LC | palmitic acid | ADRA1A | IPCSVZSSVZVIGE-UHFFFAOYSA-N | TCMSP | 0.436402482 |
| LC | palmitic acid | PDX1 | IPCSVZSSVZVIGE-UHFFFAOYSA-N | TCMSP | 0.436402482 |
| LC | palmitic acid | CDK8 | IPCSVZSSVZVIGE-UHFFFAOYSA-N | TCMSP | 0.436402482 |
| LC | palmitic acid | CHRM5 | IPCSVZSSVZVIGE-UHFFFAOYSA-N | TCMSP | 0.436402482 |
| LC | palmitic acid | GPX3 | IPCSVZSSVZVIGE-UHFFFAOYSA-N | TCMSP | 0.436402482 |
| LC | palmitic acid | GPX2 | IPCSVZSSVZVIGE-UHFFFAOYSA-N | TCMSP | 0.436402482 |
| LC | palmitic acid | ARNTL | IPCSVZSSVZVIGE-UHFFFAOYSA-N | TCMSP | 0.436402482 |
| LC | palmitic acid | GRK5 | IPCSVZSSVZVIGE-UHFFFAOYSA-N | TCMSP | 0.436402482 |
| LC | palmitic acid | MED24 | IPCSVZSSVZVIGE-UHFFFAOYSA-N | TCMSP | 0.436402482 |
| LC | palmitic acid | GPC3 | IPCSVZSSVZVIGE-UHFFFAOYSA-N | TCMSP | 0.436402482 |
| LC | palmitic acid | QRFPR | IPCSVZSSVZVIGE-UHFFFAOYSA-N | TCMSP | 0.436402482 |
| LC | palmitic acid | NMB | IPCSVZSSVZVIGE-UHFFFAOYSA-N | TCMSP | 0.436402482 |
| LC | palmitic acid | HNF4G | IPCSVZSSVZVIGE-UHFFFAOYSA-N | TCMSP | 0.436402482 |
| LC | palmitic acid | HRH1 | IPCSVZSSVZVIGE-UHFFFAOYSA-N | TCMSP | 0.436402482 |
| LC | palmitic acid | MED13 | IPCSVZSSVZVIGE-UHFFFAOYSA-N | TCMSP | 0.436402482 |
| LC | palmitic acid | NPS | IPCSVZSSVZVIGE-UHFFFAOYSA-N | TCMSP | 0.436402482 |
| LC | palmitic acid | GCGR | IPCSVZSSVZVIGE-UHFFFAOYSA-N | TCMSP | 0.436402482 |
| LC | palmitic acid | NCOR2 | IPCSVZSSVZVIGE-UHFFFAOYSA-N | TCMSP | 0.436402482 |
| LC | palmitic acid | LPAR2 | IPCSVZSSVZVIGE-UHFFFAOYSA-N | TCMSP | 0.436402482 |
| LC | palmitic acid | MYO7A | IPCSVZSSVZVIGE-UHFFFAOYSA-N | TCMSP | 0.436402482 |
| LC | palmitic acid | GCG | IPCSVZSSVZVIGE-UHFFFAOYSA-N | TCMSP | 0.436402482 |
| LC | palmitic acid | CREB1 | IPCSVZSSVZVIGE-UHFFFAOYSA-N | TCMSP | 0.436402482 |
| LC | palmitic acid | MLN | IPCSVZSSVZVIGE-UHFFFAOYSA-N | TCMSP | 0.436402482 |
| LC | palmitic acid | MAPK9 | IPCSVZSSVZVIGE-UHFFFAOYSA-N | TCMSP | 0.436402482 |
| LC | palmitic acid | TBXA2R | IPCSVZSSVZVIGE-UHFFFAOYSA-N | TCMSP | 0.436402482 |
| LC | palmitic acid | PPT1 | IPCSVZSSVZVIGE-UHFFFAOYSA-N | TCMSP | 0.436402482 |
| LC | palmitic acid | NFYC | IPCSVZSSVZVIGE-UHFFFAOYSA-N | TCMSP | 0.436402482 |
| LC | palmitic acid | NCOA2 | IPCSVZSSVZVIGE-UHFFFAOYSA-N | TCMSP | 0.436402482 |
| LC | palmitic acid | TBL1XR1 | IPCSVZSSVZVIGE-UHFFFAOYSA-N | TCMSP | 0.436402482 |
| LC | palmitic acid | GPX1 | IPCSVZSSVZVIGE-UHFFFAOYSA-N | TCMSP | 0.436402482 |
| LC | palmitic acid | ACADM | IPCSVZSSVZVIGE-UHFFFAOYSA-N | TCMSP | 0.436402482 |
| LC | palmitic acid | HELZ2 | IPCSVZSSVZVIGE-UHFFFAOYSA-N | TCMSP | 0.436402482 |
| LC | palmitic acid | RXRA | IPCSVZSSVZVIGE-UHFFFAOYSA-N | TCMSP | 0.436402482 |
| LC | palmitic acid | CES5A | IPCSVZSSVZVIGE-UHFFFAOYSA-N | TCMSP | 0.436402482 |
| LC | palmitic acid | CCNC | IPCSVZSSVZVIGE-UHFFFAOYSA-N | TCMSP | 0.436402482 |
| LC | palmitic acid | LTB4R2 | IPCSVZSSVZVIGE-UHFFFAOYSA-N | TCMSP | 0.436402482 |
| LC | palmitic acid | GPR68 | IPCSVZSSVZVIGE-UHFFFAOYSA-N | TCMSP | 0.436402482 |
| LC | palmitic acid | TXNRD1 | IPCSVZSSVZVIGE-UHFFFAOYSA-N | TCMSP | 0.436402482 |
| LC | palmitic acid | TRAPPC4 | IPCSVZSSVZVIGE-UHFFFAOYSA-N | TCMSP | 0.436402482 |
| LC | palmitic acid | CES4A | IPCSVZSSVZVIGE-UHFFFAOYSA-N | TCMSP | 0.436402482 |
| LC | palmitic acid | FKBP11 | IPCSVZSSVZVIGE-UHFFFAOYSA-N | TCMSP | 0.436402482 |
| LC | palmitic acid | LDLR | IPCSVZSSVZVIGE-UHFFFAOYSA-N | TCMSP | 0.436402482 |
| LC | palmitic acid | CHD9 | IPCSVZSSVZVIGE-UHFFFAOYSA-N | TCMSP | 0.436402482 |
| LC | OYA | ADH1A | NUJGJRNETVAIRJ-UHFFFAOYSA-N | TCMSP | 0.470208819 |
| LC | OYA | ADH4 | NUJGJRNETVAIRJ-UHFFFAOYSA-N | TCMSP | 0.470208819 |
| LC | OYA | ADH5 | NUJGJRNETVAIRJ-UHFFFAOYSA-N | TCMSP | 0.470208819 |
| LC | OYA | FOS | NUJGJRNETVAIRJ-UHFFFAOYSA-N | TCMSP | 0.470208819 |
| LC | OYA | ADH1B | NUJGJRNETVAIRJ-UHFFFAOYSA-N | TCMSP | 0.470208819 |
| LC | OYA | MBOAT4 | NUJGJRNETVAIRJ-UHFFFAOYSA-N | TCMSP | 0.470208819 |
| LC | OYA | GBA | NUJGJRNETVAIRJ-UHFFFAOYSA-N | TCMSP | 0.470208819 |
| LC | OYA | ALDH3A2 | NUJGJRNETVAIRJ-UHFFFAOYSA-N | TCMSP | 0.470208819 |
| LC | OYA | ALDH9A1 | NUJGJRNETVAIRJ-UHFFFAOYSA-N | TCMSP | 0.470208819 |
| LC | OYA | ALDH1B1 | NUJGJRNETVAIRJ-UHFFFAOYSA-N | TCMSP | 0.470208819 |
| LC | OYA | ADH6 | NUJGJRNETVAIRJ-UHFFFAOYSA-N | TCMSP | 0.470208819 |
| LC | OYA | ADH7 | NUJGJRNETVAIRJ-UHFFFAOYSA-N | TCMSP | 0.470208819 |
| LC | stearic acid | APOC3 | QIQXTHQIDYTFRH-UHFFFAOYSA-N | TCMSP | 0.366166024 |
| LC | stearic acid | RBP1 | QIQXTHQIDYTFRH-UHFFFAOYSA-N | TCMSP | 0.366166024 |
| LC | stearic acid | APOB | QIQXTHQIDYTFRH-UHFFFAOYSA-N | TCMSP | 0.366166024 |
| LC | stearic acid | APOA1 | QIQXTHQIDYTFRH-UHFFFAOYSA-N | TCMSP | 0.366166024 |
| LC | stearic acid | ACOT2 | QIQXTHQIDYTFRH-UHFFFAOYSA-N | TCMSP | 0.366166024 |
| LC | stearic acid | LRP1 | QIQXTHQIDYTFRH-UHFFFAOYSA-N | TCMSP | 0.366166024 |
| LC | stearic acid | APOE | QIQXTHQIDYTFRH-UHFFFAOYSA-N | TCMSP | 0.366166024 |
| LC | stearic acid | SDC1 | QIQXTHQIDYTFRH-UHFFFAOYSA-N | TCMSP | 0.366166024 |
| LC | stearic acid | FABP4 | QIQXTHQIDYTFRH-UHFFFAOYSA-N | TCMSP | 0.366166024 |
| LC | stearic acid | BAAT | QIQXTHQIDYTFRH-UHFFFAOYSA-N | TCMSP | 0.366166024 |
| LC | stearic acid | RPE65 | QIQXTHQIDYTFRH-UHFFFAOYSA-N | TCMSP | 0.366166024 |
| LC | stearic acid | PPARA | QIQXTHQIDYTFRH-UHFFFAOYSA-N | TCMSP | 0.366166024 |
| LC | stearic acid | LRP2 | QIQXTHQIDYTFRH-UHFFFAOYSA-N | TCMSP | 0.366166024 |
| LC | stearic acid | GPC1 | QIQXTHQIDYTFRH-UHFFFAOYSA-N | TCMSP | 0.366166024 |
| LC | stearic acid | GPC2 | QIQXTHQIDYTFRH-UHFFFAOYSA-N | TCMSP | 0.366166024 |
| LC | stearic acid | ALB | QIQXTHQIDYTFRH-UHFFFAOYSA-N | TCMSP | 0.366166024 |
| LC | stearic acid | ACER1 | QIQXTHQIDYTFRH-UHFFFAOYSA-N | TCMSP | 0.366166024 |
| LC | stearic acid | LRP8 | QIQXTHQIDYTFRH-UHFFFAOYSA-N | TCMSP | 0.366166024 |
| LC | stearic acid | SDC2 | QIQXTHQIDYTFRH-UHFFFAOYSA-N | TCMSP | 0.366166024 |
| LC | stearic acid | PCSK1 | QIQXTHQIDYTFRH-UHFFFAOYSA-N | TCMSP | 0.366166024 |
| LC | stearic acid | PPARD | QIQXTHQIDYTFRH-UHFFFAOYSA-N | TCMSP | 0.366166024 |
| LC | stearic acid | ACOT1 | QIQXTHQIDYTFRH-UHFFFAOYSA-N | TCMSP | 0.366166024 |
| LC | stearic acid | SCD5 | QIQXTHQIDYTFRH-UHFFFAOYSA-N | TCMSP | 0.366166024 |
| LC | stearic acid | ACOT4 | QIQXTHQIDYTFRH-UHFFFAOYSA-N | TCMSP | 0.366166024 |
| LC | stearic acid | ADORA2A | QIQXTHQIDYTFRH-UHFFFAOYSA-N | TCMSP | 0.366166024 |
| LC | stearic acid | LRAT | QIQXTHQIDYTFRH-UHFFFAOYSA-N | TCMSP | 0.366166024 |
| LC | stearic acid | ACER2 | QIQXTHQIDYTFRH-UHFFFAOYSA-N | TCMSP | 0.366166024 |
| LC | stearic acid | SDC3 | QIQXTHQIDYTFRH-UHFFFAOYSA-N | TCMSP | 0.366166024 |
| LC | stearic acid | INSIG1 | QIQXTHQIDYTFRH-UHFFFAOYSA-N | TCMSP | 0.366166024 |
| LC | stearic acid | SOD2 | QIQXTHQIDYTFRH-UHFFFAOYSA-N | TCMSP | 0.366166024 |
| LC | stearic acid | APOA2 | QIQXTHQIDYTFRH-UHFFFAOYSA-N | TCMSP | 0.366166024 |
| LC | stearic acid | SCD | QIQXTHQIDYTFRH-UHFFFAOYSA-N | TCMSP | 0.366166024 |
| LC | stearic acid | GPC4 | QIQXTHQIDYTFRH-UHFFFAOYSA-N | TCMSP | 0.366166024 |
| LC | stearic acid | FFAR4 | QIQXTHQIDYTFRH-UHFFFAOYSA-N | TCMSP | 0.366166024 |
| LC | stearic acid | PTPN1 | QIQXTHQIDYTFRH-UHFFFAOYSA-N | TCMSP | 0.366166024 |
| LC | stearic acid | SDC4 | QIQXTHQIDYTFRH-UHFFFAOYSA-N | TCMSP | 0.366166024 |
| LC | stearic acid | FABP3 | QIQXTHQIDYTFRH-UHFFFAOYSA-N | TCMSP | 0.366166024 |
| LC | stearic acid | HSPG2 | QIQXTHQIDYTFRH-UHFFFAOYSA-N | TCMSP | 0.366166024 |
| LC | stearic acid | PLA2G2D | QIQXTHQIDYTFRH-UHFFFAOYSA-N | TCMSP | 0.366166024 |
| LC | stearic acid | GPC6 | QIQXTHQIDYTFRH-UHFFFAOYSA-N | TCMSP | 0.366166024 |
| LC | stearic acid | GPC5 | QIQXTHQIDYTFRH-UHFFFAOYSA-N | TCMSP | 0.366166024 |
| LC | stearic acid | ACOT7 | QIQXTHQIDYTFRH-UHFFFAOYSA-N | TCMSP | 0.366166024 |
| LC | stearic acid | OLAH | QIQXTHQIDYTFRH-UHFFFAOYSA-N | TCMSP | 0.366166024 |
| LC | stearic acid | AGRN | QIQXTHQIDYTFRH-UHFFFAOYSA-N | TCMSP | 0.366166024 |
| LC | stearic acid | GPC3 | QIQXTHQIDYTFRH-UHFFFAOYSA-N | TCMSP | 0.366166024 |
| LC | stearic acid | ACBD5 | QIQXTHQIDYTFRH-UHFFFAOYSA-N | TCMSP | 0.366166024 |
| LC | stearic acid | MYO7A | QIQXTHQIDYTFRH-UHFFFAOYSA-N | TCMSP | 0.366166024 |
| LC | stearic acid | GCG | QIQXTHQIDYTFRH-UHFFFAOYSA-N | TCMSP | 0.366166024 |
| LC | stearic acid | ACBD4 | QIQXTHQIDYTFRH-UHFFFAOYSA-N | TCMSP | 0.366166024 |
| LC | stearic acid | ACER3 | QIQXTHQIDYTFRH-UHFFFAOYSA-N | TCMSP | 0.366166024 |
| LC | stearic acid | LDLR | QIQXTHQIDYTFRH-UHFFFAOYSA-N | TCMSP | 0.366166024 |
| LC | methyl palmitate | PTGS2 | FLIACVVOZYBSBS-UHFFFAOYSA-N | TCMSP | 0.373916111 |
| LC | methyl palmitate | TNF | FLIACVVOZYBSBS-UHFFFAOYSA-N | TCMSP | 0.373916111 |
| LC | methyl palmitate | IL6 | FLIACVVOZYBSBS-UHFFFAOYSA-N | TCMSP | 0.373916111 |
| LC | methyl palmitate | PTGER3 | FLIACVVOZYBSBS-UHFFFAOYSA-N | TCMSP | 0.373916111 |
| LC | methyl palmitate | IL10 | FLIACVVOZYBSBS-UHFFFAOYSA-N | TCMSP | 0.373916111 |
| LC | beta-elemene | CASP3 | OPFTUNCRGUEPRZ-QLFBSQMISA-N | TCMSP | 0.279562 |
