# Supplementary material for: Network Pharmacology and Metabolomics Studies on Antimigraine Mechanisms of Da Chuan Xiong Fang (DCXF)
Source: Evid Based Complement Alternat Med. 2021 Apr 20;2021:6665137. doi: 10.1155/2021/6665137 (PMC8081595; doi:10.1155/2021/6665137)
Supplement: Supplementary Materials — Supplementary S1: preparation, quality control, and HPLC of DCXF, GE, and LC. Supplementary S2: ingredients from LC and GE. Supplementary S3: QED results of GE and LC. Supplementary S4: 531 core targets. Supplementary S5: migraine genes. Supplementary S6: ARRIVE statement for animal experiments. Supplementary S7: metabolites of serum of brain tissue. Supplementary S8: all active ingredients molecular docking results. Supplementary S9: results of MCODE. Supplementary S10: effect of DCXF on serum and brain tissue metabolic profiling. Supplementary S11: gene-metabolite interaction network. Supplementary S12: GTEx RNA-seq data to verify the expression of hub genes in the brain tissues. [file 6665137.f1.zip › 6665137.f1/Supplementary S4 531 core targets.docx]

**Supplementary S4 531 core targets**

|  | GeneID | GeneName |
| --- | --- | --- |
| 1 | 7124 | TNF |
| 2 | 125 | ADH1B |
| 3 | 130 | ADH6 |
| 4 | 1579 | CYP4A11 |
| 5 | 2353 | FOS |
| 6 | 2641 | GCG |
| 7 | 3569 | IL6 |
| 8 | 3576 | CXCL8 |
| 9 | 836 | CASP3 |
| 10 | 11332 | ACOT7 |
| 11 | 2167 | FABP4 |
| 12 | 335 | APOA1 |
| 13 | 338 | APOB |
| 14 | 348 | APOE |
| 15 | 351 | APP |
| 16 | 3949 | LDLR |
| 17 | 54331 | GNG2 |
| 18 | 570 | BAAT |
| 19 | 5743 | PTGS2 |
| 20 | 7299 | TYR |
| 21 | 10891 | PPARGC1A |
| 22 | 1103 | CHAT |
| 23 | 124 | ADH1A |
| 24 | 127 | ADH4 |
| 25 | 128 | ADH5 |
| 26 | 131 | ADH7 |
| 27 | 135 | ADORA2A |
| 28 | 1558 | CYP2C8 |
| 29 | 162514 | TRPV3 |
| 30 | 213 | ALB |
| 31 | 2168 | FABP1 |
| 32 | 2170 | FABP3 |
| 33 | 2180 | ACSL1 |
| 34 | 2182 | ACSL4 |
| 35 | 3060 | HCRT |
| 36 | 3177 | SLC29A2 |
| 37 | 338557 | FFAR4 |
| 38 | 3586 | IL10 |
| 39 | 3725 | JUN |
| 40 | 376497 | SLC27A1 |
| 41 | 43 | ACHE |
| 42 | 4318 | MMP9 |
| 43 | 4843 | NOS2 |
| 44 | 4860 | PNP |
| 45 | 4886 | NPY1R |
| 46 | 51228 | GLTP |
| 47 | 5465 | PPARA |
| 48 | 55301 | OLAH |
| 49 | 5594 | MAPK1 |
| 50 | 5599 | MAPK8 |
| 51 | 5879 | RAC1 |
| 52 | 594857 | NPS |
| 53 | 5972 | REN |
| 54 | 1E+08 | PLA2G4B |
| 55 | 1.01E+08 | CYP3A7-CYP3A51P |
| 56 | 107 | ADCY1 |
| 57 | 108 | ADCY2 |
| 58 | 109 | ADCY3 |
| 59 | 11001 | SLC27A2 |
| 60 | 111 | ADCY5 |
| 61 | 112 | ADCY6 |
| 62 | 11255 | HRH3 |
| 63 | 113 | ADCY7 |
| 64 | 114 | ADCY8 |
| 65 | 115 | ADCY9 |
| 66 | 123 | PLIN2 |
| 67 | 123745 | PLA2G4E |
| 68 | 1268 | CNR1 |
| 69 | 1277 | COL1A1 |
| 70 | 1312 | COMT |
| 71 | 1374 | CPT1A |
| 72 | 1376 | CPT2 |
| 73 | 1385 | CREB1 |
| 74 | 1387 | CREBBP |
| 75 | 1499 | CTNNB1 |
| 76 | 1544 | CYP1A2 |
| 77 | 1549 | CYP2A7 |
| 78 | 1553 | CYP2A13 |
| 79 | 1555 | CYP2B6 |
| 80 | 1557 | CYP2C19 |
| 81 | 1559 | CYP2C9 |
| 82 | 156 | GRK2 |
| 83 | 1562 | CYP2C18 |
| 84 | 1565 | CYP2D6 |
| 85 | 1572 | CYP2F1 |
| 86 | 1573 | CYP2J2 |
| 87 | 1577 | CYP3A5 |
| 88 | 1580 | CYP4B1 |
| 89 | 1813 | DRD2 |
| 90 | 1814 | DRD3 |
| 91 | 1815 | DRD4 |
| 92 | 185 | AGTR1 |
| 93 | 19 | ABCA1 |
| 94 | 1909 | EDNRA |
| 95 | 1910 | EDNRB |
| 96 | 196883 | ADCY4 |
| 97 | 1977 | EIF4E |
| 98 | 199974 | CYP4Z1 |
| 99 | 2152 | F3 |
| 100 | 2169 | FABP2 |
| 101 | 2171 | FABP5 |
| 102 | 218 | ALDH3A1 |
| 103 | 2222 | FDFT1 |
| 104 | 222545 | GPRC6A |
| 105 | 224 | ALDH3A2 |
| 106 | 23236 | PLCB1 |
| 107 | 23446 | SLC44A1 |
| 108 | 2359 | FPR3 |
| 109 | 240 | ALOX5 |
| 110 | 257313 | UTS2B |
| 111 | 260293 | CYP4X1 |
| 112 | 2740 | GLP1R |
| 113 | 2770 | GNAI1 |
| 114 | 2771 | GNAI2 |
| 115 | 2773 | GNAI3 |
| 116 | 2776 | GNAQ |
| 117 | 2798 | GNRHR |
| 118 | 283748 | PLA2G4D |
| 119 | 2852 | GPER1 |
| 120 | 2867 | FFAR2 |
| 121 | 2911 | GRM1 |
| 122 | 2912 | GRM2 |
| 123 | 2913 | GRM3 |
| 124 | 2914 | GRM4 |
| 125 | 2915 | GRM5 |
| 126 | 2916 | GRM6 |
| 127 | 2917 | GRM7 |
| 128 | 2918 | GRM8 |
| 129 | 2919 | CXCL1 |
| 130 | 2920 | CXCL2 |
| 131 | 2921 | CXCL3 |
| 132 | 29785 | CYP2S1 |
| 133 | 3156 | HMGCR |
| 134 | 3157 | HMGCS1 |
| 135 | 3158 | HMGCS2 |
| 136 | 3350 | HTR1A |
| 137 | 3351 | HTR1B |
| 138 | 3354 | HTR1E |
| 139 | 3355 | HTR1F |
| 140 | 3356 | HTR2A |
| 141 | 3357 | HTR2B |
| 142 | 3358 | HTR2C |
| 143 | 3361 | HTR5A |
| 144 | 3383 | ICAM1 |
| 145 | 3630 | INS |
| 146 | 3777 | KCNK3 |
| 147 | 3992 | FADS1 |
| 148 | 406 | ARNTL |
| 149 | 4543 | MTNR1A |
| 150 | 468 | ATF4 |
| 151 | 4801 | NFYB |
| 152 | 4846 | NOS3 |
| 153 | 4852 | NPY |
| 154 | 4915 | NTRK2 |
| 155 | 51129 | ANGPTL4 |
| 156 | 5130 | PCYT1A |
| 157 | 5290 | PIK3CA |
| 158 | 5319 | PLA2G1B |
| 159 | 5320 | PLA2G2A |
| 160 | 5330 | PLCB2 |
| 161 | 5331 | PLCB3 |
| 162 | 5468 | PPARG |
| 163 | 5473 | PPBP |
| 164 | 5579 | PRKCB |
| 165 | 5730 | PTGDS |
| 166 | 5742 | PTGS1 |
| 167 | 5770 | PTPN1 |
| 168 | 57834 | CYP4F11 |
| 169 | 5881 | RAC3 |
| 170 | 596 | BCL2 |
| 171 | 6198 | RPS6KB1 |
| 172 | 627 | BDNF |
| 173 | 6319 | SCD |
| 174 | 635 | BHMT |
| 175 | 66002 | CYP4F12 |
| 176 | 6647 | SOD1 |
| 177 | 6667 | SP1 |
| 178 | 6721 | SREBF2 |
| 179 | 6863 | TAC1 |
| 180 | 6869 | TACR1 |
| 181 | 7157 | TP53 |
| 182 | 7200 | TRH |
| 183 | 7422 | VEGFA |
| 184 | 8560 | DEGS1 |
| 185 | 886 | CCKAR |
| 186 | 892 | CCNC |
| 187 | 9294 | S1PR2 |
| 188 | 9365 | KL |
| 189 | 9415 | FADS2 |
| 190 | 948 | CD36 |
| 191 | 9575 | CLOCK |
| 192 | 100 | ADA |
| 193 | 10005 | ACOT8 |
| 194 | 1.01E+08 | NT5C1B-RDH14 |
| 195 | 1025 | CDK9 |
| 196 | 10309 | CCNO |
| 197 | 10396 | ATP8A1 |
| 198 | 10434 | LYPLA1 |
| 199 | 1050 | CEBPA |
| 200 | 1052 | CEBPD |
| 201 | 1071 | CETP |
| 202 | 10768 | AHCYL1 |
| 203 | 10999 | SLC27A4 |
| 204 | 11104 | KATNA1 |
| 205 | 1119 | CHKA |
| 206 | 1120 | CHKB |
| 207 | 113179 | ADAT3 |
| 208 | 1137 | CHRNA4 |
| 209 | 1139 | CHRNA7 |
| 210 | 114548 | NLRP3 |
| 211 | 1149 | CIDEA |
| 212 | 116 | ADCYAP1 |
| 213 | 122042 | RXFP2 |
| 214 | 122553 | TRAPPC6B |
| 215 | 122618 | PLD4 |
| 216 | 132 | ADK |
| 217 | 134864 | TAAR1 |
| 218 | 136 | ADORA2B |
| 219 | 1375 | CPT1B |
| 220 | 1392 | CRH |
| 221 | 139596 | UPRT |
| 222 | 1432 | MAPK14 |
| 223 | 145226 | RDH12 |
| 224 | 1511 | CTSG |
| 225 | 1528 | CYB5A |
| 226 | 153 | ADRB1 |
| 227 | 154 | ADRB2 |
| 228 | 155 | ADRB3 |
| 229 | 1583 | CYP11A1 |
| 230 | 158584 | FAAH2 |
| 231 | 1586 | CYP17A1 |
| 232 | 1589 | CYP21A2 |
| 233 | 161823 | ADAL |
| 234 | 1633 | DCK |
| 235 | 1641 | DCX |
| 236 | 1644 | DDC |
| 237 | 1649 | DDIT3 |
| 238 | 1723 | DHODH |
| 239 | 1803 | DPP4 |
| 240 | 1806 | DPYD |
| 241 | 1807 | DPYS |
| 242 | 1816 | DRD5 |
| 243 | 1890 | TYMP |
| 244 | 191 | AHCY |
| 245 | 1958 | EGR1 |
| 246 | 1981 | EIF4G1 |
| 247 | 1991 | ELANE |
| 248 | 201164 | PLD6 |
| 249 | 2028 | ENPEP |
| 250 | 2030 | SLC29A1 |
| 251 | 2056 | EPO |
| 252 | 2058 | EPRS |
| 253 | 2064 | ERBB2 |
| 254 | 207 | AKT1 |
| 255 | 2166 | FAAH |
| 256 | 217 | ALDH2 |
| 257 | 2173 | FABP7 |
| 258 | 219 | ALDH1B1 |
| 259 | 2194 | FASN |
| 260 | 220 | ALDH1A3 |
| 261 | 221120 | ALKBH3 |
| 262 | 222 | ALDH3B2 |
| 263 | 222236 | NAPEPLD |
| 264 | 222962 | SLC29A4 |
| 265 | 223 | ALDH9A1 |
| 266 | 2247 | FGF2 |
| 267 | 2252 | FGF7 |
| 268 | 22796 | COG2 |
| 269 | 22926 | ATF6 |
| 270 | 22978 | NT5C2 |
| 271 | 23038 | WDTC1 |
| 272 | 2309 | FOXO3 |
| 273 | 2324 | FLT4 |
| 274 | 2328 | FMO3 |
| 275 | 2335 | FN1 |
| 276 | 23382 | AHCYL2 |
| 277 | 23411 | SIRT1 |
| 278 | 23451 | SF3B1 |
| 279 | 23549 | DNPEP |
| 280 | 23590 | PDSS1 |
| 281 | 23646 | PLD3 |
| 282 | 246 | ALOX15 |
| 283 | 247 | ALOX15B |
| 284 | 2475 | MTOR |
| 285 | 2521 | FUS |
| 286 | 2534 | FYN |
| 287 | 25824 | PRDX5 |
| 288 | 259 | AMBP |
| 289 | 26253 | CLEC4E |
| 290 | 26279 | PLA2G2D |
| 291 | 2629 | GBA |
| 292 | 2638 | GC |
| 293 | 2668 | GDNF |
| 294 | 27095 | TRAPPC3 |
| 295 | 27125 | AFF4 |
| 296 | 27235 | COQ2 |
| 297 | 27430 | MAT2B |
| 298 | 2760 | GM2A |
| 299 | 2778 | GNAS |
| 300 | 284904 | SEC14L4 |
| 301 | 2876 | GPX1 |
| 302 | 2877 | GPX2 |
| 303 | 2878 | GPX3 |
| 304 | 2879 | GPX4 |
| 305 | 28965 | SLC27A6 |
| 306 | 2903 | GRIN2A |
| 307 | 3039 | HBA1 |
| 308 | 3043 | HBB |
| 309 | 3094 | HINT1 |
| 310 | 310 | ANXA7 |
| 311 | 314 | AOC2 |
| 312 | 3162 | HMOX1 |
| 313 | 3164 | NR4A1 |
| 314 | 3174 | HNF4G |
| 315 | 3184 | HNRNPD |
| 316 | 32 | ACACB |
| 317 | 3251 | HPRT1 |
| 318 | 3274 | HRH2 |
| 319 | 3283 | HSD3B1 |
| 320 | 3284 | HSD3B2 |
| 321 | 3320 | HSP90AA1 |
| 322 | 3360 | HTR4 |
| 323 | 3362 | HTR6 |
| 324 | 3363 | HTR7 |
| 325 | 3479 | IGF1 |
| 326 | 353 | APRT |
| 327 | 3552 | IL1A |
| 328 | 3553 | IL1B |
| 329 | 3596 | IL13 |
| 330 | 3636 | INPPL1 |
| 331 | 3638 | INSIG1 |
| 332 | 3651 | PDX1 |
| 333 | 3667 | IRS1 |
| 334 | 367 | AR |
| 335 | 3674 | ITGA2B |
| 336 | 3687 | ITGAX |
| 337 | 3692 | EIF6 |
| 338 | 3728 | JUP |
| 339 | 3783 | KCNN4 |
| 340 | 3832 | KIF11 |
| 341 | 387 | RHOA |
| 342 | 3906 | LALBA |
| 343 | 3934 | LCN2 |
| 344 | 3952 | LEP |
| 345 | 3973 | LHCGR |
| 346 | 4056 | LTC4S |
| 347 | 412 | STS |
| 348 | 4128 | MAOA |
| 349 | 4129 | MAOB |
| 350 | 414149 | ACBD7 |
| 351 | 4144 | MAT2A |
| 352 | 4157 | MC1R |
| 353 | 4158 | MC2R |
| 354 | 4159 | MC3R |
| 355 | 4160 | MC4R |
| 356 | 4161 | MC5R |
| 357 | 4282 | MIF |
| 358 | 4311 | MME |
| 359 | 4312 | MMP1 |
| 360 | 4507 | MTAP |
| 361 | 4548 | MTR |
| 362 | 4552 | MTRR |
| 363 | 4595 | MUTYH |
| 364 | 47 | ACLY |
| 365 | 4803 | NGF |
| 366 | 4907 | NT5E |
| 367 | 4914 | NTRK1 |
| 368 | 4973 | OLR1 |
| 369 | 5045 | FURIN |
| 370 | 5047 | PAEP |
| 371 | 50507 | NOX4 |
| 372 | 5054 | SERPINE1 |
| 373 | 5066 | PAM |
| 374 | 51004 | COQ6 |
| 375 | 5105 | PCK1 |
| 376 | 5106 | PCK2 |
| 377 | 51303 | FKBP11 |
| 378 | 51399 | TRAPPC4 |
| 379 | 5160 | PDHA1 |
| 380 | 51639 | SF3B6 |
| 381 | 51738 | GHRL |
| 382 | 51816 | ADA2 |
| 383 | 52 | ACP1 |
| 384 | 5292 | PIM1 |
| 385 | 5313 | PKLR |
| 386 | 5321 | PLA2G4A |
| 387 | 5328 | PLAU |
| 388 | 5337 | PLD1 |
| 389 | 5338 | PLD2 |
| 390 | 5340 | PLG |
| 391 | 5375 | PMP2 |
| 392 | 5406 | PNLIP |
| 393 | 5409 | PNMT |
| 394 | 54205 | CYCS |
| 395 | 5424 | POLD1 |
| 396 | 5444 | PON1 |
| 397 | 54658 | UGT1A1 |
| 398 | 5467 | PPARD |
| 399 | 54963 | UCKL1 |
| 400 | 5524 | PTPA |
| 401 | 55349 | CHDH |
| 402 | 55361 | PI4K2A |
| 403 | 5538 | PPT1 |
| 404 | 554 | AVPR2 |
| 405 | 55500 | ETNK1 |
| 406 | 5566 | PRKACA |
| 407 | 55711 | FAR2 |
| 408 | 55743 | CHFR |
| 409 | 5580 | PRKCD |
| 410 | 55825 | PECR |
| 411 | 5592 | PRKG1 |
| 412 | 5595 | MAPK3 |
| 413 | 5601 | MAPK9 |
| 414 | 5602 | MAPK10 |
| 415 | 5603 | MAPK13 |
| 416 | 56261 | GPCPD1 |
| 417 | 5644 | PRSS1 |
| 418 | 56729 | RETN |
| 419 | 57107 | PDSS2 |
| 420 | 57153 | SLC44A2 |
| 421 | 5728 | PTEN |
| 422 | 5729 | PTGDR |
| 423 | 5732 | PTGER2 |
| 424 | 5734 | PTGER4 |
| 425 | 5739 | PTGIR |
| 426 | 5741 | PTH |
| 427 | 581 | BAX |
| 428 | 5837 | PYGM |
| 429 | 5894 | RAF1 |
| 430 | 590 | BCHE |
| 431 | 5914 | RARA |
| 432 | 593 | BCKDHA |
| 433 | 59350 | RXFP1 |
| 434 | 5950 | RBP4 |
| 435 | 5970 | RELA |
| 436 | 5979 | RET |
| 437 | 6010 | RHO |
| 438 | 6035 | RNASE1 |
| 439 | 60482 | SLC5A7 |
| 440 | 619373 | MBOAT4 |
| 441 | 6249 | CLIP1 |
| 442 | 6343 | SCT |
| 443 | 6347 | CCL2 |
| 444 | 6376 | CX3CL1 |
| 445 | 6401 | SELE |
| 446 | 6403 | SELP |
| 447 | 64078 | SLC28A3 |
| 448 | 6514 | SLC2A2 |
| 449 | 652 | BMP4 |
| 450 | 6532 | SLC6A4 |
| 451 | 6572 | SLC18A3 |
| 452 | 6580 | SLC22A1 |
| 453 | 6582 | SLC22A2 |
| 454 | 6584 | SLC22A5 |
| 455 | 6648 | SOD2 |
| 456 | 6696 | SPP1 |
| 457 | 6720 | SREBF1 |
| 458 | 6733 | SRPK2 |
| 459 | 6770 | STAR |
| 460 | 6774 | STAT3 |
| 461 | 6790 | AURKA |
| 462 | 6820 | SULT2B1 |
| 463 | 6839 | SUV39H1 |
| 464 | 6862 | TBXT |
| 465 | 6885 | MAP3K7 |
| 466 | 6916 | TBXAS1 |
| 467 | 695 | BTK |
| 468 | 6996 | TDG |
| 469 | 7076 | TIMP1 |
| 470 | 7097 | TLR2 |
| 471 | 7099 | TLR4 |
| 472 | 7253 | TSHR |
| 473 | 7276 | TTR |
| 474 | 7345 | UCHL1 |
| 475 | 7351 | UCP2 |
| 476 | 7357 | UGCG |
| 477 | 7374 | UNG |
| 478 | 7412 | VCAM1 |
| 479 | 7424 | VEGFC |
| 480 | 7432 | VIP |
| 481 | 7433 | VIPR1 |
| 482 | 7442 | TRPV1 |
| 483 | 7494 | XBP1 |
| 484 | 7511 | XPNPEP1 |
| 485 | 760 | CA2 |
| 486 | 79090 | TRAPPC6A |
| 487 | 7915 | ALDH5A1 |
| 488 | 796 | CALCA |
| 489 | 79777 | ACBD4 |
| 490 | 284541 | CYP4A22 |
| 491 | 7980 | TFPI2 |
| 492 | 79966 | SCD5 |
| 493 | 80036 | TRPM3 |
| 494 | 834 | CASP1 |
| 495 | 83480 | PUS3 |
| 496 | 839 | CASP6 |
| 497 | 8398 | PLA2G6 |
| 498 | 8399 | PLA2G10 |
| 499 | 84188 | FAR1 |
| 500 | 8428 | STK24 |
| 501 | 8443 | GNPAT |
| 502 | 84618 | NT5C1A |
| 503 | 847 | CAT |
| 504 | 8540 | AGPS |
| 505 | 857 | CAV1 |
| 506 | 8573 | CASK |
| 507 | 8639 | AOC3 |
| 508 | 8685 | MARCO |
| 509 | 8739 | HRK |
| 510 | 8795 | TNFRSF10B |
| 511 | 8846 | ALKBH1 |
| 512 | 8856 | NR1I2 |
| 513 | 8877 | SPHK1 |
| 514 | 8930 | MBD4 |
| 515 | 8989 | TRPA1 |
| 516 | 904 | CCNT1 |
| 517 | 9131 | AIFM1 |
| 518 | 91452 | ACBD5 |
| 519 | 9153 | SLC28A2 |
| 520 | 9154 | SLC28A1 |
| 521 | 93 | ACVR2B |
| 522 | 9370 | ADIPOQ |
| 523 | 9374 | PPT2 |
| 524 | 9420 | CYP7B1 |
| 525 | 9429 | ABCG2 |
| 526 | 9468 | PCYT1B |
| 527 | 9536 | PTGES |
| 528 | 9735 | KNTC1 |
| 529 | 9791 | PTDSS1 |
| 530 | 9970 | NR1I3 |
| 531 | 9976 | CLEC2B |
