# Supplementary material for: Network Pharmacology and Metabolomics Studies on Antimigraine Mechanisms of Da Chuan Xiong Fang (DCXF)
Source: Evid Based Complement Alternat Med. 2021 Apr 20;2021:6665137. doi: 10.1155/2021/6665137 (PMC8081595; doi:10.1155/2021/6665137)
Supplement: Supplementary Materials — Supplementary S1: preparation, quality control, and HPLC of DCXF, GE, and LC. Supplementary S2: ingredients from LC and GE. Supplementary S3: QED results of GE and LC. Supplementary S4: 531 core targets. Supplementary S5: migraine genes. Supplementary S6: ARRIVE statement for animal experiments. Supplementary S7: metabolites of serum of brain tissue. Supplementary S8: all active ingredients molecular docking results. Supplementary S9: results of MCODE. Supplementary S10: effect of DCXF on serum and brain tissue metabolic profiling. Supplementary S11: gene-metabolite interaction network. Supplementary S12: GTEx RNA-seq data to verify the expression of hub genes in the brain tissues. [file 6665137.f1.zip › 6665137.f1/Supplementary S5 Migraine genes.docx]

**Supplementary S5 Migraine genes**

In this work, migraine was considered as a disease, and 72 associated genes were collected from the GeneCards database.

| No. | Symbol | GeneID | Description | Category |
| --- | --- | --- | --- | --- |
| 1 | CLCN1 | 1180 | Chloride Voltage-Gated Channel 1 | Protein Coding |
| 2 | PPOX | 5498 | Protoporphyrinogen Oxidase | Protein Coding |
| 3 | BRCA2 | 675 | BRCA2 DNA Repair Associated | Protein Coding |
| 4 | MGR2 | 8249 | Migraine, Familial Typical, Susceptibility To | Genetic Locus |
| 5 | MGR6 | 317773 | Migraine, Several Forms | Genetic Locus |
| 6 | MGR8 | 100188790 | Migraine, Susceptibility To, 8 | Genetic Locus |
| 7 | MGR12 | 100188851 | Migraine, With Or Without Aura, Susceptibility To, 12 | Genetic Locus |
| 8 | MGR3 | 337892 | Migraine, Familial, With Or Without Aura, Susceptibility To | Genetic Locus |
| 9 | MGR5 | 387576 | Migraine With Or Without Aura, Susceptibility To, 5 | Genetic Locus |
| 10 | MGR1 | 192115 | Migraine With Aura, Susceptibility To | Genetic Locus |
| 11 | ATP1A2 | 477 | ATPase Na+/K+ Transporting Subunit Alpha 2 | Protein Coding |
| 12 | CACNA1A | 773 | Calcium Voltage-Gated Channel Subunit Alpha1 A | Protein Coding |
| 13 | CALCA | 796 | Calcitonin Related Polypeptide Alpha | Protein Coding |
| 14 | TNF | 7124 | Tumor Necrosis Factor | Protein Coding |
| 15 | ESR1 | 2099 | Estrogen Receptor 1 | Protein Coding |
| 16 | HTR1B | 3351 | 5-Hydroxytryptamine Receptor 1B | Protein Coding |
| 17 | NOTCH3 | 4854 | Notch Receptor 3 | Protein Coding |
| 18 | SCN1A | 6323 | Sodium Voltage-Gated Channel Alpha Subunit 1 | Protein Coding |
| 19 | HTR3A | 3359 | 5-Hydroxytryptamine Receptor 3A | Protein Coding |
| 20 | TAC1 | 6863 | Tachykinin Precursor 1 | Protein Coding |
| 21 | HTR2A | 3356 | 5-Hydroxytryptamine Receptor 2A | Protein Coding |
| 22 | HTR7 | 3363 | 5-Hydroxytryptamine Receptor 7 | Protein Coding |
| 23 | DRD2 | 1813 | Dopamine Receptor D2 | Protein Coding |
| 24 | HTR2C | 3358 | 5-Hydroxytryptamine Receptor 2C | Protein Coding |
| 25 | MTHFR | 4524 | Methylenetetrahydrofolate Reductase | Protein Coding |
| 26 | MAOA | 4128 | Monoamine Oxidase A | Protein Coding |
| 27 | HTR2B | 3357 | 5-Hydroxytryptamine Receptor 2B | Protein Coding |
| 28 | HTR1D | 3352 | 5-Hydroxytryptamine Receptor 1D | Protein Coding |
| 29 | HTR1F | 3355 | 5-Hydroxytryptamine Receptor 1F | Protein Coding |
| 30 | RAMP1 | 10267 | Receptor Activity Modifying Protein 1 | Protein Coding |
| 31 | CALCRL | 10203 | Calcitonin Receptor Like Receptor | Protein Coding |
| 32 | TACR1 | 6869 | Tachykinin Receptor 1 | Protein Coding |
| 33 | HCRT | 3060 | Hypocretin Neuropeptide Precursor | Protein Coding |
| 34 | APOH | 350 | Apolipoprotein H | Protein Coding |
| 35 | HRH3 | 11255 | Histamine Receptor H3 | Protein Coding |
| 36 | HTR1A | 3350 | 5-Hydroxytryptamine Receptor 1A | Protein Coding |
| 37 | TRPV1 | 7442 | Transient Receptor Potential Cation Channel Subfamily V Member 1 | Protein Coding |
| 38 | VIP | 7432 | Vasoactive Intestinal Peptide | Protein Coding |
| 39 | NPY | 4852 | Neuropeptide Y | Protein Coding |
| 40 | GOLPH3 | 64083 | Golgi Phosphoprotein 3 | Protein Coding |
| 41 | DRD5 | 1816 | Dopamine Receptor D5 | Protein Coding |
| 42 | SLC6A4 | 6532 | Solute Carrier Family 6 Member 4 | Protein Coding |
| 43 | ADCYAP1 | 116 | Adenylate Cyclase Activating Polypeptide 1 | Protein Coding |
| 44 | SLC1A3 | 6507 | Solute Carrier Family 1 Member 3 | Protein Coding |
| 45 | TPH1 | 7166 | Tryptophan Hydroxylase 1 | Protein Coding |
| 46 | KCNK18 | 338567 | Potassium Two Pore Domain Channel Subfamily K Member 18 | Protein Coding |
| 47 | NPS | 594857 | Neuropeptide S | Protein Coding |
| 48 | DRD3 | 1814 | Dopamine Receptor D3 | Protein Coding |
| 49 | DRD4 | 1815 | Dopamine Receptor D4 | Protein Coding |
| 50 | DRD1 | 1812 | Dopamine Receptor D1 | Protein Coding |
| 51 | PPBP | 5473 | Pro-Platelet Basic Protein | Protein Coding |
| 52 | FOS | 2353 | Fos Proto-Oncogene, AP-1 Transcription Factor Subunit | Protein Coding |
| 53 | DBH | 1621 | Dopamine Beta-Hydroxylase | Protein Coding |
| 54 | PRRT2 | 112476 | Proline Rich Transmembrane Protein 2 | Protein Coding |
| 55 | CNR1 | 1268 | Cannabinoid Receptor 1 | Protein Coding |
| 56 | KANTR | 102723508 | KDM5C Adjacent Transcript | RNA Gene |
| 57 | COMT | 1312 | Catechol-O-Methyltransferase | Protein Coding |
| 58 | P2RX3 | 5024 | Purinergic Receptor P2X 3 | Protein Coding |
| 59 | EDNRA | 1909 | Endothelin Receptor Type A | Protein Coding |
| 60 | PDE5A | 8654 | Phosphodiesterase 5A | Protein Coding |
| 61 | TRPA1 | 8989 | Transient Receptor Potential Cation Channel Subfamily A Member 1 | Protein Coding |
| 62 | TRPM8 | 79054 | Transient Receptor Potential Cation Channel Subfamily M Member 8 | Protein Coding |
| 63 | SLC6A3 | 6531 | Solute Carrier Family 6 Member 3 | Protein Coding |
| 64 | EDNRB | 1910 | Endothelin Receptor Type B | Protein Coding |
| 65 | MAOB | 4129 | Monoamine Oxidase B | Protein Coding |
| 66 | PHACTR1 | 221692 | Phosphatase And Actin Regulator 1 | Protein Coding |
| 67 | CLNK | 116449 | Cytokine Dependent Hematopoietic Cell Linker | Protein Coding |
| 68 | CYP2D6 | 1565 | Cytochrome P450 Family 2 Subfamily D Member 6 | Protein Coding |
| 69 | MGR4 | 338342 | Migraine, Susceptibility To, 4 | Genetic Locus |
| 70 | ASTN2 | 23245 | Astrotactin 2 | Protein Coding |
| 71 | PRL | 5617 | Prolactin | Protein Coding |
| 72 | GRIA1 | 2890 | Glutamate Ionotropic Receptor AMPA Type Subunit 1 | Protein Coding |
