# Supplementary material for: Network Pharmacology and Metabolomics Studies on Antimigraine Mechanisms of Da Chuan Xiong Fang (DCXF)
Source: Evid Based Complement Alternat Med. 2021 Apr 20;2021:6665137. doi: 10.1155/2021/6665137 (PMC8081595; doi:10.1155/2021/6665137)
Supplement: Supplementary Materials — Supplementary S1: preparation, quality control, and HPLC of DCXF, GE, and LC. Supplementary S2: ingredients from LC and GE. Supplementary S3: QED results of GE and LC. Supplementary S4: 531 core targets. Supplementary S5: migraine genes. Supplementary S6: ARRIVE statement for animal experiments. Supplementary S7: metabolites of serum of brain tissue. Supplementary S8: all active ingredients molecular docking results. Supplementary S9: results of MCODE. Supplementary S10: effect of DCXF on serum and brain tissue metabolic profiling. Supplementary S11: gene-metabolite interaction network. Supplementary S12: GTEx RNA-seq data to verify the expression of hub genes in the brain tissues. [file 6665137.f1.zip › 6665137.f1/Supplementary S6 ARRIVE Statement for Animal Experiments (1).docx]

| Item | Section/  Paragraph |
| --- | --- |
| **1. Study design** **[1a](https://arriveguidelines.org/arrive-guidelines/study-design/1a)**[The groups being compared, including control groups. If no control group has been used, the rationale should be stated.](https://arriveguidelines.org/arrive-guidelines/study-design/1a)  **[1b](https://arriveguidelines.org/arrive-guidelines/study-design/1b)**[The experimental unit (e.g. a single animal, litter, or cage of animals).](https://arriveguidelines.org/arrive-guidelines/study-design/1b) | **2.4 Animals and treatment**  **Paragraph 1** |
| **2. Sample size** **[2a](https://arriveguidelines.org/arrive-guidelines/sample-size/2a)**[Specify the exact number of experimental units allocated to each group, and the total number in each experiment. Also indicate the total number of animals used.](https://arriveguidelines.org/arrive-guidelines/sample-size/2a)  **[2b](https://arriveguidelines.org/arrive-guidelines/sample-size/2b)**[Explain how the sample size was decided. Provide details of any a priori sample size calculation, if done.](https://arriveguidelines.org/arrive-guidelines/sample-size/2b) | **2.4 Animals and treatment**  **Paragraph 2**  **(We selected a small sample size because our experiment was not a Pre-Clinical experiment and DCXF had been widely applied in ancient China for thousands of years. And the sample size of our experiment was also consistent with our previous research to illustrate the mechanism of DCXF on acute migraine.）** |
| **3. Inclusion and exclusion criteria** **[3a](https://arriveguidelines.org/arrive-guidelines/inclusion-and-exclusion-criteria/3a)**[Describe any criteria used for including or excluding animals (or experimental units) during the experiment, and data points during the analysis. Specify if these criteria were established a priori. If no criteria were set, state this explicitly.](https://arriveguidelines.org/arrive-guidelines/inclusion-and-exclusion-criteria/3a)  **[3b](https://arriveguidelines.org/arrive-guidelines/inclusion-and-exclusion-criteria/3b)**[For each experimental group, report any animals, experimental units, or data points not included in the analysis and explain why. If there were no exclusions, state so.](https://arriveguidelines.org/arrive-guidelines/inclusion-and-exclusion-criteria/3b)  **[3c](https://arriveguidelines.org/arrive-guidelines/inclusion-and-exclusion-criteria/3c)**[For each analysis, report the exact value of n in each experimental group.](https://arriveguidelines.org/arrive-guidelines/inclusion-and-exclusion-criteria/3c) | **2.4 Animals and treatment**  **Paragraph 1 and Paragraph 2** |
| **4. Randomisation** **[4a](https://arriveguidelines.org/arrive-guidelines/randomisation/4a)**[State whether randomisation was used to allocate experimental units to control and treatment groups. If done, provide the method used to generate the randomisation sequence.](https://arriveguidelines.org/arrive-guidelines/randomisation/4a)  **[4b](https://arriveguidelines.org/arrive-guidelines/randomisation/4b)**[Describe the strategy used to minimise potential confounders such as the order of treatments and measurements, or animal/cage location. If confounders were not controlled, state this explicitly.](https://arriveguidelines.org/arrive-guidelines/randomisation/4b) | **2.4 Animals and treatment**  **Paragraph 2**  **(Randomisation was carried out as follows. A total number of 24 rats were divided into three different weight groups (eight animals per group). Each animal was assigned a temporary random number within the weight range group. On the basis of their position on the rack, cages were given a numerical designation. For each group, a cage was selected randomly from the pool of all cages. Two rats were removed from each weight range group and given their permanent numerical designation in the cages. Then, the cages were randomized within the exposure group.)** |
| **5. Blinding** **[5](https://arriveguidelines.org/arrive-guidelines/blinding/5)**[Describe who was aware of the group allocation at the different stages of the experiment (during the allocation, the conduct of the experiment, the outcome assessment, and the data analysis).](https://arriveguidelines.org/arrive-guidelines/blinding/5) | **NA**  **(due to overt migraine activity the experimenter could not be blinded to whether the animal was injected with DCXF or with saline)** |
| **6. Outcome measures** **[6a](https://arriveguidelines.org/arrive-guidelines/outcome-measures/6a)**[Clearly define all outcome measures assessed (e.g. cell death, molecular markers, or behavioural changes).](https://arriveguidelines.org/arrive-guidelines/outcome-measures/6a)  **[6b](https://arriveguidelines.org/arrive-guidelines/outcome-measures/6b)**[For hypothesis-testing studies, specify the primary outcome measure, i.e. the outcome measure that was used to determine the sample size.](https://arriveguidelines.org/arrive-guidelines/outcome-measures/6b) | **2.4 Animals and treatment**  **Last Paragraph** |
| **7. Statistical methods** **[7a](https://arriveguidelines.org/arrive-guidelines/statistical-methods/7a)**[Provide details of the statistical methods used for each analysis, including software used.](https://arriveguidelines.org/arrive-guidelines/statistical-methods/7a)  **[7b](https://arriveguidelines.org/arrive-guidelines/statistical-methods/7b)**[Describe any methods used to assess whether the data met the assumptions of the statistical approach, and what was done if the assumptions were not met.](https://arriveguidelines.org/arrive-guidelines/statistical-methods/7b) | **2.5 Biomedical analysis-Last Paragraph 2.6.3 Data collection and analysis-Last Paragraph**  **2.7.3 Data collection and analysis-Last Paragraph**  **2.8Multivariate analysis and potential biomarker selection** |
| **8. Experimental animals** **[8a](https://arriveguidelines.org/arrive-guidelines/experimental-animals/8a)**[Provide species-appropriate details of the animals used, including species, strain and substrain, sex, age or developmental stage, and, if relevant, weight.](https://arriveguidelines.org/arrive-guidelines/experimental-animals/8a)  **[8b](https://arriveguidelines.org/arrive-guidelines/experimental-animals/8b)**[Provide further relevant information on the provenance of animals, health/immune status, genetic modification status, genotype, and any previous procedures.](https://arriveguidelines.org/arrive-guidelines/experimental-animals/8b) | **2.4 Animals and treatment**  **Paragraph 1 and Paragraph 2** |
| **9. Experimental procedures** For each experimental group, including controls, describe the procedures in enough detail to allow others to replicate them, including:  **[9a](https://arriveguidelines.org/arrive-guidelines/experimental-procedures/9a)**[What was done, how it was done, and what was used.](https://arriveguidelines.org/arrive-guidelines/experimental-procedures/9a)  **[9b](https://arriveguidelines.org/arrive-guidelines/experimental-procedures/9b)**[When and how often.](https://arriveguidelines.org/arrive-guidelines/experimental-procedures/9b)  **[9c](https://arriveguidelines.org/arrive-guidelines/experimental-procedures/9c)**[Where (including detail of any acclimatisation periods).](https://arriveguidelines.org/arrive-guidelines/experimental-procedures/9c)  **[9d](https://arriveguidelines.org/arrive-guidelines/experimental-procedures/9d)**[Why (provide rationale for procedures).](https://arriveguidelines.org/arrive-guidelines/experimental-procedures/9d) | **2.4 Animals and treatment**  **Paragraph 2 and Paragraph 3** |
| **10. Results** For each experiment conducted, including independent replications, report:  **[10a](https://arriveguidelines.org/arrive-guidelines/results/10a)**[Summary/descriptive statistics for each experimental group, with a measure of variability where applicable (e.g. mean and SD, or median and range).](https://arriveguidelines.org/arrive-guidelines/results/10a)  **[10b](https://arriveguidelines.org/arrive-guidelines/results/10b)**[If applicable, the effect size with a confidence interval.](https://arriveguidelines.org/arrive-guidelines/results/10b) | **3.3 Nitroglycerin(NTG) modeling and Biomedical analysis**  **3.4 Metabonomic of serum analysis**  **3.5 Metabonomic of brain tissue analysis**  **Figure 8**  **Figure 9**  **Supplementary S8** |
