# Supplementary material for: Network Pharmacology and Metabolomics Studies on Antimigraine Mechanisms of Da Chuan Xiong Fang (DCXF)
Source: Evid Based Complement Alternat Med. 2021 Apr 20;2021:6665137. doi: 10.1155/2021/6665137 (PMC8081595; doi:10.1155/2021/6665137)
Supplement: Supplementary Materials — Supplementary S1: preparation, quality control, and HPLC of DCXF, GE, and LC. Supplementary S2: ingredients from LC and GE. Supplementary S3: QED results of GE and LC. Supplementary S4: 531 core targets. Supplementary S5: migraine genes. Supplementary S6: ARRIVE statement for animal experiments. Supplementary S7: metabolites of serum of brain tissue. Supplementary S8: all active ingredients molecular docking results. Supplementary S9: results of MCODE. Supplementary S10: effect of DCXF on serum and brain tissue metabolic profiling. Supplementary S11: gene-metabolite interaction network. Supplementary S12: GTEx RNA-seq data to verify the expression of hub genes in the brain tissues. [file 6665137.f1.zip › 6665137.f1/Supplementary S7 Metabolites of serum of brain tissue (1).docx]

**Supplementary S7 Metabolites of serum of brain tissue**

1. **Metabolites of serum**

**1.1 Sample preparation**

Thawed serum samples were centrifuged at 3500 rpm for 10 min at 4 ◦C, then 100 μL supernatant was obtained, 400 μL methanol was added to the supernatant and vortex-mixed for 2 min, then the mixture was centrifuged at 10,000 rpm for 15 min at 4 ◦C and 20 μL of the supernatant was transferred into injection vials and kept at -20 ◦C for UPLC- TOF/MS analysis.

**1.2 UPLC-TOF/MS analysis**

Chromatographic separations were performed on an ACQUITYTM UPLC System (Waters Corporation, Milford, MA), equipped with a binary solvent delivery system and an autosampler. A BEH C18 column (2.1 mm×100 mm, 1.7 μm, Waters Corporation, Milford, USA) was used. The column was maintained at 40 ◦C with a flow rate of 0.4 mL/min. The mobile phase was composed of water (Phase A), acetonitrile (Phase B) (containing 0.1% formic acid) and the optimized gradient elution program of positive ions was set as followed: 0-4 min: 1-30% B; 4-6 min: 30-80% B; 6-8 min: 80-90% B; 8-9 min: 90-100% B; 9-11 min: 100% B; 11-13 min: 100-1% B. The optimized gradient elution program of negative ions was set as followed: 0-1 min: 1-10% B; 1-3 min: 20-70% B; 3-8 min: 70-85% B; 8-9 min: 85-100% B; 9-11 min: 100% B; 11-13 min: 100-1% B. MS spectrometry was carried out on a Waters Q-TOF Premier MS system (Waters Corp., Milford, MA) with an electrospray ionization source (ESI) operating in positive ion mode and negative ion mode. Nitrogen was used as the drying gas. The desolvation gas flow rate was 650 L/h and desolvation temperature was maintained at 350 ◦C. Cone gas flow rate was maintained at 50 L/h and the source temperature was set at 110 ◦C. Capillary voltage and cone voltage was 3000 V and positive mode in 35 V (negative mode in 55V ), respectively. The scan time and interscan delays were 0.28 s and 0.02 s, respectively. All analyses were obtained using an independent reference lock mass ion to ensure accuracy and reproducibility and leucine-enkephalin (m/z 566.2771 in positive mode, m/z 554.2615 in negative mode) was used as the lock mass at the concentration of 50 pg/μL and a flow rate of 10 mL/min. Data were acquired in centroid mode with a scan range from 50 to 1000 and a lock spray frequency of 10 s and averaged over 10 scans for correction.

**1.3 Data collection and analysis**

UPLC-TOF/MS spectra data were first processed by Markerlynx Applications Manager Version 4.1 (Waters, Manchester, UK), including the detection and retention time (R.T.) alignment of peaks in each chromatogram by Apex-Track-peak detection package incorporated in this software. The data were combined into a single matrix after aligning peaks with retention time-exact m/z pair and associated peak intensity. Then, ion intensities of each detected peak were normalized within each sample, to the sum of the intensities in that sample. Specifically, some parameters proposed28 were used as parameters for data processing and were set as follows: retention time: 0-13.10 min, mass range: 50-1000 Da, mass tolerance: 0.05 Da, minimum intensity: 15% of the base peak intensity, maximum mass per retention time: 6 min and retention time tolerance: 0.04 min. The processed data were then imported to SIMCA-P version 12.0 (Umetrics, Umea, Sweden). A feature selection procedure was performed to identify the discriminative metabolites based on both univariate analysis (t-test) and multivariable analysis (Partial Least Square Discriminant Analysis, PLS-DA). Variable Importance in the Projection (VIP) value of the validated PLS-DA model was taken as the measurement index for peak selecting. A variable with higher VIP value implies a greater contribution to the separation of different treatment groups. The metabolites with P<0.05 and VIP>1.0 were considered as potential metabolites in accounting for class discrimination.

**1.4 UPLC-TOF MS method validation**

In our preliminary study, the volume ratio of methanol was added to the serum sample as 4:1, which could produce more detectable and stronger peaks with lower noise, matrix effect, and baseline. Methanol was added to serum samples to avoid the protein “plug” phenomenon, which was a conventional extraction method in metabonomic studies. A full scan of serum metabolites was set in both positive mode and negative mode. More information was obtained in positive mode than in negative mode. The representative base peak intensity (BPI) chromatograms in both positive mode and negative mode were obtained. Method validation was carried out using 10 ions of chromatographic peaks. Precision and reproducibility of UPLC-TOF/MS technique were validated by 6 replicated analyses of one sample and 6 parallel samples prepared by the same protocols (data not shown). The relative standard deviations of peak intensities and areas of the 8 ions were all <0.5%, demonstrating that the established method was valid.

1. **Metabolites of brain tissue**

**2.1 Sample preparation**

The protocol was finally set as followed: 200 μLmethanol water was added to 50 μg brain tissue sample to extract metabolites and the mixture was then vigorously vortexed and centrifuged at 12 000 rpm for 15 min (4 ◦C). The supernatant was collected and the residues were afterward extracted by 200 μL methanol and the methanol extracted aliquots were then mixed with the water-extracted supernatants. The mixtures were dried completely and followed by methoxylation reaction with 80 μL methoxylamine (15 mg/mL in pyridine) at 37 ◦C for 2 h. Subsequently, the sample was trimethylsilylated with BSTFA (with 1% TMCS) at 70 ◦C for 1 h. All the processed samples were immediately analyzed by GC TOF/MS.

**2.2 GC TOF/MS analysis**

GC TOF/MS analysis was performed on an Aligent 6890 gas chromatography system equipped with a Pegasus III time-of-flight mass spectrometer (Pegasus HT, Leco Co., CA, USA). Chromatography was carried out using a DB-5MS capillary column 30 mm×250 mm i.d., 0.25 mm film thickness (Agilent J & W Scientific, Folsom, CA, USA) and helium were used as the carrier gas at the flow rate of 1 mL/min. The temperature of the injector and the transfer line was set at 270 ◦C and 250 ◦C, respectively. And the oven temperature was initially set at 80 ◦C for 0.2 min and raised to 180 ◦C at the rate of 5 ◦C /min, then to 220 ◦C at the rate of 4 ◦C/ min and ramped to 280 ◦C at the rate of 20 ◦C /min and held for 10 min. The ion source temperature was 220 ◦C. The mass spectra of m/z 20-600 were acquired with electron impact ionization (70 eV) at the full scan mode. The detector voltage was set at 1450 V.

**2.3 Data collection and analysis**

**2.3.1 Data extraction and pretreatment**

Raw GC TOF/MS data files were converted to CDF files by ChromaTOF software (v3.30, Leco Co., CA, USA) and CDF files were then processed in Matlab 7.0 (the MathWorks, Inc, USA), including baseline correction, background reduction, smoothing, alignment, time-window setting, and multivariate curve resolution. The resultant data were comprised of sample names, peak retention time, and peak intensity. Some artificial peaks generated by noise, column bleed, and by-products in the silylation procedure were removed manually from the dataset. Then, the three-dimensional dataset was normalized to the area of the internal standard, mean-centered, and treated by unit variance scaling for further statistical analysis.

**2.3.2 Metabolite identification**

Metabolites were identified by importing the resolved mass spectra to mass spectra library search software National Institute of Standards and Technology (NIST) 08 library using NIST MS Search 2.0 software. Metabolites with similarity >600 were considered reliable (similarity 99.9 means a perfect match between the compound in the sample and the compound in the NIST library) and authentic reference standards were used to further validate identified metabolites including both retention time and mass spectrum match.

**2.4 Multivariate analysis and potential biomarker selection**

The data consisting of sample names, peak indices (RT-m/z pair) and peak areas were introduced to SIMCA-P 12.0 software (Umetrics, Ume Sweden) for multivariate statistical analysis like Partial Least Square Discriminant Analysis (PLS-DA), which was a supervised pattern recognition if samples belonged to different classes or groups, based on prior knowledge. By using PLS-DA, it was possible to visualize clustering and trends of all samples and efficiently detect the influential variables (e.g.metabolites) explaining the differences between samples, or groups of samples, by interpreting the variable weights. Similarly, those with P<0.05 and VIP>1.0 were considered influential in separating different treatment groups and would be chosen as potential biomarker candidates.

**2.5 Method validation**

A representative GC TOF/MS fingerprinting of the control rats’ brain tissue was obtained. GC TOF/MS parameters (the oven temperature program, detector voltage, etc.) were also optimized for better separation, more peaks, and stronger peak intensity, less noise, and matrix effects.

The stability and repeatability of the GC TOF/MS method were verified by 6 replicated analyses of one sample throughout the GC TOF/MS runs. The relative standard deviations of retention time and peak areas of the 8 major peaks were < 1.0% and 12%, respectively, demonstrating that the established method was valid.

References:

S. Ma, L. Shen, M. Chen, et al., “The study of metabonomics combined with diversity of intestinal flora in LDP intervention in kidney-yin deficiency hyperthyroid rats,” RSC Advance, vol. 5, pp. 57975-57983, 2015.

1. Zhu, Y. Feng, L. Shen, et al. “Effect of metformin on the urinary metabolites of diet-induced-obese mice studied by ultra performance liquid chromatography coupled to time-of-flight mass spectrometry (UPLC-TOF/MS),” *Journal of Chromatography B*, vol.925, pp.110-116, 2013.

Y. Zhu, W. Cong, L. Shen, et al. “Fecal metabonomic study of a polysaccharide, MDG-1 from Ophiopogon japonicus on diabetic mice based on gas chromatography/time-of-flight mass spectrometry (GC TOF/MS)”, *Molecular Biosystems*, vol.2, pp.304-312, 2014.

Y. Lei, D. Li, J. Deng, et al., “Metabolomic profiling of three brain regions from a postnatal infected Borna disease virus Hu-H1 rat model,” *Metabolomics*, vol.10, pp. 484-495, 2014.
