# Supplementary material for: Network Pharmacology and Metabolomics Studies on Antimigraine Mechanisms of Da Chuan Xiong Fang (DCXF)
Source: Evid Based Complement Alternat Med. 2021 Apr 20;2021:6665137. doi: 10.1155/2021/6665137 (PMC8081595; doi:10.1155/2021/6665137)
Supplement: Supplementary Materials — Supplementary S1: preparation, quality control, and HPLC of DCXF, GE, and LC. Supplementary S2: ingredients from LC and GE. Supplementary S3: QED results of GE and LC. Supplementary S4: 531 core targets. Supplementary S5: migraine genes. Supplementary S6: ARRIVE statement for animal experiments. Supplementary S7: metabolites of serum of brain tissue. Supplementary S8: all active ingredients molecular docking results. Supplementary S9: results of MCODE. Supplementary S10: effect of DCXF on serum and brain tissue metabolic profiling. Supplementary S11: gene-metabolite interaction network. Supplementary S12: GTEx RNA-seq data to verify the expression of hub genes in the brain tissues. [file 6665137.f1.zip › 6665137.f1/Supplementary S8 All active ingredients molecular.docx]

**Supplementary S8 All active ingredients molecular docking results**

**(the smaller the value of Best-affinity, the better the docking effect)**

| InChIKey | PDB | Best-affinity |
| --- | --- | --- |
| RGHHSNMVTDWUBI-UHFFFAOYSA-N | 6a93 | -5.4 |
| RGHHSNMVTDWUBI-UHFFFAOYSA-N | 3aqf | -4.3 |
| RGHHSNMVTDWUBI-UHFFFAOYSA-N | 3pbl | -5.3 |
| RGHHSNMVTDWUBI-UHFFFAOYSA-N | 4iar | -5.6 |
| RGHHSNMVTDWUBI-UHFFFAOYSA-N | 4n8t | -5.2 |
| RGHHSNMVTDWUBI-UHFFFAOYSA-N | 1nsi | -6.8 |
| MWOOGOJBHIARFG-UHFFFAOYSA-N | 6a93 | -5.5 |
| MWOOGOJBHIARFG-UHFFFAOYSA-N | 3aqf | -4.6 |
| MWOOGOJBHIARFG-UHFFFAOYSA-N | 3pbl | -5.7 |
| MWOOGOJBHIARFG-UHFFFAOYSA-N | 4iar | -5.8 |
| MWOOGOJBHIARFG-UHFFFAOYSA-N | 4n8t | -5.4 |
| MWOOGOJBHIARFG-UHFFFAOYSA-N | 1nsi | -6.9 |
| FJKROLUGYXJWQN-UHFFFAOYSA-N | 6a93 | -5.6 |
| FJKROLUGYXJWQN-UHFFFAOYSA-N | 3aqf | -4.7 |
| FJKROLUGYXJWQN-UHFFFAOYSA-N | 3pbl | -5.6 |
| FJKROLUGYXJWQN-UHFFFAOYSA-N | 4iar | -5.9 |
| FJKROLUGYXJWQN-UHFFFAOYSA-N | 4n8t | -5.8 |
| FJKROLUGYXJWQN-UHFFFAOYSA-N | 1nsi | -7.2 |
| HYBBIBNJHNGZAN-UHFFFAOYSA-N | 6a93 | -4.6 |
| HYBBIBNJHNGZAN-UHFFFAOYSA-N | 3aqf | -3.4 |
| HYBBIBNJHNGZAN-UHFFFAOYSA-N | 3pbl | -4.2 |
| HYBBIBNJHNGZAN-UHFFFAOYSA-N | 4iar | -4.1 |
| HYBBIBNJHNGZAN-UHFFFAOYSA-N | 4n8t | -4.1 |
| HYBBIBNJHNGZAN-UHFFFAOYSA-N | 1nsi | -5 |
| QAIPRVGONGVQAS-DUXPYHPUSA-N | 6a93 | -6.6 |
| QAIPRVGONGVQAS-DUXPYHPUSA-N | 3aqf | -5.6 |
| QAIPRVGONGVQAS-DUXPYHPUSA-N | 3pbl | -6.7 |
| QAIPRVGONGVQAS-DUXPYHPUSA-N | 4iar | -6.6 |
| QAIPRVGONGVQAS-DUXPYHPUSA-N | 4n8t | -6.5 |
| QAIPRVGONGVQAS-DUXPYHPUSA-N | 1nsi | -7.2 |
| XMGQYMWWDOXHJM-SNVBAGLBSA-N | 6a93 | -6.3 |
| XMGQYMWWDOXHJM-SNVBAGLBSA-N | 3aqf | -4.4 |
| XMGQYMWWDOXHJM-SNVBAGLBSA-N | 3pbl | -6 |
| XMGQYMWWDOXHJM-SNVBAGLBSA-N | 4iar | -6 |
| XMGQYMWWDOXHJM-SNVBAGLBSA-N | 4n8t | -6.4 |
| XMGQYMWWDOXHJM-SNVBAGLBSA-N | 1nsi | -7 |
| MWOOGOJBHIARFG-UHFFFAOYSA-N | 6a93 | -5.5 |
| MWOOGOJBHIARFG-UHFFFAOYSA-N | 3aqf | -4.7 |
| MWOOGOJBHIARFG-UHFFFAOYSA-N | 3pbl | -5.7 |
| MWOOGOJBHIARFG-UHFFFAOYSA-N | 4iar | -5.7 |
| MWOOGOJBHIARFG-UHFFFAOYSA-N | 4n8t | -5.4 |
| MWOOGOJBHIARFG-UHFFFAOYSA-N | 1nsi | -6.8 |
| JARKCYVAAOWBJS-UHFFFAOYSA-N | 6a93 | -4.3 |
| JARKCYVAAOWBJS-UHFFFAOYSA-N | 3aqf | -3.5 |
| JARKCYVAAOWBJS-UHFFFAOYSA-N | 3pbl | -4.1 |
| JARKCYVAAOWBJS-UHFFFAOYSA-N | 4iar | -3.9 |
| JARKCYVAAOWBJS-UHFFFAOYSA-N | 4n8t | -4.2 |
| JARKCYVAAOWBJS-UHFFFAOYSA-N | 1nsi | -5.1 |
| WVDDGKGOMKODPV-UHFFFAOYSA-N | 6a93 | -5.7 |
| WVDDGKGOMKODPV-UHFFFAOYSA-N | 3aqf | -3.9 |
| WVDDGKGOMKODPV-UHFFFAOYSA-N | 3pbl | -5.1 |
| WVDDGKGOMKODPV-UHFFFAOYSA-N | 4iar | -5.2 |
| WVDDGKGOMKODPV-UHFFFAOYSA-N | 4n8t | -5.1 |
| WVDDGKGOMKODPV-UHFFFAOYSA-N | 1nsi | -7 |
| LQGUBLBATBMXHT-UHFFFAOYSA-N | 6a93 | -8.9 |
| LQGUBLBATBMXHT-UHFFFAOYSA-N | 3aqf | -7.8 |
| LQGUBLBATBMXHT-UHFFFAOYSA-N | 3pbl | -7.5 |
| LQGUBLBATBMXHT-UHFFFAOYSA-N | 4iar | -8.8 |
| LQGUBLBATBMXHT-UHFFFAOYSA-N | 4n8t | -8.1 |
| LQGUBLBATBMXHT-UHFFFAOYSA-N | 1nsi | -10.2 |
| ISAKRJDGNUQOIC-UHFFFAOYSA-N | 6a93 | -5.6 |
| ISAKRJDGNUQOIC-UHFFFAOYSA-N | 3aqf | -4.8 |
| ISAKRJDGNUQOIC-UHFFFAOYSA-N | 3pbl | -6.1 |
| ISAKRJDGNUQOIC-UHFFFAOYSA-N | 4iar | -5.8 |
| ISAKRJDGNUQOIC-UHFFFAOYSA-N | 4n8t | -6 |
| ISAKRJDGNUQOIC-UHFFFAOYSA-N | 1nsi | -7.5 |
| YOMSJEATGXXYPX-UHFFFAOYSA-N | 6a93 | -5.9 |
| YOMSJEATGXXYPX-UHFFFAOYSA-N | 3aqf | -4.7 |
| YOMSJEATGXXYPX-UHFFFAOYSA-N | 3pbl | -6.1 |
| YOMSJEATGXXYPX-UHFFFAOYSA-N | 4iar | -5.8 |
| YOMSJEATGXXYPX-UHFFFAOYSA-N | 4n8t | -6 |
| YOMSJEATGXXYPX-UHFFFAOYSA-N | 1nsi | -7.4 |
| GFFGJBXGBJISGV-UHFFFAOYSA-N | 6a93 | -5.3 |
| GFFGJBXGBJISGV-UHFFFAOYSA-N | 3aqf | -4.7 |
| GFFGJBXGBJISGV-UHFFFAOYSA-N | 3pbl | -5.3 |
| GFFGJBXGBJISGV-UHFFFAOYSA-N | 4iar | -5.3 |
| GFFGJBXGBJISGV-UHFFFAOYSA-N | 4n8t | -4.9 |
| GFFGJBXGBJISGV-UHFFFAOYSA-N | 1nsi | -5.7 |
| ORNBQBCIOKFOEO-QGVNFLHTSA-N | 6a93 | -9.2 |
| ORNBQBCIOKFOEO-QGVNFLHTSA-N | 3aqf | -5.8 |
| ORNBQBCIOKFOEO-QGVNFLHTSA-N | 3pbl | -9 |
| ORNBQBCIOKFOEO-QGVNFLHTSA-N | 4iar | -9.4 |
| ORNBQBCIOKFOEO-QGVNFLHTSA-N | 4n8t | -6.7 |
| ORNBQBCIOKFOEO-QGVNFLHTSA-N | 1nsi | -8.5 |
| GETQZCLCWQTVFV-UHFFFAOYSA-N | 6a93 | -2.3 |
| GETQZCLCWQTVFV-UHFFFAOYSA-N | 3aqf | -2 |
| GETQZCLCWQTVFV-UHFFFAOYSA-N | 3pbl | -2.2 |
| GETQZCLCWQTVFV-UHFFFAOYSA-N | 4iar | -2.1 |
| GETQZCLCWQTVFV-UHFFFAOYSA-N | 4n8t | -2.2 |
| GETQZCLCWQTVFV-UHFFFAOYSA-N | 1nsi | -2.3 |
| KRIOVPPHQSLHCZ-UHFFFAOYSA-N | 6a93 | -6.3 |
| KRIOVPPHQSLHCZ-UHFFFAOYSA-N | 3aqf | -4.6 |
| KRIOVPPHQSLHCZ-UHFFFAOYSA-N | 3pbl | -6.3 |
| KRIOVPPHQSLHCZ-UHFFFAOYSA-N | 4iar | -5.6 |
| KRIOVPPHQSLHCZ-UHFFFAOYSA-N | 4n8t | -6.3 |
| KRIOVPPHQSLHCZ-UHFFFAOYSA-N | 1nsi | -7.6 |
| FINHMKGKINIASC-UHFFFAOYSA-N | 6a93 | -5.5 |
| FINHMKGKINIASC-UHFFFAOYSA-N | 3aqf | -4.4 |
| FINHMKGKINIASC-UHFFFAOYSA-N | 3pbl | -6 |
| FINHMKGKINIASC-UHFFFAOYSA-N | 4iar | -5.1 |
| FINHMKGKINIASC-UHFFFAOYSA-N | 4n8t | -5.3 |
| FINHMKGKINIASC-UHFFFAOYSA-N | 1nsi | -6.2 |
| XHXUANMFYXWVNG-WCQGTBRESA-N | 6a93 | -6.6 |
| XHXUANMFYXWVNG-WCQGTBRESA-N | 3aqf | -4.8 |
| XHXUANMFYXWVNG-WCQGTBRESA-N | 3pbl | -6 |
| XHXUANMFYXWVNG-WCQGTBRESA-N | 4iar | -6.7 |
| XHXUANMFYXWVNG-WCQGTBRESA-N | 4n8t | -6.7 |
| XHXUANMFYXWVNG-WCQGTBRESA-N | 1nsi | -6 |
| WMBOCUXXNSOQHM-FLIBITNWSA-N | 6a93 | -8 |
| WMBOCUXXNSOQHM-FLIBITNWSA-N | 3aqf | -5.7 |
| WMBOCUXXNSOQHM-FLIBITNWSA-N | 3pbl | -7.7 |
| WMBOCUXXNSOQHM-FLIBITNWSA-N | 4iar | -7.1 |
| WMBOCUXXNSOQHM-FLIBITNWSA-N | 4n8t | -7.6 |
| WMBOCUXXNSOQHM-FLIBITNWSA-N | 1nsi | -9.1 |
| BQOFWKZOCNGFEC-UHFFFAOYSA-N | 6a93 | -6.6 |
| BQOFWKZOCNGFEC-UHFFFAOYSA-N | 3aqf | -5 |
| BQOFWKZOCNGFEC-UHFFFAOYSA-N | 3pbl | -6.6 |
| BQOFWKZOCNGFEC-UHFFFAOYSA-N | 4iar | -6.3 |
| BQOFWKZOCNGFEC-UHFFFAOYSA-N | 4n8t | -6.4 |
| BQOFWKZOCNGFEC-UHFFFAOYSA-N | 1nsi | -7.4 |
| HJXMNVQARNZTEE-UHFFFAOYSA-N | 6a93 | -7.2 |
| HJXMNVQARNZTEE-UHFFFAOYSA-N | 3aqf | -5.6 |
| HJXMNVQARNZTEE-UHFFFAOYSA-N | 3pbl | -7.4 |
| HJXMNVQARNZTEE-UHFFFAOYSA-N | 4iar | -6.8 |
| HJXMNVQARNZTEE-UHFFFAOYSA-N | 4n8t | -7.2 |
| HJXMNVQARNZTEE-UHFFFAOYSA-N | 1nsi | -8.5 |
| MGSRCZKZVOBKFT-UHFFFAOYSA-N | 6a93 | -5.7 |
| MGSRCZKZVOBKFT-UHFFFAOYSA-N | 3aqf | -5 |
| MGSRCZKZVOBKFT-UHFFFAOYSA-N | 3pbl | -6.3 |
| MGSRCZKZVOBKFT-UHFFFAOYSA-N | 4iar | -5.7 |
| MGSRCZKZVOBKFT-UHFFFAOYSA-N | 4n8t | -6.1 |
| MGSRCZKZVOBKFT-UHFFFAOYSA-N | 1nsi | -7.4 |
| CDOSHBSSFJOMGT-UHFFFAOYSA-N | 6a93 | -5.2 |
| CDOSHBSSFJOMGT-UHFFFAOYSA-N | 3aqf | -4.3 |
| CDOSHBSSFJOMGT-UHFFFAOYSA-N | 3pbl | -5.8 |
| CDOSHBSSFJOMGT-UHFFFAOYSA-N | 4iar | -5.3 |
| CDOSHBSSFJOMGT-UHFFFAOYSA-N | 4n8t | -5.8 |
| CDOSHBSSFJOMGT-UHFFFAOYSA-N | 1nsi | -6.3 |
| FINHMKGKINIASC-UHFFFAOYSA-N | 6a93 | -5.5 |
| FINHMKGKINIASC-UHFFFAOYSA-N | 3aqf | -4.4 |
| FINHMKGKINIASC-UHFFFAOYSA-N | 3pbl | -5.9 |
| FINHMKGKINIASC-UHFFFAOYSA-N | 4iar | -5.1 |
| FINHMKGKINIASC-UHFFFAOYSA-N | 4n8t | -5.3 |
| FINHMKGKINIASC-UHFFFAOYSA-N | 1nsi | -6.2 |
| DSSYKIVIOFKYAU-UHFFFAOYSA-N | 6a93 | -5.4 |
| DSSYKIVIOFKYAU-UHFFFAOYSA-N | 3aqf | -4.6 |
| DSSYKIVIOFKYAU-UHFFFAOYSA-N | 3pbl | -6.2 |
| DSSYKIVIOFKYAU-UHFFFAOYSA-N | 4iar | -5.7 |
| DSSYKIVIOFKYAU-UHFFFAOYSA-N | 4n8t | -6 |
| DSSYKIVIOFKYAU-UHFFFAOYSA-N | 1nsi | -5.5 |
| UFWIBTONFRDIAS-UHFFFAOYSA-N | 6a93 | -7.4 |
| UFWIBTONFRDIAS-UHFFFAOYSA-N | 3aqf | -4.7 |
| UFWIBTONFRDIAS-UHFFFAOYSA-N | 3pbl | -6.5 |
| UFWIBTONFRDIAS-UHFFFAOYSA-N | 4iar | -6.5 |
| UFWIBTONFRDIAS-UHFFFAOYSA-N | 4n8t | -6.6 |
| UFWIBTONFRDIAS-UHFFFAOYSA-N | 1nsi | -9.2 |
| KBPLFHHGFOOTCA-UHFFFAOYSA-N | 6a93 | -4.3 |
| KBPLFHHGFOOTCA-UHFFFAOYSA-N | 3aqf | -3.5 |
| KBPLFHHGFOOTCA-UHFFFAOYSA-N | 3pbl | -5 |
| KBPLFHHGFOOTCA-UHFFFAOYSA-N | 4iar | -4.2 |
| KBPLFHHGFOOTCA-UHFFFAOYSA-N | 4n8t | -4.4 |
| KBPLFHHGFOOTCA-UHFFFAOYSA-N | 1nsi | -5.1 |
| QWVGKYWNOKOFNN-UHFFFAOYSA-N | 6a93 | -5.7 |
| QWVGKYWNOKOFNN-UHFFFAOYSA-N | 3aqf | -4.1 |
| QWVGKYWNOKOFNN-UHFFFAOYSA-N | 3pbl | -5 |
| QWVGKYWNOKOFNN-UHFFFAOYSA-N | 4iar | -5.3 |
| QWVGKYWNOKOFNN-UHFFFAOYSA-N | 4n8t | -5.4 |
| QWVGKYWNOKOFNN-UHFFFAOYSA-N | 1nsi | -7.1 |
| YGHRJJRRZDOVPD-UHFFFAOYSA-N | 6a93 | -4.2 |
| YGHRJJRRZDOVPD-UHFFFAOYSA-N | 3aqf | -3.3 |
| YGHRJJRRZDOVPD-UHFFFAOYSA-N | 3pbl | -3.8 |
| YGHRJJRRZDOVPD-UHFFFAOYSA-N | 4iar | -3.8 |
| YGHRJJRRZDOVPD-UHFFFAOYSA-N | 4n8t | -4 |
| YGHRJJRRZDOVPD-UHFFFAOYSA-N | 1nsi | -4.5 |
| OEYIOHPDSNJKLS-UHFFFAOYSA-N | 6a93 | -3.4 |
| OEYIOHPDSNJKLS-UHFFFAOYSA-N | 3aqf | -3.1 |
| OEYIOHPDSNJKLS-UHFFFAOYSA-N | 3pbl | -3.6 |
| OEYIOHPDSNJKLS-UHFFFAOYSA-N | 4iar | -3.2 |
| OEYIOHPDSNJKLS-UHFFFAOYSA-N | 4n8t | -3.3 |
| OEYIOHPDSNJKLS-UHFFFAOYSA-N | 1nsi | -3.1 |
| IQVQXVFMNOFTMU-UHFFFAOYSA-N | 6a93 | -8.2 |
| IQVQXVFMNOFTMU-UHFFFAOYSA-N | 3aqf | -5.8 |
| IQVQXVFMNOFTMU-UHFFFAOYSA-N | 3pbl | -7.5 |
| IQVQXVFMNOFTMU-UHFFFAOYSA-N | 4iar | -7.8 |
| IQVQXVFMNOFTMU-UHFFFAOYSA-N | 4n8t | -7.7 |
| IQVQXVFMNOFTMU-UHFFFAOYSA-N | 1nsi | -9 |
| DOIRQSBPFJWKBE-UHFFFAOYSA-N | 6a93 | -7.4 |
| DOIRQSBPFJWKBE-UHFFFAOYSA-N | 3aqf | -5 |
| DOIRQSBPFJWKBE-UHFFFAOYSA-N | 3pbl | -7.1 |
| DOIRQSBPFJWKBE-UHFFFAOYSA-N | 4iar | -6.2 |
| DOIRQSBPFJWKBE-UHFFFAOYSA-N | 4n8t | -6.6 |
| DOIRQSBPFJWKBE-UHFFFAOYSA-N | 1nsi | -8.1 |
| QAIPRVGONGVQAS-RQOWECAXSA-N | 6a93 | -6.6 |
| QAIPRVGONGVQAS-RQOWECAXSA-N | 3aqf | -5.6 |
| QAIPRVGONGVQAS-RQOWECAXSA-N | 3pbl | -6.7 |
| QAIPRVGONGVQAS-RQOWECAXSA-N | 4iar | -6.6 |
| QAIPRVGONGVQAS-RQOWECAXSA-N | 4n8t | -6.5 |
| QAIPRVGONGVQAS-RQOWECAXSA-N | 1nsi | -7.2 |
| GRWFGVWFFZKLTI-RKDXNWHRSA-N | 6a93 | -5.6 |
| GRWFGVWFFZKLTI-RKDXNWHRSA-N | 3aqf | -4.9 |
| GRWFGVWFFZKLTI-RKDXNWHRSA-N | 3pbl | -6.8 |
| GRWFGVWFFZKLTI-RKDXNWHRSA-N | 4iar | -5.7 |
| GRWFGVWFFZKLTI-RKDXNWHRSA-N | 4n8t | -6.7 |
| GRWFGVWFFZKLTI-RKDXNWHRSA-N | 1nsi | -5.4 |
| JARKCYVAAOWBJS-UHFFFAOYSA-N | 6a93 | -4.4 |
| JARKCYVAAOWBJS-UHFFFAOYSA-N | 3aqf | -3.2 |
| JARKCYVAAOWBJS-UHFFFAOYSA-N | 3pbl | -4.1 |
| JARKCYVAAOWBJS-UHFFFAOYSA-N | 4iar | -4 |
| JARKCYVAAOWBJS-UHFFFAOYSA-N | 4n8t | -4.1 |
| JARKCYVAAOWBJS-UHFFFAOYSA-N | 1nsi | -5.1 |
| FINHMKGKINIASC-UHFFFAOYSA-N | 6a93 | -5.5 |
| FINHMKGKINIASC-UHFFFAOYSA-N | 3aqf | -4.4 |
| FINHMKGKINIASC-UHFFFAOYSA-N | 3pbl | -5.9 |
| FINHMKGKINIASC-UHFFFAOYSA-N | 4iar | -5.1 |
| FINHMKGKINIASC-UHFFFAOYSA-N | 4n8t | -5.3 |
| FINHMKGKINIASC-UHFFFAOYSA-N | 1nsi | -6.2 |
| XMGQYMWWDOXHJM-SNVBAGLBSA-N | 6a93 | -6.3 |
| XMGQYMWWDOXHJM-SNVBAGLBSA-N | 3aqf | -4.7 |
| XMGQYMWWDOXHJM-SNVBAGLBSA-N | 3pbl | -6.1 |
| XMGQYMWWDOXHJM-SNVBAGLBSA-N | 4iar | -6 |
| XMGQYMWWDOXHJM-SNVBAGLBSA-N | 4n8t | -6.3 |
| XMGQYMWWDOXHJM-SNVBAGLBSA-N | 1nsi | -6.3 |
| IQVQXVFMNOFTMU-FLIBITNWSA-N | 6a93 | -8.3 |
| IQVQXVFMNOFTMU-FLIBITNWSA-N | 3aqf | -5.7 |
| IQVQXVFMNOFTMU-FLIBITNWSA-N | 3pbl | -7.6 |
| IQVQXVFMNOFTMU-FLIBITNWSA-N | 4iar | -7.6 |
| IQVQXVFMNOFTMU-FLIBITNWSA-N | 4n8t | -7.7 |
| IQVQXVFMNOFTMU-FLIBITNWSA-N | 1nsi | -9 |
| DTUQWGWMVIHBKE-UHFFFAOYSA-N | 6a93 | -5.8 |
| DTUQWGWMVIHBKE-UHFFFAOYSA-N | 3aqf | -4.2 |
| DTUQWGWMVIHBKE-UHFFFAOYSA-N | 3pbl | -5.2 |
| DTUQWGWMVIHBKE-UHFFFAOYSA-N | 4iar | -5.5 |
| DTUQWGWMVIHBKE-UHFFFAOYSA-N | 4n8t | -5.3 |
| DTUQWGWMVIHBKE-UHFFFAOYSA-N | 1nsi | -7.3 |
| OPFTUNCRGUEPRZ-UHFFFAOYSA-N | 6a93 | -6.9 |
| OPFTUNCRGUEPRZ-UHFFFAOYSA-N | 3aqf | -5.6 |
| OPFTUNCRGUEPRZ-UHFFFAOYSA-N | 3pbl | -6.3 |
| OPFTUNCRGUEPRZ-UHFFFAOYSA-N | 4iar | -6.9 |
| OPFTUNCRGUEPRZ-UHFFFAOYSA-N | 4n8t | -6 |
| OPFTUNCRGUEPRZ-UHFFFAOYSA-N | 1nsi | -6.1 |
| GYHFUZHODSMOHU-UHFFFAOYSA-N | 6a93 | -4.6 |
| GYHFUZHODSMOHU-UHFFFAOYSA-N | 3aqf | -3.6 |
| GYHFUZHODSMOHU-UHFFFAOYSA-N | 3pbl | -5 |
| GYHFUZHODSMOHU-UHFFFAOYSA-N | 4iar | -4.8 |
| GYHFUZHODSMOHU-UHFFFAOYSA-N | 4n8t | -5 |
| GYHFUZHODSMOHU-UHFFFAOYSA-N | 1nsi | -5.4 |
| OYHQOLUKZRVURQ-HZJYTTRNSA-N | 6a93 | -6 |
| OYHQOLUKZRVURQ-HZJYTTRNSA-N | 3aqf | -5.1 |
| OYHQOLUKZRVURQ-HZJYTTRNSA-N | 3pbl | -6.1 |
| OYHQOLUKZRVURQ-HZJYTTRNSA-N | 4iar | -6.1 |
| OYHQOLUKZRVURQ-HZJYTTRNSA-N | 4n8t | -6.4 |
| OYHQOLUKZRVURQ-HZJYTTRNSA-N | 1nsi | -7 |
| OIRDTQYFTABQOQ-KQYNXXCUSA-N | 6a93 | -6.5 |
| OIRDTQYFTABQOQ-KQYNXXCUSA-N | 3aqf | -6.2 |
| OIRDTQYFTABQOQ-KQYNXXCUSA-N | 3pbl | -7 |
| OIRDTQYFTABQOQ-KQYNXXCUSA-N | 4iar | -7.4 |
| OIRDTQYFTABQOQ-KQYNXXCUSA-N | 4n8t | -6 |
| OIRDTQYFTABQOQ-KQYNXXCUSA-N | 1nsi | -7.1 |
| TVMXDCGIABBOFY-UHFFFAOYSA-N | 6a93 | -4.7 |
| TVMXDCGIABBOFY-UHFFFAOYSA-N | 3aqf | -3.4 |
| TVMXDCGIABBOFY-UHFFFAOYSA-N | 3pbl | -4.6 |
| TVMXDCGIABBOFY-UHFFFAOYSA-N | 4iar | -4.4 |
| TVMXDCGIABBOFY-UHFFFAOYSA-N | 4n8t | -4.6 |
| TVMXDCGIABBOFY-UHFFFAOYSA-N | 1nsi | -5.3 |
| ZDPHROOEEOARMN-UHFFFAOYSA-N | 6a93 | -5.2 |
| ZDPHROOEEOARMN-UHFFFAOYSA-N | 3aqf | -4.4 |
| ZDPHROOEEOARMN-UHFFFAOYSA-N | 3pbl | -5.7 |
| ZDPHROOEEOARMN-UHFFFAOYSA-N | 4iar | -5.1 |
| ZDPHROOEEOARMN-UHFFFAOYSA-N | 4n8t | -5.5 |
| ZDPHROOEEOARMN-UHFFFAOYSA-N | 1nsi | -5.9 |
| ZQPPMHVWECSIRJ-KTKRTIGZSA-N | 6a93 | -5.8 |
| ZQPPMHVWECSIRJ-KTKRTIGZSA-N | 3aqf | -3.8 |
| ZQPPMHVWECSIRJ-KTKRTIGZSA-N | 3pbl | -6.3 |
| ZQPPMHVWECSIRJ-KTKRTIGZSA-N | 4iar | -5.5 |
| ZQPPMHVWECSIRJ-KTKRTIGZSA-N | 4n8t | -5.6 |
| ZQPPMHVWECSIRJ-KTKRTIGZSA-N | 1nsi | -6.3 |
| IPCSVZSSVZVIGE-UHFFFAOYSA-N | 6a93 | -5.1 |
| IPCSVZSSVZVIGE-UHFFFAOYSA-N | 3aqf | -4 |
| IPCSVZSSVZVIGE-UHFFFAOYSA-N | 3pbl | -5.4 |
| IPCSVZSSVZVIGE-UHFFFAOYSA-N | 4iar | -5.2 |
| IPCSVZSSVZVIGE-UHFFFAOYSA-N | 4n8t | -5.4 |
| IPCSVZSSVZVIGE-UHFFFAOYSA-N | 1nsi | -5.8 |
| NUJGJRNETVAIRJ-UHFFFAOYSA-N | 6a93 | -4.7 |
| NUJGJRNETVAIRJ-UHFFFAOYSA-N | 3aqf | -3.5 |
| NUJGJRNETVAIRJ-UHFFFAOYSA-N | 3pbl | -4.9 |
| NUJGJRNETVAIRJ-UHFFFAOYSA-N | 4iar | -4.8 |
| NUJGJRNETVAIRJ-UHFFFAOYSA-N | 4n8t | -4.9 |
| NUJGJRNETVAIRJ-UHFFFAOYSA-N | 1nsi | -5.1 |
| QIQXTHQIDYTFRH-UHFFFAOYSA-N | 6a93 | -5.3 |
| QIQXTHQIDYTFRH-UHFFFAOYSA-N | 3aqf | -4.3 |
| QIQXTHQIDYTFRH-UHFFFAOYSA-N | 3pbl | -5.4 |
| QIQXTHQIDYTFRH-UHFFFAOYSA-N | 4iar | -5.1 |
| QIQXTHQIDYTFRH-UHFFFAOYSA-N | 4n8t | -5.2 |
| QIQXTHQIDYTFRH-UHFFFAOYSA-N | 1nsi | -6 |
| FLIACVVOZYBSBS-UHFFFAOYSA-N | 6a93 | -5.4 |
| FLIACVVOZYBSBS-UHFFFAOYSA-N | 3aqf | -4.4 |
| FLIACVVOZYBSBS-UHFFFAOYSA-N | 3pbl | -5.6 |
| FLIACVVOZYBSBS-UHFFFAOYSA-N | 4iar | -5.4 |
| FLIACVVOZYBSBS-UHFFFAOYSA-N | 4n8t | -5.5 |
| FLIACVVOZYBSBS-UHFFFAOYSA-N | 1nsi | -5.6 |
| OPFTUNCRGUEPRZ-QLFBSQMISA-N | 6a93 | -6.5 |
| OPFTUNCRGUEPRZ-QLFBSQMISA-N | 3aqf | -4.9 |
| OPFTUNCRGUEPRZ-QLFBSQMISA-N | 3pbl | -6.2 |
| OPFTUNCRGUEPRZ-QLFBSQMISA-N | 4iar | -6.8 |
| OPFTUNCRGUEPRZ-QLFBSQMISA-N | 4n8t | -6.2 |
| OPFTUNCRGUEPRZ-QLFBSQMISA-N | 1nsi | -5.6 |
| WYKQPGOKTKQHQG-SHGJSZTHSA-N | 6a93 | -7.8 |
| WYKQPGOKTKQHQG-SHGJSZTHSA-N | 3aqf | -7.3 |
| WYKQPGOKTKQHQG-SHGJSZTHSA-N | 3pbl | -6.6 |
| WYKQPGOKTKQHQG-SHGJSZTHSA-N | 4iar | -8.4 |
| WYKQPGOKTKQHQG-SHGJSZTHSA-N | 4n8t | -7.4 |
| WYKQPGOKTKQHQG-SHGJSZTHSA-N | 1nsi | -8.7 |
| PUQSUZTXKPLAPR-UJPOAAIJSA-N | 6a93 | -7.1 |
| PUQSUZTXKPLAPR-UJPOAAIJSA-N | 3aqf | -6.4 |
| PUQSUZTXKPLAPR-UJPOAAIJSA-N | 3pbl | -7.4 |
| PUQSUZTXKPLAPR-UJPOAAIJSA-N | 4iar | -7.1 |
| PUQSUZTXKPLAPR-UJPOAAIJSA-N | 4n8t | -6.9 |
| PUQSUZTXKPLAPR-UJPOAAIJSA-N | 1nsi | -7.5 |
| KSEBMYQBYZTDHS-HWKANZROSA-N | 6a93 | -6.5 |
| KSEBMYQBYZTDHS-HWKANZROSA-N | 3aqf | -5.6 |
| KSEBMYQBYZTDHS-HWKANZROSA-N | 3pbl | -6.9 |
| KSEBMYQBYZTDHS-HWKANZROSA-N | 4iar | -6.5 |
| KSEBMYQBYZTDHS-HWKANZROSA-N | 4n8t | -6.6 |
| KSEBMYQBYZTDHS-HWKANZROSA-N | 1nsi | -7.2 |
| BVJSUAQZOZWCKN-UHFFFAOYSA-N | 6a93 | -5.3 |
| BVJSUAQZOZWCKN-UHFFFAOYSA-N | 3aqf | -4.3 |
| BVJSUAQZOZWCKN-UHFFFAOYSA-N | 3pbl | -5.2 |
| BVJSUAQZOZWCKN-UHFFFAOYSA-N | 4iar | -5.6 |
| BVJSUAQZOZWCKN-UHFFFAOYSA-N | 4n8t | -5 |
| BVJSUAQZOZWCKN-UHFFFAOYSA-N | 1nsi | -6.7 |
| DQNGMIQSXNGHOA-RCBCECLLSA-N | 6a93 | -7.2 |
| DQNGMIQSXNGHOA-RCBCECLLSA-N | 3aqf | -6.3 |
| DQNGMIQSXNGHOA-RCBCECLLSA-N | 3pbl | -7.7 |
| DQNGMIQSXNGHOA-RCBCECLLSA-N | 4iar | -6.8 |
| DQNGMIQSXNGHOA-RCBCECLLSA-N | 4n8t | -7 |
| DQNGMIQSXNGHOA-RCBCECLLSA-N | 1nsi | -7.5 |
| MWOOGOJBHIARFG-UHFFFAOYSA-N | 6a93 | -5.5 |
| MWOOGOJBHIARFG-UHFFFAOYSA-N | 3aqf | -4.7 |
| MWOOGOJBHIARFG-UHFFFAOYSA-N | 3pbl | -5.7 |
| MWOOGOJBHIARFG-UHFFFAOYSA-N | 4iar | -5.8 |
| MWOOGOJBHIARFG-UHFFFAOYSA-N | 4n8t | -5.4 |
| MWOOGOJBHIARFG-UHFFFAOYSA-N | 1nsi | -6.8 |
| DQNGMIQSXNGHOA-JXQVETIVSA-N | 6a93 | -7.5 |
| DQNGMIQSXNGHOA-JXQVETIVSA-N | 3aqf | -6.2 |
| DQNGMIQSXNGHOA-JXQVETIVSA-N | 3pbl | -7.7 |
| DQNGMIQSXNGHOA-JXQVETIVSA-N | 4iar | -6.9 |
| DQNGMIQSXNGHOA-JXQVETIVSA-N | 4n8t | -6.9 |
| DQNGMIQSXNGHOA-JXQVETIVSA-N | 1nsi | -7.7 |
