# Supplementary material for: Network Pharmacology and Metabolomics Studies on Antimigraine Mechanisms of Da Chuan Xiong Fang (DCXF)
Source: Evid Based Complement Alternat Med. 2021 Apr 20;2021:6665137. doi: 10.1155/2021/6665137 (PMC8081595; doi:10.1155/2021/6665137)
Supplement: Supplementary Materials — Supplementary S1: preparation, quality control, and HPLC of DCXF, GE, and LC. Supplementary S2: ingredients from LC and GE. Supplementary S3: QED results of GE and LC. Supplementary S4: 531 core targets. Supplementary S5: migraine genes. Supplementary S6: ARRIVE statement for animal experiments. Supplementary S7: metabolites of serum of brain tissue. Supplementary S8: all active ingredients molecular docking results. Supplementary S9: results of MCODE. Supplementary S10: effect of DCXF on serum and brain tissue metabolic profiling. Supplementary S11: gene-metabolite interaction network. Supplementary S12: GTEx RNA-seq data to verify the expression of hub genes in the brain tissues. [file 6665137.f1.zip › 6665137.f1/Supplementary S9 Results of MCODE.docx]

**Supplementary S9 Results of MCODE(FDR < 0.05)**

Genes in module A were mainly enriched adenylate cyclase activity, guanylate cyclase activity, and response to forskolin. Genes in both modules B and C were enriched in long-chain fatty acid metabolism, peroxisome proliferator-activated receptor (PPAR) signaling pathway, and cholesterol metabolism.

|  | Node IDs | Score (Density*#Nodes) | Nodes | Edges | average node degree | avg. local clustering coefficient | expected number of edges | PPI enrichment p-value |
| --- | --- | --- | --- | --- | --- | --- | --- | --- |
| Cluster1 | ADCY1, ADCY2, PTGS2, GNG2, GCG, CASP3, CTNNB1, NOS2, NPS, ALB, APOE, MAPK1, IL6, TNF, IL10, FOS, JUN, MAPK8, REN, NPY1R, HRH3, CNR1, ADCY3, ADCY6, ADCY9, ADCY8, MMP9, ADCY7, ADCY5 | 15.143 | 29 | 212 | 12.7 | 0.813 | 65 | 1.00*10^-16^ |
| Cluster2 | ACSL1, FABP1, CPT2, PPARGC1A, ACSL4, CPT1A, SLC27A2 | 6.333 | 7 | 19 | 5.43 | 0.924 | 0 | 1.00*10^-16^ |
| Cluster3 | CREBBP, CREB1, PLIN2, APOA1, SLC27A1, ACHE, FABP4, APP, CXCL8, ADORA2A, FABP3, LDLR | 4.727 | 12 | 26 | 4.33 | 0.567 | 5 | 3.26*10^-11^ |
