# Supplementary material for: Network Pharmacology and Metabolomics Studies on Antimigraine Mechanisms of Da Chuan Xiong Fang (DCXF)
Source: Evid Based Complement Alternat Med. 2021 Apr 20;2021:6665137. doi: 10.1155/2021/6665137 (PMC8081595; doi:10.1155/2021/6665137)
Supplement: Supplementary Materials — Supplementary S1: preparation, quality control, and HPLC of DCXF, GE, and LC. Supplementary S2: ingredients from LC and GE. Supplementary S3: QED results of GE and LC. Supplementary S4: 531 core targets. Supplementary S5: migraine genes. Supplementary S6: ARRIVE statement for animal experiments. Supplementary S7: metabolites of serum of brain tissue. Supplementary S8: all active ingredients molecular docking results. Supplementary S9: results of MCODE. Supplementary S10: effect of DCXF on serum and brain tissue metabolic profiling. Supplementary S11: gene-metabolite interaction network. Supplementary S12: GTEx RNA-seq data to verify the expression of hub genes in the brain tissues. [file 6665137.f1.zip › 6665137.f1/Supplenmentary S12GTEx RNA-seq data to verify the expression of hub genes in the brain tissues (1).docx]

We used the GTEx RNA-seq data to verify the expression of hub genes in the brain tissues. RNA-seq tissue data generated by the Genotype-Tissue Expression (GTEx) project was collected and analyzed multiple human post mortem tissues and reported as mean pTPM (protein-coding transcripts per million), corresponding to mean values of the different individual samples for the respective subregion. RNA-seq data from 36 of their tissue types were mapped based on RSEMv1.2.22 (v7) and the resulting TPM values have been included in the Human Protein Atlas for all corresponding genes. From Figure 11 and 12, ADCY2, APP, FOS, IL6, CREB1, ACHE, CASP3, COMT were with the highest expression among the subregions representing the brain region.


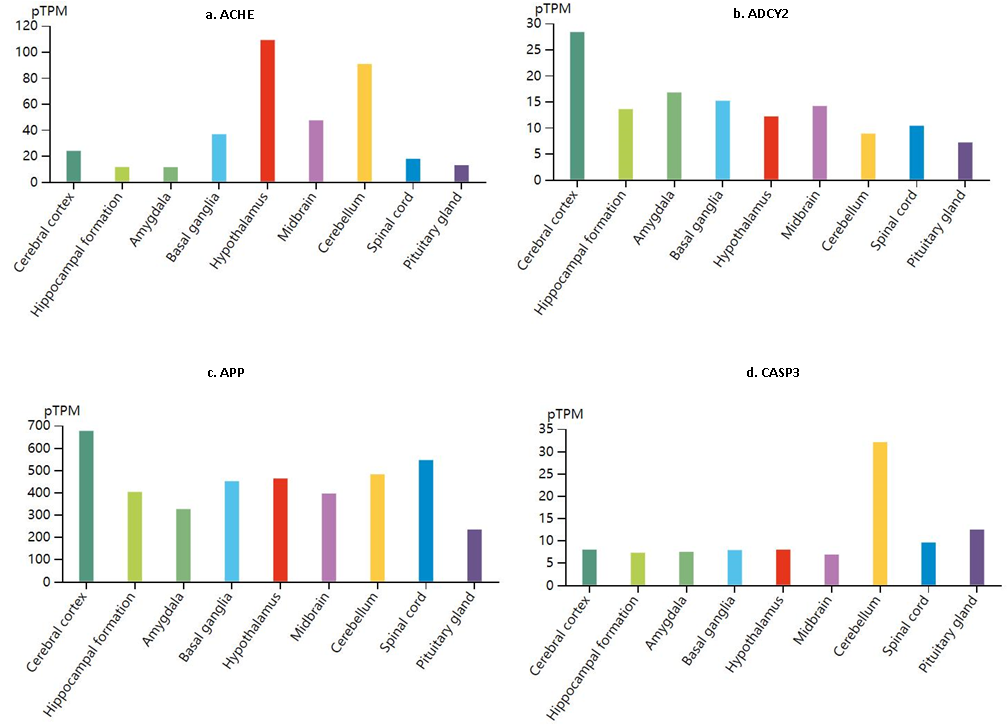


**Figure 11 GTEx RNA-seq data to verify the expression of hub genes in the brain tissues.**

**
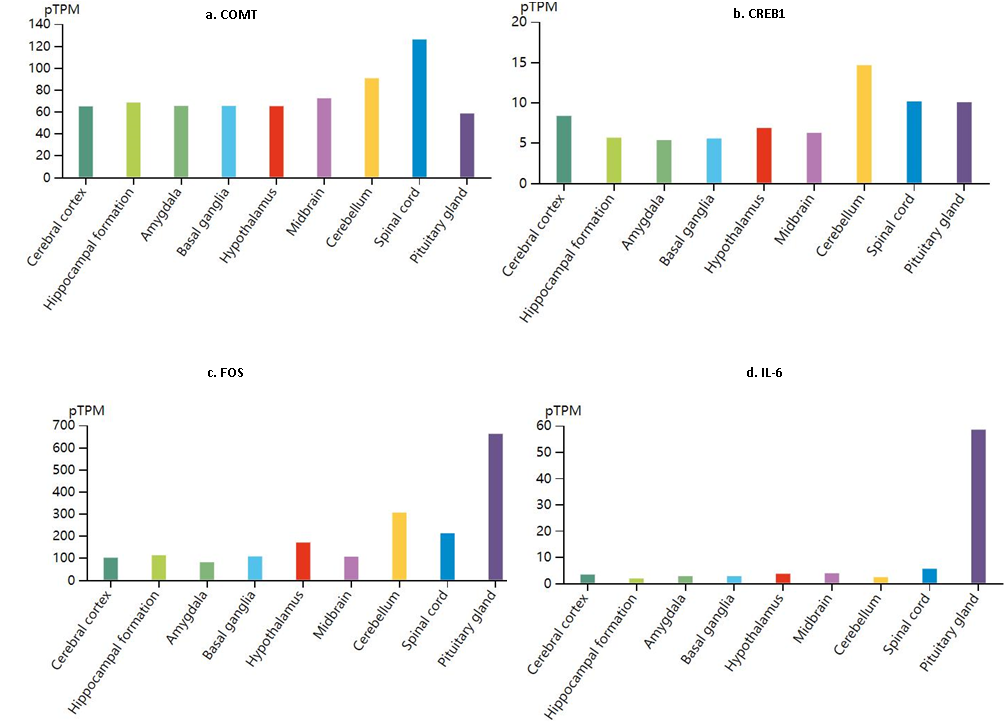
**

**Figure 12 GTEx RNA-seq data to verify the expression of hub genes in the brain tissues.**
