# Supplementary material for: Network Pharmacology and Metabolomics Studies on Antimigraine Mechanisms of Da Chuan Xiong Fang (DCXF)
Source: Evid Based Complement Alternat Med. 2021 Apr 20;2021:6665137. doi: 10.1155/2021/6665137 (PMC8081595; doi:10.1155/2021/6665137)
Supplement: Supplementary Materials — Supplementary S1: preparation, quality control, and HPLC of DCXF, GE, and LC. Supplementary S2: ingredients from LC and GE. Supplementary S3: QED results of GE and LC. Supplementary S4: 531 core targets. Supplementary S5: migraine genes. Supplementary S6: ARRIVE statement for animal experiments. Supplementary S7: metabolites of serum of brain tissue. Supplementary S8: all active ingredients molecular docking results. Supplementary S9: results of MCODE. Supplementary S10: effect of DCXF on serum and brain tissue metabolic profiling. Supplementary S11: gene-metabolite interaction network. Supplementary S12: GTEx RNA-seq data to verify the expression of hub genes in the brain tissues. [file 6665137.f1.zip › 6665137.f1/Supplenmentary S1 Preparation,Quality control and HPLC figure of DCXF, GE, and LC (1).doc]

**Supplenmentary S1 Preparation,Quality control and HPLC figure of DCXF, GE, and LC**

**1.Preparation of DCXF**

LC and GE were from Shanghai Cambridge Chinese Herbal Medicine Co.Ltd., which were identified as Ligusticum chuanxiong Hort. (Umbelliferae)and Gastrodia elata Blume (Orchidaceae) by Professor Ya-Jun CUI from Shanghai unversity of TCM, respectively (Shanghai, China).One kg of the dry powder from the LC was loaded in 12 L of 70% ethanol for 2 h and 1.5 h at 80 ◦C, respectively. The mixed extraction solutions were concentrated to 2 L and centrifuged at 5000 rpm for 15 min. The supernatant was added in HPD-100 macroporous resin with a flow rate of 1 BV/h, firstly the ultrapure water was added until the eluent remained nearly colorless,then 50% ethanol was added to 4 BV, the ethanol eluent was collected,evaporated and dried. Active components of LC and GE were made into pellets by extrusion-spheronization technology.

DCXF was self-made(with 0.95g A and 2.15g B, then dissolved in 10ml 0.9%NS), with A [LC pellets of 33% drug loading for active components (including 9.52% ferulic acid, 18.31% Senkyunolide I)] and B [GE pellets of 33% drug loading for active components (including 6.13% gastrodin)]. The Prescription dose ratios of A and B were 4: 9.

**2.Instruments and chromatographic conditions**

An Agilent Technologies 1260 HPLC system equipped with an Agilent G1329A automatic temperature control autosampler and Agilent G1314B variable wavelength detector were used. Samples were separated using a Kromasil C18 column (250 mm ×

4.6 mm, 5 μm). The mobile phase was composed of acetonitrile (A) and water containing 0.01 % HCOOH (B) and was delivered at flow rate of 0.8 mL·min-1. A gradient program was used as follows: 0-5 min: 97 % B, 5-12 min: 97-85 % B, 12-32 min: 85-75 % B, 32-37 min: 75-73 % B, 37-42 min: 73-64 % B, 42-52 min: 64-54 % B, 52-58 min: 54-22 % B, 58-62 min: 22-15 % B, 62-65 min: 15-97 % B, 65-70 min: 97 % B. The detection wavelength was 270 nm and the column temperature was set at 30 ◦C.

The precision of the chromatographic method was assessed by performing multiple injections of the same concentration. The repeat-abilities of the extraction procedure and analysis were validated by repeating the extraction and HPLC analysis procedure six times. The stability test was performed using the same sample that was stored at room temperature (about 25 ◦C) and analysed at 1, 3, 6, 9, 12, 24 h. The similarities of fingerprints parameters, the relative standard deviation (RSD) of retention time (RT) and peak area (PA) of the common peaks in all samples were used to determine method precision, repeatability and stability.

**3.Validation of the chromatography method**

The precision of the method was determined by multiple injections of the same concentration of the sample of reference preparation. The RSDs of RT and PA of selected peaks were less than 0.12 % and 3.9 %, respectively (n=6). The results for repeatability were less than 2.80 % with respect to RT and 8.62 % for PA. For the stability tests, the RSDs for RT and PA were less than 2.38 % and 4.15 %, respectively, indicating that the sample was stable over 24 h. The validation data indicated that the analytical method was specific, sensitive and stable.

**4.HPLC-UV fingerprints**

Ten batch samples of DCXF preparation were analysed shown in Fig.1. The data were generated from the reference standard fingerprint chromatogram by using the similarity evaluation system for the chromatographic fingerprint of DCXF. Fourteen characteristic peaks were identified. From Fig.2, by compared the LC characteristic effective components (LC-extract), GE characteristic effective components (GE-extract) and mixed standards, peak numbers 1, 2, 3, 4, 5, 6 were from GE, peak numbers 7, 8, 9, 10, 11, 12, 13, 14 were from LC, and peak 1 was gastrodin, peak 3 was 4-hydroxybenzyl alcohol, peak 7 was parisin, peak 8 was vanillin, peak 9 was

ferulic acid, peak 12 was senkyunolide I, peak 13 was senkyunolide H. The average similarities of the 10 randomly-selected samples were more than 0.96.

**Supplementary Figure 1.	Standard HPLC fingerprints and reference HPLC fingerprints of 10 batches DCXF**

**S1.131001;	S2.131002;	S3.131003;	S4.131004;	S5.131005;	S6.131006;**

**S7.131007; S8.131008; S9.131009; S10.131010; Reference HPLC fingerprints**

**Supplementary Figure 2.	Common peaks of standard HPLC fingerprints**

**S1. Mixture standard reference of Gastrodin, 4-Hydroxyphenethyl alcohol, Parishin, Vanillin, Ferulic acid, Senkyunolide I and Senkyunolide H; S2. Ligusticum striatum DC. Characteristic effective ingredients (LC-extract); S3. Gastrodia elata Blume Characteristic effective ingredients (GE-extract); S4. Standard HPLC fingerprints of DCXF**

**References:**

Wang, L. Shen, S. Ma, et al., “Effects of Ligusticum chuanxiong and Gastrodia elata on blood-brain barrier permeability in migraine rats,” Die Pharmazie, vol. 70, pp. 421-426, 2015.

Y. L. Hong, Y. Feng, D. S. Xu, et al., “Study on extraction and purification of active parts from Da ChuanXiong Fang for treatment of migraine,” Journal of Chinese Medica Materia, vol. 6, pp. 721-723, 2007.

Q. Wang, L. Shen, S. Y. Ma, et al., “Determination of the levels of two types of neurotransmitter and the anti-migraine effects of different dose-ratios of Ligusticum chuanxiong and Gastrodia elata,” Journal of Food and Drug Analysis, vol. 24, no. 1, pp.189-198, 2016.

Q.q. WANG, L. SHEN, J. ZHANG, et al., “Effects of active components extracted from Dachuanxiong Decoction on the expressions of CGRP and its receptors in rats with migraine,” Chinese Traditional Patent Medicine, vol. 40, pp. 14-20, 2018.

S.y. MA, L. SHEN, X. LIN, et al., “Intervention Effect of Dachuanxiong Fang Multi-component Preparation on Acute Migraine Rat Model by Urine Metabolomics,” Chinese Journal of Experience and Traditional Medicine Formula, vol. 25, pp. 101-107, 2019.

Ni N, Wang Q, Lin X, et al., “Studies on the Mechanism of Glutamate Metabolism in NTG-Induced Migraine Rats Treated with DCXF”, Evid Based Complement Alternat Med, 2019; doi: 10.1155/2019/1324797.
